# Effect of mechanical unloading on genome-wide DNA methylation profile of the failing human heart

**DOI:** 10.1172/jci.insight.161788

**Published:** 2023-02-22

**Authors:** Xianghai Liao, Peter J. Kennel, Bohao Liu, Trevor R. Nash, Richard Z. Zhuang, Amandine F. Godier-Furnemont, Chenyi Xue, Rong Lu, Paolo C. Colombo, Nir Uriel, Muredach P. Reilly, Steven O. Marx, Gordana Vunjak-Novakovic, Veli K. Topkara

**Affiliations:** 1 Division of Cardiology, Columbia University Irving Medical Center – New York Presbyterian, New York, New York, USA.; 2 Department of Biomedical Engineering, Columbia University, New York, New York, USA.

**Keywords:** Cardiology, Epigenetics, Heart failure, Noncoding RNAs

## Abstract

Heart failure (HF) is characterized by global alterations in myocardial DNA methylation, yet little is known about the epigenetic regulation of the noncoding genome and potential reversibility of DNA methylation with left ventricular assist device (LVAD) therapy. Genome-wide mapping of myocardial DNA methylation in 36 patients with HF at LVAD implantation, 8 patients at LVAD explantation, and 7 nonfailing (NF) donors using a high-density bead array platform identified 2,079 differentially methylated positions (DMPs) in ischemic cardiomyopathy (ICM) and 261 DMPs in nonischemic cardiomyopathy (NICM). LVAD support resulted in normalization of 3.2% of HF-associated DMPs. Methylation-expression correlation analysis yielded several protein-coding genes that are hypomethylated and upregulated (*HTRA1*, *FBXO16*, *EFCAB13*, and *AKAP13*) or hypermethylated and downregulated (*TBX3*) in HF. A potentially novel cardiac-specific super-enhancer long noncoding RNA (lncRNA) (*LINC00881*) is hypermethylated and downregulated in human HF. *LINC00881* is an upstream regulator of sarcomere and calcium channel gene expression including *MYH6*, *CACNA1C*, and *RYR2*. *LINC00881* knockdown reduces peak calcium amplitude in the beating human induced pluripotent stem cell–derived cardiomyocytes (hiPSC-CMs). These data suggest that HF-associated changes in myocardial DNA methylation within coding and noncoding genomes are minimally reversible with mechanical unloading. Epigenetic reprogramming strategies may be necessary to achieve sustained clinical recovery from heart failure.

## Graphical abstract

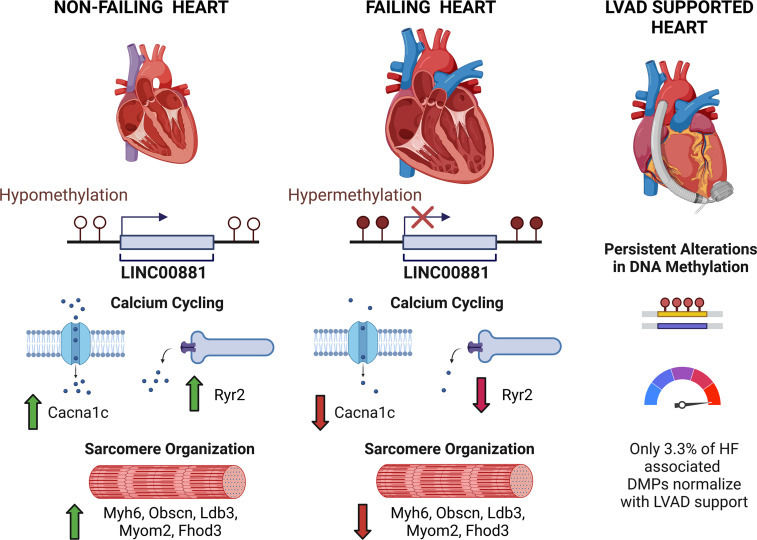

## Introduction

Heart failure (HF) is a major public health problem with more than 6 million patients affected in the United States alone (1). Despite significant progress achieved in the diagnosis and treatment of HF, the majority of patients progress to advanced stages of the disease, leading to unacceptably high rates of morbidity and mortality, even exceeding most cancers. Pharmacological management of HF has traditionally focused on targeting endogenous neurohormonal signaling cascades that are associated with disease progression as well as diuretic therapy for symptom relief (2). However, our limited mechanistic understanding of the complex HF pathophysiology does not fully explain the wide variation in disease progression and treatment response observed in this population, highlighting the need for novel molecular diagnostic and therapeutic strategies.

The failing human heart undergoes structural and functional remodeling that is accompanied by profound alterations in the myocardial transcriptome including recapitulation of the fetal gene expression program and downregulation of the genes involved in the oxidative phosphorylation pathway (3, 4). While some of these changes are common to all forms of HF, gene expression could be etiology specific and help distinguish patients with different types of HF (5, 6). Transcriptional profiling of paired myocardial samples obtained from patients with advanced HF before and after left ventricular assist device (LVAD) support demonstrate that only a small percentage of genes that are dysregulated in HF normalize with mechanical unloading of the failing human heart (7–9). While the mechanisms responsible for the persistent dysregulation of myocardial gene expression in the end-stage human HF remains unknown, emerging evidence suggests that epigenetic regulation may play important roles in transcriptional reprogramming by altering gene accessibility and TF binding to gene promoters or enhancers (10, 11).

DNA methylation is an essential epigenetic modification involving transfer of a methyl group onto the fifth position of the cytosine catalyzed by a family of DNA methyltransferases (DNMTs) and generally signals for transcription repression at the promoter sites (12). Several pathological conditions, in particular malignant transformation, have been associated with hypermethylation of specific target gene promoters as well as global hypomethylation leading to genomic instability (13). DNA methylation assays have been FDA approved for early detection of cancer and DNA demethylation agents have become standard-of-care therapy for high-risk patients with myelodysplastic syndrome (14–16). While the data is lagging for HF, several studies to date investigated genome-wide DNA methylation in the failing human heart using targeted bisulphite sequencing or chip-based approaches (17–23). However, none of these studies have examined whether or not, and if so to what extent, aberrant DNA methylation is reversible in the failing human heart. Moreover, HF-associated changes in DNA methylation of noncoding genomic elements including long noncoding RNAs (lncRNAs) remain largely unknown. Hence, in the current study, we characterize the myocardial DNA methylation profile of patients with end-stage cardiomyopathy before and after mechanical unloading with LVAD support. Furthermore, we identify what we believe to be a novel cardiac-specific– and super-enhancer–associated lncRNA (*LINC00881*) that is hypermethylated and downregulated in the failing human heart as a key regulator of calcium handling in the cardiomyocyte.

## Results

### Myocardial DNA methylation in ischemic and nonischemic cardiomyopathy.

**Figure 1:**
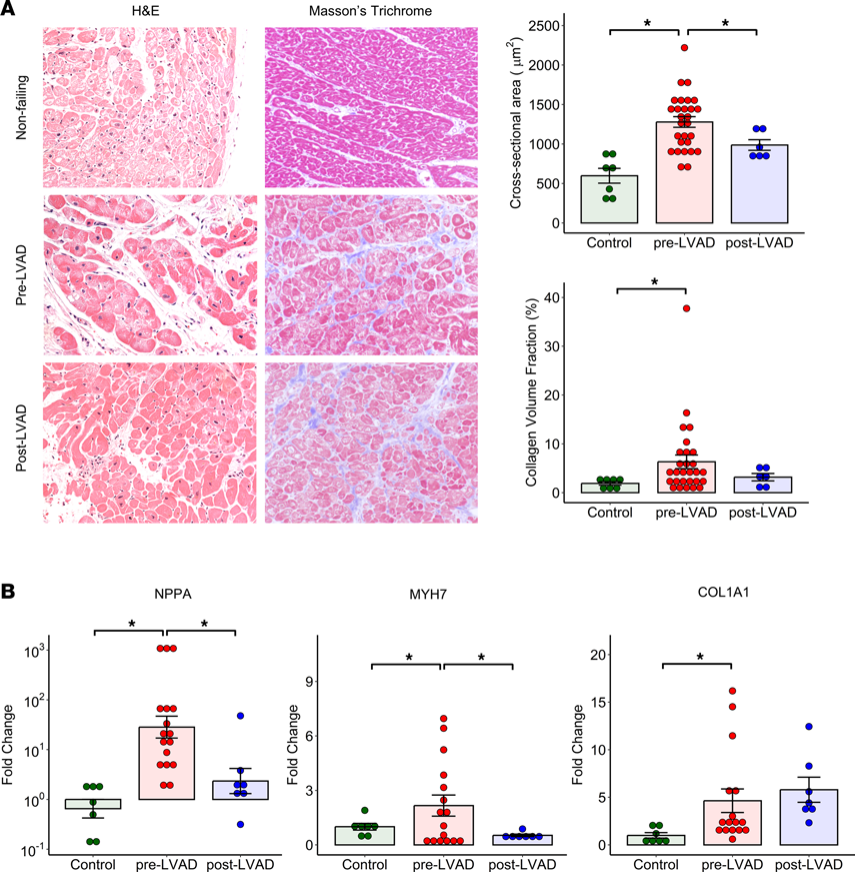
Impact of mechanical unloading on histopathology and gene expression of the failing human heart. (**A**) Cardiomyocyte CSA by H&E staining and myocardial collagen deposition by Masson’s trichrome staining (*P < 0.05, Kruskal-Wallis test with pairwise BH-corrected Wilcoxon test, *n* = 7 controls, 28 pre-LVAD, and 6 post-LVAD patients). Original magnification fields, ×20. (**B**) Myocardial *NPPA*, *MYH7*, and *COL1A1* mRNA levels by qPCR (*P < 0.05, Kruskal-Wallis test with pairwise BH-corrected Wilcoxon test, *n* = 7 controls, 17 pre-LVAD, and 7 post-LVAD patients).

A total of 36 patients with end-stage HF, i.e., 12 with ischemic cardiomyopathy (ICM) and 24 with nonischemic dilated cardiomyopathy (NICM), who underwent LVAD implantation at Columbia University Irving Medical Center and 7 nonfailing (NF) controls were included for genome-wide DNA methylation analysis. The analysis pipeline has been summarized in Supplemental Figure 1; supplemental material available online with this article; https://doi.org/10.1172/jci.insight.161788DS1 Patients with myocarditis, amyloid cardiomyopathy, restrictive/hypertrophic cardiomyopathy, and previous cardiac transplantation were excluded from the study. Demographics and clinical characteristics of patients with HF with myocardial genome-wide DNA methylation profiling at the time of LVAD implantation were summarized in Table 1. Paired post-LVAD cardiac samples were obtained from 8 patients who were bridged to heart transplantation. LVAD support resulted in significant reductions in left ventricular (LV) end-diastolic diameter, and a trend toward increased left ventricular ejection fraction (LVEF) and decreased serum B-type natriuretic peptide (BNP) levels in 8 patients (Supplemental Table 1A). Clinical information of NF cardiac donors has been provided in Supplemental Table 1B. Histopathological examination of human heart tissue samples demonstrated cardiomyocyte hypertrophy and myocardial fibrosis in pre-LVAD patients compared with NF controls consistent with HF phenotype (Figure 1A). Cardiomyocyte hypertrophy significantly regressed with LVAD support while myocardial fibrosis did not. Quantitative PCR (qPCR) analysis demonstrated upregulation of *NPPA,* *MYH7,* and *COL1A1* genes in HF compared with NF controls. LVAD support resulted in significant downregulation of *NPPA* and *MYH7* genes but not the *COL1A1* gene (Figure 1B).

**Figure 2:**
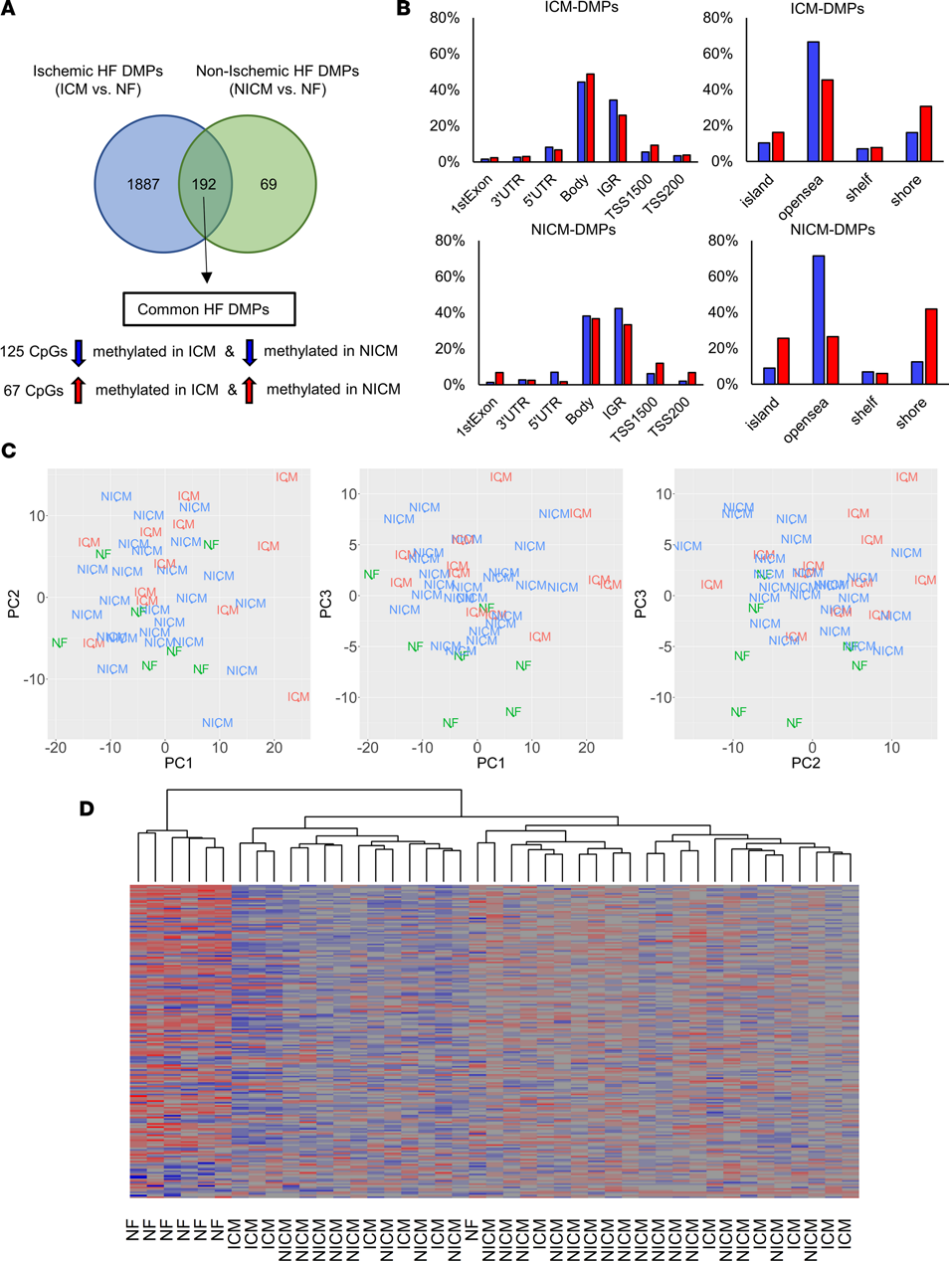
Genome-wide changes in myocardial DNA methylation based on HF etiology. (**A**) Venn diagram depicting the number of DMPs in patients with ICM versus NICM (*q* < 0.05, |Δ β |  > 10%). (**B**) Proportion of ICM and NICM DMPs by genomic location and feature. (**C**) PCA of myocardial DNA methylation grouped by etiology of HF (NF, green; ICM, red; and NICM, blue). (**D**) Heatmap clustering of subjects by methylation profile of 192 common HF DMPs.

Genome-wide DNA methylation profiling identified 2,079 differentially methylated positions (DMPs) in the myocardium of patients with ICM (ICM versus NF, *q* < 0.05; Supplemental Table 2). Of those, 625 DMPs were hypermethylated and 1,454 DMPs were hypomethylated. A total of 261 DMPs were differentially methylated in the myocardium of patients with NICM (NICM versus NF, *q* < 0.05; Supplemental Table 3). Of those, 117 DMPs were hypermethylated and 144 DMPs were hypomethylated in NICM. A total of 192 DMPs (Supplemental Table 4) were common to both patients with ICM and NICM (Figure 2A). All of these “common HF DMPs” were either concordantly hypomethylated (*n* = 125) or concordantly hypermethylated (*n* = 67) in ICM and NICM.

Characterizing the location of DMPs within gene regions, for ICM and NICM similarly, a higher proportion of probes located within the transcription start site were hypermethylated than hypomethylated, whereas intergenic region probes were more likely to be hypomethylated than hypermethylated (Figure 2B). DMPs within open sea regions were more likely to be hypomethylated whereas DMPs within CpG islands and associated shores were more likely to be hypermethylated in both ICM and NICM (Figure 2B). A total of 17.9% of DMPs mapped to promoter regions (TSS200, TSS1500, and 5′UTR) in patients with ICM compared with 22.2% of DMPs in patients with NICM. Principal component analysis (PCA) of genome-wide methylation levels demonstrates clustering of the NF versus failing samples, however, did not clearly separate between subjects with ischemic versus nonischemic etiology (Figure 2C). Heatmap of methylation level Z scores of 192 common HF DMPs across NF healthy controls and HF samples with unbiased hierarchical heatmap clustering separated the NF controls from the failing heart DMPs but did not separate ICM from NICM (Figure 2D).

### Minimal reversibility of myocardial DNA methylation with LVAD support.

**Figure 3:**
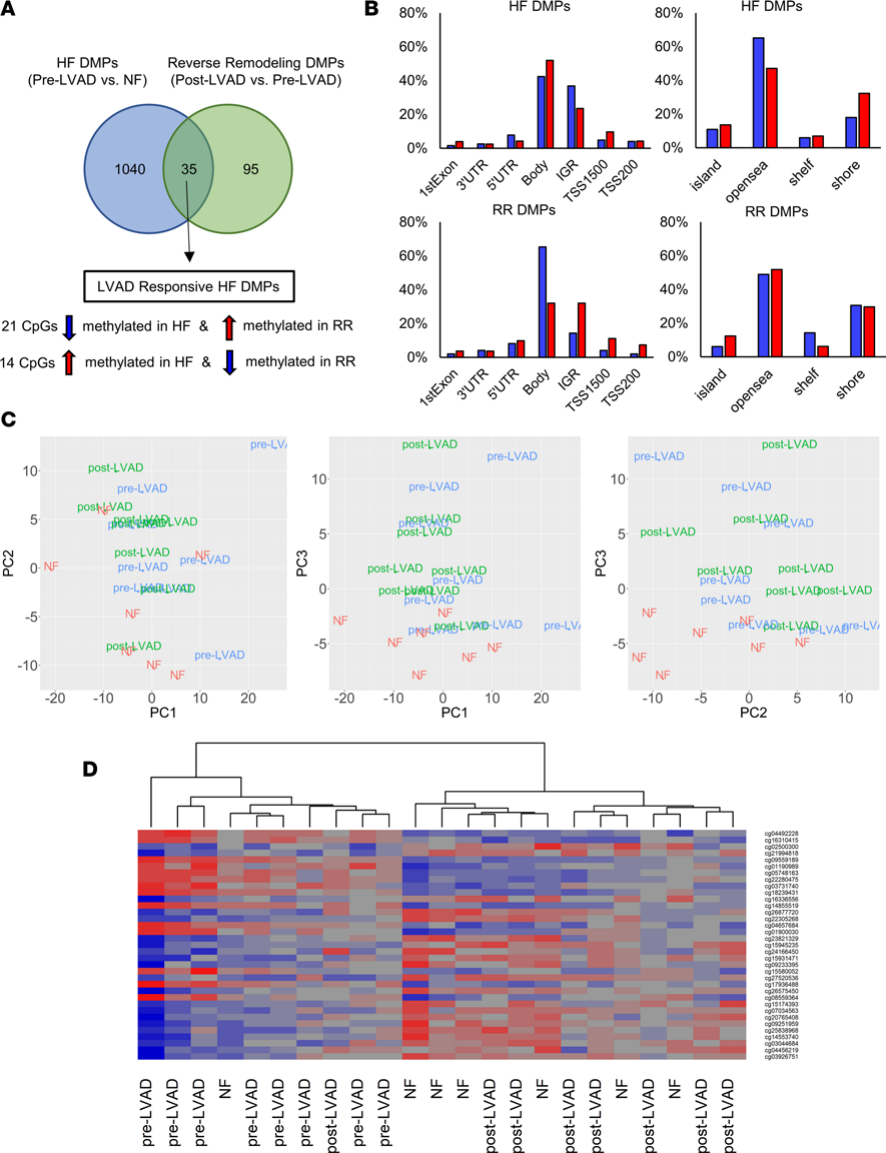
Impact of mechanical unloading on myocardial DNA methylation profile. (**A**) Venn diagram depicting the number of DMPs in HF (pre-LVAD versus NF) versus RR (post-LVAD versus pre-LVAD) (*q* < 0.05, |Δ β|  > 10%). (**B**) Proportion of HF and RR DMPs by genomic location and feature. (**C**) PCA of myocardial DNA methylation before and after LVAD support (NF, red; pre-LVAD, blue; and post-LVAD, green). (**D**) Heatmap clustering of subjects by 35 LVAD responsive HF DMPs.

Patterns of myocardial DNA methylation were analyzed in paired myocardial samples obtained from 8 patients before and after LVAD support. A total of 1,075 CpG sites were differentially methylated in the failing myocardium compared with NF (pre-LVAD versus NF, *q* < 0.05; Supplemental Table 5). In contrast, only 130 CpG sites were differentially methylated with LVAD support (post-LVAD versus pre-LVAD, *q* < 0.05; Supplemental Table 6). Only 35 CpG sites (Supplemental Table 7) were common in HF and reverse remodeling (RR) (Figure 3A), all of which were methylated in opposite directions.

A higher proportion of DMPs located within gene bodies and transcription start sites were hypermethylated while DMPs located within intergenic regions (IGR) were hypomethylated in HF. Conversely, a higher proportion of probes located within gene bodies and transcription start sites were hypomethylated while DMPs located within IGRs were hypermethylated in RR, suggesting an opposite trend between HF and RR (Figure 3B). PCA of genome-wide methylation levels demonstrated clustering of the NF versus pre-LVAD samples, but not pre- versus post-LVAD samples, suggesting only minor changes in global DNA methylation with mechanical unloading (Figure 3C). Heatmap of methylation level Z scores of 35 LVAD-responsive HF DMPs across NF and paired LVAD samples with unbiased hierarchical clustering demonstrated grouping of post-LVAD samples with 8 out of 9 NF control samples (Figure 3D), confirming LVAD-induced changes in DNA methylation toward the NF state in these genomic positions.

### Integrated analysis of DNA methylation with gene expression in the failing human heart.

**Figure 4:**
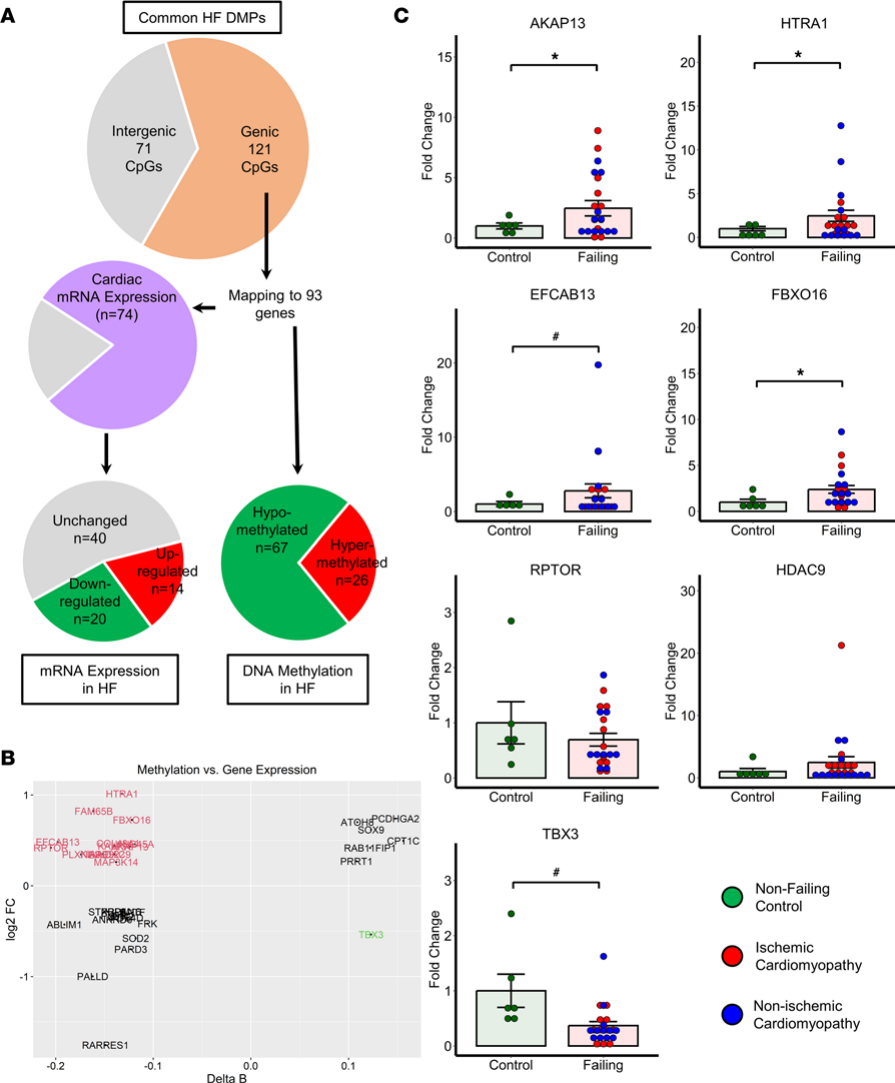
Correlation of DNA methylation with gene expression in human HF. (**A**) Differential methylation and gene expression of 192 common HF DMPs. (**B**) Methylation versus gene expression correlation plot. (**C**) qPCR validation of candidate genes that are epigenetically regulated in independent samples obtained from patients with HF. *P < 0.05, ^#^*P* < 0.10; unpaired 2-tailed *t* test; *n* = 6 (control) and 23 (failing). Data shown as mean ± SEM.

The classical paradigm of promoter DNA methylation as a transcriptional silencing mechanism has recently been challenged with growing lines of evidence suggesting that DNA hypermethylation could also result in transcriptional activation (24–26). Accordingly, we assessed the relationship between differentially methylated CpG sites and changes in gene expression levels using a large publicly available transcriptional data set obtained from patients with HF (5). Out of 192 common HF DMPs, 121 CpG sites map to 93 protein-coding genes (Figure 4A). Of those, 74 genes were expressed in the myocardium (RPKM > 1), and 34 were differentially expressed in human HF. When changes in DNA methylation were correlated with the changes in gene expression, 14 genes were hypomethylated and transcriptionally upregulated, 13 genes were hypomethylated and transcriptionally downregulated, 6 genes were hypermethylated and transcriptionally upregulated, and 1 gene was hypermethylated and transcriptionally downregulated (Figure 4B and Table 2). Using independent cardiac tissue samples obtained from patients with end-stage ICM and NICM, we validated upregulation of *AKAP13* (fold change [FC] = 2.98)*, HTRA1* (FC = 1.52)*, EFCAB13* (FC = 2.86), and *FBXO16* (FC = 1.92) as well as downregulation of *TBX3* (FC = 0.39) in the failing human hearts by qPCR analysis, while changes in *RPTOR* and *HDAC9* transcripts did not reach statistical significance (Figure 4C).

### Super-enhancer associated LINC00881 is downregulated in the failing human heart.

**Figure 5:**
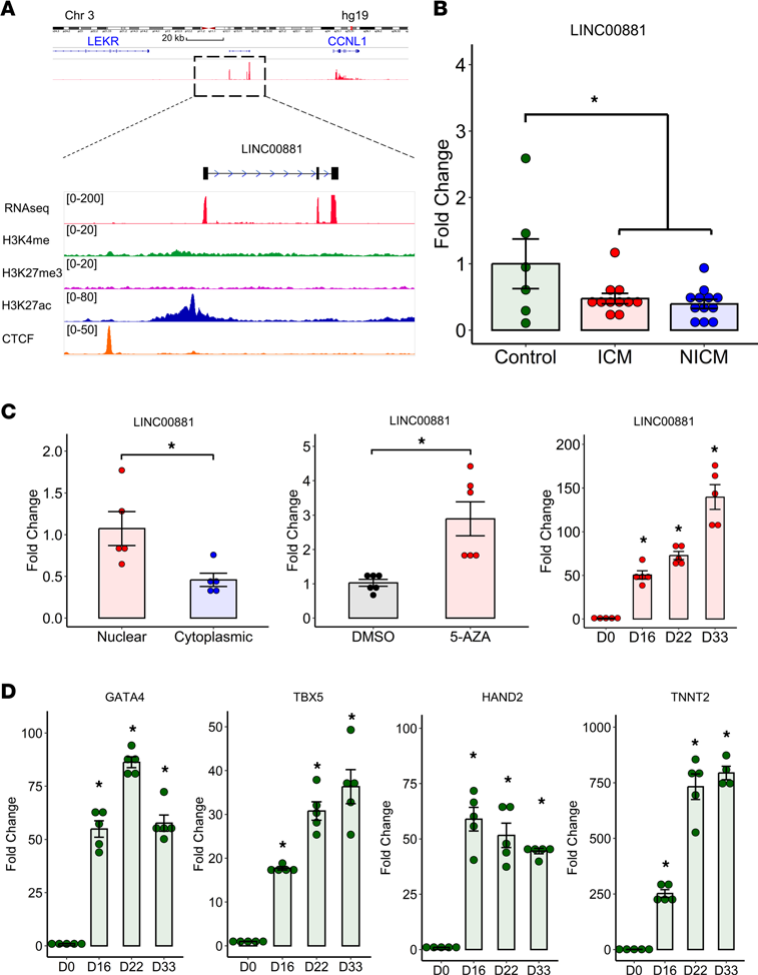
*LINC00881* is a cardiomyocyte lineage-specific super-enhancer lincRNA. (**A**) Position, expression, and epigenetic regulation of the *LINC00881* locus in the NF human heart. (**B**) Expression of *LINC00881* in patients with ICM and NICM cardiomyopathy by qPCR (*P < 0.05, 1-way ANOVA with Fisher’s post hoc test; *n* = 6 controls, 11 ICM, and 12 NICM). (**C**) Expression of *LINC00881* in the nuclear versus cytoplasmic fractions (*P < 0.05, unpaired 2-tailed *t* test, *n* = 6/group), with or without treatment with 20 nM 5-AZA (*P < 0.05, unpaired 2-tailed *t* test, *n* = 6/group), in hiPSC-CMs (*P < 0.05, 1-way ANOVA with Fisher’s post hoc test compared with day 0, *n* = 5/group). (**D**) Expression levels of *LINC00881* and cardiac transcription factors during hiPSC-CM differentiation (*P < 0.05, 1-way ANOVA with Fisher’s post hoc test compared with day 0, *n* = 5/group). Data shown as mean ± SEM.

Common HF and LVAD-responsive HF DMPs located in intergenic regions were screened for the presence of overlapping noncoding RNAs using GENCODE and NONCODE data sets. We identified a novel long intergenic nonprotein coding RNA (*LINC00881*) located approximately 9 kb upstream of a significantly hypermethylated CpG site (cg01535205) (Supplemental Table 8). *LINC00881* is highly and exclusively expressed in the human myocardium according to GTEx, which made it an interesting candidate for further exploration in the context of epigenetic modification of the failing heart (Supplemental Figure 2). Interestingly, *LINC00881* and cg01535205 are located within a cardiac super-enhancer region (Supplemental Figure 3). RNA-Seq and CHIP-Seq tracks confirm high expression of *LINC00881* in the adult NF human heart as well as the presence of active chromatin marks in this region including H3K27ac (Figure 5A). qPCR of *LINC00881* in independent HF samples confirmed that this intergenic RNA is downregulated in ICM and NICM compared with myocardium from healthy controls (Figure 5B). *LINC00881* is largely restricted to the nuclear compartment as opposed to cytoplasmic in hiPSC-CMs (Figure 5C). Inhibition of DNA methyltransferase using 5-Azacytidine (5-AZA) resulted in significant upregulation of *LINC00881* transcript levels in the beating hiPSC-CMs, suggesting epigenetic regulation of *LINC00881* in cardiomyocytes (Figure 5C). *LINC00881* expression was significantly upregulated during differentiation of hiPSC-CMs, in parallel with upregulation of transcription factors including *GATA4*, *HAND2*, and *TBX5*, confirming its role as a cardiomyocyte lineage-specific super-enhancer lncRNA (Figure 5D).

**Figure 6:**
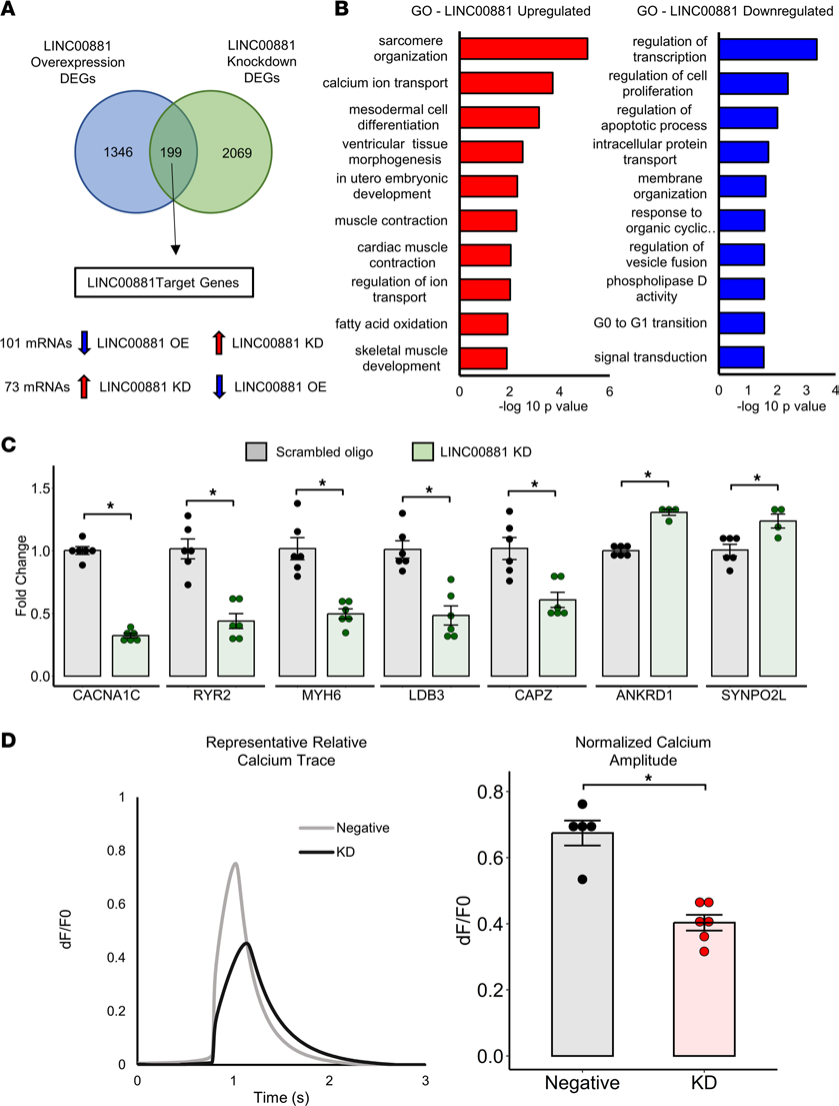
*LINC00881* is an essential regulator of cardiomyocyte calcium cycling. (**A**) Venn diagram depicting the number of DEGs in hiPSC-CMs with *LINC00881* plasmid-based overexpression or GapmeR-mediated knockdown. (**B**) Gene Ontology analysis of gene targets that are positively or negatively regulated by *LINC00881* for biological process. (**C**) qPCR validation of LINC00881 target genes (*P < 0.05, unpaired 2-tailed *t* test, *n* = 4–6/group). (**D**) Representative relative calcium traces in beating hiPSC-CMs with *LINC00881* versus scrambled oligonucleotide knockdown with averaged normalized calcium amplitude (*P < 0.05, unpaired 2-tailed *t* test, *n* = 5–6/group). Data shown as mean ± SEM.

To explore the mechanistic basis of *LINC00881* dysregulation in human HF, we used plasmid-mediated overexpression and GapmeR-based knockdown of *LINC00881* in the beating hiPSC-CMs (Supplemental Figures 4 and 5). RNA-Seq identified 1,545 genes that were differentially expressed with *LINC00881* overexpression (Supplemental Table 9) and 2,268 genes that were differentially expressed with *LINC00881* knockdown (*P* value cutoff < 0.05) (Supplemental Table 10). Among 199 common genes that were differentially regulated in both *LINC00881* overexpression and *LINC00881* knockdown models, 174 (87.4%) were regulated in opposite directions, including 73 genes that are positively regulated and 101 genes that are negatively regulated by *LINC00881* (Figure 6A and Supplemental Table 11). Gene Ontology analysis of gene targets that are positively regulated by *LINC00881* were enriched in sarcomere organization (*MYH6, OBSCN, LDB3, MYOM2,* and *FHOD3*), calcium ion transport (*CACNA1C, CACNA1D, RYR2, CAMK2A,* and *ANXA6*), ventricular tissue morphogenesis (*FGFR2, MYH6,* and *TNNI1*), and fatty acid β-oxidation (*MECR, ACAD10,* and *PPARD*) pathways (Figure 6B). Gene Ontology analysis of gene targets that are negatively regulated by *LINC00881* were enriched in transcription from RNA II polymerase promoter (*CEBPG*, *KLF5*, *KLF10*, *SMAD5*, *ATF1*, *ATF3*, and *ANKRD1*) and regulation of apoptotic process (*BCLAF1, DNAJA1, GNA13, MCL1, ANRKRD1, PHLDA1,* and *SIRT1*) pathways. *LINC00881* regulation of select sarcomere and calcium channel target genes were validated in hiPSC-CMs by qPCR with or without *LINC00881* knockdown (Figure 6C). To determine whether *LINC00881* regulation of sarcomere and calcium channel genes have functional relevance in the heart, we measured calcium transients in beating hiPSC-CMs treated with GapmeRs targeting *LINC00881* versus scrambled control oligonucleotide. *LINC00881* knockdown resulted in significant reductions in the peak calcium amplitude in hiPSC-CMs (Figure 6D).

**Figure 7:**
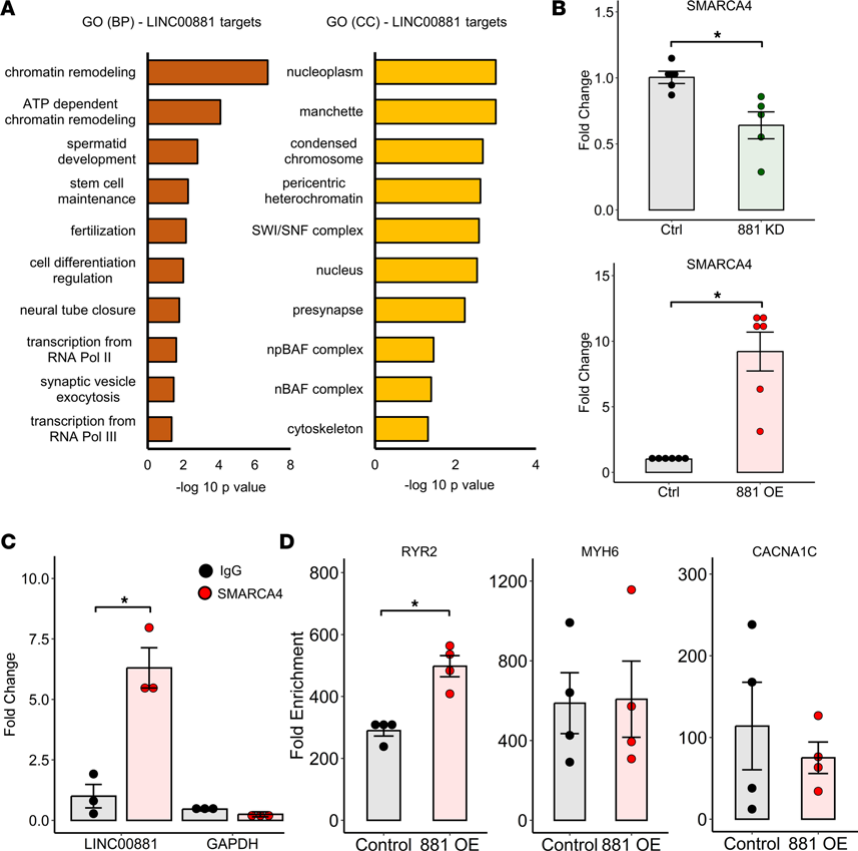
*LINC00881* regulates chromatin remodeling in human cardiomyocytes. (**A**) Gene Ontology analysis of putative LINC00881 protein targets for biological process (BP) and cellular component (CC) subontologies. (**B**) *SMARCA4* mRNA levels with *LINC00881* knockdown or *LINC00881* overexpression in hiPSC-CMs by qPCR (*P < 0.05, unpaired 2-tailed *t* test, *n* = 5–6/group). (**C**) RNA IP of *LINC00881* or *GAPDH* transcripts using *SMARCA4 Ab* versus IgG control by qPCR in hiPSC-CMs (*P < 0.05, unpaired 2-tailed *t* test, *n* = 3/group, data representative of 3 experiments). (**D**) Chromatin accessibility of *MYH6, RYR2,* and *CACNA1C* promoters with or without *LINC00881* overexpression in hiPSC-CMs by qPCR (*P < 0.05, unpaired 2-tailed *t* test, *n* = 4/group).

To gain further insights into *LINC00881* regulation of cardiomyocyte gene expression, we performed in silico target prediction analysis using RNAct database and identified 54 putative *LINC00881* interacting proteins (Supplemental Table 12). Candidate *LINC00881* targets were significantly enriched for chromatin remodeling pathway proteins including *BICRA, CECR2, ERCC6, SMARCA2, SMARCA4, BAZ1A, BAZ1B,* and *RSF1* (Figure 7A). RNA-Seq analysis in hiPSC-CMs suggested borderline significance for differential regulation of *SMARCA4* by *LINC00881* overexpression and knockdown*,* but not the other putative targets. *LINC00881* regulation of *SMARCA4* was validated in hiPSC-CMs by qPCR (Figure 7B). In vitro RNA IP in hiPSC-CMs showed that *LINC00881* but not *GAPDH* was coprecipitated by *SMARCA4* (Figure 7C). Chromatin accessibility experiment in hiPSC-CMs demonstrated a significant increase in *RYR2* promoter accessibility with *LINC00881* overexpression in hiPSC-CMs (Figure 7D).

## Discussion

The present study utilized bead array technology for high-density genome-wide mapping of DNA methylation in the failing human heart before and after LVAD support. Our analysis identified myocardial DNA methylation patterns that are associated with reciprocal regulation of gene expression in patients with ICM and NICM. In addition to providing the most comprehensive mapping of etiology-specific changes in myocardial DNA methylation to date, we show, for what we believe to be the first time, that mechanical unloading with LVAD is associated with an incomplete normalization of the myocardial DNA methylation profile, suggesting that HF-related epigenetic alterations could be persistent. Moreover, our analysis identified a potentially novel cardiac-specific super-enhancer lncRNA gene (*LINC00881*), which is hypermethylated and downregulated in the failing human heart, as an essential regulator of cardiomyocyte calcium cycling.

### DNA methylation in human HF.

Consistent with previous reports, we found that the majority of DMPs in end-stage human HF had a reduction in DNA methylation levels, suggesting a global hypomethylation similar to what has been demonstrated in cancer biology (17, 18, 20, 27). Movassagh et al. demonstrated that HF-related differential methylation in CpG islands was predominantly located in gene promoters and gene bodies, but not in intergenic or 3′UTRs (17). Our analysis expands on this observation and demonstrates a large number of intergenic positions that are also differentially methylated, including a subset mapping to the noncoding genome. Our etiology-specific analysis identified 192 DMPs that were common to patients with ICM (2,079 DMPs) and NICM (261 DMPs), similar to a recent study by Glezeva et al., which identified 13 common HF DMPs from patients with ICM (51 DMPs) and NICM (118 DMPs) (20). Overlapping DNA methylation profiles in patients with ICM and NICM support a convergent “final common pathway” of gene expression that are found in all forms of HF irrespective of the inciting event. Our analysis showed that hypomethylated CpG sites, particularly in ICM, were more likely to be within the gene body or intergenic regions as opposed to transcription start sites, which is consistent with a prior study by Pepin et al. demonstrating a relative hypermethylation of promoter-associated CGIs in patients with ICM (28). Of the 93 common HF DMPs mapping to a protein-coding gene in the current study, 25 were also found to be differentially methylated in at least 1 previous study validating our analytical approach (18, 22, 23). Interestingly, we did not find differential DNA methylation for HF marker genes with the exception of *NPPB* promoter, which was significantly hypomethylated in NICM and ICM samples with a Δ β value below the detection cutoff level of 10%. Hypomethylation of natriuretic peptide gene promoters in patients with HF has been previously demonstrated by some (22, 23), but not all genome-wide DNA methylation studies (17, 18, 20). A plausible explanation for this discrepancy is that most studies to date used whole myocardium for DNA methylation profiling which could dilute cardiomyocyte-specific epigenetic signals. It is also possible that alternative epigenetic modifications such as histone modification may play a role in transcriptional regulation of HF marker genes (29).

### Effect of mechanical unloading with LVAD on myocardial DNA methylation patterns.

Mechanical unloading with LVAD is associated with favorable changes in the biology of the failing cardiomyocyte, including regression of myocyte hypertrophy, improvement in excitation-contraction coupling, and downregulation of the fetal gene expression, also termed RR, which results in normalization of cardiac function in a small number of patients, allowing for LVAD explantation (30–33). Transcriptional studies of paired myocardial samples obtained from patients with end-stage HF before and after LVAD support demonstrate that only less than 5% of HF-related transcripts normalize with LVAD support (7, 9). Consistent with these observations, we found that only 3.2% of HF-related DMPs were reverse methylated with LVAD support, which may in part explain persistent transcriptional dysregulation and the low incidence of myocardial recovery in patients supported by LVAD. Since DNA methylation is a reversible phenomenon, these findings raise the possibility that epigenetic modulation may be necessary to normalize the HF transcriptome and achieve myocardial recovery. In support of this hypothesis, inhibition of DNA methylation using 5-aza-2’-deoxycytidine rescued a HF phenotype in a rat model of norepinephrine-induced cardiac hypertrophy (34). Mice with cardiomyocyte-specific deletion of *Dnmt3b* develop cardiomyopathy with sarcomeric disarray and interstitial fibrosis (35). Similarly, differences in myocardial DNA methylation among mouse strains has been shown to determine susceptibility to cardiac hypertrophy following isoproterenol treatment (36). Taken together, these findings suggest that epigenetic reprogramming may have therapeutic relevance in HF and additional research is necessary to elucidate the mechanistic basis of this approach.

### Common HF DMPs with reciprocal changes in gene expression.

Our analysis identified a subset of differentially methylated protein-coding genes, which were transcriptionally regulated in the opposite direction of methylation in HF. Among the upregulated genes were *HTRA1*, *FAM65B*, *UNC45A*, *KALRN*, *AKAP13*, *RPTOR*, and *HDAC9*, which have been previously implicated in maladaptive hypertrophy. *HTRA1* has been identified as part of a candidate gene signature correlated with cardiomyopathies in a gene correlation network analysis model and its mRNA expression is upregulated 6.9 fold in DCM (37, 38). *PINK1* dependent phosphorylation of *FAM65B* attenuates ischemia reperfusion injury by suppressing autophagy (39). The *UNC45A* gene has been characterized as a potential de novo mosaic variant in sporadic cardiomyopathy (40). *KLRN* is a Rho Guanine Nucleotide Exchange Factor (*GEF*), which is downregulated in ICM and NICM hearts (41). A-Kinase Anchoring Protein 13 (*AKAP13*) promotes downstream hypertrophic gene expression, mediated at least in part via *HDAC5* phosphorylation and *MEF2*-mediated transcription in a transverse aortic constriction (TAC) model of cardiac hypertrophy (42). Genetic deficiency of *RPTOR*, regulatory-associated protein of mTOR Complex 1 (mTORC1), leads to reduction mTORc1 activity and development of dilated cardiomyopathy in mice (43). Mice lacking *HDAC9* are sensitized to hypertrophic signals and exhibit stress-dependent cardiomegaly (44). *miR-21* levels are increased selectively in fibroblasts of the failing heart (45). In vivo silencing of *miR-21* has been shown to attenuate cardiac dysfunction (45, 46).

*TBX3* was the only protein-coding gene common to ICM and NICM with DNA hypermethylation and transcriptional downregulation in our analysis. *TBX3* is located within the 12q24.21 locus, which harbors several other genes that have previously been linked to cardiomyopathies. Meder et al. identified this gene locus to be differentially methylated in patients with NICM, validating our observation (23). Genetic variation in *TBX3* was associated with LV mass in healthy Japanese population, highlighting potential implications in cardiac hypertrophy; however, precise mechanisms warrant further investigation (47). In addition, we identified and validated several genes that were hypomethylated and upregulated in the failing human heart with previously unknown links to HF, including *FXBO16*, *EFCAB13*, *COL18A1*, *PLXNA2*, *BRE*, *KIAA0922*, and *MAP3K14*. Additional research is warranted to investigate the function of these genes in the HF pathophysiology.

### Role of LINC00881 in human HF.

Our genome-wide screening identified a novel lincRNA with epigenetic and transcriptional dysregulation in human HF. *LINC00881* is transcribed from a cardiac-specific super-enhancer region with abundant expression levels in the adult human heart. We show that this super-enhancer region is hypermethylated, which is associated with downregulation of *LINC00881* gene expression in patients with ICM and NICM. In cardiomyocytes, DNA demethylation treatment also resulted in upregulation of *LINC00881* transcript levels. To date, very little has been known regarding the role and function of *LINC00881* except that it is expressed in cardiomyocytes and regulated by a *GATA4* responsive super-enhancer element (48, 49). *LINC00881* (NR_034008) was listed among significantly downregulated transcripts in the failing human heart in 2 independent transcriptomic publications, validating our observation (5, 50). Our in vitro work in beating hiPSC-CMs expands on the role of LINC00881 in human HF and suggests that it is an essential regulator of cardiomyocyte calcium cycling and an upstream transcriptional regulator of several key calcium channel and sarcomere organization genes, including *CACNA1C*, *RYR2*, and *MYH6*. In silico target prediction and chromatin accessibility data suggest that transcriptional regulation of *LINC00881* target genes is in part mediated by chromatin remodeling, which is a common mechanism for lncRNAs that are localized in the nucleus. Furthermore, we identify *SMARCA4* as a *LINC00881* interacting protein in hiPSC-CMs. *SMARCA4* belongs to ATP-dependent chromatin remodeling complex SWI/SNF and plays critical roles during cardiac differentiation and development (51, 52). Initial studies in mice have demonstrated that *Smarca4* is turned off in adult cardiomyocytes and reactivated upon cardiac stress to regulate myosin heavy chain isoform switching as a maladaptive response (53). Emerging data, however, indicate that depletion of *Smarca4* levels in postnatal cardiomyocytes by adenoviral-based siRNA knockdown or dual genetic KO strategy with *Smarca2* causes lethal cardiomyopathy in mice (54, 55). Furthermore, single-cell transcriptome profiling from the normal adult human heart demonstrated that *SMARCA4* was highly expressed in human atrioventricular cardiomyocytes (56). Expression of *SMARCA4* and its occupancy at target gene promoters were significantly decreased in human HF (55, 57), suggesting distinct roles for this chromatin remodeler in the adult human heart under basal and stress conditions.

Limitations of the study include unknown confounders that may influence DNA methylation patterns in the human myocardium. Cardiac tissue is composed of a multitude of cell types, including cardiomyocytes, fibroblasts, endothelial cells, and immune cells, which likely have distinct methylation profiles. Since our study utilized myocardial DNA for profiling, it does not address cell-specific alterations in the DNA methylation profile (19). We used bead array technology for methylome profiling and were restricted to genomic sites that were included in the platform inferring a selection bias. Epigenetic modifications other than DNA methylation such as histone modifications, which may have an impact on transcriptional dysregulation in HF, were not assessed in this study.

In conclusion, HF is associated with common and distinct alterations in DNA methylation in ICM versus NICM. Mechanical unloading with LVAD fails to normalize the majority of HF-related DNA methylation, which remain persistently dysregulated. Differential DNA methylation regulates expression of both protein-coding and noncoding transcripts in the failing human heart with previously uncharacterized functions. Among these, *LINC00881* is a cardiac super-enhancer lncRNA, which is an essential regulator of cardiomyocyte calcium cycling. These findings suggest that epigenetic-targeted therapies could be necessary to normalize the dysregulated transcriptome in the failing human myocardium and help achieve sustained clinical recovery from HF.

## Methods

### Study subjects.

Myocardial DNA methylation profiling was performed with 36 patients with end-stage HF (12 ICM and 24 NICM, *n* = 36 pre-LVAD) who underwent LVAD implantation at Columbia University Irving Medical Center, including 8 patients with paired cardiac tissue at the time of heart transplantation (*n* = 8 post-LVAD) and NF control cardiac tissue (*n* = 7, NF deceased donors) obtained from the National Disease Research Interchange (NDRI).

### Genome-wide DNA methylation profiling.

LV apical myocardial tissue samples were washed in ice-cold saline (0.9% NaCl) and stored in liquid nitrogen until DNA and RNA were extracted. DNA was extracted using a DNA Kit (Qiagen). Purity and concentration were determined using a Bioanalyzer (Agilent Technologies). For DNA methylation profiling, DNA was bisulfite converted using a commercially available kit (Zymo Research). Illumina Infinium Human Methylation 450K Bead Chip and EPIC Bead Chip were used for genome-wide profiling of DNA methylation (58). The EPIC chip measures over 850,000 methylation sites, with high reproducibility compared with the previous 450k chip (59). After whole genome DNA amplification, the samples were applied following the Illumina Infinium Human Methylation DNA chip manufacturer protocols (Illumina). Chips were analyzed using the Illumina Hi-Scan system at the McDonnell Genome Institute (MGI) at Washington University.

### Bioinformatics analysis.

Integrated analysis of the 450K and EPIC Bead Array data was conducted using Bioconductor package ChAMP in R (60). The analysis pipeline has been summarized in Supplemental Figure 1. Briefly, raw methylation data were imported into R and normalized using BMIQ. Then, 450K and EPIC data were merged on common probes and corrected for batch effects using combat function. Additional filtering was performed for common SNPs. DMPs were identified using a cutoff of a 10% change in methylation and q value less than 0.05. The first analysis focused on HF etiology and identified DMPs associated with ICM (ICM versus NF) and NICM (NICM versus NF) using data from pre-LVAD tissue obtained from 36 patients and 7 NF controls. The second analysis focused on the impact of mechanical unloading and identified DMPs that are associated with HF (pre-LVAD versus NF) and RR (post-LVAD versus pre-LVAD) in 8 paired cardiac tissue samples. The variance in global DNA methylation between subjects was assessed using PCA plots using the first 3 principal components. Hierarchical clustering of subjects was performed using the complete linkage method. DMPs common to ICM and NICM were screened for reciprocal changes in gene expression. RNA-Seq data from LV samples of 50 patients with HF (13 with ICM and 37 with NICM) and 14 NF controls was obtained from the Gene Expression Omnibus (GEO), accession number 116250, and analyzed using the limma package in R. A total of 36,755 transcripts with RPKM level of 0 in more than 50% of patients were filtered out. Data were log transformed using (RPKM + 1). Differential expression analysis was performed using linear model 1-way ANOVA. Differentially expressed genes (DEGs) were defined as genes with a *P* value adjusted for Benjamini-Hochberg FDR less than or equal to 0.05 between HF and control samples. Genes with an absolute log_2_FC difference greater than 0.25 were also included for DNA methylation versus gene expression correlation discovery analysis. The locations of human cardiac super enhancers were acquired from previous publications (61, 62). ChIP-Seq data are available through the GEO using the following accession numbers: adult human heart H3K27ac (GSE101345), adult human heart H3K4me1 (GSE101156), adult human heart H3K27me3 (GSE101387), and adult human heart CTCF (GSE 127553). DMPs located within intergenic regions were mapped to lncRNAs using GENCODE annotation database (63). LINC00881 protein interactions were retrieved from the RNAct database using the catRAPID algorithm (64).

### Histopathological examination of human heart samples.

Cardiomyocyte hypertrophy was determined by measuring the cross-sectional area (CSA) of cardiomyocytes in H&E-stained paraffin-embedded sections of human heart tissue. CSAs of more than 100 cardiomyocytes with a centrally located nuclear were measured and averaged in each sample. The extent of myocardial fibrosis was determined by the percent area of Masson’s trichrome staining using random images obtained in each heart tissue section. Large epicardial vessels as well as cutting or compression artifacts were excluded from the analysis.

### Cell culture and human cardiomyocyte differentiation.

iPS cells were obtained through material transfer agreements with G. Vunjak-Novakovic from B. Conklin, Gladstone Institute (San Francisco, California, USA; WTC-11, healthy). Cells were maintained on 1:20 diluted growth factor reduced Matrigel (Corning) in mTeSR-plus medium (StemCell Technologies) supplemented with 1% penicillin/streptomycin (Thermo Fisher Scientific) at 37°C, 5% O_2_. iPS cells were passaged at 30%–50% confluence using 0.5 mM EDTA (Thermo Fisher Scientific) and cultured for 24 hours in iPS media supplemented with 5 μM Y-27632 (Tocris Biosciences) prior to maintenance in iPS media. Cells were used between passages 40 and 70.

Cardiac differentiation of human iPS cells was performed using a stage-based protocol in RPMI 1640 medium (Thermo Fisher Scientific) supplemented with 0.5 mg/mL recombinant human albumin (Sigma-Aldrich), 213 μg/mL L-ascorbic acid 2-phosphate (Sigma-Aldrich), and 1% penicillin/streptomycin (CM Media). iPS cells were grown to 80%–90% confluence and changed into CM Media supplemented with 3 μM CHIR99021 (Tocris Biosciences) for 2 days. Media was then changed to CM Media supplemented with 2 μM Wnt-C59 (Tocris Biosciences) for 2 days prior to switching to CM Media without any supplements. CM Media is changed every 48 hours until contracting cells were noted by around day 10 following the initiation of differentiation, at which time the medium was changed to RPMI 1640 medium supplemented with B27 (50X; Gibco). Experiments were performed using hiPSC-CMs at day 15–35.

### Flow cytometry.

Cells were dissociated using 500 μL TrypLE for 20 minutes at 37°C and after a quick wash with PBS. Cells were collected by spinning down at 400*g* for 3 minutes at RT. Cell pellet was resuspended in 4% fixative solution for 15 minutes at 4°C and then treated in 0.1% Triton PBS buffer for 10 minutes at 4°C. Cold PBS wash steps were performed. Cells were incubated for 4 hours at 4°C in the dark with *TNNT2* Ab at 1:500 (Santa Cruz, catalog sc-20025). Cells were washed 3 times by cold PBS and centrifuged at 400*g* for 3 minutes at room temperature. Cell pellet was resuspended in 500 μL of Alexa Fluor–labeled secondary Ab at 1:1000 (Thermo Fisher Scientific, catalog A21203) and incubated for 2 hours at 4°C in the dark. At least 10,000 events were analyzed for each sample in FACSCanto and data were analyzed and presented using FlowJo software. Approximately 85% of differentiated hiPSC-CMs stained positive for cardiac troponin T at day 22 (Supplemental Figure 6).

### LINC00881 overexpression and knockdown in hiPSC-CMs.

Beating hiPSC-CMs were switched to Opti-MEM Reduced Serum Medium (Thermo Fisher Scientific). *LINC000881* knockdown was achieved by lipofectamine-based transfection of antisense LNA GapmeRs designed against *LINC00881* versus scrambled oligonucleotide (QIAGEN) using RNAiMAX reagent (Thermo Fisher Scientific) (Supplemental Table 13). hiPSC-CMs were harvested at 48–72 hours following transfection for functional and gene expression studies. Knockdown of *LINC00881* was confirmed by qPCR. For the *LINC00881* overexpression experiment, full-length human LINC00881 was cloned into p3XFLAG-CMV-7 (Millipore Sigma) vector and amplified in DH5α strain of *Escherichia coli* (*E coli*) (Life Technologies). After amplification, plasmids were extracted through QIAGEN Plasmid Midi Kit and stored at −80°C until use. Beating hiPSC-CMs were switched to Opti-MEM Reduced Serum Medium (Thermo Fisher Scientific). *LINC000881* overexpression was achieved by lipofectamine-based transfection of *LINC00881* plasmid versus bacterial alkaline phosphatase control plasmid using RNAiMAX reagent (Thermo Fisher Scientific). hiPSC-CMs were harvested at 48–72 hours following transfection for functional and gene expression studies. Overexpression of *LINC00881* was confirmed by qPCR.

### qPCR.

Total RNA was isolated from LV apical myocardial tissue and from beating hiPSC-CMs using Quick-RNA Miniprep Plus (Zymo Research). The qPCR was performed using SYBR mix (Thermo Fisher Scientific) on PicoReal96 Real-time PCR Systems (Thermo Fisher Scientific). Transcript quantification for mRNAs were performed using the Δ-Δ method using forward and reverse primers designed specifically for each of the target mRNAs and lncRNAs. The primer sequences used for independent validation are listed in Supplemental Table 13. For internal control, 18S was used.

### RNA-Seq and data analysis.

RNA concentration and integrity were assessed using a 2100 BioAnalyzer (Agilent). Sequencing libraries were constructed using the TruSeq Stranded Total RNA Library Prep Gold mRNA (Illumina) with an input of 1,000 ng and 11 cycles final amplification. Final libraries were quantified using High Sensitivity D1000 ScreenTape on a 2200 TapeStation (Agilent) and Qubit 1x dsDNA HS Assay Kit (Invitrogen). Samples were pooled equimolar with sequencing performed on an Illumina NovaSeq6000 SP 300 Cycle Flow Cell v1.5 as paired-end 151 reads. Sequences from fastq files were aligned to reference human genome (Gencode v38) using the splice-aware aligner STAR v.2.7.3a. Differential gene expression analysis with *LINC00881* knockdown or overexpression was performed using the R package DESeq2 (v1.34.0) from unnormalized count data.

### hiPSC-CM calcium imaging.

hiPSC-CM monolayers were loaded with 5 μM Calbryte-590 in RPMI + B27 medium at 37°C for 45 minutes. Loading media was washed and replaced with Tyrode’s salt solution. Calcium transience videos were acquired at 50 frames per second using a sCMOS camera (Zyla 4.2, Andor Technology) connected to an inverted fluorescence microscope (IX-81, Olympus) with cells placed in a live-cell chamber (STX Temp & CO_2_ Stage Top Incubator, Tokai Hit). Calcium signal analysis was performed using custom Python script as previously described (65).

### RNA IP.

The IP assay was performed in 2 million hiPSC-CMs. Cells were crosslinked with 1% formaldehyde in PBS for 10 minutes at 37°C and quenched with 1.25 M glycine. Cells were suspended in 1 mL of RIPA buffer (50 mM Tris, pH 7.4, 150 mM NaCl, 1 mM EDTA, 0.1% SDS, 1% NP-40, 0.5% sodium deoxycholate, 0.5mM DTT, 100 U/mL RNaseOUT, and 1X protease inhibitor cocktail), and incubated on ice for 30 minutes followed by centrifugation at 16,200*g* for 10 minutes. Lysates were precleared with washed Protein A/G magnetic beads (Thermo Fisher Scientific) at 4°C for 30 minutes. Then, 30 μL Protein A/G magnetic beads (Thermo Fisher Scientific) were incubated with 5 μg *SMARCA4* Ab (Proteintech, catalog 31634-1-AP) or IgG control (Proteintech, catalog 30000-0-AP) in 200 μL of RIPA buffer for 30 minutes at room temperature followed by incubation with precleared lysate for 4 hours at 4°C. Samples were washed 2 times in RIPA buffer, 4 times in 1 M high salt RIPA buffer (50 mM Tris, pH 7.4, 1 M NaCl, 1 mM EDTA, 0.1% SDS, 1% NP-40, and 0.5% sodium deoxycholate), and then twice in RIPA. RNA samples were extracted with Trizol.

### Chromatin accessibility assay.

Chromatin accessibility was performed using the EpiQuik Chromatin Accessibility Assay Kit (Epigentek). Briefly, hiPSC-CMs with or without *LINC00881* overexpression were lysed and chromatin was isolated. One chromatin aliquot was digested with nuclease, while the other was untreated. After incubation at 37°C for 4 minutes, reaching was quenched by adding stop solution. DNA was isolated followed by qPCR to amplify DNA for *MYH6, CACNA1C, and RYR2* gene promoters. Primer sequences used for chromatin accessibility are listed in Supplemental Table 13. Fold enrichment was calculated using formula: FE = 2 ^(Ct^ ^level^ ^with^ ^nuclease^^–^^Ct^ ^level^ ^without^ ^nuclease)^.

### Data sharing.

Genome-wide DNA methylation data from human HF (GSE197670) and RNA-Seq data from hiPSC-CMs (GSE197671) are available in NCBI’s GEO database.

### Statistics.

Statistical analyses were performed using R (R Core Team, 2021). Continuous data are presented as mean ± standard error of the mean. For comparisons between 2 groups, a 2-tailed unpaired *t* test was used. For comparisons between 3 or more groups, 1-way ANOVA with Fisher’s post hoc test or Kruskal-Wallis with Wilcoxon post hoc test was used. A *P* value of less than 0.05 was considered statistically significant in all analyses.

### Study approval.

The study was approved by Columbia University Irving Medical Center Institutional Review Board (IRB AAAR0055). Written informed consent was obtained for the procurement of discarded LV apical myocardial tissue at the time of LVAD implantation and cardiac transplantation.

## Author contributions

XL performed a majority of the experiments with help from PJK, BL, TRN, RZZ, AFGF, CX, and RL. VKT and XL designed the experiments. VKT, XL, and PJK wrote the manuscript with input from the coauthors. PCC, NU, SOM, MPR, and GVN provided conceptual advice. VKT coordinated and oversaw the whole project. XL was listed first because he performed a majority of the experiments.

## Supplementary Material

Supplemental data

## Figures and Tables

**Table 1:**
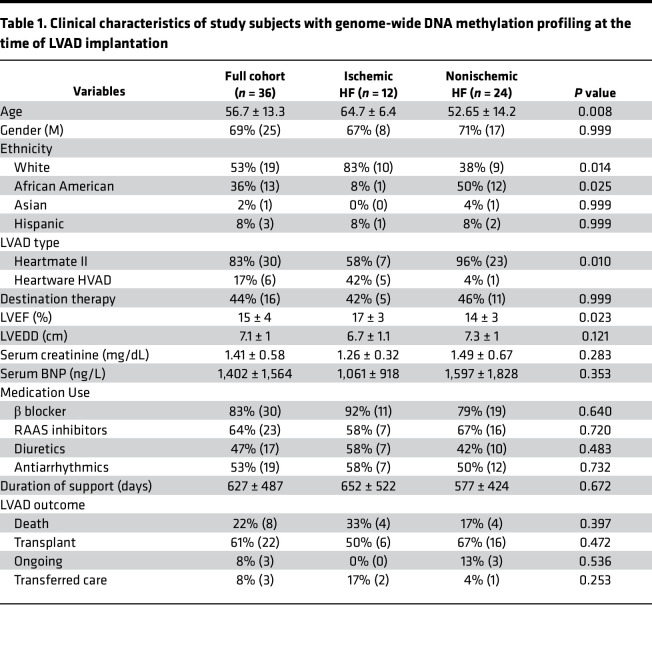
Clinical characteristics of study subjects with genome-wide DNA methylation profiling at the time of LVAD implantation

**Table 2:**
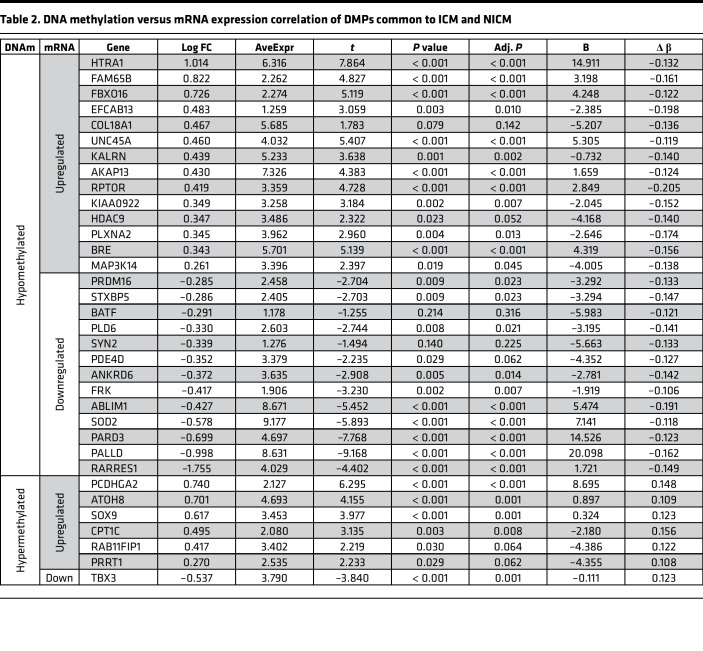
DNA methylation versus mRNA expression correlation of DMPs common to ICM and NICM

## Version 1 01/19/2023

In-Press Preview

## Version 2 02/22/2023

Electronic publication
