# Supplementary material for: Effect of mechanical unloading on genome-wide DNA methylation profile of the failing human heart
Source: JCI Insight. 2023 Feb 22;8(4):e161788. doi: 10.1172/jci.insight.161788 (PMC9977498; doi:10.1172/jci.insight.161788)
Supplement: Supplemental data [file jciinsight-8-161788-s142.pdf]

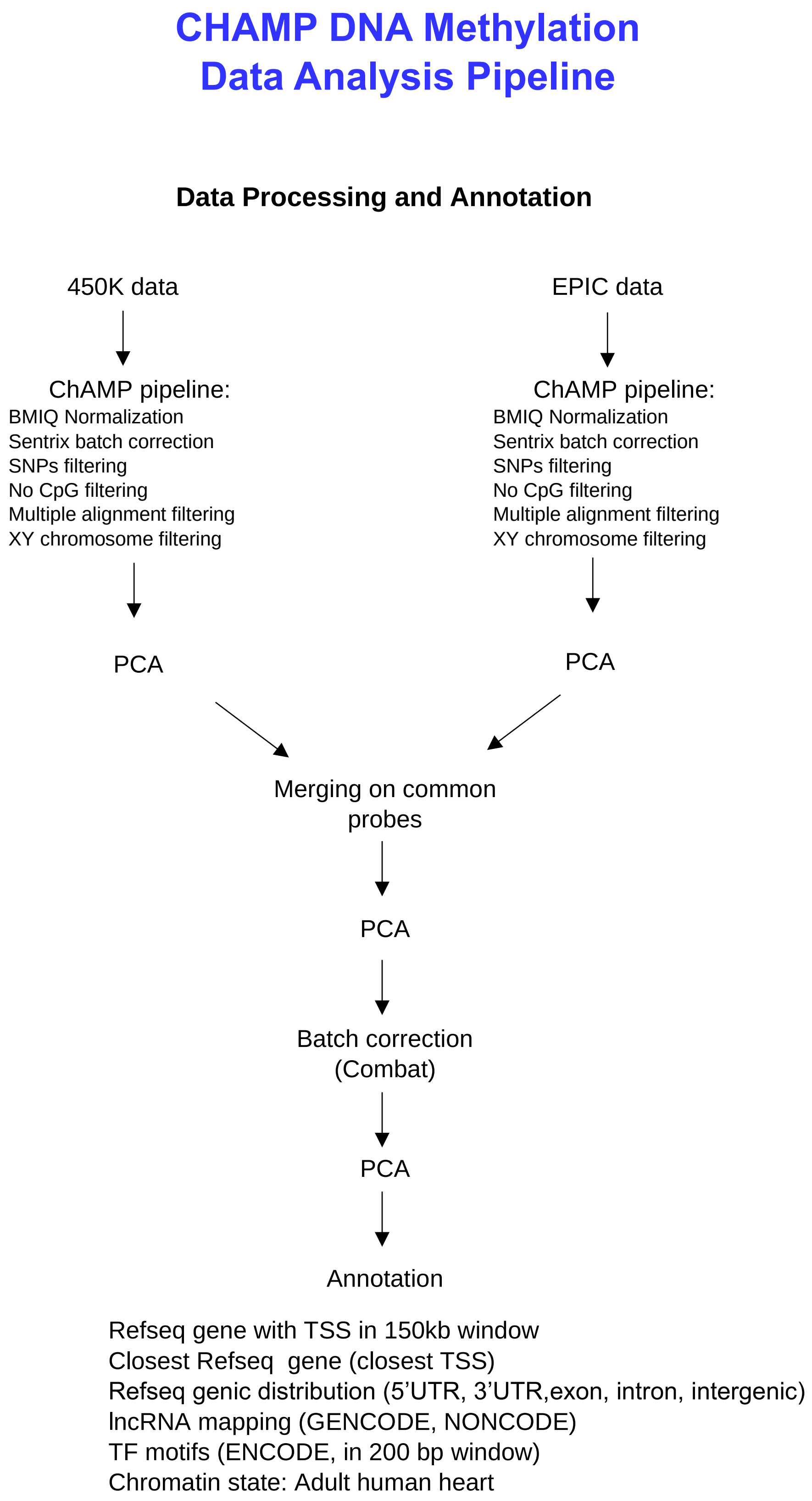

Supplemental Figure 1A. DNA Methylation Analysis pipeline using Bioconductor CHAMP Package from Illumina 450K and EPIC bead-array chip data.

Analysis 1: Etiology Specific HF DMPs

DMP Associated with Heart Failure

Annotated and filtered merged dataset

Case control:	Case control:
Ischemic HF vs Non-Failing	Non-Ischemic HF vs Non-Failing
DM: absolute Δ fract. Methyl>0.1	DM: absolute Δ fract. Methyl>0.1
q-value <0.05	q-value <0.05
ISCHEMIC DMPs	NON-ISCHEMIC DMPs

Analysis 2: LVAD Responsive HF DMPs

DMP Associated with Reverse Remodeling on LVAD

Annotated and filtered merged dataset

Case control:	Paired Analysis:
Pre-LVAD vs Non-Failing	Pre-LVAD vs Post-LVAD
DM: absolute Δ fract. Methyl>0.1	DM: absolute Δ fract. Methyl>0.1
q-value <0.05	q-value <0.05
HEART FAILURE DMPs	REVERSE REMODELING DMPs

Supplemental Figure 1B. Approach for human heart differential DNA Methylation analysis focusing on HF etiology specific (Analysis #1) and LVAD responsive (Analysis #2) differentially methylated positions.

A

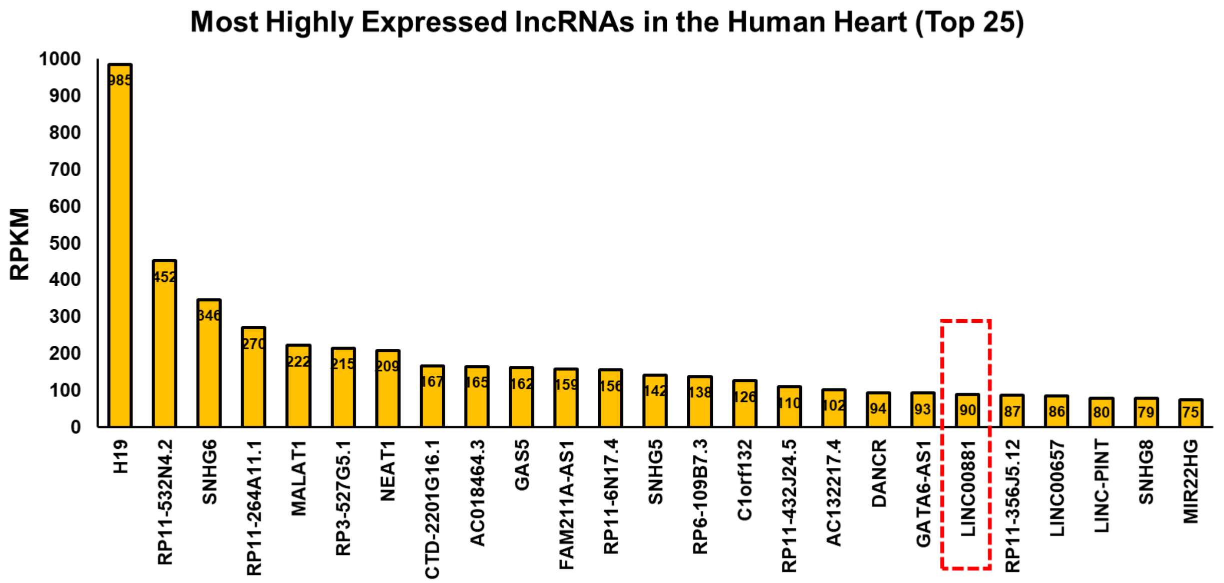

Most Highly Expressed lncRNAs in the Human Heart (Top 25)
RPKM
1000
900
800
700
600
500
400
300
200
100
0
985
452
346
270
222
215
209
167
165
162
159
156
142
138
126
110
102
94
93
90
87
86
80
79
75
H19
RP11-532N4.2
SNHG6
RP11-264A11.1
MALAT1
RP3-527G5.1
NEAT1
CTD-2201G16.1
AC018464.3
GAS5
FAM211A-AS1
RP11-6N17.4
SNHG5
RP6-109B7.3
C1orf132
RP11-432J24.5
AC132217.4
DANCR
GATA6-AS1
LINC00881
RP11-356J5.12
LINC00657
LINC-PINT
SNHG8
MIR22HG

B

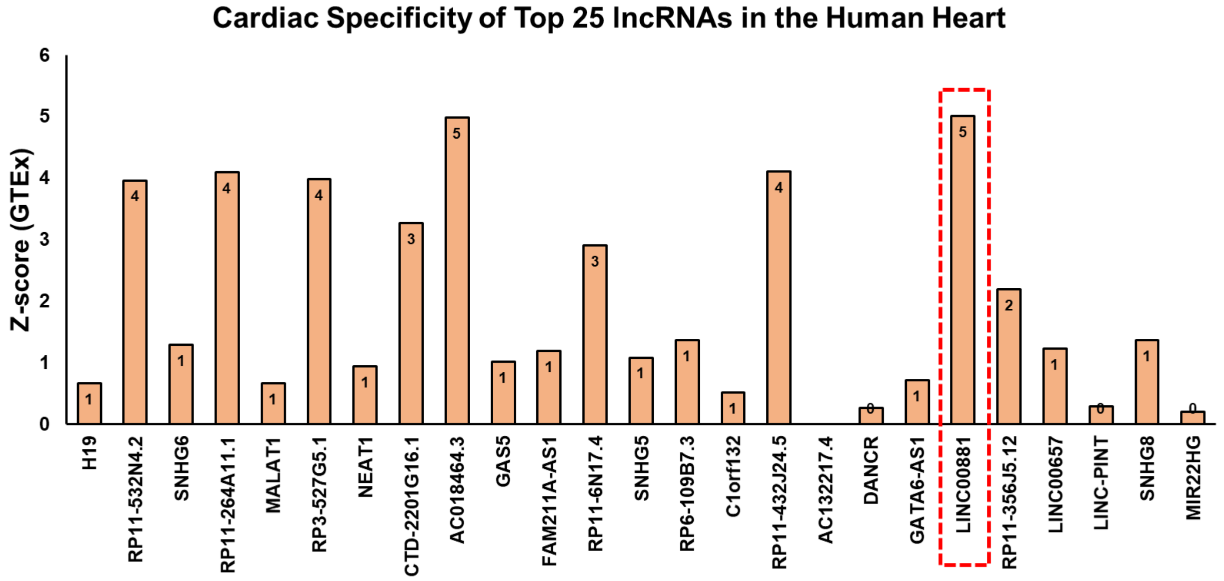

Cardiac Specificity of Top 25 lncRNAs in the Human Heart
Z-score (GTEx)
6
5
4
3
2
1
0
H19
RP11-532N4.2
SNHG6
RP11-264A11.1
MALAT1
RP3-527G5.1
NEAT1
CTD-2201G16.1
AC018464.3
GAS5
FAM211A-AS1
RP11-6N17.4
SNHG5
RP6-109B7.3
C1orf132
RP11-432J24.5
AC132217.4
DANCR
GATA6-AS1
LINC00881
RP11-356J5.12
LINC00657
LINC-PINT
SNHG8
MIR22HG

C

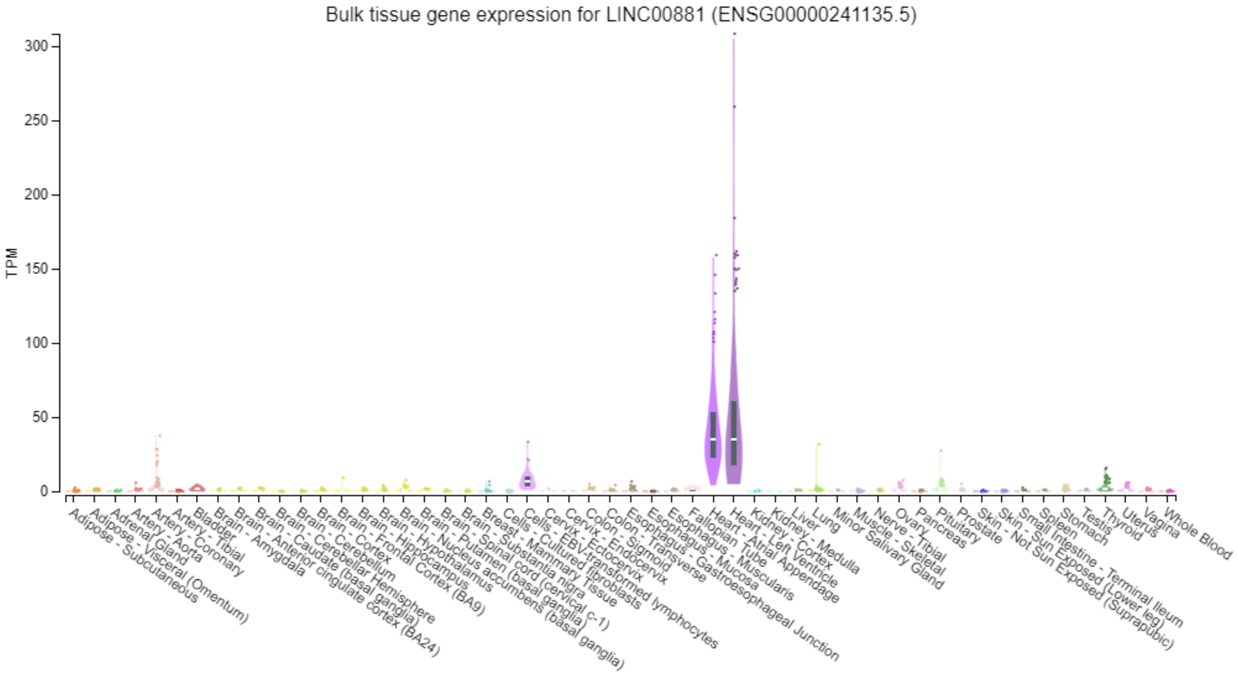

Supplemental Figure 2. Most highly expressed lncRNAs in the Human Heart. (A) Most highly expressed lnc-RNAs in the non-failing human heart in the descending order of RPKM using RNA-seq data obtained from GEO accession number 116250, **(B)** Cardiac-specificity of the most highly expressed cardiac lncRNAs determined by Z-score in the GTEx database, **(C)** Expression level of LINC00881 across diverse tissue types in the GTEx database (release v8).

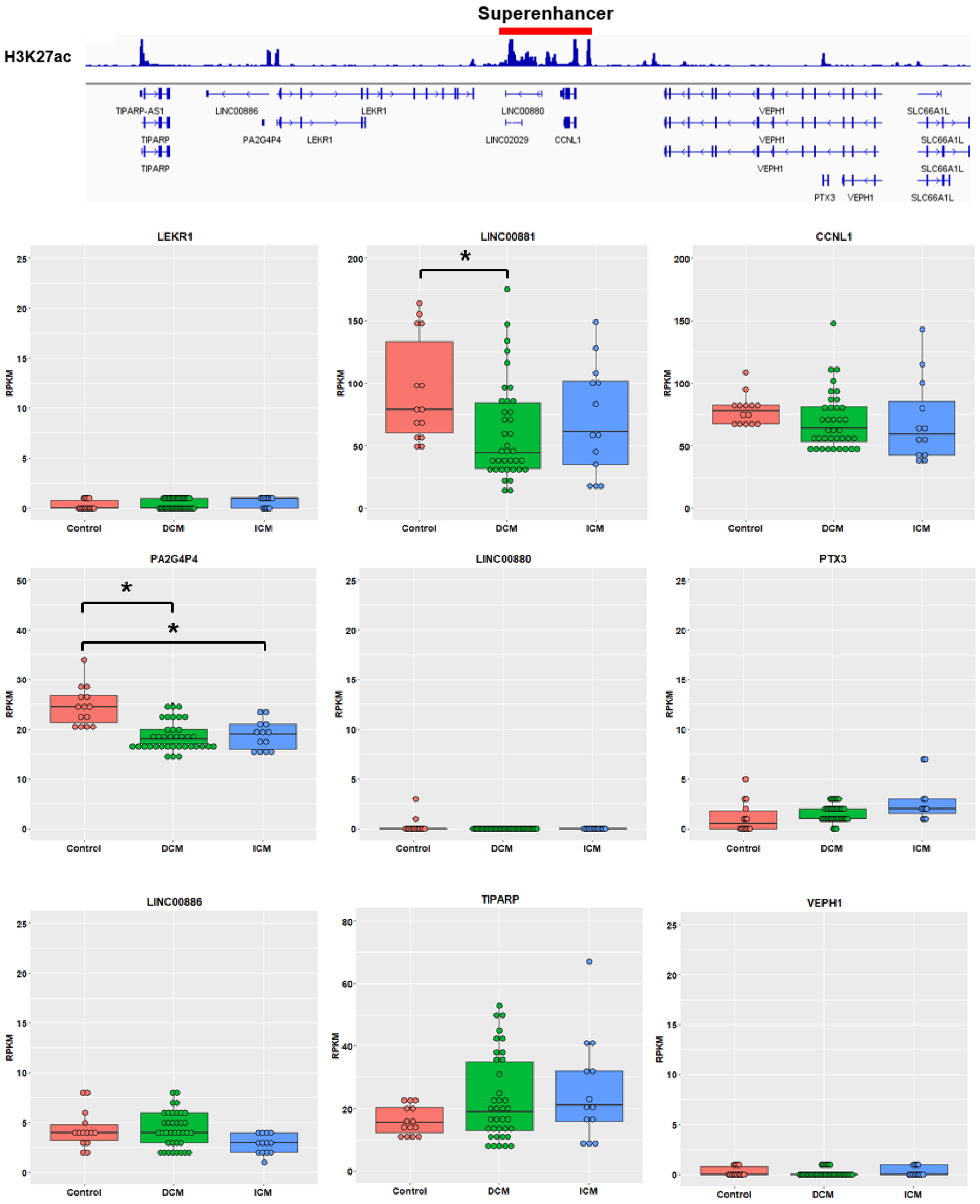

Supplemental Figure 3. LINC00881 cardiac super-enhancer region. Expression levels of LINC00881 and neighboring coding and non-coding transcripts in the non-failing (Control-red), non-ischemic (DCM-green), and ischemic (ICM-blue) human heart failure. RNA-seq data obtained from GEO accession number 116250. * FDR value <0.05

A

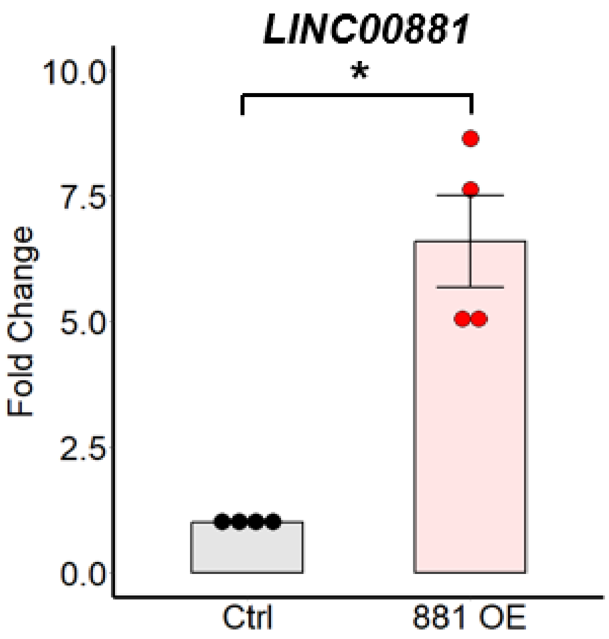

B

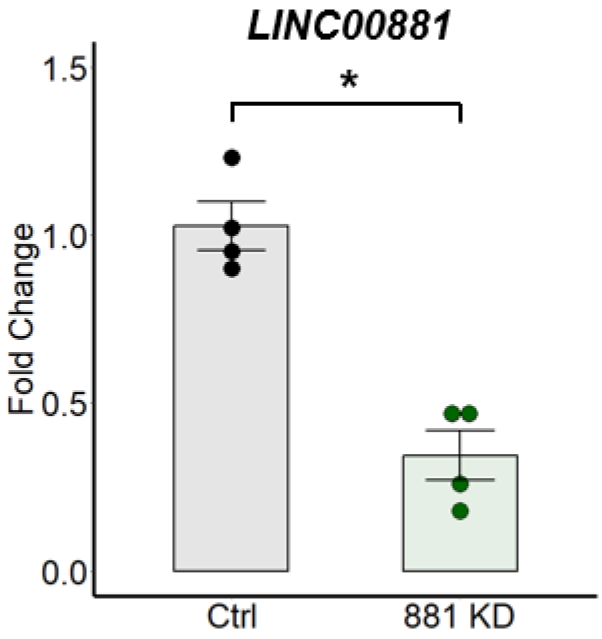

Supplemental Figure 4. LINC00881 overexpression and knockdown in the beating human iPS cell derived cardiomyocytes. (A) Plasmid-based overexpression of *LINC00881* validated by qPCR (B) GapmeR-mediated knockdown of *LINC00881* validated by qPCR * p-value <0.05 by unpaired t-test (n=4/group)

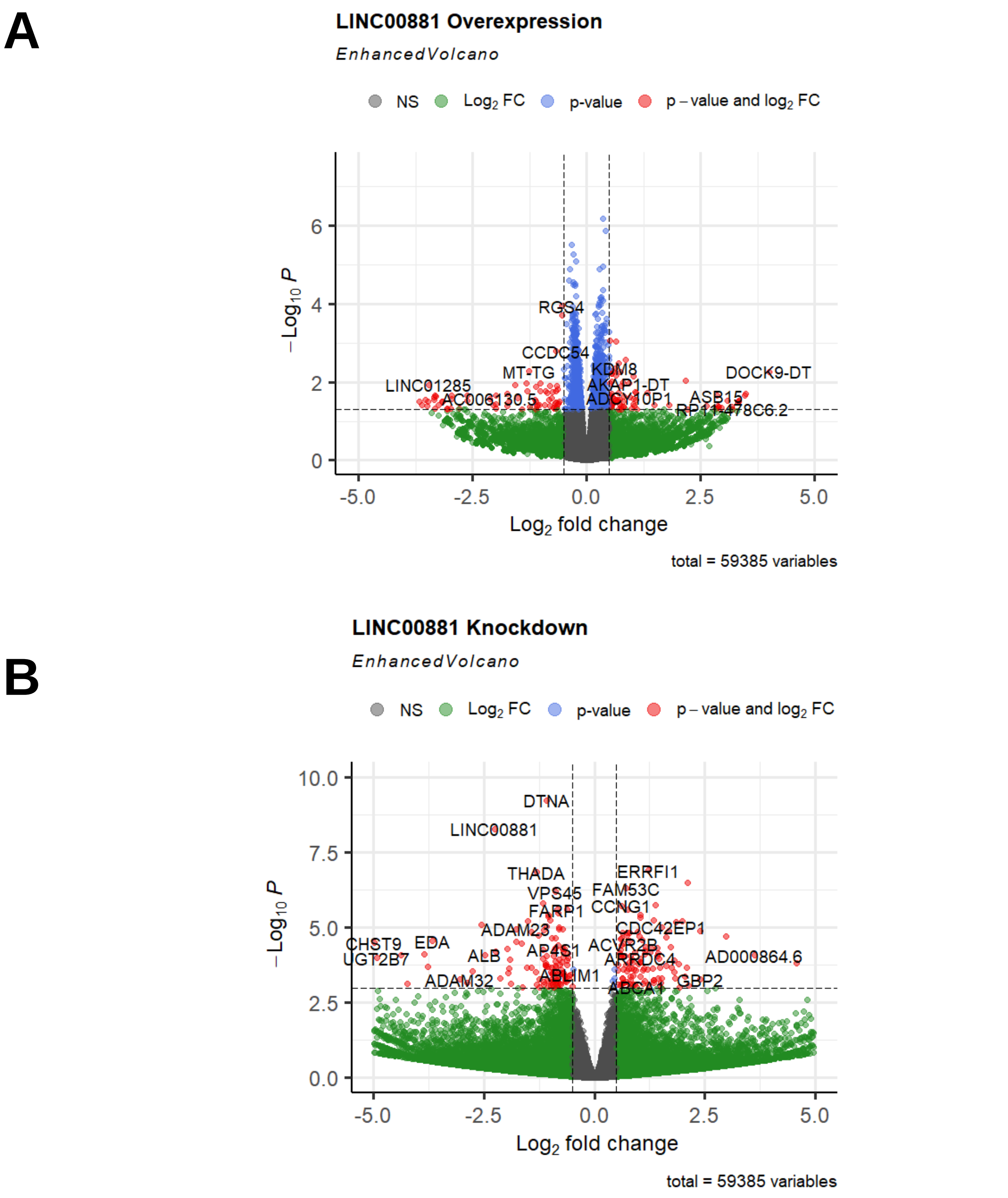

Supplemental Figure 5. RNA-seq in the beating human iPS cell derived cardiomyocytes.
(A) Volcano plot of differentially expressed transcripts with *LINC00881* plasmid overexpression
(B) Volcano plot of differentially expressed transcripts with *LINC00881* GapmeR knockdown

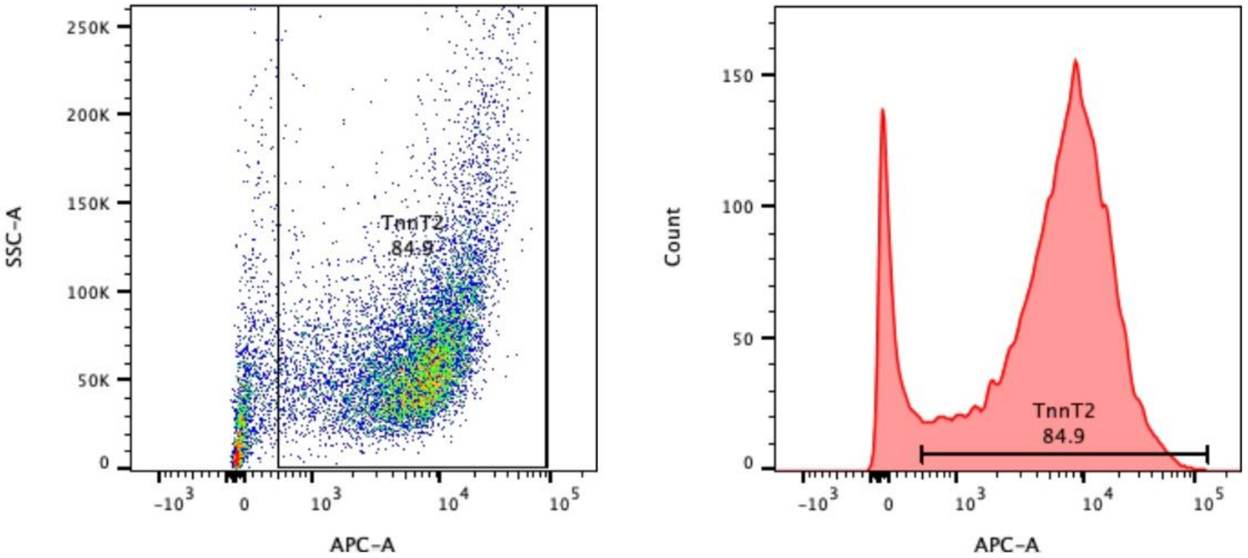

Supplemental Figure 6. Purity of beating human iPS cell derived cardiomyocytes (hiPSC-CMs). Flow cytometry analysis demonstrating percentage of human iPS cell derived cardiomyocytes staining positive (84.9% at day 20) for cardiac troponin T (*TNNT2*). Data is representative of at least 3 independent experiments.

Supplemental Table 1A. Echocardiographic and Laboratory Markers of 8 patients with paired pre- and post- LVAD cardiac tissue

Variables	Pre-LVAD (n= 8)	Post-LVAD (n=8)	p-value
**LVEF (%)**	16 ± 3	26 ± 14	0.072
**LVEDD (cm)**	7.3 ± 0.8	6.3 ± 0.96	**0.05**
**Serum Creatinine**	1.74 ± 1	1.86 ± 1.9	0.88
**Serum BNP**	1654 ± 1677	450 ± 619	0.08

Supplemental Table 1B. Characteristics of non-failing cardiac tissue donors used for genome-wide DNA methylation analysis

Donor #	Chip	Age	Sex	Ethnicity	Mechanism of Death	Height (inches)	Weight (pounds)
1	450K	Unknown	F	Unknown	Unknown	Unknown	Unknown
2	450K	Unknown	M	Unknown	Unknown	Unknown	Unknown
3	450K	Unknown	F	Unknown	Unknown	Unknown	Unknown
4	850K	60	M	Caucasian	Respiratory Arrest	Unknown	Unknown
5	850K	21	M	Caucasian	Head Trauma	74	218
6	850K	47	M	Caucasian	Respiratory Arrest	72	350
7	850K	29	F	Black	Intracranial bleed	63	153

Supplemental Table 2. 2079 differentially methylated positions in Ischemic HF vs. Non-Failing (adjusted p-value <0.05, abs (delta beta) > 10%)

CpG Site	log FC	Adj p-val	ICM	NF	Delta B	Gene	Feature	CGI
cg07167872	-0.34	0.0076	0.58	0.24	-0.34	PM20D1	TSS200	shore
cg04118610	-0.33	0.0034	0.76	0.43	-0.33	LPHN3	Body	opensea
cg05528899	-0.33	0.0432	0.67	0.35	-0.33		IGR	island
cg14159672	-0.32	0.0182	0.55	0.23	-0.32	PM20D1	1stExon	island
cg11965913	-0.30	0.0178	0.39	0.09	-0.30	PM20D1	TSS200	shore
cg04811114	-0.26	0.0001	0.47	0.21	-0.26	LGR6	TSS200	opensea
cg14893161	-0.26	0.0206	0.39	0.14	-0.26	PM20D1	5'UTR	shore
cg26536949	-0.25	0.0442	0.76	0.51	-0.25		IGR	island
cg17178900	-0.24	0.0262	0.63	0.39	-0.24	PM20D1	Body	island
cg24503407	-0.24	0.0182	0.64	0.40	-0.24	PM20D1	TSS1500	shore
cg12777520	-0.20	0.0002	0.55	0.35	-0.20	LMX1B	Body	island
cg13283845	-0.20	0.0266	0.77	0.57	-0.20		IGR	shore
cg04160030	-0.19	0.0002	0.63	0.43	-0.19	FUCA1	TSS1500	shore
cg24122364	-0.19	0.0038	0.59	0.40	-0.19	DOCK9	Body	opensea
cg14204784	-0.18	0.0002	0.53	0.35	-0.18	LMX1B	Body	island
cg12563372	-0.18	0.0001	0.50	0.32	-0.18		IGR	shore
cg02928365	-0.18	0.0000	0.44	0.26	-0.18	HLX	Body	shore
cg12930727	-0.18	0.0023	0.47	0.30	-0.18	HYAL1	TSS1500	opensea
cg24382823	-0.18	0.0005	0.44	0.26	-0.18	LMX1B	Body	island
cg17436134	-0.17	0.0034	0.47	0.30	-0.17		IGR	shore
cg12583076	-0.17	0.0060	0.56	0.39	-0.17	RASSF3	Body	opensea
cg10070864	-0.17	0.0009	0.72	0.55	-0.17		IGR	shelf
cg23066280	-0.17	0.0007	0.54	0.37	-0.17	PTPRN2	Body	opensea
cg14318858	-0.17	0.0000	0.77	0.60	-0.17	CPT1C	Body	island
cg19657945	-0.17	0.0452	0.92	0.75	-0.17		IGR	shore
cg18918831	-0.17	0.0418	0.66	0.50	-0.17	MUC4	Body	island
cg11679455	-0.16	0.0002	0.68	0.51	-0.16	GATA3	Body	island
cg10902396	-0.16	0.0008	0.52	0.36	-0.16	CHN2	Body	opensea
cg02455346	-0.16	0.0008	0.68	0.52	-0.16	HLX	Body	shore
cg23936410	-0.16	0.0072	0.43	0.27	-0.16		IGR	opensea
cg24838345	-0.16	0.0020	0.69	0.53	-0.16	MTSS1	Body	shelf
cg06410057	-0.16	0.0014	0.35	0.19	-0.16		IGR	shore
cg22077361	-0.16	0.0064	0.42	0.27	-0.16	FUCA1	TSS1500	shore
cg16200531	-0.16	0.0001	0.61	0.45	-0.16		IGR	opensea
cg01525538	-0.16	0.0003	0.45	0.30	-0.16	DUSP5P	Body	island
cg01107874	-0.16	0.0003	0.73	0.58	-0.16	C10orf41	Body	island
cg13752114	-0.16	0.0271	0.84	0.68	-0.16	MUC4	Body	island
cg03329019	-0.15	0.0001	0.62	0.47	-0.15		IGR	shore
cg10653297	-0.15	0.0094	0.68	0.53	-0.15	LOC285033	TSS200	opensea

cg25495534	-0.15	0.0006	0.55	0.39	-0.15		IGR	opensea
cg21283066	-0.15	0.0000	0.50	0.35	-0.15		IGR	opensea
cg23500537	-0.15	0.0001	0.49	0.33	-0.15		IGR	opensea
cg13375589	-0.15	0.0161	0.54	0.39	-0.15	SMTNL2	TSS200	shore
cg15131258	-0.15	0.0286	0.60	0.45	-0.15	C20orf71	5'UTR	opensea
cg02723558	-0.15	0.0039	0.55	0.40	-0.15	BDNFOS	Body	opensea
cg16732787	-0.15	0.0003	0.56	0.41	-0.15	DUSP5P	Body	island
cg06013117	-0.15	0.0000	0.78	0.63	-0.15	MSX2	Body	shore
cg20454002	-0.15	0.0008	0.49	0.34	-0.15	HLX	Body	shore
cg10604476	-0.15	0.0004	0.35	0.21	-0.15	ICAM5	Body	island
cg00765233	-0.15	0.0000	0.87	0.72	-0.15	SH3BP4	5'UTR	opensea
cg15273575	-0.15	0.0000	0.90	0.75	-0.15	UTRN	Body	opensea
cg09378783	-0.15	0.0001	0.63	0.48	-0.15	EGFR	Body	opensea
cg02861260	-0.15	0.0001	0.73	0.58	-0.15		IGR	opensea
cg04492228	-0.15	0.0000	0.57	0.42	-0.15	GATA3	Body	shore
cg06317507	-0.15	0.0014	0.42	0.27	-0.15	MGC34034	Body	island
cg01285926	-0.15	0.0016	0.38	0.23	-0.15		IGR	island
cg05044291	-0.15	0.0000	0.50	0.35	-0.15	LGR6	TSS200	opensea
cg20618651	-0.15	0.0240	0.78	0.64	-0.15	EXOC1	TSS1500	shore
cg23246095	-0.15	0.0077	0.67	0.52	-0.15	DPP4	TSS1500	shore
cg15948785	-0.15	0.0001	0.85	0.70	-0.15	PTPRN2	Body	opensea
cg13829104	-0.14	0.0001	0.76	0.62	-0.14	TBX3	Body	shore
cg22056094	-0.14	0.0009	0.67	0.52	-0.14	PCDHGA2	1stExon	shore
cg19306970	-0.14	0.0008	0.51	0.36	-0.14	HLX	Body	shore
cg05357209	-0.14	0.0007	0.68	0.54	-0.14	UNC84A	5'UTR	opensea
cg10880006	-0.14	0.0003	0.70	0.56	-0.14	PRDM8	Body	shore
cg03731740	-0.14	0.0161	0.53	0.39	-0.14	YTHDF2	TSS1500	shore
cg04244354	-0.14	0.0020	0.36	0.22	-0.14		IGR	shore
cg18242288	-0.14	0.0008	0.82	0.67	-0.14		IGR	opensea
cg26484001	-0.14	0.0259	0.48	0.34	-0.14		IGR	shelf
cg01535205	-0.14	0.0008	0.59	0.45	-0.14		IGR	opensea
cg07000514	-0.14	0.0008	0.77	0.63	-0.14	C7orf50	Body	island
cg11661493	-0.14	0.0001	0.74	0.60	-0.14	UBE2O	Body	opensea
cg01190989	-0.14	0.0045	0.45	0.30	-0.14	GPR125	Body	opensea
cg24060730	-0.14	0.0163	0.68	0.54	-0.14	CAV1	Body	opensea
cg10507304	-0.14	0.0020	0.40	0.26	-0.14		IGR	opensea
cg01900030	-0.14	0.0060	0.71	0.57	-0.14	CDK6	Body	opensea
cg11936410	-0.14	0.0184	0.60	0.46	-0.14	TPM1	Body	shelf
cg01912040	-0.14	0.0000	0.51	0.38	-0.14		IGR	shore
cg24953078	-0.14	0.0042	0.53	0.39	-0.14	NTM	Body	opensea
cg19699264	-0.14	0.0000	0.91	0.78	-0.14	SDPR	Body	opensea
cg23289079	-0.14	0.0007	0.52	0.39	-0.14	PRDM6	Body	shore
cg14774440	-0.14	0.0001	0.68	0.54	-0.14	RAB11FIP1	Body	opensea

cg13475333	-0.14	0.0220	0.40	0.26	-0.14		IGR	shore
cg07226964	-0.14	0.0149	0.50	0.36	-0.14	PPARGC1B	Body	opensea
cg02848875	-0.14	0.0100	0.61	0.47	-0.14	SNX1	TSS1500	shore
cg07050692	-0.14	0.0178	0.42	0.28	-0.14	CHD7	Body	opensea
cg01302853	-0.14	0.0074	0.73	0.59	-0.14	MAD1L1	Body	opensea
cg05214708	-0.14	0.0175	0.45	0.31	-0.14	KLF7	Body	opensea
cg07286216	-0.14	0.0186	0.47	0.34	-0.14	LOC650226	Body	island
cg08370082	-0.14	0.0306	0.59	0.45	-0.14	SENP5	Body	opensea
cg27625491	-0.14	0.0021	0.41	0.28	-0.14	CD36	TSS1500	opensea
cg26680989	-0.14	0.0000	0.78	0.64	-0.14		IGR	opensea
cg26654770	-0.14	0.0060	0.61	0.47	-0.14	NINJ2	Body	opensea
cg06118122	-0.14	0.0147	0.56	0.43	-0.14	LGALS8	Body	opensea
cg22770911	-0.13	0.0000	0.56	0.42	-0.13	GATA3	Body	shore
cg17489908	-0.13	0.0002	0.67	0.53	-0.13	GATA3	Body	shore
cg09753657	-0.13	0.0079	0.34	0.21	-0.13	CCDC19	TSS200	island
cg01275887	-0.13	0.0001	0.54	0.40	-0.13	FOXK1	Body	opensea
cg08070200	-0.13	0.0168	0.50	0.36	-0.13	FAT1	Body	opensea
cg00630991	-0.13	0.0205	0.57	0.44	-0.13	BCL2L14	3'UTR	opensea
cg10742917	-0.13	0.0095	0.26	0.12	-0.13		IGR	island
cg23080355	-0.13	0.0384	0.70	0.57	-0.13		IGR	opensea
cg22668906	-0.13	0.0058	0.64	0.51	-0.13		IGR	opensea
cg24436715	-0.13	0.0001	0.29	0.16	-0.13		IGR	island
cg04643690	-0.13	0.0000	0.85	0.72	-0.13	RORA	Body	opensea
cg10512202	-0.13	0.0127	0.62	0.49	-0.13	LIMD1	Body	opensea
cg02762475	-0.13	0.0488	0.37	0.24	-0.13	SLC12A7	Body	shelf
cg10207609	-0.13	0.0245	0.49	0.36	-0.13	CD36	TSS1500	opensea
cg19689427	-0.13	0.0000	0.45	0.32	-0.13	PCDHGA2	1stExon	shore
cg11197258	-0.13	0.0412	0.56	0.43	-0.13	NCOR2	Body	opensea
cg26884658	-0.13	0.0001	0.71	0.57	-0.13		IGR	shelf
cg01837362	-0.13	0.0009	0.32	0.19	-0.13		IGR	shore
cg11350586	-0.13	0.0019	0.54	0.41	-0.13	SOX9	TSS1500	shore
cg26926765	-0.13	0.0276	0.49	0.35	-0.13	C6orf142	Body	opensea
cg26254045	-0.13	0.0032	0.34	0.21	-0.13	SPRED3	Body	shore
cg24441899	-0.13	0.0215	0.34	0.21	-0.13	SDK1	Body	opensea
cg03348100	-0.13	0.0000	0.72	0.59	-0.13		IGR	shelf
cg16298867	-0.13	0.0161	0.58	0.45	-0.13		IGR	opensea
cg05290820	-0.13	0.0022	0.39	0.26	-0.13		IGR	shore
cg22041228	-0.13	0.0003	0.41	0.28	-0.13	HLX	Body	shore
cg18463607	-0.13	0.0133	0.84	0.71	-0.13	EXOC1	TSS1500	shore
cg18433146	-0.13	0.0139	0.70	0.57	-0.13	CD36	1stExon	opensea
cg04322298	-0.13	0.0303	0.61	0.48	-0.13	ORMDL1	3'UTR	opensea
cg14197071	-0.13	0.0001	0.66	0.53	-0.13	PRDM8	5'UTR	island
cg10248878	-0.13	0.0007	0.44	0.32	-0.13	GSX2	TSS1500	shore

cg05146756	-0.13	0.0095	0.48	0.35	-0.13	LOC554202	Body	shore
cg24760869	-0.13	0.0017	0.43	0.30	-0.13	ABLIM1	Body	opensea
cg12583591	-0.13	0.0444	0.83	0.70	-0.13	MUC16	TSS200	opensea
cg03061778	-0.13	0.0007	0.77	0.64	-0.13		IGR	opensea
cg26648818	-0.13	0.0000	0.44	0.31	-0.13	TOX3	TSS200	shore
cg25720795	-0.13	0.0018	0.55	0.42	-0.13		IGR	shore
cg16034541	-0.13	0.0275	0.74	0.62	-0.13	MUC4	Body	shore
cg20528787	-0.13	0.0004	0.47	0.35	-0.13	PTPRN2	Body	opensea
cg04471192	-0.13	0.0027	0.79	0.66	-0.13		IGR	opensea
cg04048392	-0.13	0.0050	0.39	0.26	-0.13	LMX1B	Body	shore
cg16512163	-0.13	0.0004	0.81	0.68	-0.13	RASA3	Body	opensea
cg24135491	-0.13	0.0109	0.70	0.58	-0.13	SMTNL2	TSS200	shore
cg03233940	-0.13	0.0016	0.77	0.64	-0.13		IGR	island
cg11430077	-0.13	0.0001	0.65	0.52	-0.13	GATA3	Body	shore
cg26489439	-0.13	0.0054	0.59	0.46	-0.13	SFMBT2	Body	opensea
cg25379549	-0.13	0.0010	0.81	0.68	-0.13	ADARB2	Body	opensea
cg00991744	-0.13	0.0239	0.62	0.49	-0.13	PDZRN4	TSS1500	shore
cg23458168	-0.13	0.0086	0.51	0.39	-0.13	ZNF536	5'UTR	shore
cg25887789	-0.13	0.0010	0.73	0.60	-0.13	PDZRN4	TSS1500	shore
cg15415259	-0.13	0.0001	0.55	0.43	-0.13	MGC34034	Body	shore
cg26769984	-0.13	0.0003	0.85	0.73	-0.13	C7orf50	Body	island
cg24750887	-0.13	0.0035	0.51	0.39	-0.13	HERC3	Body	opensea
cg08005692	-0.13	0.0039	0.32	0.20	-0.13	ASH1L	5'UTR	opensea
cg26500142	-0.13	0.0006	0.56	0.44	-0.13		IGR	shore
cg04290171	-0.13	0.0204	0.62	0.49	-0.13	CD46	TSS1500	shore
cg04392266	-0.13	0.0202	0.75	0.62	-0.13		IGR	island
cg17669581	-0.13	0.0021	0.38	0.25	-0.13		IGR	shore
cg04154653	-0.13	0.0314	0.93	0.80	-0.13	TTLL10	3'UTR	shore
cg19561274	-0.12	0.0104	0.72	0.59	-0.12	LOC285033	TSS1500	opensea
cg01310397	-0.12	0.0164	0.83	0.71	-0.12	MUC4	Body	shore
cg13466694	-0.12	0.0064	0.48	0.35	-0.12	LMX1B	Body	shelf
cg04438997	-0.12	0.0018	0.50	0.38	-0.12	SOX9	TSS1500	shore
cg15359163	-0.12	0.0012	0.35	0.23	-0.12	PRDM6	Body	shore
cg10798815	-0.12	0.0041	0.49	0.37	-0.12	VPS13D	3'UTR	opensea
cg08210507	-0.12	0.0004	0.89	0.77	-0.12	MAD1L1	Body	shore
cg15061330	-0.12	0.0283	0.54	0.41	-0.12		IGR	opensea
cg23596123	-0.12	0.0037	0.39	0.27	-0.12	PCDHB6	1stExon	shore
cg18713687	-0.12	0.0349	0.81	0.69	-0.12	MUC4	Body	island
cg03329576	-0.12	0.0000	0.51	0.38	-0.12		IGR	opensea
cg25129097	-0.12	0.0003	0.78	0.66	-0.12		IGR	opensea
cg12302647	-0.12	0.0005	0.53	0.41	-0.12	ABLIM3	Body	opensea
cg12354192	-0.12	0.0079	0.53	0.40	-0.12	TNIK	Body	opensea
cg25906537	-0.12	0.0000	0.85	0.73	-0.12	TYRO3	Body	opensea

cg12864721	-0.12	0.0008	0.54	0.42	-0.12	C10orf41	Body	island
cg13213165	-0.12	0.0000	0.84	0.72	-0.12	SGIP1	Body	opensea
cg13755866	-0.12	0.0218	0.77	0.65	-0.12		IGR	opensea
cg04105726	-0.12	0.0088	0.74	0.62	-0.12	LOC728661	Body	island
cg01301252	-0.12	0.0001	0.73	0.61	-0.12	PCDHGA2	Body	shore
cg26481249	-0.12	0.0241	0.46	0.34	-0.12	SORBS2	5'UTR	opensea
cg20438915	-0.12	0.0486	0.55	0.43	-0.12	PKP4	5'UTR	shelf
cg12334013	-0.12	0.0012	0.47	0.35	-0.12	EEPD1	Body	shelf
cg17705066	-0.12	0.0169	0.69	0.57	-0.12	GIPC3	Body	island
cg07028950	-0.12	0.0267	0.67	0.55	-0.12		IGR	opensea
cg12910268	-0.12	0.0203	0.64	0.52	-0.12	DNAJB6	Body	opensea
cg04388989	-0.12	0.0228	0.37	0.24	-0.12		IGR	island
cg10401362	-0.12	0.0013	0.75	0.63	-0.12	DNAJB6	Body	opensea
cg14920808	-0.12	0.0338	0.90	0.78	-0.12		IGR	opensea
cg01581084	-0.12	0.0183	0.39	0.26	-0.12	OSR2	Body	shore
cg02551980	-0.12	0.0339	0.43	0.31	-0.12	MCC	Body	shore
cg02128244	-0.12	0.0004	0.42	0.30	-0.12		IGR	island
cg09936799	-0.12	0.0012	0.45	0.33	-0.12	PCDHA6	Body	shore
cg12308391	-0.12	0.0000	0.72	0.60	-0.12	NCAM1	Body	opensea
cg23490829	-0.12	0.0008	0.57	0.44	-0.12	PCDHA1	Body	shelf
cg22432760	-0.12	0.0013	0.73	0.60	-0.12	TBCD	Body	island
cg09881503	-0.12	0.0000	0.77	0.65	-0.12	CDC45L	3'UTR	shelf
cg27425228	-0.12	0.0000	0.85	0.73	-0.12	TBC1D22A	Body	shelf
cg10735454	-0.12	0.0000	0.84	0.72	-0.12	PARK2	Body	opensea
cg25979526	-0.12	0.0093	0.70	0.58	-0.12	MYO5A	Body	opensea
cg05452645	-0.12	0.0007	0.61	0.49	-0.12	PRDM8	TSS1500	shore
cg16583884	-0.12	0.0000	0.67	0.55	-0.12	CCDC68	TSS200	shore
cg26360026	-0.12	0.0005	0.70	0.58	-0.12		IGR	opensea
cg27065003	-0.12	0.0006	0.44	0.32	-0.12	PRDM6	Body	shore
cg15074047	-0.12	0.0067	0.40	0.28	-0.12	SDPR	Body	opensea
cg03080147	-0.12	0.0001	0.84	0.72	-0.12	MTMR7	Body	shore
cg06360465	-0.12	0.0086	0.49	0.37	-0.12	HYAL1	TSS200	opensea
cg19763428	-0.12	0.0042	0.38	0.26	-0.12	PDE1C	Body	shore
cg08125574	-0.12	0.0164	0.58	0.46	-0.12		IGR	opensea
cg16376902	-0.12	0.0016	0.26	0.14	-0.12		IGR	shelf
cg12934382	-0.12	0.0035	0.26	0.14	-0.12	GRM2	1stExon	island
cg18304448	-0.12	0.0008	0.60	0.48	-0.12	MGC45800	Body	shore
cg11024506	-0.12	0.0233	0.59	0.47	-0.12	RHOBTB1	Body	opensea
cg14497054	-0.12	0.0090	0.27	0.15	-0.12		IGR	island
cg00877329	-0.12	0.0000	0.47	0.35	-0.12	HPSE2	TSS1500	shelf
cg23588121	-0.12	0.0000	0.54	0.42	-0.12	SIM1	Body	shore
cg22946147	-0.12	0.0499	0.59	0.48	-0.12	ZNF804B	Body	opensea
cg06648759	-0.12	0.0042	0.42	0.30	-0.12		IGR	opensea

cg12587213	-0.12	0.0374	0.48	0.36	-0.12	SRGAP2	Body	opensea
cg05826245	-0.12	0.0302	0.43	0.32	-0.12	STYK1	TSS1500	shore
cg14635269	-0.12	0.0036	0.59	0.48	-0.12	LMX1B	Body	opensea
cg05342469	-0.12	0.0374	0.65	0.53	-0.12	ODZ3	Body	opensea
cg24807169	-0.12	0.0031	0.42	0.30	-0.12	EMCN	TSS1500	opensea
cg09775238	-0.12	0.0057	0.75	0.64	-0.12	MTHFSD	Body	opensea
cg18645081	-0.12	0.0003	0.45	0.33	-0.12	HLX	TSS1500	shore
cg05345283	-0.12	0.0000	0.73	0.62	-0.12	AP2A2	Body	shelf
cg26883434	-0.12	0.0402	0.62	0.50	-0.12	C5orf13	5'UTR	shore
cg14714922	-0.12	0.0000	0.68	0.57	-0.12		IGR	opensea
cg09484852	-0.12	0.0309	0.51	0.39	-0.12	ZDHHC14	Body	opensea
cg02989448	-0.12	0.0000	0.76	0.64	-0.12	SLC45A1	TSS200	shore
cg11190278	-0.12	0.0070	0.42	0.30	-0.12	NIN	Body	opensea
cg20161089	-0.12	0.0008	0.64	0.53	-0.12	IFI27	5'UTR	opensea
cg04566694	-0.12	0.0234	0.45	0.33	-0.12	H6PD	Body	opensea
cg11951818	-0.12	0.0043	0.61	0.50	-0.12	MSX2	Body	shore
cg26067760	-0.12	0.0000	0.44	0.33	-0.12	HPSE2	TSS1500	shelf
cg20367712	-0.12	0.0009	0.35	0.23	-0.12	GSX2	Body	island
cg09430976	-0.12	0.0028	0.36	0.24	-0.12		IGR	shore
cg17946038	-0.12	0.0002	0.88	0.77	-0.12		IGR	shore
cg10163955	-0.12	0.0001	0.85	0.73	-0.12	GATA3	Body	shore
cg25490334	-0.12	0.0037	0.73	0.61	-0.12	ESR1	Body	opensea
cg22822630	-0.12	0.0156	0.57	0.45	-0.12	USP12	Body	opensea
cg21386863	-0.12	0.0007	0.72	0.60	-0.12		IGR	opensea
cg10388307	-0.12	0.0032	0.73	0.61	-0.12		IGR	opensea
cg25090604	-0.12	0.0001	0.79	0.68	-0.12	ATP11A	Body	shelf
cg02513556	-0.12	0.0206	0.63	0.52	-0.12		IGR	opensea
cg09232478	-0.12	0.0010	0.37	0.26	-0.12		IGR	shore
cg23077820	-0.12	0.0011	0.40	0.29	-0.12	PAX3	Body	shore
cg22902505	-0.12	0.0002	0.49	0.37	-0.12	PRDM8	5'UTR	shore
cg06307913	-0.12	0.0013	0.31	0.19	-0.12	PRDM8	5'UTR	shore
cg11842367	-0.12	0.0003	0.63	0.51	-0.12	MGC27382	Body	opensea
cg16725974	-0.12	0.0326	0.41	0.29	-0.12	SYNE2	5'UTR	opensea
cg14959425	-0.12	0.0187	0.59	0.48	-0.12	ITGB8	Body	opensea
cg25142479	-0.12	0.0007	0.77	0.66	-0.12	COL9A1	Body	shore
cg03441770	-0.12	0.0003	0.75	0.64	-0.12		IGR	shore
cg00061629	-0.12	0.0049	0.67	0.55	-0.12	ALX4	Body	shore
cg26186954	-0.12	0.0292	0.45	0.34	-0.12	PSKH2	TSS200	island
cg05483388	-0.12	0.0002	0.83	0.72	-0.12	TM4SF4	1stExon	opensea
cg23387597	-0.12	0.0264	0.59	0.47	-0.12	ITPRIP	TSS200	shelf
cg09673570	-0.12	0.0005	0.83	0.72	-0.12	MICALCL	3'UTR	opensea
cg03786043	-0.12	0.0031	0.73	0.61	-0.12		IGR	opensea
cg23518439	-0.12	0.0000	0.52	0.40	-0.12	FGF22	TSS1500	island

cg07575193	-0.12	0.0051	0.58	0.46	-0.12	RBPMS	Body	opensea
cg12161848	-0.12	0.0004	0.77	0.65	-0.12	RAPGEF5	Body	opensea
cg16530086	-0.12	0.0168	0.44	0.33	-0.12	CUGBP2	Body	shelf
cg15129144	-0.12	0.0015	0.71	0.59	-0.12	EPAS1	Body	shore
cg09439204	-0.12	0.0111	0.56	0.45	-0.12		IGR	shore
cg25451765	-0.12	0.0414	0.56	0.45	-0.12	SDK1	Body	opensea
cg02081006	-0.12	0.0045	0.41	0.30	-0.12	PRDM6	Body	shore
cg03829739	-0.12	0.0171	0.44	0.33	-0.12	LOC650226	Body	shore
cg18849847	-0.12	0.0033	0.86	0.75	-0.12	PTPRN2	Body	opensea
cg24059119	-0.12	0.0046	0.35	0.24	-0.12		IGR	island
cg17083209	-0.12	0.0145	0.35	0.23	-0.12	LPHN2	5'UTR	opensea
cg10503133	-0.11	0.0000	0.74	0.62	-0.11	SNN	5'UTR	shelf
cg14214797	-0.11	0.0045	0.59	0.47	-0.11	CCDC88C	Body	opensea
cg02794892	-0.11	0.0048	0.43	0.31	-0.11	CD209	TSS1500	opensea
cg24677093	-0.11	0.0002	0.77	0.66	-0.11	C10orf41	Body	island
cg11125249	-0.11	0.0121	0.59	0.48	-0.11	GYG1	Body	opensea
cg11565786	-0.11	0.0013	0.75	0.64	-0.11	PDZD2	Body	opensea
cg13638420	-0.11	0.0057	0.41	0.30	-0.11	WT1	Body	island
cg22689316	-0.11	0.0002	0.80	0.69	-0.11		IGR	opensea
cg10580282	-0.11	0.0042	0.57	0.46	-0.11	HYAL1	5'UTR	shelf
cg15934776	-0.11	0.0066	0.63	0.51	-0.11	AFF3	Body	opensea
cg26958524	-0.11	0.0004	0.28	0.17	-0.11	FOXL1	1stExon	island
cg05604800	-0.11	0.0000	0.88	0.76	-0.11	SPIRE2	Body	shelf
cg05914150	-0.11	0.0267	0.50	0.38	-0.11	PIK3R1	TSS200	opensea
cg26926138	-0.11	0.0056	0.41	0.30	-0.11		IGR	shore
cg12211856	-0.11	0.0000	0.29	0.17	-0.11	SDCCAG8	Body	island
cg17117243	-0.11	0.0216	0.61	0.49	-0.11	SESN1	Body	opensea
cg10334976	-0.11	0.0006	0.77	0.65	-0.11	ECHS1	Body	opensea
cg24127414	-0.11	0.0230	0.63	0.51	-0.11	PCDHB11	1stExon	shore
cg23610041	-0.11	0.0017	0.29	0.17	-0.11		IGR	shore
cg22480109	-0.11	0.0418	0.36	0.25	-0.11	ESYT2	Body	opensea
cg01835580	-0.11	0.0039	0.69	0.58	-0.11	RNASE7	Body	opensea
cg24439448	-0.11	0.0000	0.75	0.64	-0.11		IGR	shore
cg27067781	-0.11	0.0006	0.50	0.38	-0.11	PRRT1	3'UTR	island
cg05578480	-0.11	0.0003	0.71	0.60	-0.11	FAM89A	Body	shore
cg02327530	-0.11	0.0006	0.60	0.48	-0.11	C7orf51	3'UTR	shore
cg05748163	-0.11	0.0033	0.29	0.18	-0.11	EBF2	Body	shore
cg12881854	-0.11	0.0052	0.42	0.31	-0.11		IGR	shelf
cg16406892	-0.11	0.0001	0.73	0.62	-0.11	TBX3	Body	shore
cg27332878	-0.11	0.0002	0.62	0.51	-0.11	ARNT2	Body	opensea
cg16566353	-0.11	0.0096	0.77	0.66	-0.11	ITGA9	3'UTR	opensea
cg05661093	-0.11	0.0068	0.73	0.62	-0.11		IGR	opensea
cg00947478	-0.11	0.0045	0.76	0.64	-0.11		IGR	opensea

cg07524214	-0.11	0.0139	0.77	0.66	-0.11		IGR	opensea
cg03113878	-0.11	0.0010	0.31	0.19	-0.11	C7orf51	Body	island
cg00931339	-0.11	0.0000	0.83	0.72	-0.11	NAT14	TSS1500	island
cg03089725	-0.11	0.0003	0.79	0.67	-0.11	FGFR1	Body	opensea
cg12819431	-0.11	0.0022	0.81	0.69	-0.11		IGR	shelf
cg18500988	-0.11	0.0027	0.54	0.43	-0.11		IGR	shore
cg19214097	-0.11	0.0039	0.62	0.51	-0.11		IGR	opensea
cg10547527	-0.11	0.0186	0.97	0.86	-0.11	BOLL	Body	island
cg08554603	-0.11	0.0028	0.63	0.51	-0.11	C7orf58	Body	opensea
cg14141912	-0.11	0.0005	0.55	0.44	-0.11	ATOH8	Body	opensea
cg05522011	-0.11	0.0006	0.65	0.54	-0.11	PRDM8	Body	shore
cg13250209	-0.11	0.0013	0.76	0.65	-0.11	CCDC85C	Body	opensea
cg15575320	-0.11	0.0000	0.79	0.68	-0.11	SLC45A1	1stExon	island
cg03068843	-0.11	0.0000	0.70	0.59	-0.11	DLC1	Body	shelf
cg21533331	-0.11	0.0008	0.23	0.12	-0.11		IGR	island
cg11117717	-0.11	0.0011	0.66	0.55	-0.11	AP4S1	Body	opensea
cg18702935	-0.11	0.0000	0.86	0.75	-0.11		IGR	opensea
cg11948367	-0.11	0.0023	0.63	0.52	-0.11		IGR	opensea
cg03776662	-0.11	0.0003	0.29	0.18	-0.11	PRDM6	Body	island
cg12190994	-0.11	0.0002	0.71	0.59	-0.11	SDK1	Body	island
cg11391335	-0.11	0.0019	0.41	0.30	-0.11	TBX15	5'UTR	shore
cg23291280	-0.11	0.0004	0.32	0.21	-0.11		IGR	island
cg20487860	-0.11	0.0006	0.80	0.69	-0.11		IGR	opensea
cg10508347	-0.11	0.0012	0.83	0.72	-0.11	SLC45A1	1stExon	island
cg14413262	-0.11	0.0010	0.45	0.34	-0.11	SLC45A1	TSS1500	shore
cg26706759	-0.11	0.0032	0.74	0.63	-0.11	CCDC99	5'UTR	shore
cg22695986	-0.11	0.0220	0.24	0.13	-0.11		IGR	island
cg04982834	-0.11	0.0001	0.43	0.32	-0.11	EVI5	TSS1500	opensea
cg22867629	-0.11	0.0000	0.67	0.56	-0.11	BDKRB2	5'UTR	opensea
cg20016599	-0.11	0.0206	0.61	0.49	-0.11		IGR	opensea
cg11460282	-0.11	0.0288	0.51	0.40	-0.11		IGR	island
cg00748640	-0.11	0.0001	0.90	0.78	-0.11	GPRC5B	5'UTR	opensea
cg01300495	-0.11	0.0294	0.69	0.58	-0.11	LHFP	Body	opensea
cg12479878	-0.11	0.0002	0.48	0.37	-0.11	HLX	Body	island
cg26572392	-0.11	0.0152	0.40	0.29	-0.11	KIAA1217	5'UTR	opensea
cg20494563	-0.11	0.0139	0.38	0.27	-0.11		IGR	opensea
cg18537383	-0.11	0.0001	0.87	0.76	-0.11	MCPH1	Body	opensea
cg11094568	-0.11	0.0016	0.61	0.50	-0.11	TSSC1	Body	shore
cg04044203	-0.11	0.0000	0.61	0.50	-0.11	ESAM	Body	shore
cg04427498	-0.11	0.0000	0.39	0.28	-0.11		IGR	island
cg05006473	-0.11	0.0046	0.70	0.59	-0.11		IGR	shore
cg23874437	-0.11	0.0288	0.25	0.14	-0.11	SPRED3	Body	island
cg13997553	-0.11	0.0035	0.59	0.48	-0.11	TNC	Body	opensea

cg11855643	-0.11	0.0415	0.44	0.33	-0.11	C1orf21	Body	opensea
cg14085715	-0.11	0.0015	0.72	0.61	-0.11	MACC1	TSS200	opensea
cg03113038	-0.11	0.0003	0.65	0.54	-0.11		IGR	opensea
cg26312807	-0.11	0.0256	0.36	0.25	-0.11		IGR	island
cg06834313	-0.11	0.0037	0.70	0.59	-0.11		IGR	opensea
cg26919014	-0.11	0.0248	0.69	0.58	-0.11	MMP15	Body	shore
cg02427468	-0.11	0.0375	0.37	0.26	-0.11	ESYT2	Body	opensea
cg12392104	-0.11	0.0048	0.38	0.27	-0.11	CCPG1	Body	opensea
cg06647600	-0.11	0.0001	0.70	0.59	-0.11		IGR	shore
cg14377416	-0.11	0.0067	0.70	0.59	-0.11	VAV2	Body	opensea
cg04674315	-0.11	0.0005	0.48	0.37	-0.11		IGR	shore
cg26624021	-0.11	0.0003	0.63	0.52	-0.11	CETP	TSS200	opensea
cg14355374	-0.11	0.0087	0.54	0.43	-0.11		IGR	opensea
cg13799838	-0.11	0.0002	0.69	0.58	-0.11	TLE1	Body	opensea
cg00426709	-0.11	0.0003	0.84	0.73	-0.11		IGR	shore
cg14612733	-0.11	0.0097	0.61	0.50	-0.11	EFNA1	Body	shelf
cg25781595	-0.11	0.0014	0.48	0.37	-0.11	GRIN2D	Body	shore
cg16852483	-0.11	0.0002	0.76	0.65	-0.11	ITGBL1	TSS1500	shelf
cg17303119	-0.11	0.0002	0.82	0.71	-0.11	NAV2	Body	opensea
cg09164580	-0.11	0.0001	0.33	0.22	-0.11	GDF6	Body	island
cg04231085	-0.11	0.0001	0.83	0.72	-0.11		IGR	opensea
cg10362869	-0.11	0.0050	0.47	0.36	-0.11	NCKAP5	TSS1500	opensea
cg27203924	-0.11	0.0000	0.85	0.74	-0.11	ADAMTS9	Body	opensea
cg16602500	-0.11	0.0053	0.44	0.33	-0.11	ZCCHC11	3'UTR	opensea
cg17873750	-0.11	0.0001	0.54	0.43	-0.11	NHLH2	5'UTR	shore
cg12701302	-0.11	0.0003	0.83	0.72	-0.11	MAN1C1	Body	opensea
cg15724945	-0.11	0.0074	0.47	0.36	-0.11		IGR	shore
cg08470264	-0.11	0.0045	0.65	0.54	-0.11	MTHFSD	Body	opensea
cg01418539	-0.11	0.0000	0.87	0.76	-0.11		IGR	shore
cg09982883	-0.11	0.0007	0.75	0.64	-0.11		IGR	shelf
cg04497015	-0.11	0.0001	0.92	0.81	-0.11	C16orf74	5'UTR	shelf
cg02497785	-0.11	0.0036	0.62	0.51	-0.11	ABCA13	Body	island
cg08234418	-0.11	0.0002	0.79	0.68	-0.11	SFMBT2	Body	opensea
cg26076905	-0.11	0.0083	0.53	0.42	-0.11	PIK3R1	TSS200	opensea
cg26227569	-0.11	0.0000	0.72	0.61	-0.11		IGR	shelf
cg02907415	-0.11	0.0000	0.91	0.80	-0.11		IGR	opensea
cg16867657	-0.11	0.0004	0.60	0.49	-0.11	ELOVL2	TSS1500	island
cg11736230	-0.11	0.0257	0.34	0.23	-0.11	PPP1R13B	Body	shore
cg00512459	-0.11	0.0010	0.58	0.47	-0.11		IGR	opensea
cg06952801	-0.11	0.0005	0.77	0.67	-0.11		IGR	opensea
cg07145988	-0.11	0.0269	0.57	0.46	-0.11	RERE	Body	opensea
cg26299084	-0.11	0.0000	0.31	0.20	-0.11	PRDM8	5'UTR	island
cg15618758	-0.11	0.0134	0.65	0.55	-0.11	ARID1B	Body	opensea

cg26407316	-0.11	0.0005	0.69	0.58	-0.11	CDKL1	Body	opensea
cg18044113	-0.11	0.0027	0.69	0.59	-0.11	MAD1L1	Body	opensea
cg01255894	-0.11	0.0002	0.38	0.27	-0.11	GATA3	Body	shore
cg12288941	-0.11	0.0007	0.36	0.25	-0.11	LOC145845	Body	shelf
cg14065590	-0.11	0.0009	0.70	0.60	-0.11	PCDHB11	TSS200	shore
cg02479744	-0.11	0.0079	0.60	0.49	-0.11	ATXN1	3'UTR	opensea
cg15516226	-0.11	0.0000	0.84	0.73	-0.11	BTNL9	5'UTR	opensea
cg04165859	-0.11	0.0000	0.86	0.75	-0.11		IGR	opensea
cg07341007	-0.11	0.0067	0.62	0.52	-0.11	MUC4	Body	island
cg03867429	-0.11	0.0000	0.33	0.22	-0.11		IGR	shore
cg14071179	-0.11	0.0017	0.36	0.25	-0.11	PRDM6	Body	island
cg09122414	-0.11	0.0129	0.41	0.30	-0.11	INPP5B	Body	opensea
cg12451671	-0.11	0.0027	0.58	0.47	-0.11	ATXN1	3'UTR	opensea
cg01993468	-0.11	0.0002	0.79	0.68	-0.11	LOC154449	TSS200	opensea
cg17971113	-0.11	0.0017	0.79	0.68	-0.11	CORIN	Body	shelf
cg17328052	-0.11	0.0044	0.78	0.67	-0.11	EEPD1	Body	shore
cg03316237	-0.11	0.0012	0.38	0.27	-0.11	EXOC3L2	TSS1500	shore
cg11465639	-0.11	0.0000	0.52	0.41	-0.11		IGR	opensea
cg14871588	-0.11	0.0003	0.47	0.36	-0.11		IGR	shore
cg22910295	-0.11	0.0010	0.34	0.23	-0.11	ICAM5	Body	island
cg03122674	-0.11	0.0223	0.54	0.43	-0.11	LOC650226	Body	island
cg07746514	-0.11	0.0340	0.71	0.61	-0.11	MLEC	3'UTR	opensea
cg07741162	-0.11	0.0002	0.47	0.36	-0.11	PRDM6	Body	shore
cg01655667	-0.11	0.0168	0.37	0.26	-0.11		IGR	opensea
cg24356544	-0.11	0.0077	0.74	0.64	-0.11	OGDHL	TSS1500	shore
cg05427639	-0.11	0.0061	0.51	0.40	-0.11	NT5C3	1stExon	opensea
cg13944507	-0.11	0.0259	0.68	0.57	-0.11		IGR	opensea
cg27099262	-0.11	0.0082	0.35	0.24	-0.11		IGR	opensea
cg09491120	-0.11	0.0022	0.69	0.58	-0.11	FOXF2	Body	shore
cg27353899	-0.11	0.0425	0.42	0.31	-0.11	MUC4	Body	island
cg19651757	-0.11	0.0446	0.66	0.55	-0.11		IGR	opensea
cg24919768	-0.11	0.0109	0.37	0.27	-0.11	MALL	Body	island
cg10913077	-0.11	0.0024	0.60	0.49	-0.11	ARHGEF3	5'UTR	opensea
cg01799521	-0.11	0.0480	0.56	0.45	-0.11	RRAGC	3'UTR	opensea
cg25140419	-0.11	0.0006	0.41	0.31	-0.11		IGR	island
cg13018402	-0.11	0.0044	0.63	0.52	-0.11	SH3TC2	TSS1500	opensea
cg15998761	-0.11	0.0003	0.78	0.67	-0.11	MFSD6	Body	opensea
cg05754861	-0.11	0.0083	0.49	0.38	-0.11	OSR2	Body	shore
cg13470063	-0.11	0.0000	0.68	0.58	-0.11	TIE1	TSS1500	opensea
cg07046818	-0.11	0.0179	0.39	0.28	-0.11	GDF6	Body	shelf
cg24066843	-0.11	0.0008	0.76	0.65	-0.11	PCDHGA2	Body	shore
cg00596222	-0.11	0.0001	0.71	0.60	-0.11		IGR	opensea
cg11002923	-0.11	0.0003	0.60	0.50	-0.11	CCDC36	5'UTR	island

cg26196860	-0.11	0.0033	0.77	0.66	-0.11		IGR	opensea
cg00551647	-0.11	0.0000	0.72	0.62	-0.11	CYYR1	Body	shore
cg23971170	-0.11	0.0101	0.32	0.22	-0.11	LMX1A	TSS1500	island
cg22647929	-0.11	0.0002	0.75	0.64	-0.11	SOX7	Body	shore
cg23038520	-0.11	0.0034	0.84	0.74	-0.11	RPLP0P2	Body	opensea
cg03750736	-0.11	0.0010	0.73	0.63	-0.11		IGR	shelf
cg15474435	-0.11	0.0006	0.80	0.69	-0.11	COL6A6	Body	opensea
cg03066050	-0.11	0.0101	0.39	0.28	-0.11	KLF4	Body	island
cg08439122	-0.11	0.0000	0.90	0.79	-0.11	CAPN2	TSS1500	shore
cg20717585	-0.11	0.0020	0.48	0.37	-0.11	PCDHGA2	1stExon	island
cg02793451	-0.11	0.0018	0.58	0.48	-0.11	TOX3	TSS1500	shore
cg11234013	-0.11	0.0002	0.80	0.69	-0.11	BDNFOS	Body	opensea
cg04955333	-0.11	0.0084	0.72	0.61	-0.11	IQCE	Body	shore
cg00923880	-0.11	0.0004	0.58	0.47	-0.11		IGR	shore
cg25436886	-0.11	0.0369	0.28	0.18	-0.11		IGR	island
cg13761419	-0.11	0.0194	0.44	0.33	-0.11		IGR	shelf
cg00982548	-0.11	0.0020	0.92	0.82	-0.11	BOLL	5'UTR	island
cg17522332	-0.11	0.0030	0.74	0.63	-0.11	ADARB2	Body	opensea
cg22897615	-0.11	0.0035	0.32	0.21	-0.11	PRRT1	Body	island
cg11045331	-0.11	0.0135	0.59	0.49	-0.11	AGAP1	Body	opensea
cg18004701	-0.11	0.0182	0.26	0.15	-0.11	PTPRN2	Body	island
cg20570797	-0.11	0.0147	0.37	0.26	-0.11	PXDN	Body	opensea
cg12799888	-0.11	0.0001	0.85	0.74	-0.11		IGR	opensea
cg27322863	-0.11	0.0155	0.65	0.55	-0.11	TMCO7	Body	opensea
cg23999170	-0.11	0.0098	0.72	0.61	-0.11	TSPAN2	Body	shelf
cg14204462	-0.11	0.0001	0.29	0.18	-0.11		IGR	shelf
cg00009750	-0.11	0.0001	0.67	0.56	-0.11	CDK15	Body	opensea
cg08980987	-0.11	0.0006	0.56	0.46	-0.11	BCAT1	Body	shore
cg05477823	-0.11	0.0007	0.62	0.51	-0.11	TBX2	Body	island
cg10090844	-0.11	0.0093	0.31	0.20	-0.11		IGR	shelf
cg25386234	-0.11	0.0157	0.30	0.20	-0.11		IGR	shore
cg07891658	-0.11	0.0000	0.79	0.69	-0.11	BANP	5'UTR	opensea
cg02143988	-0.11	0.0076	0.73	0.62	-0.11		IGR	opensea
cg15689835	-0.11	0.0018	0.40	0.29	-0.11	LMX1B	Body	island
cg24125710	-0.11	0.0087	0.46	0.35	-0.11		IGR	opensea
cg06595211	-0.11	0.0376	0.67	0.57	-0.11		IGR	opensea
cg22265452	-0.11	0.0004	0.76	0.65	-0.11		IGR	island
cg07179669	-0.11	0.0006	0.24	0.14	-0.11	HLX	Body	island
cg18055467	-0.11	0.0147	0.51	0.41	-0.11	LMX1B	Body	shore
cg02380802	-0.11	0.0457	0.60	0.50	-0.11		IGR	shore
cg14827391	-0.11	0.0001	0.72	0.61	-0.11	NXN	Body	opensea
cg14008999	-0.11	0.0036	0.60	0.50	-0.11	NXN	Body	opensea
cg03915432	-0.11	0.0007	0.81	0.70	-0.11		IGR	opensea

cg22190023	-0.11	0.0356	0.32	0.22	-0.11	PCDHB7	1stExon	shore
cg21575706	-0.11	0.0031	0.74	0.63	-0.11	RSPO4	3'UTR	opensea
cg19095143	-0.11	0.0107	0.42	0.32	-0.11	TEX264	Body	opensea
cg09829551	-0.10	0.0279	0.38	0.27	-0.10		IGR	shore
cg16907496	-0.10	0.0009	0.59	0.49	-0.10	CNTN4	Body	opensea
cg10091265	-0.10	0.0006	0.66	0.55	-0.10	SGPP2	TSS1500	shore
cg15749858	-0.10	0.0063	0.60	0.49	-0.10	MACC1	1stExon	opensea
cg17786697	-0.10	0.0000	0.47	0.37	-0.10	PRDM6	Body	shore
cg15001843	-0.10	0.0116	0.64	0.53	-0.10		IGR	opensea
cg00742851	-0.10	0.0224	0.85	0.75	-0.10	LRRN1	5'UTR	shore
cg06881898	-0.10	0.0065	0.47	0.36	-0.10	F3	Body	shore
cg12600534	-0.10	0.0001	0.83	0.73	-0.10		IGR	opensea
cg22507154	-0.10	0.0042	0.31	0.21	-0.10		IGR	island
cg16931969	-0.10	0.0082	0.53	0.43	-0.10	ANK2	Body	opensea
cg00366435	-0.10	0.0018	0.81	0.71	-0.10		IGR	opensea
cg06266189	-0.10	0.0047	0.30	0.20	-0.10	NGEF	Body	shore
cg16580765	-0.10	0.0001	0.81	0.71	-0.10	PRKD2	Body	shore
cg11946963	-0.10	0.0004	0.80	0.69	-0.10	NCAM1	Body	opensea
cg10350689	-0.10	0.0037	0.79	0.69	-0.10	CXCL1	Body	shore
cg00219282	-0.10	0.0046	0.40	0.29	-0.10	FNDC1	Body	island
cg09559189	-0.10	0.0173	0.32	0.22	-0.10	EBF2	Body	shore
cg00957698	-0.10	0.0024	0.82	0.72	-0.10	C4orf31	5'UTR	shore
cg20178924	-0.10	0.0346	0.70	0.59	-0.10	ANKRD1	Body	opensea
cg24947064	-0.10	0.0007	0.73	0.62	-0.10	ITGA1	Body	shelf
cg15129097	-0.10	0.0005	0.86	0.76	-0.10	SFMBT2	Body	opensea
cg22031964	-0.10	0.0010	0.82	0.71	-0.10	CHRM1	Body	opensea
cg08785724	-0.10	0.0001	0.40	0.30	-0.10		IGR	opensea
cg13981043	-0.10	0.0062	0.84	0.73	-0.10		IGR	opensea
cg01881549	-0.10	0.0150	0.57	0.47	-0.10	SH3BP4	5'UTR	opensea
cg13687594	-0.10	0.0072	0.51	0.41	-0.10	CAMTA1	Body	shore
cg22122862	-0.10	0.0284	0.42	0.32	-0.10	PRDM16	Body	island
cg23517999	-0.10	0.0006	0.57	0.47	-0.10		IGR	opensea
cg22753668	-0.10	0.0102	0.64	0.54	-0.10	FLT1	Body	opensea
cg11276172	-0.10	0.0228	0.60	0.50	-0.10	ITPRIP	TSS200	shelf
cg04162299	-0.10	0.0272	0.49	0.39	-0.10	DOCK4	Body	opensea
cg01861509	-0.10	0.0004	0.88	0.78	-0.10	SPOCK2	TSS1500	shore
cg08279008	-0.10	0.0065	0.31	0.21	-0.10	POLR1A	Body	opensea
cg23881119	-0.10	0.0274	0.34	0.23	-0.10	KIAA1217	5'UTR	opensea
cg16412772	-0.10	0.0009	0.72	0.62	-0.10		IGR	island
cg11741335	-0.10	0.0001	0.66	0.55	-0.10	EEPD1	Body	opensea
cg17462107	-0.10	0.0081	0.63	0.53	-0.10	MAST4	Body	opensea
cg04436994	-0.10	0.0077	0.63	0.52	-0.10	HLX	Body	island
cg01862363	-0.10	0.0021	0.72	0.61	-0.10	NTN1	Body	shore

cg05426299	-0.10	0.0015	0.73	0.63	-0.10	C6orf195	TSS1500	shore
cg05493042	-0.10	0.0000	0.78	0.68	-0.10		IGR	opensea
cg06373870	-0.10	0.0007	0.50	0.40	-0.10	PRDM8	TSS1500	shore
cg18533201	-0.10	0.0002	0.32	0.21	-0.10	GDF6	Body	island
cg27273054	-0.10	0.0000	0.67	0.57	-0.10	EHD1	3'UTR	island
cg19788727	-0.10	0.0019	0.73	0.63	-0.10	NTN1	Body	shore
cg16049600	-0.10	0.0147	0.33	0.23	-0.10	PCDHB11	TSS200	shore
cg06564875	-0.10	0.0002	0.71	0.60	-0.10		IGR	opensea
cg03905718	-0.10	0.0016	0.31	0.21	-0.10	SRL	Body	opensea
cg20694241	-0.10	0.0040	0.65	0.55	-0.10	CNTNAP3	Body	shore
cg02140384	-0.10	0.0105	0.67	0.56	-0.10		IGR	shore
cg23288563	-0.10	0.0077	0.34	0.24	-0.10	NDRG2	Body	shore
cg15626350	-0.10	0.0289	0.35	0.25	-0.10	ESR1	Body	shore
cg25019989	-0.10	0.0018	0.23	0.13	-0.10		IGR	shelf
cg12364786	-0.10	0.0199	0.52	0.41	-0.10	HERC1	Body	opensea
cg10953003	-0.10	0.0046	0.28	0.18	-0.10	FUCA1	TSS1500	shore
cg01404163	-0.10	0.0013	0.46	0.35	-0.10	TOX3	TSS200	shore
cg01185754	-0.10	0.0020	0.50	0.39	-0.10	F3	Body	shore
cg03755748	-0.10	0.0004	0.77	0.67	-0.10	BANP	5'UTR	opensea
cg27541691	-0.10	0.0001	0.33	0.22	-0.10	TUBG2	TSS1500	shore
cg01201512	-0.10	0.0203	0.64	0.54	-0.10	NINJ2	Body	opensea
cg01024458	-0.10	0.0000	0.87	0.77	-0.10	RERE	Body	opensea
cg24795614	-0.10	0.0008	0.69	0.59	-0.10	C2orf34	Body	opensea
cg22088248	-0.10	0.0031	0.72	0.61	-0.10	ZADH2	Body	shore
cg12620806	-0.10	0.0009	0.84	0.73	-0.10	NTF3	Body	shore
cg21282663	-0.10	0.0222	0.50	0.40	-0.10	HLX	Body	shore
cg24724428	-0.10	0.0001	0.22	0.12	-0.10	ELOVL2	TSS1500	island
cg09844094	-0.10	0.0109	0.57	0.47	-0.10		IGR	opensea
cg26946311	-0.10	0.0001	0.75	0.65	-0.10	DECR2	Body	shore
cg15450224	-0.10	0.0000	0.78	0.68	-0.10		IGR	opensea
cg01456640	-0.10	0.0034	0.87	0.77	-0.10		IGR	island
cg05138702	-0.10	0.0301	0.60	0.50	-0.10	CTBP2	5'UTR	opensea
cg07195926	-0.10	0.0418	0.40	0.30	-0.10	GATA2	Body	shelf
cg02720618	-0.10	0.0018	0.32	0.22	-0.10	ESR1	Body	shore
cg00241664	-0.10	0.0001	0.85	0.75	-0.10	IFT140	Body	opensea
cg05721751	-0.10	0.0010	0.82	0.72	-0.10	ADARB2	Body	opensea
cg26819695	-0.10	0.0027	0.40	0.30	-0.10	ASAM	TSS1500	island
cg16201418	-0.10	0.0081	0.73	0.63	-0.10		IGR	opensea
cg13691108	-0.10	0.0001	0.83	0.72	-0.10	PPP1R16B	TSS1500	shore
cg10105681	-0.10	0.0243	0.20	0.10	-0.10		IGR	opensea
cg00075920	-0.10	0.0024	0.46	0.36	-0.10	B4GALT7	TSS1500	shore
cg23479065	-0.10	0.0059	0.74	0.64	-0.10	LRRN1	5'UTR	shore
cg13267140	-0.10	0.0001	0.71	0.61	-0.10		IGR	opensea

cg22311403	-0.10	0.0002	0.85	0.75	-0.10	MLLT4	Body	island
cg22025206	-0.10	0.0044	0.49	0.39	-0.10	SLC9A3	Body	shore
cg06536469	-0.10	0.0007	0.74	0.64	-0.10		IGR	shelf
cg15580052	-0.10	0.0300	0.32	0.21	-0.10	B4GALNT3	Body	opensea
cg21794419	-0.10	0.0000	0.80	0.70	-0.10	MCF2L2	Body	shore
cg06826341	-0.10	0.0012	0.34	0.24	-0.10	LHX4	Body	shelf
cg07270153	-0.10	0.0011	0.32	0.22	-0.10	FERD3L	TSS1500	shore
cg21170796	-0.10	0.0023	0.45	0.35	-0.10	TBX15	5'UTR	shore
cg20326248	-0.10	0.0053	0.76	0.65	-0.10	TNFRSF19	Body	opensea
cg09270879	-0.10	0.0000	0.87	0.77	-0.10		IGR	opensea
cg03463411	-0.10	0.0023	0.36	0.26	-0.10	PRDM8	TSS1500	island
cg04213746	-0.10	0.0023	0.79	0.69	-0.10	GATA3	Body	opensea
cg16353800	-0.10	0.0064	0.83	0.72	-0.10	ZADH2	Body	shore
cg10832655	-0.10	0.0014	0.76	0.66	-0.10	C4orf31	5'UTR	shore
cg13398470	-0.10	0.0010	0.57	0.47	-0.10		IGR	opensea
cg03670393	-0.10	0.0001	0.49	0.39	-0.10		IGR	island
cg06496885	-0.10	0.0004	0.82	0.71	-0.10		IGR	opensea
cg26836204	-0.10	0.0000	0.72	0.61	-0.10		IGR	opensea
cg16990168	-0.10	0.0131	0.38	0.27	-0.10	TBX15	5'UTR	shore
cg10593816	-0.10	0.0000	0.81	0.71	-0.10	NDUFA12	TSS1500	shore
cg01159576	-0.10	0.0018	0.41	0.31	-0.10	LMX1B	Body	island
cg11350356	-0.10	0.0001	0.87	0.77	-0.10		IGR	opensea
cg12601909	-0.10	0.0007	0.64	0.54	-0.10	TOLLIP	Body	opensea
cg12639192	-0.10	0.0002	0.81	0.71	-0.10	WDR37	Body	opensea
cg00620190	-0.10	0.0093	0.40	0.30	-0.10	HIST1H4F	TSS1500	shore
cg14642696	-0.10	0.0072	0.29	0.19	-0.10	LMX1B	Body	island
cg01263942	-0.10	0.0231	0.50	0.40	-0.10	DIP2C	Body	opensea
cg02146079	-0.10	0.0017	0.71	0.60	-0.10	PCDHGA2	Body	shore
cg11616411	-0.10	0.0144	0.65	0.55	-0.10	MIR372	TSS1500	opensea
cg19691778	-0.10	0.0006	0.24	0.14	-0.10	GDF6	Body	island
cg07799386	-0.10	0.0103	0.33	0.23	-0.10	PRDM6	Body	island
cg17588578	-0.10	0.0002	0.40	0.30	-0.10	PCDHGA2	Body	island
cg04867115	-0.10	0.0015	0.82	0.72	-0.10		IGR	shore
cg09703114	-0.10	0.0137	0.64	0.54	-0.10	EP400	Body	shore
cg03401519	-0.10	0.0010	0.64	0.54	-0.10		IGR	shore
cg06355870	-0.10	0.0004	0.82	0.72	-0.10	KIRREL3	Body	opensea
cg07152812	-0.10	0.0002	0.80	0.70	-0.10	SPATA13	5'UTR	shore
cg26472684	-0.10	0.0001	0.82	0.72	-0.10		IGR	opensea
cg00520933	-0.10	0.0056	0.38	0.28	-0.10	ZEB1	TSS1500	shore
cg07661704	-0.10	0.0255	0.61	0.51	-0.10	SLC7A11	Body	opensea
cg00095431	-0.10	0.0343	0.47	0.36	-0.10		IGR	opensea
cg07615351	-0.10	0.0127	0.78	0.68	-0.10	ZNRF1	Body	opensea
cg02336364	-0.10	0.0049	0.48	0.38	-0.10	NIPAL3	Body	opensea

cg24842815	-0.10	0.0151	0.52	0.42	-0.10		IGR	opensea
cg00130947	-0.10	0.0190	0.61	0.51	-0.10	LAMA2	Body	opensea
cg09697578	-0.10	0.0081	0.80	0.70	-0.10	FOXK2	Body	shore
cg01148781	-0.10	0.0304	0.53	0.43	-0.10	OSR2	Body	shore
cg00551679	-0.10	0.0135	0.43	0.33	-0.10	FOXF1	3'UTR	shore
cg01572460	-0.10	0.0000	0.66	0.56	-0.10	FBXL7	Body	opensea
cg05512100	-0.10	0.0016	0.34	0.24	-0.10	PDE1C	Body	shore
cg20687616	-0.10	0.0000	0.81	0.71	-0.10		IGR	opensea
cg22559013	-0.10	0.0001	0.51	0.41	-0.10	MGC27382	TSS1500	opensea
cg02755455	-0.10	0.0026	0.86	0.76	-0.10	JAK1	5'UTR	opensea
cg10786043	-0.10	0.0340	0.53	0.43	-0.10	TACC1	TSS1500	shore
cg10575219	-0.10	0.0058	0.64	0.54	-0.10	ATXN7L1	Body	opensea
cg02227813	-0.10	0.0000	0.41	0.31	-0.10	SAMD3	Body	opensea
cg16086579	-0.10	0.0002	0.86	0.76	-0.10	EP400	3'UTR	opensea
cg02467382	-0.10	0.0068	0.72	0.62	-0.10		IGR	opensea
cg27369423	-0.10	0.0010	0.48	0.38	-0.10	DKFZP434H168	TSS1500	shelf
cg27069285	-0.10	0.0351	0.70	0.60	-0.10	GIT2	Body	opensea
cg09321109	-0.10	0.0053	0.75	0.65	-0.10	SLC9A3R2	Body	shore
cg22537334	-0.10	0.0450	0.49	0.39	-0.10	TLE3	Body	opensea
cg20723436	-0.10	0.0164	0.47	0.36	-0.10	NXPH2	Body	opensea
cg01205299	-0.10	0.0001	0.78	0.68	-0.10		IGR	opensea
cg07616499	-0.10	0.0307	0.49	0.39	-0.10	KIAA1462	Body	opensea
cg15507690	-0.10	0.0064	0.80	0.70	-0.10		IGR	shelf
cg16528272	-0.10	0.0057	0.83	0.73	-0.10		IGR	island
cg07104135	-0.10	0.0004	0.63	0.53	-0.10	IPO9	Body	shore
cg07359518	-0.10	0.0001	0.83	0.73	-0.10	EP400	3'UTR	opensea
cg17972213	-0.10	0.0006	0.58	0.48	-0.10	S1PR1	Body	shelf
cg15082166	0.10	0.0117	0.83	0.93	0.10	ZNF608	Body	opensea
cg07886195	0.10	0.0088	0.43	0.53	0.10	SPC24	Body	shelf
cg06876053	0.10	0.0290	0.63	0.73	0.10	SBF2	Body	opensea
cg07004075	0.10	0.0001	0.63	0.73	0.10	REC8	3'UTR	opensea
cg05307141	0.10	0.0070	0.40	0.50	0.10	METTL11B	Body	opensea
cg03980224	0.10	0.0175	0.54	0.64	0.10	RAI14	TSS1500	opensea
cg20640862	0.10	0.0080	0.72	0.82	0.10		IGR	opensea
cg16258657	0.10	0.0124	0.78	0.88	0.10	RBMS3	Body	opensea
cg09360041	0.10	0.0062	0.83	0.93	0.10	ABCA4	TSS1500	opensea
cg10464130	0.10	0.0021	0.34	0.44	0.10	KALRN	Body	shelf
cg15068428	0.10	0.0025	0.76	0.86	0.10	TRIM3	Body	shelf
cg03134230	0.10	0.0024	0.48	0.58	0.10	MYO1B	Body	opensea
cg18816426	0.10	0.0137	0.66	0.76	0.10		IGR	shelf
cg04834502	0.10	0.0021	0.16	0.26	0.10	N4BP1	TSS1500	shore
cg14199833	0.10	0.0072	0.70	0.80	0.10	DAXX	Body	island
cg22330749	0.10	0.0072	0.70	0.80	0.10	GRHL1	Body	opensea

cg06941156	0.10	0.0033	0.82	0.92	0.10	FOXN3	Body	opensea
cg24884820	0.10	0.0046	0.64	0.74	0.10	DNHD1	Body	opensea
cg14722693	0.10	0.0107	0.50	0.60	0.10	CSGALNACT1	5'UTR	opensea
cg18399451	0.10	0.0056	0.64	0.74	0.10	ATXN1	5'UTR	opensea
cg18766468	0.10	0.0002	0.13	0.23	0.10	LRRC15	TSS200	opensea
cg03342032	0.10	0.0149	0.39	0.49	0.10	GPC6	Body	opensea
cg02579668	0.10	0.0024	0.69	0.79	0.10		IGR	opensea
cg15088522	0.10	0.0001	0.71	0.81	0.10		IGR	opensea
cg18914751	0.10	0.0050	0.75	0.85	0.10		IGR	opensea
cg25009842	0.10	0.0024	0.40	0.50	0.10	ARID1B	Body	opensea
cg24516106	0.10	0.0004	0.62	0.73	0.10		IGR	island
cg02579959	0.10	0.0360	0.70	0.80	0.10	FLJ42289	TSS1500	shore
cg25286679	0.10	0.0024	0.60	0.70	0.10		IGR	opensea
cg00325920	0.10	0.0030	0.85	0.95	0.10	PTPRG	Body	opensea
cg02386822	0.10	0.0032	0.51	0.61	0.10	MUC1	TSS200	shore
cg10501210	0.10	0.0076	0.54	0.64	0.10		IGR	opensea
ch.8.969355F	0.10	0.0004	0.05	0.15	0.10	SFRP1	Body	opensea
cg13325904	0.10	0.0032	0.66	0.76	0.10		IGR	opensea
cg21453420	0.10	0.0081	0.57	0.67	0.10		IGR	shelf
cg23852348	0.10	0.0185	0.61	0.71	0.10	PDGFD	Body	shelf
cg19100169	0.10	0.0206	0.41	0.51	0.10	CCNJL	Body	opensea
cg06187336	0.10	0.0328	0.42	0.52	0.10	MAFA	TSS1500	island
cg20419272	0.10	0.0179	0.60	0.70	0.10		IGR	opensea
cg20683445	0.10	0.0039	0.69	0.79	0.10	CAMK1D	Body	opensea
cg14979674	0.10	0.0189	0.31	0.41	0.10	HCN1	3'UTR	opensea
cg15323840	0.10	0.0106	0.62	0.72	0.10	SYNJ2	Body	shore
cg15866542	0.10	0.0003	0.71	0.81	0.10		IGR	opensea
cg10517202	0.10	0.0046	0.80	0.90	0.10		IGR	opensea
cg20935363	0.10	0.0001	0.52	0.62	0.10	PDE2A	Body	island
cg05774698	0.10	0.0135	0.65	0.75	0.10	HIBADH	Body	opensea
cg23684410	0.10	0.0303	0.46	0.56	0.10	SIK3	Body	opensea
cg20026178	0.10	0.0001	0.33	0.43	0.10	WRNIP1	Body	shore
cg20196537	0.10	0.0228	0.33	0.43	0.10		IGR	shore
cg04987335	0.10	0.0248	0.65	0.75	0.10	SLC1A2	Body	opensea
cg18912160	0.10	0.0056	0.46	0.56	0.10		IGR	opensea
cg19725377	0.10	0.0430	0.62	0.72	0.10	DCAF5	Body	opensea
cg00663183	0.10	0.0004	0.71	0.81	0.10		IGR	opensea
cg27375286	0.10	0.0143	0.43	0.53	0.10		IGR	opensea
cg06927337	0.10	0.0032	0.66	0.76	0.10	LFNG	Body	shore
cg07011168	0.10	0.0123	0.57	0.67	0.10	ARHGAP22	Body	opensea
cg01079651	0.10	0.0113	0.53	0.63	0.10	COL18A1	Body	island
cg20726664	0.10	0.0017	0.50	0.60	0.10	AKAP13	Body	opensea
cg18278694	0.10	0.0257	0.68	0.78	0.10	CEP350	5'UTR	opensea

cg10397932	0.10	0.0390	0.35	0.46	0.10	SKI	Body	opensea
cg24877093	0.10	0.0043	0.43	0.53	0.10	PRMT2	Body	island
cg22298430	0.10	0.0376	0.30	0.40	0.10	LOC728392	1stExon	island
cg10837783	0.10	0.0420	0.54	0.64	0.10	LCK	Body	island
cg08155325	0.10	0.0279	0.70	0.80	0.10	PCDHGA4	Body	shore
cg09357589	0.10	0.0348	0.54	0.64	0.10		IGR	island
cg27220401	0.10	0.0010	0.68	0.78	0.10	HDAC7	Body	shore
cg14405137	0.10	0.0000	0.21	0.31	0.10	NCKAP5L	5'UTR	shelf
cg03731202	0.10	0.0093	0.61	0.71	0.10		IGR	opensea
cg08233235	0.10	0.0039	0.50	0.60	0.10	RPL28	Body	shore
cg22943115	0.10	0.0016	0.46	0.56	0.10	PDGFRB	5'UTR	opensea
cg06641342	0.10	0.0322	0.37	0.47	0.10	CYB561	5'UTR	island
cg21818807	0.10	0.0021	0.72	0.82	0.10	RPTOR	Body	shore
cg26719625	0.10	0.0190	0.81	0.91	0.10	TRPV2	Body	opensea
cg22855325	0.10	0.0006	0.11	0.21	0.10		IGR	shore
cg11906021	0.10	0.0171	0.73	0.83	0.10		IGR	opensea
cg00685314	0.10	0.0093	0.60	0.70	0.10	CIT	Body	opensea
cg25278144	0.10	0.0134	0.71	0.81	0.10	TBC1D16	Body	shelf
cg14606082	0.10	0.0019	0.68	0.78	0.10	LFNG	Body	shore
cg17802005	0.10	0.0475	0.69	0.79	0.10		IGR	opensea
cg04231050	0.10	0.0109	0.56	0.66	0.10	BFSP2	Body	opensea
cg18558767	0.10	0.0011	0.27	0.38	0.10	FOXP1	5'UTR	opensea
cg21620078	0.10	0.0215	0.75	0.86	0.10		IGR	opensea
cg15156975	0.10	0.0000	0.13	0.24	0.10		IGR	island
cg10564001	0.10	0.0148	0.63	0.74	0.10		IGR	opensea
cg25856090	0.10	0.0153	0.52	0.62	0.10	CLCN1	Body	opensea
cg04703620	0.10	0.0008	0.61	0.71	0.10		IGR	opensea
cg08742575	0.10	0.0260	0.24	0.35	0.10	C21orf56	5'UTR	shore
cg06062378	0.10	0.0457	0.63	0.73	0.10	MCC	Body	opensea
cg19758859	0.10	0.0071	0.61	0.71	0.10	SASH1	Body	opensea
cg26479630	0.10	0.0075	0.31	0.41	0.10	BTNL9	Body	island
cg04057956	0.10	0.0488	0.53	0.63	0.10	CD9	Body	opensea
cg25310097	0.10	0.0074	0.75	0.85	0.10	ARHGAP24	Body	opensea
cg09620689	0.10	0.0008	0.71	0.81	0.10		IGR	opensea
cg05657292	0.10	0.0102	0.43	0.53	0.10	CECR2	Body	opensea
cg19776072	0.10	0.0044	0.67	0.77	0.10	SMC3	Body	opensea
cg05526364	0.10	0.0105	0.49	0.60	0.10	IFT122	Body	opensea
cg13417862	0.10	0.0047	0.31	0.41	0.10	NXN	Body	opensea
cg01426303	0.10	0.0001	0.42	0.52	0.10	SNAP47	Body	opensea
cg06471491	0.10	0.0032	0.40	0.50	0.10	MIR21	TSS1500	opensea
cg25881591	0.10	0.0026	0.76	0.87	0.10	NTM	TSS1500	shore
cg24074594	0.10	0.0239	0.35	0.45	0.10	FCRLA	TSS200	opensea
cg24877675	0.10	0.0020	0.55	0.65	0.10	JAKMIP3	3'UTR	shelf

cg18891210	0.10	0.0146	0.82	0.92	0.10	ABLIM3	Body	opensea
cg19721326	0.10	0.0002	0.77	0.87	0.10	SGPL1	Body	opensea
cg01718479	0.10	0.0019	0.46	0.56	0.10	NTM	1stExon	opensea
cg00008629	0.10	0.0288	0.67	0.78	0.10	ROD1	Body	shore
cg21479131	0.10	0.0008	0.51	0.61	0.10		IGR	opensea
cg08029299	0.10	0.0021	0.68	0.78	0.10		IGR	opensea
cg24995678	0.10	0.0141	0.68	0.78	0.10	SH3BP4	5'UTR	opensea
cg02930667	0.10	0.0171	0.48	0.58	0.10	CYP27A1	Body	opensea
cg11100581	0.10	0.0145	0.78	0.88	0.10	COL12A1	Body	shore
cg27450668	0.10	0.0125	0.63	0.74	0.10	MEGF6	Body	island
cg13092405	0.10	0.0353	0.32	0.42	0.10		IGR	island
cg03429644	0.10	0.0062	0.38	0.48	0.10		IGR	opensea
cg09584188	0.10	0.0017	0.54	0.64	0.10		IGR	opensea
cg05484949	0.10	0.0007	0.55	0.65	0.10		IGR	opensea
cg19590421	0.10	0.0045	0.29	0.39	0.10		IGR	opensea
cg19962424	0.10	0.0011	0.44	0.54	0.10		IGR	opensea
cg06354695	0.10	0.0380	0.51	0.61	0.10		IGR	opensea
cg11160572	0.10	0.0194	0.83	0.93	0.10	PALLD	Body	opensea
cg10472840	0.10	0.0015	0.74	0.84	0.10		IGR	opensea
cg08015107	0.10	0.0031	0.38	0.48	0.10		IGR	shelf
cg05389174	0.10	0.0115	0.55	0.65	0.10	LOC150197	TSS1500	opensea
cg16516295	0.10	0.0081	0.60	0.70	0.10	C3orf25	TSS1500	shore
cg13100449	0.10	0.0151	0.74	0.84	0.10	FAM129A	Body	opensea
cg05103064	0.10	0.0422	0.71	0.81	0.10	SFMBT2	Body	opensea
cg18082515	0.10	0.0020	0.19	0.29	0.10	MYOF	Body	opensea
cg09684846	0.10	0.0219	0.63	0.73	0.10	FAM18A	TSS200	island
cg04663932	0.10	0.0042	0.19	0.29	0.10	LIMK1	Body	opensea
cg02482730	0.10	0.0271	0.52	0.62	0.10	9-Sep	Body	opensea
cg24368383	0.10	0.0169	0.46	0.56	0.10	MIB2	Body	island
cg02137970	0.10	0.0013	0.77	0.87	0.10		IGR	opensea
cg14562054	0.10	0.0007	0.50	0.60	0.10	RIN1	TSS1500	shelf
cg24804782	0.10	0.0196	0.40	0.51	0.10		IGR	opensea
cg00087792	0.10	0.0154	0.65	0.76	0.10		IGR	opensea
cg11231735	0.10	0.0135	0.51	0.61	0.10		IGR	opensea
cg22242614	0.10	0.0044	0.55	0.66	0.10	SYNJ2	Body	shore
cg19414711	0.10	0.0028	0.65	0.75	0.10	SND1	Body	opensea
cg00443307	0.10	0.0330	0.76	0.86	0.10	KLRG1	TSS1500	opensea
cg05124918	0.10	0.0199	0.57	0.68	0.10		IGR	island
cg17748242	0.10	0.0058	0.69	0.80	0.10	STAT3	5'UTR	opensea
cg17766560	0.10	0.0003	0.31	0.41	0.10	MYO1F	Body	island
cg21814178	0.10	0.0393	0.38	0.48	0.10		IGR	opensea
cg08831077	0.10	0.0019	0.54	0.64	0.10	HRH1	TSS200	opensea
cg10103850	0.10	0.0021	0.23	0.33	0.10	SLC8A1	TSS1500	opensea

cg02153814	0.10	0.0037	0.53	0.64	0.10		IGR	island
cg26645242	0.10	0.0125	0.64	0.74	0.10	CHD9	5'UTR	shore
cg21193484	0.10	0.0016	0.11	0.22	0.10	PRKCH	Body	opensea
cg03119748	0.10	0.0152	0.68	0.79	0.10	CAMK1D	Body	opensea
cg06321596	0.10	0.0007	0.33	0.43	0.10	XYLT1	Body	opensea
cg06050964	0.10	0.0160	0.68	0.78	0.10		IGR	opensea
cg24121069	0.10	0.0028	0.73	0.83	0.10	TMCO3	Body	island
cg17841267	0.10	0.0011	0.63	0.74	0.10		IGR	opensea
cg20184464	0.10	0.0231	0.65	0.75	0.10		IGR	opensea
cg05343665	0.10	0.0362	0.65	0.75	0.10	EPS8	Body	opensea
cg25153741	0.10	0.0345	0.49	0.59	0.10	COL23A1	Body	opensea
cg18837542	0.10	0.0061	0.61	0.71	0.10		IGR	opensea
cg22542731	0.10	0.0053	0.75	0.85	0.10	LIMS2	TSS1500	opensea
cg08206092	0.10	0.0082	0.66	0.76	0.10	SYT17	Body	opensea
cg27171704	0.10	0.0056	0.65	0.75	0.10	TBKBP1	Body	shore
cg15226275	0.10	0.0021	0.41	0.51	0.10	FRK	TSS200	opensea
cg15826437	0.10	0.0219	0.21	0.32	0.10	RAPGEFL1	TSS200	island
cg09030852	0.10	0.0156	0.80	0.90	0.10	CLCN3	Body	opensea
cg27230784	0.10	0.0419	0.29	0.39	0.10	LOC728392	1stExon	island
cg24896460	0.10	0.0080	0.74	0.85	0.10	RGNEF	Body	opensea
cg26233084	0.10	0.0034	0.80	0.90	0.10	FBXL7	Body	opensea
cg25884442	0.10	0.0012	0.56	0.67	0.10	VWF	Body	opensea
cg06267617	0.10	0.0019	0.55	0.66	0.10	DNM3	Body	opensea
cg27092248	0.10	0.0050	0.36	0.46	0.10	HRH1	5'UTR	opensea
cg10907148	0.10	0.0013	0.57	0.67	0.10	C17orf28	Body	opensea
cg15735157	0.10	0.0050	0.35	0.45	0.10		IGR	opensea
cg22635096	0.10	0.0001	0.08	0.18	0.10	ADARB1	5'UTR	shelf
cg14743210	0.10	0.0002	0.71	0.81	0.10		IGR	opensea
cg15796818	0.10	0.0196	0.78	0.88	0.10	PXDN	Body	opensea
cg20909017	0.10	0.0023	0.33	0.44	0.10	ITGA5	Body	opensea
cg06580770	0.10	0.0019	0.76	0.86	0.10	TNXB	Body	shore
cg25912009	0.10	0.0147	0.71	0.82	0.10	AFAP1	Body	opensea
cg20454073	0.10	0.0202	0.59	0.69	0.10	NFIX	Body	island
cg27261733	0.10	0.0004	0.65	0.76	0.10	LSP1	5'UTR	shore
cg24428144	0.10	0.0190	0.37	0.47	0.10		IGR	opensea
cg14733031	0.10	0.0041	0.56	0.66	0.10	B3GNT3	TSS1500	shore
cg22708150	0.10	0.0047	0.62	0.73	0.10	LY6G5C	TSS1500	shore
cg06658404	0.10	0.0311	0.45	0.55	0.10		IGR	opensea
cg13942283	0.10	0.0001	0.14	0.25	0.10	RBM20	Body	opensea
cg05290058	0.10	0.0013	0.25	0.35	0.10	KCNQ1DN	TSS1500	island
cg17655970	0.10	0.0435	0.18	0.29	0.10		IGR	island
cg25820257	0.10	0.0037	0.12	0.23	0.10	RTN2	Body	shore
cg19249516	0.10	0.0189	0.72	0.82	0.10		IGR	opensea

cg01779383	0.10	0.0009	0.16	0.27	0.10		IGR	island
cg08710564	0.10	0.0013	0.58	0.68	0.10	ST5	5'UTR	opensea
cg22636722	0.10	0.0025	0.70	0.80	0.10	RPTOR	Body	shore
cg05079544	0.10	0.0164	0.59	0.70	0.10	N4BP1	TSS1500	shore
cg04865113	0.10	0.0016	0.37	0.47	0.10		IGR	opensea
cg25541653	0.10	0.0109	0.43	0.53	0.10	MPPED2	Body	opensea
cg17505852	0.10	0.0004	0.66	0.76	0.10	NF2	Body	shelf
cg03517250	0.10	0.0082	0.47	0.57	0.10		IGR	shore
cg10733123	0.10	0.0044	0.73	0.83	0.10	RAD51L1	Body	opensea
cg08530036	0.10	0.0002	0.69	0.80	0.10	UBR4	Body	opensea
cg15693937	0.10	0.0269	0.41	0.51	0.10	LOC728723	Body	opensea
cg13341982	0.10	0.0005	0.49	0.60	0.10		IGR	opensea
cg26148904	0.10	0.0000	0.14	0.24	0.10		IGR	opensea
cg17727795	0.10	0.0015	0.67	0.77	0.10		IGR	opensea
cg27492102	0.10	0.0172	0.39	0.49	0.10		IGR	shore
cg00325139	0.10	0.0012	0.72	0.82	0.10		IGR	opensea
cg13088471	0.10	0.0128	0.51	0.62	0.10	ASAP1	Body	opensea
cg27662789	0.10	0.0094	0.74	0.84	0.10		IGR	opensea
cg26017874	0.10	0.0272	0.69	0.79	0.10	REG4	Body	opensea
cg24795748	0.10	0.0000	0.64	0.74	0.10	COL6A1	Body	island
cg10002860	0.10	0.0006	0.68	0.78	0.10	BRSK2	Body	island
cg21175685	0.10	0.0059	0.73	0.83	0.10	COL8A1	5'UTR	opensea
cg19893494	0.10	0.0005	0.61	0.72	0.10	NSMCE2	Body	opensea
cg04964617	0.10	0.0130	0.57	0.68	0.10		IGR	opensea
cg17947992	0.10	0.0009	0.78	0.88	0.10		IGR	opensea
cg14055004	0.10	0.0048	0.60	0.70	0.10		IGR	shelf
cg04605590	0.10	0.0014	0.18	0.28	0.10		IGR	opensea
cg21139587	0.10	0.0010	0.29	0.40	0.10	CSRP1	Body	opensea
cg01643712	0.10	0.0018	0.79	0.89	0.10		IGR	opensea
cg25638443	0.10	0.0175	0.68	0.78	0.10	ST8SIA1	Body	opensea
cg00465319	0.10	0.0035	0.55	0.66	0.10	MYLK	TSS200	opensea
cg18664508	0.10	0.0156	0.29	0.40	0.10	ARPM1	1stExon	island
cg03258139	0.10	0.0291	0.56	0.66	0.10	SMARCA2	TSS1500	shore
cg21476494	0.10	0.0198	0.44	0.55	0.10		IGR	opensea
cg21453378	0.10	0.0030	0.76	0.87	0.10	GAPDHS	TSS200	island
cg07127410	0.10	0.0013	0.64	0.75	0.10	ZNRF3	Body	shore
cg07749442	0.10	0.0014	0.73	0.84	0.10		IGR	opensea
cg00380780	0.10	0.0085	0.77	0.88	0.10		IGR	opensea
cg00243480	0.10	0.0297	0.19	0.29	0.10	NPAS3	Body	island
cg07144408	0.10	0.0058	0.75	0.85	0.10		IGR	opensea
cg06083506	0.10	0.0066	0.54	0.65	0.10		IGR	opensea
cg23640802	0.10	0.0038	0.17	0.28	0.10		IGR	shore
cg14029759	0.10	0.0073	0.51	0.61	0.10	FARS2	Body	opensea

cg18436214	0.10	0.0011	0.59	0.69	0.10		IGR	opensea
cg23270841	0.10	0.0018	0.34	0.45	0.10	PARD3B	Body	opensea
cg05582690	0.10	0.0062	0.72	0.82	0.10		IGR	island
cg09380069	0.10	0.0049	0.63	0.74	0.10	PCTP	Body	opensea
cg15135047	0.10	0.0151	0.37	0.48	0.10		IGR	opensea
cg25468915	0.10	0.0020	0.44	0.55	0.10	RPS6KA2	Body	opensea
cg00781388	0.10	0.0070	0.60	0.71	0.10		IGR	opensea
cg02233459	0.10	0.0070	0.72	0.82	0.10	MEGF6	Body	shore
cg02637282	0.10	0.0459	0.56	0.67	0.10		IGR	opensea
cg08080735	0.10	0.0095	0.31	0.42	0.10	FOXP1	5'UTR	opensea
cg25593573	0.10	0.0102	0.44	0.54	0.10		IGR	shore
cg03116016	0.10	0.0000	0.23	0.33	0.10	C16orf45	Body	opensea
cg09817016	0.10	0.0017	0.30	0.40	0.10	KDM3B	Body	shore
cg04121771	0.10	0.0041	0.68	0.78	0.10	TM4SF4	TSS1500	opensea
cg18489994	0.10	0.0391	0.54	0.64	0.10		IGR	opensea
cg06234051	0.10	0.0072	0.55	0.65	0.10	SOX9	3'UTR	shore
cg13064679	0.10	0.0002	0.50	0.61	0.10	TNXB	Body	shelf
cg15572907	0.10	0.0039	0.38	0.49	0.10	SPTBN4	Body	island
cg11936643	0.10	0.0018	0.28	0.38	0.10	ABLIM1	Body	opensea
cg18418335	0.10	0.0044	0.19	0.30	0.10	MKNK2	Body	island
cg26568031	0.10	0.0204	0.56	0.67	0.10		IGR	opensea
cg01881322	0.10	0.0004	0.17	0.28	0.10	LRRC1	Body	island
cg10753610	0.10	0.0359	0.58	0.68	0.10	ITGB3	Body	shelf
cg09782889	0.10	0.0009	0.13	0.23	0.10		IGR	shore
cg13560548	0.10	0.0071	0.67	0.78	0.10	C3orf24	TSS1500	shore
cg03525467	0.10	0.0011	0.50	0.60	0.10	ATP1A2	Body	opensea
cg26260038	0.10	0.0051	0.37	0.47	0.10	PROM1	TSS200	island
cg14502818	0.10	0.0000	0.78	0.88	0.10		IGR	opensea
cg22325606	0.10	0.0172	0.80	0.91	0.10		IGR	opensea
cg08038033	0.10	0.0013	0.28	0.39	0.10	FOXP1	5'UTR	opensea
cg00352106	0.10	0.0150	0.70	0.81	0.10	MACF1	Body	opensea
cg22077197	0.10	0.0001	0.39	0.49	0.10	HDAC4	Body	opensea
cg06958766	0.10	0.0118	0.49	0.59	0.10	TGFB3	Body	shelf
cg21911930	0.10	0.0100	0.85	0.96	0.10	EPM2A	Body	opensea
cg15394255	0.10	0.0052	0.53	0.64	0.10	MGAT4C	TSS200	opensea
cg10485752	0.10	0.0112	0.42	0.52	0.10	MAN1C1	Body	opensea
cg01188509	0.10	0.0133	0.66	0.76	0.10	IGF2R	Body	shelf
cg22507023	0.10	0.0071	0.52	0.62	0.10	CUEDC1	Body	opensea
cg22418829	0.10	0.0022	0.26	0.37	0.10	BTNL9	Body	island
cg16636721	0.10	0.0160	0.27	0.37	0.10	DIP2A	Body	shore
cg03687650	0.10	0.0083	0.35	0.46	0.10	OSBPL5	Body	shelf
cg23005797	0.10	0.0029	0.74	0.85	0.10	C2orf48	Body	opensea
cg16426293	0.10	0.0056	0.39	0.49	0.10		IGR	island

cg04849985	0.10	0.0101	0.41	0.52	0.10	GNG12	5'UTR	opensea
ch.20.1295406F	0.10	0.0015	0.10	0.20	0.10	PMEPA1	Body	shore
cg03310937	0.10	0.0020	0.51	0.62	0.10		IGR	opensea
cg16707227	0.10	0.0095	0.57	0.67	0.10		IGR	opensea
cg26874367	0.10	0.0445	0.74	0.85	0.10		IGR	opensea
cg24427850	0.10	0.0159	0.59	0.69	0.10		IGR	island
cg15812586	0.10	0.0245	0.78	0.89	0.10		IGR	opensea
cg09030672	0.10	0.0027	0.18	0.28	0.10	HSF4	TSS200	island
cg14325112	0.10	0.0446	0.65	0.75	0.10	GLIS3	Body	shelf
cg20541870	0.10	0.0393	0.25	0.36	0.10	KIAA0652	Body	opensea
cg00024471	0.10	0.0175	0.61	0.71	0.10		IGR	opensea
cg06565975	0.10	0.0063	0.76	0.86	0.10	SLURP1	TSS200	shelf
cg13794390	0.10	0.0000	0.76	0.86	0.10	FOXJ3	Body	opensea
cg24479752	0.10	0.0047	0.40	0.51	0.10	NCRNA00171	Body	opensea
cg01918604	0.10	0.0004	0.64	0.74	0.10	ADORA3	TSS1500	opensea
cg04118124	0.11	0.0210	0.43	0.53	0.11	LEPRE1	Body	shore
cg13492737	0.11	0.0286	0.78	0.89	0.11	COL5A1	Body	opensea
cg19031575	0.11	0.0078	0.25	0.35	0.11	RTN2	5'UTR	shore
cg16745930	0.11	0.0022	0.34	0.44	0.11	HPSE2	3'UTR	opensea
cg11946719	0.11	0.0159	0.21	0.32	0.11		IGR	island
cg26154534	0.11	0.0305	0.42	0.52	0.11	MYEOV2	TSS1500	shore
cg06162751	0.11	0.0003	0.55	0.66	0.11		IGR	opensea
cg27511525	0.11	0.0011	0.72	0.83	0.11	ADAMTS17	Body	opensea
cg07529654	0.11	0.0286	0.49	0.59	0.11	TGIF1	5'UTR	shore
cg08738280	0.11	0.0023	0.50	0.60	0.11	SFT2D2	Body	shore
cg10332003	0.11	0.0000	0.75	0.86	0.11	ENPP6	Body	opensea
cg03001305	0.11	0.0095	0.45	0.55	0.11	STAT5A	TSS200	shore
cg21994818	0.11	0.0112	0.49	0.60	0.11		IGR	opensea
cg23413697	0.11	0.0004	0.09	0.19	0.11		IGR	island
cg09363733	0.11	0.0002	0.14	0.24	0.11	MAT2A	Body	shore
cg11389756	0.11	0.0004	0.66	0.76	0.11	TRIM27	Body	opensea
cg26668675	0.11	0.0224	0.46	0.57	0.11		IGR	island
cg02793828	0.11	0.0044	0.30	0.41	0.11		IGR	shore
cg00316800	0.11	0.0094	0.67	0.77	0.11		IGR	opensea
cg10440877	0.11	0.0031	0.78	0.89	0.11		IGR	opensea
cg08459746	0.11	0.0028	0.64	0.74	0.11		IGR	opensea
cg05339847	0.11	0.0104	0.72	0.83	0.11	ARHGAP12	Body	opensea
cg17504394	0.11	0.0170	0.54	0.65	0.11		IGR	opensea
cg10627737	0.11	0.0019	0.62	0.72	0.11	COL4A2	Body	opensea
cg05633523	0.11	0.0188	0.70	0.80	0.11		IGR	opensea
cg06086177	0.11	0.0023	0.68	0.79	0.11	ZFHX3	5'UTR	shore
cg14659930	0.11	0.0112	0.43	0.53	0.11	ZBTB20	5'UTR	opensea
cg05376465	0.11	0.0327	0.13	0.23	0.11		IGR	opensea

cg25611057	0.11	0.0000	0.75	0.86	0.11		IGR	opensea
cg13132121	0.11	0.0047	0.45	0.55	0.11		IGR	opensea
cg00117018	0.11	0.0076	0.55	0.65	0.11	ZNF251	Body	island
cg26330809	0.11	0.0028	0.76	0.87	0.11		IGR	opensea
ch.22.528917R	0.11	0.0002	0.16	0.27	0.11	HMGXB4	Body	opensea
cg14316629	0.11	0.0429	0.31	0.41	0.11	KIF25	Body	shore
cg16477879	0.11	0.0269	0.53	0.64	0.11	ASB1	Body	shelf
cg18688062	0.11	0.0010	0.49	0.60	0.11	PSORS1C3	TSS1500	shore
cg04785902	0.11	0.0070	0.64	0.75	0.11		IGR	opensea
cg12992827	0.11	0.0010	0.31	0.42	0.11		IGR	opensea
cg00995986	0.11	0.0011	0.32	0.43	0.11	MEIS1	Body	shelf
cg24321971	0.11	0.0048	0.61	0.71	0.11	LGR4	Body	opensea
cg07737135	0.11	0.0045	0.42	0.52	0.11	MUM1	5'UTR	shore
cg23549902	0.11	0.0483	0.37	0.47	0.11		IGR	island
cg06640593	0.11	0.0060	0.55	0.66	0.11		IGR	opensea
cg10639933	0.11	0.0044	0.46	0.57	0.11	SLC25A38	Body	shore
cg18392139	0.11	0.0146	0.66	0.76	0.11		IGR	opensea
cg11894504	0.11	0.0050	0.22	0.33	0.11	MKNK2	Body	island
cg09015246	0.11	0.0169	0.52	0.62	0.11	CIITA	1stExon	shore
cg14656948	0.11	0.0015	0.51	0.62	0.11		IGR	opensea
cg02196592	0.11	0.0023	0.52	0.63	0.11		IGR	opensea
cg17327331	0.11	0.0214	0.57	0.67	0.11	MPP5	5'UTR	opensea
cg03478739	0.11	0.0226	0.44	0.55	0.11	CHN1	Body	opensea
cg23651812	0.11	0.0002	0.72	0.83	0.11	MIR429	TSS200	shelf
cg12358041	0.11	0.0039	0.63	0.73	0.11	GRB10	5'UTR	opensea
cg03745160	0.11	0.0001	0.53	0.64	0.11	ANKLE1	Body	shore
cg02878289	0.11	0.0005	0.73	0.83	0.11	INF2	Body	opensea
cg09217157	0.11	0.0257	0.47	0.58	0.11	ENTPD1	Body	opensea
cg18862597	0.11	0.0120	0.66	0.76	0.11	CROCC	Body	opensea
cg25542041	0.11	0.0004	0.15	0.26	0.11	LHX6	Body	island
cg19833103	0.11	0.0014	0.42	0.53	0.11	LRCH1	TSS1500	shore
cg11728145	0.11	0.0131	0.83	0.94	0.11	PXDN	Body	opensea
cg27193080	0.11	0.0023	0.54	0.64	0.11	IFT122	Body	shore
cg09359103	0.11	0.0166	0.25	0.35	0.11	KCNN3	Body	opensea
cg14656180	0.11	0.0105	0.64	0.75	0.11	COL5A1	Body	opensea
cg24593559	0.11	0.0052	0.17	0.28	0.11	MLLT4	TSS1500	island
cg18332838	0.11	0.0007	0.83	0.93	0.11		IGR	opensea
cg01620165	0.11	0.0010	0.21	0.32	0.11	DTX1	Body	island
cg13860281	0.11	0.0260	0.67	0.77	0.11	VOPP1	Body	shore
cg13582028	0.11	0.0063	0.54	0.64	0.11	ERICH1	Body	opensea
cg23963984	0.11	0.0176	0.28	0.39	0.11	DLG5	Body	opensea
cg22070156	0.11	0.0009	0.70	0.81	0.11	ITGBL1	1stExon	shore
cg26593946	0.11	0.0065	0.14	0.25	0.11	NR2F2	TSS200	shelf

cg23019886	0.11	0.0343	0.24	0.34	0.11		IGR	opensea
cg19192626	0.11	0.0047	0.67	0.78	0.11		IGR	opensea
cg02517337	0.11	0.0015	0.71	0.82	0.11	TSHZ2	Body	shore
cg11326574	0.11	0.0022	0.35	0.45	0.11	LRIG3	Body	opensea
cg17108141	0.11	0.0300	0.51	0.62	0.11	DDAH1	TSS200	shore
cg08356028	0.11	0.0495	0.65	0.76	0.11		IGR	opensea
cg06399735	0.11	0.0007	0.27	0.38	0.11	PSD3	Body	opensea
cg16608498	0.11	0.0066	0.38	0.49	0.11	RNF145	Body	opensea
cg09476092	0.11	0.0149	0.50	0.61	0.11		IGR	shore
cg16243646	0.11	0.0006	0.12	0.23	0.11	PLA2G12A	TSS1500	shore
cg26926221	0.11	0.0053	0.34	0.45	0.11	UTRN	Body	opensea
cg10377510	0.11	0.0031	0.44	0.55	0.11		IGR	opensea
cg00306951	0.11	0.0070	0.83	0.94	0.11		IGR	opensea
cg08848140	0.11	0.0003	0.29	0.39	0.11	LHX6	Body	island
cg25583580	0.11	0.0003	0.34	0.45	0.11	SFRS2	3'UTR	shore
cg16680214	0.11	0.0059	0.15	0.26	0.11	KCNN3	Body	opensea
cg07958502	0.11	0.0153	0.58	0.69	0.11	ITGA11	Body	opensea
cg05581878	0.11	0.0255	0.79	0.90	0.11		IGR	opensea
cg13019306	0.11	0.0249	0.39	0.50	0.11	PRELID2	Body	opensea
cg19609438	0.11	0.0047	0.77	0.88	0.11	VPS13B	Body	opensea
cg06737942	0.11	0.0056	0.71	0.82	0.11	ESYT2	Body	opensea
cg16711983	0.11	0.0202	0.62	0.72	0.11	FAM129A	Body	opensea
cg05497253	0.11	0.0335	0.67	0.78	0.11	LHFPL2	5'UTR	opensea
cg04585669	0.11	0.0065	0.66	0.77	0.11	VGLL4	3'UTR	opensea
cg02968606	0.11	0.0039	0.76	0.87	0.11	BRMS1L	Body	shelf
cg03503642	0.11	0.0135	0.63	0.74	0.11	COL12A1	5'UTR	shore
cg07559178	0.11	0.0445	0.74	0.84	0.11		IGR	opensea
cg06136199	0.11	0.0255	0.26	0.37	0.11	WEE1	Body	shore
cg17569154	0.11	0.0003	0.82	0.93	0.11	DFNA5	5'UTR	opensea
cg09501372	0.11	0.0003	0.20	0.31	0.11	C20orf117	Body	shelf
cg26026748	0.11	0.0041	0.48	0.59	0.11	PBX1	Body	opensea
cg24939196	0.11	0.0203	0.50	0.61	0.11		IGR	opensea
cg25592413	0.11	0.0084	0.51	0.62	0.11	STC2	Body	shore
cg08381046	0.11	0.0011	0.40	0.51	0.11		IGR	opensea
ch.3.2480551F	0.11	0.0002	0.13	0.24	0.11	ZXDC	Body	opensea
cg16364066	0.11	0.0009	0.57	0.68	0.11		IGR	opensea
cg19170009	0.11	0.0032	0.64	0.74	0.11	VIM	Body	opensea
cg13627062	0.11	0.0021	0.16	0.27	0.11		IGR	shore
cg01265860	0.11	0.0291	0.68	0.79	0.11	RUNX1	Body	shelf
cg10397934	0.11	0.0003	0.73	0.84	0.11		IGR	opensea
cg14101485	0.11	0.0031	0.49	0.60	0.11	NFIC	Body	island
cg14538785	0.11	0.0004	0.70	0.80	0.11		IGR	opensea
cg04966159	0.11	0.0020	0.67	0.78	0.11	INF2	Body	opensea

cg06685282	0.11	0.0009	0.13	0.24	0.11		IGR	opensea
cg20572537	0.11	0.0013	0.54	0.65	0.11	LFNG	Body	shore
cg00489394	0.11	0.0048	0.27	0.38	0.11	HRASLS5	TSS1500	shore
cg22157087	0.11	0.0061	0.61	0.72	0.11	ESR1	5'UTR	opensea
cg08654960	0.11	0.0031	0.40	0.51	0.11		IGR	shore
cg08161546	0.11	0.0223	0.66	0.77	0.11	ESR1	TSS1500	opensea
cg08687540	0.11	0.0026	0.62	0.73	0.11	LSP1	5'UTR	shore
ch.12.1023240F	0.11	0.0029	0.13	0.23	0.11	LIMA1	Body	opensea
cg14985891	0.11	0.0031	0.29	0.40	0.11	CASQ2	Body	opensea
cg05201300	0.11	0.0064	0.33	0.43	0.11	ATP6V0E1	Body	opensea
cg26689077	0.11	0.0299	0.63	0.73	0.11	ITGB7	5'UTR	opensea
cg12034869	0.11	0.0002	0.26	0.37	0.11	SLCO3A1	Body	opensea
cg02571055	0.11	0.0023	0.68	0.79	0.11	TULP4	Body	opensea
cg10995873	0.11	0.0030	0.69	0.80	0.11	ZBTB20	5'UTR	opensea
cg27144670	0.11	0.0033	0.53	0.64	0.11	TMOD3	Body	opensea
cg06092869	0.11	0.0014	0.57	0.68	0.11		IGR	opensea
cg22941573	0.11	0.0016	0.71	0.82	0.11	TMEM189	Body	opensea
cg20457051	0.11	0.0032	0.62	0.73	0.11	GPR115	5'UTR	opensea
cg01476969	0.11	0.0002	0.32	0.43	0.11	PALM	Body	island
cg26037936	0.11	0.0111	0.62	0.73	0.11		IGR	opensea
cg27547543	0.11	0.0128	0.48	0.59	0.11		IGR	island
cg01381586	0.11	0.0011	0.19	0.30	0.11	ODC1	5'UTR	shore
cg02583618	0.11	0.0418	0.41	0.51	0.11		IGR	opensea
cg04293307	0.11	0.0034	0.55	0.66	0.11	AXIN2	Body	shelf
cg06055229	0.11	0.0057	0.42	0.53	0.11	ZNF608	Body	opensea
cg10814005	0.11	0.0016	0.26	0.37	0.11	GPR68	5'UTR	opensea
ch.6.1693624F	0.11	0.0003	0.20	0.30	0.11	UBE2CBP	Body	opensea
cg24428389	0.11	0.0015	0.47	0.58	0.11		IGR	opensea
cg11911769	0.11	0.0050	0.59	0.70	0.11	CUX1	Body	opensea
cg11484721	0.11	0.0007	0.65	0.75	0.11		IGR	opensea
cg14414911	0.11	0.0033	0.51	0.62	0.11		IGR	opensea
cg14557288	0.11	0.0029	0.73	0.83	0.11	ADAMTS14	Body	opensea
cg25382821	0.11	0.0021	0.74	0.84	0.11	UBE2E2	Body	opensea
cg16237031	0.11	0.0036	0.48	0.59	0.11	MAP3K7IP2	5'UTR	opensea
cg10134833	0.11	0.0127	0.24	0.35	0.11		IGR	shore
cg03198733	0.11	0.0003	0.74	0.85	0.11	C1S	TSS1500	opensea
cg06710596	0.11	0.0263	0.52	0.63	0.11	GAPDHS	TSS200	island
cg04093633	0.11	0.0198	0.65	0.75	0.11	EFTUD1	Body	opensea
cg01835474	0.11	0.0078	0.71	0.82	0.11		IGR	shelf
cg07229001	0.11	0.0020	0.51	0.62	0.11		IGR	opensea
cg24446429	0.11	0.0009	0.68	0.78	0.11	MBP	Body	shore
cg16763089	0.11	0.0481	0.27	0.37	0.11	LOC149837	TSS200	opensea
cg17345741	0.11	0.0007	0.21	0.32	0.11		IGR	opensea

cg16566943	0.11	0.0025	0.68	0.79	0.11	MUC4	Body	opensea
cg00954105	0.11	0.0096	0.67	0.77	0.11	CCDC46	Body	opensea
cg16874185	0.11	0.0061	0.68	0.79	0.11	GDPD5	5'UTR	opensea
cg19049754	0.11	0.0008	0.59	0.69	0.11		IGR	island
cg26109093	0.11	0.0146	0.63	0.74	0.11		IGR	shelf
cg15044181	0.11	0.0010	0.60	0.70	0.11	IGF2R	Body	opensea
cg17871621	0.11	0.0103	0.19	0.30	0.11		IGR	island
cg25361506	0.11	0.0003	0.56	0.66	0.11		IGR	opensea
cg00974204	0.11	0.0114	0.48	0.59	0.11	UACA	TSS1500	shore
cg14200368	0.11	0.0039	0.66	0.77	0.11	THSD7A	Body	opensea
cg02681842	0.11	0.0087	0.62	0.73	0.11	PLEC1	Body	opensea
cg20981219	0.11	0.0020	0.25	0.36	0.11		IGR	opensea
cg03466587	0.11	0.0187	0.20	0.31	0.11		IGR	opensea
cg00968640	0.11	0.0014	0.81	0.92	0.11	HMCN1	Body	opensea
cg21244135	0.11	0.0000	0.53	0.64	0.11		IGR	opensea
cg11986760	0.11	0.0405	0.52	0.63	0.11		IGR	opensea
cg13829849	0.11	0.0004	0.52	0.63	0.11	MAMDC2	Body	opensea
cg02516419	0.11	0.0072	0.33	0.44	0.11		IGR	opensea
cg21049501	0.11	0.0008	0.64	0.75	0.11	SOX9	Body	island
cg23467079	0.11	0.0000	0.43	0.54	0.11	FAM83A	Body	island
cg23414876	0.11	0.0056	0.24	0.35	0.11	HSF4	5'UTR	island
cg21106486	0.11	0.0070	0.09	0.20	0.11	CR1L	Body	island
cg03274456	0.11	0.0046	0.38	0.48	0.11	AHNAK	Body	opensea
cg23565821	0.11	0.0092	0.31	0.42	0.11	CUTA	Body	shore
cg03018496	0.11	0.0242	0.18	0.29	0.11	NR2F2	TSS200	shelf
cg22172610	0.11	0.0033	0.60	0.71	0.11		IGR	opensea
cg10370305	0.11	0.0087	0.59	0.70	0.11		IGR	opensea
cg23138250	0.11	0.0089	0.31	0.42	0.11		IGR	opensea
cg07244202	0.11	0.0023	0.50	0.61	0.11	ADAM12	Body	opensea
cg25579180	0.11	0.0012	0.07	0.18	0.11	WBSCR17	Body	opensea
cg01368217	0.11	0.0401	0.72	0.83	0.11	ADAM12	Body	opensea
cg10776533	0.11	0.0008	0.37	0.48	0.11	EIF4G1	Body	opensea
cg00850538	0.11	0.0010	0.44	0.55	0.11	CRIM1	TSS1500	shore
cg26120842	0.11	0.0066	0.32	0.43	0.11		IGR	opensea
cg03469682	0.11	0.0064	0.45	0.56	0.11		IGR	opensea
cg08287724	0.11	0.0059	0.30	0.40	0.11	KCNE1	5'UTR	island
cg10582648	0.11	0.0020	0.30	0.41	0.11	IFFO2	TSS1500	island
cg03041650	0.11	0.0077	0.72	0.83	0.11	ROR1	Body	opensea
cg01284595	0.11	0.0014	0.61	0.72	0.11	ZFAND3	Body	opensea
cg24046474	0.11	0.0044	0.30	0.41	0.11	RPL12	Body	shore
cg13503148	0.11	0.0171	0.55	0.66	0.11	TMEM212	TSS200	opensea
cg16049690	0.11	0.0022	0.35	0.46	0.11	BTNL9	Body	island
cg14565465	0.11	0.0301	0.59	0.70	0.11	TMEM2	3'UTR	opensea

cg08478125	0.11	0.0004	0.47	0.58	0.11		IGR	opensea
cg23398076	0.11	0.0007	0.31	0.42	0.11	MEIS1	Body	shelf
cg04308089	0.11	0.0146	0.14	0.25	0.11	NR2F2	TSS200	shelf
cg08189198	0.11	0.0009	0.46	0.57	0.11	NIN	5'UTR	opensea
cg07143863	0.11	0.0061	0.64	0.75	0.11		IGR	opensea
cg20366549	0.11	0.0010	0.20	0.31	0.11	SCNN1A	Body	opensea
cg16962115	0.11	0.0010	0.53	0.64	0.11	LYST	5'UTR	opensea
cg09510531	0.11	0.0304	0.30	0.41	0.11	AIRE	TSS200	island
cg02638523	0.11	0.0030	0.79	0.90	0.11	AGAP1	Body	opensea
cg11606195	0.11	0.0088	0.80	0.91	0.11	GTF2H3	3'UTR	opensea
cg20824939	0.11	0.0377	0.76	0.87	0.11		IGR	opensea
cg20007021	0.11	0.0166	0.12	0.23	0.11	LTB4R2	Body	island
cg14065857	0.11	0.0283	0.35	0.46	0.11	LTBP1	Body	opensea
cg26239233	0.11	0.0077	0.38	0.49	0.11	CIITA	TSS1500	shore
cg06850787	0.11	0.0461	0.45	0.56	0.11		IGR	opensea
cg25298596	0.11	0.0001	0.72	0.83	0.11	LUM	Body	opensea
cg08306614	0.11	0.0117	0.69	0.80	0.11	NUDCD3	3'UTR	opensea
cg07238439	0.11	0.0018	0.53	0.64	0.11	TPCN1	Body	opensea
cg16557204	0.11	0.0115	0.58	0.69	0.11		IGR	opensea
cg10296718	0.11	0.0003	0.79	0.90	0.11	ARHGEF10	Body	opensea
cg15759721	0.11	0.0081	0.42	0.53	0.11	MIR21	Body	opensea
cg14872952	0.11	0.0014	0.22	0.33	0.11		IGR	shore
cg21399203	0.11	0.0267	0.79	0.90	0.11		IGR	opensea
cg20228731	0.11	0.0314	0.80	0.91	0.11	FLJ43663	Body	opensea
cg09941581	0.11	0.0016	0.72	0.83	0.11	SPATA5	Body	opensea
cg17714703	0.11	0.0187	0.36	0.47	0.11	UHRF1	Body	shore
cg09010707	0.11	0.0136	0.61	0.72	0.11		IGR	opensea
cg14031473	0.11	0.0006	0.56	0.67	0.11		IGR	opensea
cg21116284	0.11	0.0044	0.29	0.40	0.11	TGFBR3	Body	opensea
cg20119891	0.11	0.0052	0.67	0.78	0.11	FIBIN	1stExon	opensea
cg01056400	0.11	0.0088	0.33	0.44	0.11		IGR	opensea
cg22349332	0.11	0.0012	0.61	0.72	0.11	MORN1	3'UTR	shore
cg04756252	0.11	0.0009	0.62	0.73	0.11	SLA	3'UTR	opensea
cg16117513	0.11	0.0138	0.55	0.66	0.11	C1orf86	TSS1500	shelf
cg04874782	0.11	0.0001	0.31	0.42	0.11		IGR	opensea
cg06495631	0.11	0.0084	0.26	0.37	0.11	ADAMTS2	Body	opensea
cg00210856	0.11	0.0277	0.64	0.75	0.11	EBF1	Body	opensea
cg26055899	0.11	0.0157	0.63	0.74	0.11	KIAA0922	Body	opensea
cg03374148	0.11	0.0093	0.72	0.83	0.11		IGR	opensea
cg26504835	0.11	0.0170	0.38	0.49	0.11	PSORS1C3	Body	opensea
cg14642392	0.11	0.0348	0.76	0.87	0.11	TNXB	Body	opensea
cg25799589	0.11	0.0071	0.53	0.64	0.11	ZNF827	Body	opensea
cg04095069	0.11	0.0059	0.70	0.81	0.11	NUB1	Body	opensea

cg04228083	0.11	0.0293	0.70	0.81	0.11	LOC100130872-SPON2	TSS200	shore
cg00093080	0.11	0.0022	0.81	0.92	0.11	FBXW8	Body	opensea
cg16288318	0.11	0.0045	0.63	0.74	0.11		IGR	shore
cg10005998	0.11	0.0007	0.29	0.40	0.11	DNMBP	5'UTR	opensea
cg21440776	0.11	0.0312	0.64	0.75	0.11	IFFO1	Body	shore
cg14241836	0.11	0.0049	0.72	0.83	0.11		IGR	shelf
cg04971779	0.11	0.0087	0.25	0.36	0.11	SLC29A1	5'UTR	shore
cg19163395	0.11	0.0008	0.31	0.42	0.11	HDAC5	Body	opensea
cg19600538	0.11	0.0109	0.33	0.44	0.11	SLC44A3	Body	opensea
cg07364927	0.11	0.0119	0.78	0.89	0.11	GALNT2	Body	opensea
cg15972264	0.11	0.0176	0.78	0.89	0.11	NEDD9	Body	opensea
cg25596754	0.11	0.0159	0.80	0.91	0.11	TNXB	Body	opensea
cg07840446	0.11	0.0174	0.56	0.67	0.11	PALLD	Body	opensea
cg09807875	0.11	0.0032	0.44	0.55	0.11	BMPER	Body	opensea
cg10586672	0.11	0.0020	0.70	0.81	0.11	SLC6A6	Body	opensea
cg08198071	0.11	0.0003	0.83	0.94	0.11		IGR	shelf
cg19830657	0.11	0.0015	0.13	0.24	0.11		IGR	island
cg06092502	0.11	0.0143	0.50	0.61	0.11		IGR	island
cg07011029	0.11	0.0005	0.78	0.89	0.11	ARHGEF10	Body	opensea
cg11274172	0.11	0.0313	0.69	0.80	0.11	UVRAG	Body	opensea
cg08219218	0.11	0.0137	0.64	0.75	0.11	LRBA	Body	shore
cg14160433	0.11	0.0136	0.82	0.93	0.11	CAMSAP1	Body	opensea
cg21756208	0.11	0.0008	0.53	0.64	0.11		IGR	opensea
cg09398856	0.11	0.0044	0.72	0.83	0.11		IGR	opensea
cg25838465	0.11	0.0452	0.63	0.74	0.11		IGR	shore
cg06738040	0.11	0.0011	0.35	0.46	0.11		IGR	shelf
cg01323777	0.11	0.0104	0.51	0.63	0.11	KCNAB3	TSS200	island
cg07025785	0.11	0.0256	0.14	0.25	0.11		IGR	opensea
cg02219601	0.11	0.0359	0.53	0.64	0.11		IGR	shelf
cg00715197	0.11	0.0046	0.75	0.86	0.11	RAD51L1	Body	opensea
cg02598079	0.11	0.0263	0.58	0.70	0.11		IGR	shelf
cg17047222	0.11	0.0000	0.69	0.80	0.11	GIGYF1	3'UTR	shelf
cg05226008	0.11	0.0120	0.22	0.33	0.11	DLC1	1stExon	opensea
cg11230112	0.11	0.0061	0.67	0.79	0.11	SYNJ2	Body	opensea
cg27639620	0.11	0.0104	0.29	0.40	0.11	TSPAN4	5'UTR	island
cg09416188	0.11	0.0054	0.44	0.55	0.11	DAG1	5'UTR	opensea
cg08265392	0.11	0.0022	0.80	0.91	0.11	PRKAG2	Body	opensea
cg13492245	0.11	0.0304	0.44	0.55	0.11	FPGS	Body	shore
cg26854588	0.11	0.0025	0.77	0.89	0.11		IGR	shelf
cg16847428	0.11	0.0163	0.72	0.83	0.11		IGR	opensea
cg23085143	0.11	0.0125	0.52	0.63	0.11	SVOPL	Body	island
cg19997196	0.11	0.0102	0.62	0.73	0.11	ADAM12	Body	opensea

cg11942594	0.11	0.0396	0.65	0.76	0.11		IGR	opensea
cg20765408	0.11	0.0426	0.60	0.71	0.11	PARP4	5'UTR	shore
cg09358973	0.11	0.0018	0.27	0.38	0.11	NAV1	1stExon	island
cg22790839	0.11	0.0060	0.60	0.71	0.11		IGR	opensea
cg26828017	0.11	0.0123	0.78	0.90	0.11		IGR	opensea
cg19317600	0.11	0.0059	0.38	0.49	0.11	ACOX3	Body	opensea
cg04794141	0.11	0.0293	0.30	0.41	0.11	TC2N	5'UTR	opensea
cg26125690	0.11	0.0148	0.51	0.62	0.11	DLC1	Body	opensea
cg26988138	0.11	0.0175	0.46	0.57	0.11	GNG7	5'UTR	shelf
cg10692140	0.11	0.0351	0.43	0.54	0.11		IGR	opensea
cg15867307	0.11	0.0135	0.65	0.76	0.11		IGR	opensea
cg09179646	0.11	0.0153	0.51	0.63	0.11		IGR	island
cg04482110	0.11	0.0206	0.13	0.24	0.11	TMEM106A	5'UTR	island
cg07551060	0.11	0.0452	0.76	0.87	0.11	GRK5	Body	opensea
cg09782560	0.11	0.0179	0.21	0.32	0.11		IGR	opensea
cg13857354	0.11	0.0071	0.42	0.53	0.11		IGR	opensea
cg00974944	0.11	0.0008	0.66	0.77	0.11	PTPRG	Body	opensea
cg23088810	0.11	0.0052	0.69	0.80	0.11		IGR	opensea
cg14361033	0.11	0.0009	0.23	0.34	0.11	LHX6	Body	shore
cg09997546	0.11	0.0072	0.44	0.55	0.11	C11orf17	TSS1500	shore
cg13473086	0.11	0.0014	0.69	0.81	0.11	PCDH9	Body	opensea
cg19757382	0.11	0.0005	0.31	0.42	0.11	MYEOV2	TSS1500	shore
cg04638150	0.11	0.0013	0.44	0.55	0.11	AHNAK	Body	opensea
cg00502926	0.11	0.0022	0.56	0.67	0.11	RNASE7	TSS1500	opensea
cg16001865	0.11	0.0027	0.48	0.59	0.11	NFIA	Body	opensea
cg05701478	0.11	0.0015	0.80	0.91	0.11		IGR	opensea
cg08781365	0.11	0.0012	0.59	0.70	0.11		IGR	shore
cg05264870	0.11	0.0188	0.70	0.81	0.11	NRP2	Body	opensea
cg17687367	0.11	0.0366	0.35	0.47	0.11		IGR	opensea
cg03491087	0.11	0.0017	0.76	0.88	0.11	LTBP2	Body	shelf
cg18045100	0.11	0.0037	0.38	0.49	0.11		IGR	opensea
cg26937434	0.11	0.0039	0.73	0.84	0.11	ANKS4B	1stExon	opensea
cg21971800	0.11	0.0105	0.65	0.76	0.11	NXN	Body	opensea
cg09001939	0.11	0.0001	0.58	0.70	0.11	NTSR2	TSS1500	shore
cg05596650	0.11	0.0013	0.75	0.86	0.11		IGR	opensea
cg08994082	0.11	0.0008	0.44	0.55	0.11	GNG7	5'UTR	island
cg12496211	0.11	0.0086	0.74	0.85	0.11	CACNA1C	Body	opensea
cg14675361	0.11	0.0000	0.15	0.26	0.11	LMO7	TSS1500	opensea
cg12253931	0.11	0.0374	0.71	0.82	0.11	STX18	Body	opensea
cg04994217	0.11	0.0005	0.69	0.80	0.11	C9orf170	Body	shore
cg06899522	0.11	0.0092	0.72	0.83	0.11	SPSB1	5'UTR	opensea
cg10771851	0.11	0.0065	0.65	0.76	0.11	LRP5	Body	shore
cg03517776	0.11	0.0468	0.57	0.68	0.11		IGR	shore

cg26449178	0.11	0.0116	0.71	0.82	0.11	SPRY1	3'UTR	opensea
cg21112099	0.11	0.0168	0.57	0.68	0.11	COL12A1	5'UTR	shore
cg03502601	0.11	0.0133	0.53	0.65	0.11	RPTOR	Body	shore
cg18795169	0.11	0.0200	0.06	0.17	0.11		IGR	opensea
cg06963192	0.11	0.0120	0.64	0.75	0.11	C1orf9	Body	opensea
cg02763807	0.11	0.0003	0.61	0.72	0.11	KRBA1	5'UTR	shore
cg10974479	0.11	0.0004	0.72	0.83	0.11	MLN	TSS1500	opensea
cg06786153	0.11	0.0391	0.34	0.45	0.11	TBC1D2B	Body	opensea
cg07816687	0.11	0.0011	0.18	0.29	0.11	HSF4	TSS200	island
cg16550264	0.11	0.0001	0.31	0.42	0.11		IGR	opensea
cg22708961	0.11	0.0024	0.61	0.73	0.11	MORN1	Body	island
cg19478820	0.11	0.0025	0.66	0.77	0.11		IGR	opensea
cg13074458	0.11	0.0037	0.24	0.36	0.11		IGR	opensea
cg22377643	0.11	0.0067	0.31	0.42	0.11		IGR	shore
cg16434331	0.11	0.0085	0.59	0.70	0.11	SLC39A11	Body	opensea
cg00288598	0.11	0.0282	0.63	0.74	0.11	EIF2C2	Body	shelf
cg04619120	0.11	0.0009	0.63	0.74	0.11	ASPH	Body	opensea
cg03272066	0.11	0.0088	0.55	0.66	0.11		IGR	opensea
cg26147311	0.11	0.0047	0.74	0.85	0.11		IGR	opensea
cg26992634	0.11	0.0112	0.51	0.62	0.11	UBTD2	Body	shore
cg21303763	0.11	0.0010	0.17	0.29	0.11	TSPAN9	5'UTR	island
cg01550348	0.11	0.0081	0.69	0.81	0.11	DDAH1	Body	opensea
cg21870038	0.11	0.0035	0.28	0.40	0.11	RFFL	1stExon	opensea
cg07393854	0.11	0.0003	0.72	0.83	0.11	CCDC50	Body	opensea
cg24820936	0.11	0.0004	0.52	0.63	0.11	RNF19A	3'UTR	opensea
cg13911697	0.11	0.0033	0.66	0.77	0.11		IGR	opensea
cg26986871	0.11	0.0083	0.47	0.58	0.11		IGR	opensea
cg15928106	0.11	0.0214	0.79	0.90	0.11	FLJ43663	Body	opensea
cg04157658	0.11	0.0069	0.63	0.74	0.11		IGR	opensea
cg06676119	0.11	0.0028	0.69	0.80	0.11	PDE1A	Body	opensea
cg12991093	0.11	0.0082	0.66	0.78	0.11	NNT	Body	opensea
cg24631102	0.11	0.0008	0.63	0.74	0.11	NPS	Body	opensea
cg08878651	0.11	0.0007	0.70	0.82	0.11		IGR	opensea
cg07582825	0.11	0.0123	0.41	0.52	0.11		IGR	opensea
cg10167316	0.11	0.0141	0.70	0.81	0.11	SEC61A1	Body	opensea
cg13978777	0.11	0.0026	0.64	0.75	0.11		IGR	shelf
cg18577239	0.11	0.0102	0.78	0.89	0.11	ATF7	Body	opensea
cg22701603	0.11	0.0091	0.53	0.65	0.11		IGR	island
cg15964672	0.11	0.0472	0.76	0.87	0.11		IGR	opensea
cg08423142	0.11	0.0119	0.65	0.77	0.11	MYO1E	Body	opensea
cg25899154	0.11	0.0009	0.52	0.63	0.11		IGR	opensea
cg19628739	0.11	0.0002	0.31	0.43	0.11		IGR	shelf
cg00786406	0.11	0.0192	0.37	0.49	0.11	HIPK1	5'UTR	shore

cg26929700	0.11	0.0003	0.37	0.48	0.11	ZNF423	Body	island
cg14927126	0.11	0.0001	0.42	0.54	0.11	SIK3	Body	opensea
cg20332503	0.11	0.0129	0.49	0.60	0.11	ZYX	Body	shelf
cg22218543	0.11	0.0000	0.09	0.20	0.11	ZNF652	Body	opensea
cg08035694	0.11	0.0048	0.70	0.81	0.11		IGR	opensea
cg14340889	0.11	0.0123	0.68	0.79	0.11	LTBP2	Body	opensea
cg22631616	0.11	0.0245	0.70	0.82	0.11	TBC1D9	Body	opensea
cg04604708	0.11	0.0022	0.69	0.80	0.11	CREB3L2	Body	opensea
ch.2.884792F	0.11	0.0000	0.08	0.19	0.11	CRIM1	Body	opensea
cg05502349	0.11	0.0002	0.64	0.76	0.11	MPP2	Body	opensea
cg11767757	0.11	0.0109	0.49	0.60	0.11	NCRNA00114	TSS200	opensea
cg24847366	0.11	0.0025	0.45	0.57	0.11		IGR	opensea
cg03432241	0.11	0.0025	0.71	0.82	0.11	ITIH5	Body	opensea
cg17486314	0.11	0.0188	0.37	0.48	0.11	BACH2	5'UTR	opensea
cg07546943	0.11	0.0077	0.63	0.74	0.11	AGAP1	Body	opensea
cg03873153	0.11	0.0102	0.27	0.39	0.11		IGR	shelf
cg02739280	0.11	0.0127	0.80	0.91	0.11	NAV2	Body	opensea
cg14398228	0.11	0.0166	0.61	0.73	0.11	VRK1	Body	opensea
cg22626506	0.11	0.0040	0.78	0.90	0.11	KANK2	Body	shore
cg02934719	0.11	0.0014	0.47	0.58	0.11	TK2	3'UTR	opensea
cg23780146	0.11	0.0001	0.58	0.69	0.11		IGR	opensea
cg25642955	0.11	0.0088	0.57	0.68	0.11		IGR	opensea
cg21074766	0.11	0.0064	0.56	0.68	0.11		IGR	opensea
cg21144009	0.11	0.0466	0.42	0.54	0.11	PLXNA2	Body	opensea
cg05606089	0.11	0.0490	0.62	0.74	0.11	AP3B1	Body	opensea
cg13821077	0.11	0.0002	0.32	0.44	0.11	TLN2	Body	opensea
cg27097018	0.11	0.0002	0.12	0.24	0.11	F13A1	5'UTR	opensea
cg19778776	0.12	0.0431	0.56	0.68	0.12	DYRK3	TSS1500	shore
cg15033552	0.12	0.0060	0.70	0.82	0.12	CAPN2	TSS200	opensea
cg09929879	0.12	0.0065	0.58	0.69	0.12	DIP2C	Body	shelf
cg12394201	0.12	0.0319	0.46	0.58	0.12		IGR	opensea
cg00598204	0.12	0.0032	0.61	0.73	0.12	ASPSCR1	Body	shore
cg03580292	0.12	0.0026	0.56	0.68	0.12		IGR	opensea
cg23021584	0.12	0.0159	0.66	0.78	0.12	NCOR2	Body	shelf
cg14032261	0.12	0.0086	0.79	0.90	0.12		IGR	opensea
cg07267600	0.12	0.0042	0.15	0.27	0.12	CACNA1C	Body	opensea
cg06159404	0.12	0.0125	0.21	0.32	0.12		IGR	island
cg12807764	0.12	0.0101	0.75	0.87	0.12		IGR	opensea
cg25951430	0.12	0.0061	0.23	0.35	0.12	SLC43A3	Body	shore
cg14891195	0.12	0.0478	0.41	0.53	0.12	KLHL14	TSS200	shore
cg13585930	0.12	0.0126	0.57	0.68	0.12	NPFFR1	TSS1500	opensea
cg14463068	0.12	0.0059	0.62	0.74	0.12	MYT1L	Body	opensea
cg15867652	0.12	0.0277	0.62	0.74	0.12	BAIAP2	Body	shore

cg23093090	0.12	0.0044	0.56	0.67	0.12	C10orf26	3'UTR	opensea
cg19071976	0.12	0.0161	0.72	0.84	0.12	TNXB	Body	opensea
cg02174092	0.12	0.0098	0.15	0.27	0.12		IGR	island
cg06730183	0.12	0.0011	0.81	0.93	0.12		IGR	opensea
cg10883064	0.12	0.0229	0.53	0.64	0.12	MRC2	Body	island
cg18942579	0.12	0.0018	0.18	0.30	0.12	TMEM49	Body	opensea
cg20325547	0.12	0.0066	0.63	0.74	0.12		IGR	shelf
cg16225663	0.12	0.0231	0.77	0.88	0.12	TNXB	Body	opensea
cg11560600	0.12	0.0110	0.69	0.80	0.12		IGR	opensea
cg02184280	0.12	0.0054	0.17	0.29	0.12	PIH1D1	Body	shore
cg21817750	0.12	0.0189	0.64	0.75	0.12	MRPS27	Body	opensea
cg06479057	0.12	0.0040	0.78	0.90	0.12	CCDC80	Body	opensea
cg27661394	0.12	0.0200	0.65	0.77	0.12		IGR	opensea
cg10987840	0.12	0.0433	0.60	0.71	0.12		IGR	opensea
cg02784232	0.12	0.0005	0.60	0.72	0.12	PHC3	Body	shore
cg14520913	0.12	0.0143	0.66	0.77	0.12	NNMT	TSS200	opensea
cg07985116	0.12	0.0148	0.51	0.63	0.12	LRP5	Body	shore
cg08043565	0.12	0.0034	0.32	0.44	0.12	MICALCL	1stExon	opensea
cg13828068	0.12	0.0003	0.80	0.92	0.12	RMI1	5'UTR	opensea
cg02259760	0.12	0.0006	0.64	0.76	0.12		IGR	opensea
cg04477962	0.12	0.0286	0.62	0.73	0.12	METTL7A	TSS1500	opensea
cg02074274	0.12	0.0270	0.58	0.69	0.12	PDXK	3'UTR	shore
cg15376097	0.12	0.0000	0.14	0.25	0.12	MPZL2	TSS1500	opensea
cg15181598	0.12	0.0013	0.18	0.30	0.12		IGR	island
cg10900313	0.12	0.0217	0.69	0.81	0.12	SH3D19	1stExon	opensea
cg17618872	0.12	0.0063	0.43	0.55	0.12	CBFA2T2	3'UTR	shelf
cg19036773	0.12	0.0005	0.36	0.48	0.12	MLL3	Body	opensea
cg19945931	0.12	0.0118	0.41	0.52	0.12		IGR	island
cg07041720	0.12	0.0001	0.60	0.72	0.12	LFNG	Body	shore
cg26549084	0.12	0.0004	0.67	0.79	0.12	FNDC3B	5'UTR	shore
cg04287574	0.12	0.0019	0.23	0.35	0.12	NAV1	Body	island
ch.6.2925136R	0.12	0.0031	0.25	0.37	0.12	MTHFD1L	Body	opensea
cg14608275	0.12	0.0013	0.47	0.59	0.12	NCEH1	TSS1500	shore
cg13359998	0.12	0.0390	0.40	0.51	0.12	GALNT2	Body	opensea
cg03665360	0.12	0.0017	0.20	0.31	0.12		IGR	opensea
cg13997435	0.12	0.0087	0.46	0.58	0.12	S100A2	TSS200	opensea
cg00453735	0.12	0.0013	0.70	0.82	0.12		IGR	opensea
cg21351647	0.12	0.0011	0.65	0.77	0.12	PSORS1C1	TSS1500	opensea
cg07450552	0.12	0.0002	0.13	0.24	0.12	DNAJC18	TSS1500	opensea
cg19793499	0.12	0.0055	0.65	0.76	0.12	AUTS2	Body	island
cg07111834	0.12	0.0020	0.43	0.55	0.12	PTPN22	Body	opensea
cg03922381	0.12	0.0024	0.64	0.76	0.12		IGR	opensea
cg02279108	0.12	0.0044	0.66	0.77	0.12		IGR	shelf

cg16449636	0.12	0.0027	0.77	0.89	0.12		IGR	opensea
cg15319576	0.12	0.0306	0.74	0.86	0.12		IGR	opensea
cg05197667	0.12	0.0060	0.70	0.81	0.12	SRGAP1	Body	opensea
cg07095347	0.12	0.0172	0.63	0.74	0.12		IGR	opensea
cg25599924	0.12	0.0028	0.40	0.52	0.12	ACOX3	Body	opensea
cg15723028	0.12	0.0177	0.58	0.70	0.12	RIPK2	Body	opensea
cg27582527	0.12	0.0049	0.60	0.72	0.12		IGR	shore
cg03515844	0.12	0.0078	0.58	0.70	0.12	ZBTB17	5'UTR	opensea
cg13125510	0.12	0.0006	0.49	0.61	0.12		IGR	opensea
cg07201475	0.12	0.0401	0.68	0.80	0.12		IGR	opensea
cg24116317	0.12	0.0072	0.62	0.73	0.12	STK10	Body	opensea
cg02499308	0.12	0.0008	0.72	0.84	0.12	ACMSD	TSS1500	opensea
cg16709353	0.12	0.0001	0.25	0.37	0.12	TPCN2	3'UTR	opensea
cg05032848	0.12	0.0048	0.28	0.40	0.12	RFFL	TSS200	opensea
cg08854834	0.12	0.0026	0.29	0.40	0.12	C21orf7	Body	opensea
cg07241084	0.12	0.0051	0.26	0.38	0.12	TNS3	5'UTR	opensea
cg18907942	0.12	0.0035	0.73	0.85	0.12		IGR	opensea
cg23290313	0.12	0.0022	0.32	0.44	0.12	ST3GAL3	Body	opensea
cg10888811	0.12	0.0008	0.64	0.76	0.12	DTYMK	Body	shelf
cg20744756	0.12	0.0141	0.65	0.77	0.12		IGR	opensea
cg06601203	0.12	0.0003	0.37	0.48	0.12		IGR	opensea
cg24475171	0.12	0.0090	0.44	0.55	0.12	C9orf78	TSS1500	shore
cg01280180	0.12	0.0004	0.47	0.59	0.12	TXNDC6	5'UTR	shore
cg26235990	0.12	0.0243	0.56	0.68	0.12		IGR	opensea
cg05279761	0.12	0.0293	0.60	0.71	0.12	ACAD11	Body	opensea
cg15690347	0.12	0.0101	0.21	0.33	0.12	SPIB	Body	island
cg18966688	0.12	0.0072	0.60	0.72	0.12	AUTS2	Body	island
cg13490403	0.12	0.0019	0.18	0.30	0.12	LHX6	Body	island
cg11828470	0.12	0.0002	0.11	0.23	0.12	NLRP1	Body	opensea
cg21846949	0.12	0.0161	0.78	0.89	0.12	LTBP1	Body	opensea
cg09628359	0.12	0.0086	0.59	0.71	0.12	SLC18A1	5'UTR	opensea
cg04506114	0.12	0.0039	0.61	0.73	0.12	SOAT1	Body	opensea
cg18758987	0.12	0.0031	0.43	0.55	0.12		IGR	opensea
cg24587080	0.12	0.0133	0.65	0.77	0.12		IGR	opensea
cg01621390	0.12	0.0008	0.65	0.77	0.12		IGR	opensea
cg23670353	0.12	0.0363	0.58	0.70	0.12	FGGY	Body	opensea
cg16474118	0.12	0.0007	0.63	0.75	0.12		IGR	opensea
cg06096901	0.12	0.0008	0.65	0.77	0.12	RPTOR	Body	shelf
cg24334149	0.12	0.0021	0.49	0.60	0.12	ATP6V1B1	Body	opensea
cg26806779	0.12	0.0240	0.59	0.70	0.12	KLRG1	TSS1500	opensea
cg09442613	0.12	0.0020	0.72	0.83	0.12		IGR	opensea
cg18418242	0.12	0.0004	0.71	0.83	0.12		IGR	opensea
cg10063663	0.12	0.0008	0.60	0.71	0.12	BAIAP2	Body	opensea

cg05590451	0.12	0.0036	0.39	0.51	0.12	DCUN1D3	5'UTR	opensea
cg10082398	0.12	0.0040	0.59	0.71	0.12	CLDN20	5'UTR	opensea
cg07126783	0.12	0.0105	0.65	0.77	0.12	RPTOR	Body	shore
cg04737350	0.12	0.0018	0.65	0.77	0.12	PPP1R12A	Body	opensea
cg16421411	0.12	0.0001	0.73	0.85	0.12	C2orf48	Body	opensea
cg09169117	0.12	0.0004	0.48	0.60	0.12	FMNL2	Body	opensea
cg00741954	0.12	0.0009	0.55	0.67	0.12	GMDS	Body	opensea
cg17516247	0.12	0.0042	0.59	0.71	0.12		IGR	opensea
cg00701951	0.12	0.0112	0.79	0.91	0.12	HTRA1	Body	opensea
cg20389635	0.12	0.0016	0.40	0.52	0.12	PTHLH	Body	shelf
cg15972148	0.12	0.0189	0.68	0.80	0.12	RASA3	Body	shore
cg01723892	0.12	0.0048	0.48	0.60	0.12		IGR	opensea
cg14423778	0.12	0.0028	0.48	0.60	0.12	MBNL1	TSS1500	shore
cg16497661	0.12	0.0003	0.59	0.70	0.12	CKB	Body	shore
cg06938601	0.12	0.0000	0.53	0.65	0.12	TCERG1L	Body	opensea
cg19223064	0.12	0.0065	0.67	0.79	0.12		IGR	shelf
cg10729496	0.12	0.0002	0.73	0.84	0.12	C3orf24	TSS200	island
cg06822067	0.12	0.0157	0.60	0.72	0.12		IGR	opensea
cg21341928	0.12	0.0151	0.30	0.42	0.12	PABPC3	1stExon	island
cg14308254	0.12	0.0017	0.58	0.70	0.12	FAM83A	Body	island
cg10817669	0.12	0.0018	0.68	0.80	0.12		IGR	opensea
cg20488673	0.12	0.0287	0.60	0.72	0.12		IGR	opensea
cg07600533	0.12	0.0074	0.22	0.34	0.12	KLHDC7B	TSS1500	island
cg19798881	0.12	0.0027	0.51	0.63	0.12	BMP6	Body	opensea
cg01356872	0.12	0.0044	0.72	0.84	0.12		IGR	opensea
cg26180080	0.12	0.0155	0.13	0.25	0.12		IGR	opensea
cg07791418	0.12	0.0003	0.53	0.65	0.12		IGR	opensea
cg15165154	0.12	0.0021	0.35	0.47	0.12	CXXC5	5'UTR	island
cg05575505	0.12	0.0397	0.46	0.57	0.12		IGR	opensea
cg16041798	0.12	0.0020	0.81	0.93	0.12	MTHFD1	Body	opensea
cg20786131	0.12	0.0008	0.28	0.40	0.12	CABLES1	TSS1500	shore
cg01543184	0.12	0.0000	0.23	0.35	0.12	MAFG	5'UTR	shore
cg15443403	0.12	0.0004	0.15	0.27	0.12	THUMPD3	TSS1500	shore
cg20742298	0.12	0.0009	0.71	0.83	0.12		IGR	opensea
cg18166947	0.12	0.0019	0.18	0.30	0.12	BCL2L13	Body	opensea
cg12466599	0.12	0.0017	0.66	0.78	0.12	ALPL	Body	opensea
cg24597774	0.12	0.0003	0.68	0.80	0.12	SPSB1	5'UTR	opensea
cg24392939	0.12	0.0240	0.73	0.85	0.12		IGR	opensea
cg14606478	0.12	0.0004	0.66	0.78	0.12	TRIM26	5'UTR	opensea
cg04367486	0.12	0.0096	0.30	0.42	0.12	CD200	1stExon	island
cg02459469	0.12	0.0001	0.39	0.51	0.12		IGR	opensea
cg23482132	0.12	0.0030	0.79	0.91	0.12	FMNL2	Body	opensea
cg09530108	0.12	0.0041	0.69	0.81	0.12	CRIM1	Body	shore

cg08748308	0.12	0.0022	0.50	0.62	0.12	LCP2	Body	opensea
cg16215913	0.12	0.0016	0.27	0.39	0.12		IGR	opensea
cg23732024	0.12	0.0139	0.60	0.72	0.12	LY96	Body	opensea
cg19815565	0.12	0.0382	0.32	0.44	0.12	LOC100130522	Body	shore
cg04384689	0.12	0.0021	0.77	0.89	0.12		IGR	shore
cg13104185	0.12	0.0048	0.38	0.50	0.12	RERE	Body	opensea
cg11424376	0.12	0.0024	0.60	0.72	0.12	GALNT2	Body	opensea
cg14088282	0.12	0.0440	0.48	0.60	0.12		IGR	opensea
cg03191504	0.12	0.0023	0.45	0.57	0.12	RNF149	Body	shore
cg24834394	0.12	0.0463	0.78	0.90	0.12		IGR	opensea
cg13787850	0.12	0.0408	0.47	0.59	0.12		IGR	opensea
cg19409133	0.12	0.0153	0.71	0.83	0.12	SNHG3-RCC1	Body	shore
cg27588356	0.12	0.0002	0.71	0.83	0.12	MAP3K4	Body	opensea
cg04098052	0.12	0.0202	0.45	0.57	0.12		IGR	opensea
cg08888203	0.12	0.0033	0.73	0.85	0.12	C3orf24	TSS200	island
cg16558432	0.12	0.0049	0.24	0.36	0.12	LOC728392	1stExon	island
cg17092065	0.12	0.0087	0.60	0.72	0.12	SMAD3	Body	opensea
cg01135464	0.12	0.0001	0.70	0.82	0.12		IGR	opensea
cg24597131	0.12	0.0001	0.70	0.82	0.12	KIAA1026	Body	opensea
cg14455403	0.12	0.0006	0.68	0.80	0.12	LMCD1	Body	opensea
cg14222479	0.12	0.0052	0.19	0.31	0.12	ARPM1	1stExon	shore
cg23091302	0.12	0.0014	0.47	0.59	0.12	KCNH8	Body	opensea
cg02683114	0.12	0.0019	0.71	0.83	0.12	C2orf84	Body	island
cg25408008	0.12	0.0010	0.36	0.48	0.12	MIER1	5'UTR	shore
cg00159243	0.12	0.0000	0.35	0.47	0.12	SELPLG	5'UTR	opensea
cg17220055	0.12	0.0044	0.68	0.80	0.12	HIVEP3	5'UTR	opensea
cg02153681	0.12	0.0024	0.70	0.83	0.12		IGR	opensea
cg03747456	0.12	0.0005	0.25	0.37	0.12	KRT80	TSS1500	opensea
cg14535332	0.12	0.0027	0.16	0.28	0.12	KCNE1	TSS200	island
cg14111334	0.12	0.0004	0.66	0.78	0.12		IGR	opensea
cg08908002	0.12	0.0004	0.70	0.82	0.12		IGR	shelf
cg15645287	0.12	0.0149	0.58	0.70	0.12	PARD3	Body	opensea
cg22256354	0.12	0.0007	0.51	0.63	0.12		IGR	opensea
cg17411016	0.12	0.0014	0.25	0.37	0.12		IGR	opensea
cg26828599	0.12	0.0135	0.73	0.85	0.12	TPCN2	Body	opensea
cg09264231	0.12	0.0002	0.25	0.37	0.12		IGR	opensea
cg06121808	0.12	0.0003	0.46	0.58	0.12	SLC20A1	Body	shore
cg02352281	0.12	0.0157	0.60	0.72	0.12		IGR	opensea
cg04171808	0.12	0.0234	0.78	0.90	0.12	CD44	Body	opensea
cg14387312	0.12	0.0008	0.48	0.60	0.12		IGR	opensea
cg08312765	0.12	0.0006	0.44	0.56	0.12		IGR	opensea
cg25565203	0.12	0.0004	0.38	0.50	0.12		IGR	opensea
cg05573767	0.12	0.0154	0.43	0.55	0.12	RPS6KA2	Body	opensea

cg03535079	0.12	0.0000	0.71	0.83	0.12	KIAA0146	Body	opensea
cg01351822	0.12	0.0020	0.06	0.18	0.12	UNC45A	5'UTR	island
cg20893936	0.12	0.0012	0.62	0.74	0.12	TBC1D8	Body	opensea
cg24199384	0.12	0.0053	0.51	0.63	0.12	MIPEP	Body	opensea
cg07600721	0.12	0.0320	0.76	0.89	0.12		IGR	opensea
cg27159987	0.12	0.0043	0.64	0.76	0.12	ESYT2	Body	opensea
cg18608055	0.12	0.0013	0.58	0.71	0.12	SBNO2	Body	opensea
ch.2.16090152F	0.12	0.0033	0.18	0.30	0.12		IGR	opensea
cg10373607	0.12	0.0000	0.80	0.92	0.12	TSHZ2	Body	shelf
cg12999120	0.12	0.0005	0.73	0.85	0.12		IGR	opensea
cg16713321	0.12	0.0009	0.53	0.65	0.12		IGR	opensea
cg05476182	0.12	0.0001	0.44	0.56	0.12	PHF15	Body	opensea
cg10115368	0.12	0.0082	0.72	0.84	0.12		IGR	shore
cg09173378	0.12	0.0120	0.57	0.69	0.12	KCNT2	Body	opensea
cg22790835	0.12	0.0005	0.40	0.52	0.12	C13orf29	TSS1500	opensea
cg11526198	0.12	0.0000	0.12	0.25	0.12	E2F2	Body	shelf
cg16852369	0.12	0.0154	0.61	0.73	0.12	ARHGEF10	Body	opensea
cg14036627	0.12	0.0485	0.46	0.58	0.12		IGR	shore
cg27280535	0.12	0.0004	0.16	0.28	0.12	MFHAS1	Body	shore
cg25369262	0.12	0.0000	0.11	0.23	0.12	TSPAN1	Body	opensea
cg07633317	0.12	0.0013	0.30	0.42	0.12	ZNF423	Body	island
cg00731232	0.12	0.0010	0.29	0.41	0.12		IGR	shore
cg02847220	0.12	0.0025	0.59	0.71	0.12	PLD1	5'UTR	opensea
cg15989892	0.12	0.0012	0.51	0.63	0.12	APBB2	5'UTR	opensea
cg16620032	0.12	0.0001	0.21	0.33	0.12	GPR4	5'UTR	shore
cg17694130	0.12	0.0170	0.34	0.46	0.12		IGR	opensea
cg05389236	0.12	0.0014	0.46	0.58	0.12		IGR	opensea
cg08717751	0.12	0.0029	0.74	0.86	0.12		IGR	opensea
cg01078772	0.12	0.0016	0.49	0.61	0.12	KIF3B	3'UTR	opensea
cg18277682	0.12	0.0302	0.79	0.92	0.12	C1orf69	Body	island
cg03950253	0.12	0.0104	0.52	0.65	0.12	BPGM	5'UTR	opensea
cg01704698	0.12	0.0213	0.58	0.70	0.12	CRISPLD2	3'UTR	opensea
cg24036791	0.12	0.0075	0.75	0.87	0.12	GALNTL4	Body	opensea
cg17452301	0.12	0.0000	0.26	0.38	0.12	PWWP2B	Body	shore
cg22926842	0.12	0.0031	0.59	0.71	0.12	CENPM	TSS1500	opensea
cg01940181	0.12	0.0002	0.45	0.57	0.12	PRDM16	Body	opensea
cg14016236	0.12	0.0013	0.27	0.39	0.12	FNDC3B	5'UTR	opensea
cg05256605	0.12	0.0227	0.53	0.65	0.12	LOX	Body	shore
cg10442157	0.12	0.0125	0.26	0.39	0.12	SYT7	3'UTR	island
cg25053413	0.12	0.0005	0.16	0.28	0.12	C14orf182	Body	shelf
cg03069731	0.12	0.0007	0.58	0.70	0.12	ST7	Body	opensea
cg12235073	0.12	0.0007	0.47	0.60	0.12	PFKFB3	Body	opensea
cg05194636	0.12	0.0217	0.74	0.87	0.12		IGR	opensea

cg06680511	0.12	0.0465	0.49	0.61	0.12		IGR	shore
cg01433955	0.12	0.0012	0.68	0.80	0.12		IGR	shore
cg02976588	0.12	0.0007	0.24	0.36	0.12		IGR	opensea
cg16451306	0.12	0.0004	0.55	0.67	0.12	MAP1B	Body	opensea
cg17462560	0.12	0.0010	0.45	0.58	0.12	LHFPL2	5'UTR	opensea
cg24116380	0.12	0.0004	0.21	0.33	0.12		IGR	opensea
cg01178099	0.12	0.0056	0.38	0.50	0.12		IGR	opensea
cg06160606	0.12	0.0015	0.80	0.93	0.12	PDE4B	Body	opensea
cg25546651	0.12	0.0054	0.74	0.86	0.12		IGR	opensea
cg15360181	0.12	0.0115	0.38	0.51	0.12	SLC9A9	TSS200	opensea
cg00633768	0.12	0.0006	0.56	0.69	0.12	GPR133	Body	opensea
cg23196346	0.12	0.0017	0.43	0.55	0.12	KIAA1217	Body	opensea
cg08409167	0.12	0.0010	0.69	0.81	0.12	CSNK1G1	Body	opensea
cg20788020	0.12	0.0058	0.65	0.78	0.12		IGR	opensea
cg25139649	0.12	0.0233	0.42	0.55	0.12	SKI	Body	opensea
cg19542542	0.12	0.0205	0.71	0.83	0.12	APBB2	5'UTR	opensea
cg25515997	0.12	0.0156	0.57	0.69	0.12	ANXA2	Body	opensea
cg12667792	0.12	0.0037	0.55	0.67	0.12	ZNF366	5'UTR	opensea
cg03071808	0.12	0.0000	0.09	0.21	0.12		IGR	opensea
cg10051588	0.12	0.0047	0.80	0.92	0.12	FLJ39653	Body	shelf
cg20743134	0.12	0.0080	0.39	0.51	0.12	C3orf64	5'UTR	shore
cg23512231	0.12	0.0097	0.71	0.83	0.12		IGR	opensea
cg14462670	0.12	0.0000	0.12	0.24	0.12		IGR	shelf
cg25161129	0.12	0.0039	0.33	0.46	0.12	WIPI1	Body	shore
cg18919017	0.12	0.0005	0.47	0.59	0.12	DPYSL3	Body	opensea
cg20388732	0.12	0.0038	0.31	0.44	0.12	STAT5A	TSS200	shore
cg14280382	0.12	0.0019	0.72	0.85	0.12	NTN4	Body	opensea
cg25746092	0.12	0.0042	0.71	0.83	0.12	PSORS1C1	TSS1500	opensea
cg07804749	0.12	0.0080	0.61	0.73	0.12		IGR	opensea
cg15012161	0.12	0.0026	0.69	0.81	0.12		IGR	opensea
ch.8.18329843R	0.12	0.0009	0.14	0.26	0.12		IGR	opensea
cg18108683	0.12	0.0090	0.56	0.68	0.12	FBXL13	Body	opensea
cg24681963	0.12	0.0003	0.64	0.76	0.12		IGR	shore
cg19964491	0.12	0.0000	0.18	0.30	0.12	TNXB	Body	opensea
cg08924023	0.12	0.0009	0.58	0.71	0.12		IGR	opensea
cg23462772	0.12	0.0209	0.51	0.63	0.12		IGR	opensea
cg18429374	0.12	0.0001	0.82	0.95	0.12		IGR	opensea
cg19981243	0.12	0.0009	0.50	0.62	0.12	ZBTB20	TSS200	opensea
cg26575450	0.12	0.0295	0.56	0.68	0.12		IGR	shore
cg23281555	0.12	0.0059	0.49	0.61	0.12	TM9SF3	Body	opensea
cg02873991	0.12	0.0038	0.33	0.45	0.12	C12orf77	TSS1500	opensea
cg06871529	0.12	0.0054	0.59	0.71	0.12	FRY	Body	opensea
cg10423842	0.12	0.0128	0.57	0.70	0.12		IGR	opensea

cg18107827	0.12	0.0034	0.51	0.64	0.12	GRRP1	3'UTR	shore
cg20658466	0.12	0.0017	0.35	0.48	0.12		IGR	opensea
cg16018921	0.12	0.0003	0.72	0.85	0.12	PALLD	Body	opensea
cg19028997	0.12	0.0170	0.70	0.82	0.12	ELK4	TSS1500	shore
cg21617903	0.12	0.0012	0.73	0.85	0.12	VGLL4	3'UTR	opensea
cg00296578	0.12	0.0056	0.63	0.75	0.12	CRIM1	Body	opensea
cg00975971	0.12	0.0004	0.57	0.70	0.12	ANKH	Body	opensea
cg21368566	0.12	0.0035	0.68	0.80	0.12	AGAP1	Body	opensea
cg22699314	0.12	0.0010	0.62	0.74	0.12		IGR	opensea
cg14824921	0.12	0.0052	0.47	0.60	0.12	IGFBP7	Body	opensea
cg12279397	0.13	0.0008	0.57	0.69	0.13		IGR	opensea
cg01513078	0.13	0.0009	0.50	0.63	0.13	AHNAK2	Body	opensea
cg17185710	0.13	0.0009	0.73	0.86	0.13	MBNL1	TSS1500	shore
cg00464046	0.13	0.0032	0.56	0.69	0.13	C7orf49	Body	shore
cg06018119	0.13	0.0087	0.38	0.50	0.13	MAPK11	Body	shore
cg09923954	0.13	0.0357	0.35	0.47	0.13		IGR	opensea
cg08785317	0.13	0.0161	0.61	0.74	0.13		IGR	shelf
cg12792264	0.13	0.0003	0.74	0.86	0.13	GLT25D2	Body	opensea
cg11452329	0.13	0.0000	0.42	0.54	0.13		IGR	opensea
cg23722096	0.13	0.0016	0.58	0.70	0.13	SND1	Body	opensea
cg26245202	0.13	0.0055	0.71	0.83	0.13	ELK4	TSS1500	shore
cg02019444	0.13	0.0054	0.37	0.50	0.13	ITSN1	5'UTR	shore
cg25954269	0.13	0.0276	0.68	0.80	0.13		IGR	opensea
cg10489463	0.13	0.0109	0.67	0.80	0.13	LTBP1	Body	opensea
cg20765267	0.13	0.0004	0.11	0.23	0.13	DCAF13	Body	shore
cg26870745	0.13	0.0059	0.59	0.71	0.13		IGR	opensea
cg11916478	0.13	0.0103	0.55	0.68	0.13	IQSEC3	Body	island
cg25372085	0.13	0.0057	0.56	0.68	0.13		IGR	opensea
cg01715025	0.13	0.0007	0.78	0.90	0.13	RNF115	Body	opensea
cg27592331	0.13	0.0030	0.26	0.38	0.13	GATA6	Body	shore
cg18108818	0.13	0.0016	0.61	0.74	0.13	FBXL13	Body	opensea
cg02107844	0.13	0.0026	0.48	0.61	0.13	SLCO3A1	Body	opensea
cg04128967	0.13	0.0000	0.61	0.74	0.13		IGR	opensea
cg08133496	0.13	0.0000	0.31	0.44	0.13	EXT2	TSS200	shore
cg16046375	0.13	0.0418	0.42	0.54	0.13	RASA3	Body	shore
cg22760287	0.13	0.0086	0.65	0.77	0.13		IGR	opensea
cg08145590	0.13	0.0016	0.24	0.36	0.13	C3orf15	Body	island
cg06363692	0.13	0.0090	0.62	0.75	0.13	RNF121	Body	opensea
cg24611970	0.13	0.0007	0.58	0.71	0.13		IGR	opensea
cg22900476	0.13	0.0003	0.37	0.50	0.13	RAPGEF4	Body	opensea
cg02640147	0.13	0.0040	0.61	0.74	0.13	KCNIP1	Body	opensea
cg01796223	0.13	0.0100	0.58	0.71	0.13	CPA4	1stExon	opensea
cg12518146	0.13	0.0006	0.31	0.43	0.13	F2RL1	Body	island

cg19350115	0.13	0.0167	0.72	0.85	0.13		IGR	opensea
cg03128029	0.13	0.0007	0.38	0.50	0.13	NOP58	Body	opensea
cg15153141	0.13	0.0022	0.42	0.55	0.13	ATG10	Body	opensea
cg02225720	0.13	0.0420	0.68	0.81	0.13	ITGAE	Body	shelf
cg22319784	0.13	0.0055	0.31	0.44	0.13	BTNL9	3'UTR	island
cg10513595	0.13	0.0170	0.35	0.48	0.13		IGR	opensea
cg11493661	0.13	0.0000	0.23	0.35	0.13	TNXB	Body	opensea
cg17974515	0.13	0.0072	0.73	0.86	0.13		IGR	shore
cg23375169	0.13	0.0000	0.62	0.75	0.13		IGR	opensea
cg14082886	0.13	0.0431	0.73	0.86	0.13	CD44	Body	shelf
cg03628053	0.13	0.0058	0.65	0.77	0.13	FBN1	Body	shelf
cg15922174	0.13	0.0070	0.20	0.32	0.13	CRB2	Body	island
cg08705382	0.13	0.0013	0.68	0.81	0.13	GALNT2	Body	opensea
cg12229979	0.13	0.0001	0.48	0.61	0.13	MYO9B	5'UTR	shelf
cg23679344	0.13	0.0146	0.78	0.91	0.13	MED1	3'UTR	shore
cg14931884	0.13	0.0146	0.36	0.48	0.13	DIP2C	Body	shore
cg07740894	0.13	0.0084	0.68	0.81	0.13	CCNY	Body	opensea
ch.10.2760753F	0.13	0.0006	0.15	0.28	0.13		IGR	shore
cg18621852	0.13	0.0029	0.63	0.75	0.13	C3orf24	TSS200	island
cg20236089	0.13	0.0053	0.74	0.87	0.13	DTNBP1	Body	opensea
cg19591417	0.13	0.0003	0.56	0.69	0.13		IGR	opensea
cg20073153	0.13	0.0440	0.60	0.72	0.13		IGR	opensea
cg14215105	0.13	0.0013	0.62	0.75	0.13	INTS3	Body	opensea
cg10011495	0.13	0.0006	0.65	0.78	0.13		IGR	shore
cg06160973	0.13	0.0004	0.24	0.37	0.13	LRP1	1stExon	shore
cg16231917	0.13	0.0047	0.35	0.48	0.13	PVT1	Body	opensea
cg08348121	0.13	0.0121	0.63	0.75	0.13	SKIL	TSS1500	shore
cg26829395	0.13	0.0092	0.66	0.79	0.13		IGR	opensea
cg13573928	0.13	0.0001	0.13	0.26	0.13	PALLD	5'UTR	island
cg19835796	0.13	0.0001	0.58	0.71	0.13		IGR	opensea
cg19277884	0.13	0.0000	0.67	0.80	0.13	SPATA2	5'UTR	shore
cg13531667	0.13	0.0333	0.54	0.66	0.13	MCC	TSS1500	shore
cg17097293	0.13	0.0000	0.66	0.78	0.13	PIP5K1C	Body	shelf
cg27239981	0.13	0.0000	0.38	0.51	0.13		IGR	shelf
cg18363918	0.13	0.0029	0.26	0.39	0.13	IGLON5	Body	shore
cg01610979	0.13	0.0188	0.28	0.41	0.13		IGR	opensea
cg00258316	0.13	0.0286	0.60	0.73	0.13		IGR	opensea
cg18680181	0.13	0.0064	0.49	0.61	0.13	KIAA0391	Body	opensea
cg03887721	0.13	0.0123	0.69	0.82	0.13		IGR	opensea
cg10736330	0.13	0.0001	0.70	0.83	0.13	GPR183	TSS1500	opensea
cg08305942	0.13	0.0257	0.75	0.88	0.13		IGR	opensea
cg23138413	0.13	0.0040	0.79	0.92	0.13		IGR	opensea
cg13051700	0.13	0.0002	0.50	0.63	0.13	MAGI2	Body	opensea

cg06686742	0.13	0.0004	0.29	0.42	0.13	LASS4	TSS1500	shore
cg14571813	0.13	0.0063	0.35	0.48	0.13		IGR	island
cg06799735	0.13	0.0005	0.57	0.70	0.13	AHNAK2	Body	opensea
cg01063579	0.13	0.0123	0.63	0.76	0.13		IGR	island
cg10728351	0.13	0.0048	0.74	0.87	0.13		IGR	opensea
cg06132400	0.13	0.0124	0.34	0.47	0.13		IGR	island
cg07990843	0.13	0.0025	0.21	0.34	0.13		IGR	shore
cg12591668	0.13	0.0385	0.77	0.90	0.13		IGR	opensea
cg16476991	0.13	0.0085	0.49	0.62	0.13	RASA3	Body	opensea
cg23186333	0.13	0.0083	0.76	0.89	0.13	CD44	Body	shore
cg01513157	0.13	0.0239	0.34	0.47	0.13	SDR42E1	3'UTR	opensea
cg04156483	0.13	0.0037	0.61	0.74	0.13	MORN1	Body	island
cg03156546	0.13	0.0040	0.52	0.65	0.13	TNRC6A	Body	opensea
cg06189394	0.13	0.0020	0.49	0.62	0.13	LRIG1	Body	opensea
cg14713217	0.13	0.0000	0.18	0.31	0.13		IGR	opensea
cg02899346	0.13	0.0140	0.14	0.27	0.13	RTN2	Body	shore
cg01415937	0.13	0.0102	0.32	0.45	0.13	UBE4B	Body	opensea
cg24990131	0.13	0.0018	0.29	0.41	0.13	F13A1	5'UTR	opensea
cg01466164	0.13	0.0005	0.44	0.57	0.13		IGR	shore
cg10328047	0.13	0.0004	0.67	0.80	0.13	PLEC1	Body	island
cg01700683	0.13	0.0017	0.33	0.46	0.13	RAB8B	Body	opensea
cg14157435	0.13	0.0047	0.28	0.41	0.13	NRP2	Body	opensea
cg19090437	0.13	0.0326	0.66	0.79	0.13	TBC1D1	Body	opensea
cg14292522	0.13	0.0091	0.52	0.65	0.13	UHRF1BP1L	Body	shore
cg13414270	0.13	0.0005	0.41	0.54	0.13		IGR	opensea
cg19405883	0.13	0.0128	0.45	0.58	0.13	RAI14	TSS200	opensea
cg06385583	0.13	0.0000	0.11	0.24	0.13		IGR	opensea
cg06427571	0.13	0.0007	0.59	0.72	0.13		IGR	opensea
cg10577819	0.13	0.0009	0.80	0.93	0.13		IGR	opensea
cg21137244	0.13	0.0027	0.68	0.81	0.13		IGR	shelf
cg22871253	0.13	0.0047	0.32	0.45	0.13	EZR	Body	shore
cg11084266	0.13	0.0000	0.32	0.45	0.13	PMF1	Body	shore
cg20703997	0.13	0.0209	0.28	0.41	0.13		IGR	opensea
cg05622577	0.13	0.0068	0.64	0.77	0.13		IGR	opensea
cg05499012	0.13	0.0007	0.68	0.81	0.13		IGR	opensea
cg13173567	0.13	0.0010	0.24	0.37	0.13	DENND5A	Body	opensea
cg04515200	0.13	0.0162	0.30	0.43	0.13		IGR	shore
cg01931028	0.13	0.0013	0.51	0.64	0.13	MAEA	Body	shelf
cg18442362	0.13	0.0007	0.43	0.56	0.13	OGDH	Body	opensea
cg24886867	0.13	0.0080	0.66	0.80	0.13	RIPK1	TSS1500	opensea
cg19774788	0.13	0.0001	0.59	0.72	0.13	WDR51A	Body	opensea
cg23654401	0.13	0.0158	0.70	0.83	0.13	VOPP1	Body	opensea
cg10167233	0.13	0.0082	0.66	0.80	0.13	OGFRL1	Body	opensea

cg08889114	0.13	0.0000	0.79	0.92	0.13		IGR	opensea
cg11100795	0.13	0.0035	0.47	0.60	0.13	AP3B1	Body	opensea
cg23662097	0.13	0.0111	0.41	0.54	0.13	ITPR1	Body	opensea
cg01280881	0.13	0.0036	0.24	0.37	0.13		IGR	opensea
cg27638217	0.13	0.0143	0.39	0.52	0.13		IGR	shore
cg05963604	0.13	0.0295	0.36	0.49	0.13	APPL1	Body	shore
cg19521832	0.13	0.0026	0.15	0.28	0.13	KCNE1	TSS200	island
cg26177311	0.13	0.0025	0.71	0.84	0.13	PEMT	Body	opensea
cg02578087	0.13	0.0288	0.74	0.88	0.13	C3orf32	Body	opensea
cg16643088	0.13	0.0166	0.20	0.34	0.13	CSRNP1	5'UTR	shelf
cg06933370	0.13	0.0001	0.14	0.27	0.13	MEIS2	Body	island
cg21104965	0.13	0.0000	0.34	0.47	0.13	DCBLD1	Body	island
cg21829923	0.13	0.0003	0.25	0.38	0.13	SP5	Body	island
cg02564299	0.13	0.0000	0.61	0.74	0.13	ZBTB20	5'UTR	opensea
cg05348875	0.13	0.0008	0.47	0.60	0.13	NRP2	Body	opensea
cg25335841	0.13	0.0014	0.22	0.36	0.13		IGR	island
cg01224520	0.13	0.0188	0.51	0.65	0.13		IGR	opensea
cg24667756	0.13	0.0002	0.54	0.67	0.13	RPTOR	Body	shelf
cg19145082	0.13	0.0284	0.52	0.65	0.13		IGR	opensea
cg14157107	0.13	0.0011	0.55	0.68	0.13	BNC2	Body	opensea
cg23654821	0.13	0.0173	0.23	0.37	0.13	CSRNP1	5'UTR	shore
cg13941978	0.13	0.0046	0.50	0.64	0.13	PLEKHO2	Body	opensea
cg04279411	0.13	0.0166	0.42	0.55	0.13		IGR	opensea
cg13544946	0.13	0.0051	0.37	0.50	0.13	TLN1	5'UTR	shelf
cg13805761	0.13	0.0017	0.49	0.63	0.13	DCLK1	Body	opensea
cg07388969	0.13	0.0007	0.55	0.69	0.13		IGR	opensea
cg05228359	0.13	0.0135	0.33	0.46	0.13		IGR	opensea
cg06327814	0.13	0.0076	0.64	0.78	0.13	C7orf53	5'UTR	opensea
cg24820672	0.13	0.0081	0.61	0.75	0.13		IGR	shore
cg19418458	0.13	0.0317	0.44	0.57	0.13		IGR	island
cg13221458	0.13	0.0000	0.15	0.29	0.13	SOD2	Body	shore
cg20979153	0.13	0.0144	0.37	0.50	0.13	ZNF217	TSS200	shore
cg04899656	0.13	0.0033	0.68	0.81	0.13		IGR	opensea
cg20960181	0.13	0.0089	0.47	0.61	0.13		IGR	opensea
cg25731261	0.13	0.0015	0.53	0.67	0.13	BBC3	1stExon	shore
cg14640149	0.13	0.0175	0.65	0.78	0.13		IGR	opensea
cg04645534	0.13	0.0001	0.29	0.43	0.13	STC1	TSS1500	opensea
cg12868173	0.13	0.0037	0.19	0.32	0.13		IGR	island
cg15117754	0.13	0.0067	0.53	0.66	0.13	C3orf24	TSS200	shore
cg23252259	0.14	0.0145	0.46	0.60	0.14		IGR	shore
cg03354616	0.14	0.0042	0.51	0.65	0.14	BCKDHB	Body	opensea
cg22110158	0.14	0.0022	0.52	0.65	0.14	ST14	Body	opensea
cg18087256	0.14	0.0001	0.70	0.83	0.14		IGR	opensea

cg05567440	0.14	0.0012	0.61	0.75	0.14		IGR	opensea
cg21394171	0.14	0.0011	0.32	0.46	0.14		IGR	opensea
cg22400059	0.14	0.0101	0.69	0.82	0.14	PEX14	Body	opensea
cg25538571	0.14	0.0110	0.60	0.73	0.14		IGR	opensea
cg22244986	0.14	0.0003	0.61	0.75	0.14		IGR	opensea
cg25004840	0.14	0.0021	0.28	0.41	0.14		IGR	opensea
cg26877720	0.14	0.0148	0.64	0.77	0.14	FAM107B	Body	shore
cg22550815	0.14	0.0135	0.61	0.75	0.14	KANK2	3'UTR	opensea
cg15032314	0.14	0.0014	0.19	0.32	0.14	PRR12	Body	island
cg01589587	0.14	0.0038	0.30	0.43	0.14	BATF	Body	opensea
cg16085649	0.14	0.0024	0.28	0.41	0.14	AKAP13	Body	opensea
cg03097134	0.14	0.0064	0.55	0.68	0.14		IGR	opensea
cg10840277	0.14	0.0172	0.57	0.70	0.14		IGR	opensea
cg19089701	0.14	0.0116	0.45	0.59	0.14		IGR	opensea
cg01357892	0.14	0.0020	0.27	0.41	0.14	ZXDC	Body	shelf
cg18842353	0.14	0.0000	0.15	0.29	0.14		IGR	opensea
cg15645888	0.14	0.0001	0.61	0.75	0.14	FBXO16	3'UTR	opensea
cg07181702	0.14	0.0018	0.40	0.53	0.14	MIR21	Body	opensea
cg10205038	0.14	0.0139	0.57	0.71	0.14	RIN3	Body	opensea
cg16638092	0.14	0.0064	0.67	0.80	0.14	RPTOR	Body	shore
cg23400446	0.14	0.0241	0.08	0.22	0.14	CYP2E1	Body	island
cg06633978	0.14	0.0130	0.55	0.69	0.14	CFDP1	Body	opensea
cg13780718	0.14	0.0015	0.45	0.59	0.14	ZSWIM1	3'UTR	opensea
cg06680852	0.14	0.0064	0.51	0.64	0.14		IGR	shelf
cg21664909	0.14	0.0002	0.54	0.68	0.14	PXDN	Body	opensea
cg13153708	0.14	0.0007	0.56	0.70	0.14		IGR	opensea
cg06342072	0.14	0.0022	0.47	0.61	0.14		IGR	opensea
cg09728393	0.14	0.0020	0.63	0.77	0.14	KIF13A	Body	opensea
cg03624316	0.14	0.0044	0.38	0.52	0.14	PFDN5	Body	shore
cg08382534	0.14	0.0017	0.76	0.90	0.14	SLC38A8	Body	opensea
cg10947408	0.14	0.0005	0.19	0.33	0.14		IGR	island
cg27565517	0.14	0.0000	0.22	0.36	0.14	EDN1	Body	opensea
cg26314722	0.14	0.0167	0.65	0.79	0.14		IGR	opensea
cg18117669	0.14	0.0066	0.65	0.79	0.14	TCF12	TSS1500	opensea
cg06881965	0.14	0.0029	0.37	0.51	0.14	DCAF5	Body	opensea
cg19319037	0.14	0.0032	0.72	0.86	0.14	TTF2	Body	shore
cg01005968	0.14	0.0038	0.21	0.35	0.14	RTN2	5'UTR	shore
cg08651590	0.14	0.0006	0.64	0.78	0.14	SPRED2	Body	shelf
cg20221591	0.14	0.0004	0.36	0.50	0.14	MYEOV2	TSS1500	shore
cg10574006	0.14	0.0000	0.58	0.72	0.14		IGR	opensea
cg19734190	0.14	0.0018	0.62	0.76	0.14	TBC1D14	Body	opensea
cg23202253	0.14	0.0100	0.75	0.89	0.14	ITPR2	Body	opensea
cg12939390	0.14	0.0007	0.61	0.75	0.14	SRBD1	Body	opensea

cg24671734	0.14	0.0240	0.43	0.56	0.14	BTBD11	Body	opensea
cg20493497	0.14	0.0061	0.28	0.42	0.14		IGR	opensea
cg15985132	0.14	0.0033	0.73	0.87	0.14		IGR	opensea
cg01232511	0.14	0.0026	0.55	0.69	0.14	PRSS21	Body	island
cg04452713	0.14	0.0005	0.15	0.29	0.14	DST	Body	shore
cg24028809	0.14	0.0168	0.54	0.68	0.14	RGS17	5'UTR	opensea
cg03052078	0.14	0.0001	0.51	0.65	0.14	STXBP5	Body	shore
cg15324723	0.14	0.0010	0.63	0.77	0.14	VPS13B	Body	opensea
cg11811828	0.14	0.0267	0.52	0.66	0.14		IGR	shore
cg10732611	0.14	0.0002	0.17	0.31	0.14	STARD13	Body	opensea
cg01199603	0.14	0.0003	0.75	0.89	0.14	MSRB3	Body	opensea
cg11970797	0.14	0.0007	0.75	0.89	0.14	CRYL1	Body	opensea
cg23683800	0.14	0.0000	0.12	0.26	0.14		IGR	opensea
cg19784903	0.14	0.0049	0.58	0.72	0.14	TBKBP1	Body	island
cg00166722	0.14	0.0006	0.73	0.87	0.14	C3orf24	TSS200	island
cg05876246	0.14	0.0092	0.28	0.42	0.14		IGR	opensea
cg13636014	0.14	0.0001	0.40	0.54	0.14		IGR	opensea
cg27023597	0.14	0.0020	0.30	0.44	0.14	MIR21	TSS1500	opensea
cg07493197	0.14	0.0034	0.38	0.52	0.14		IGR	opensea
cg05543593	0.14	0.0001	0.53	0.67	0.14		IGR	opensea
cg07506560	0.14	0.0043	0.59	0.74	0.14		IGR	opensea
cg06405186	0.14	0.0091	0.39	0.53	0.14	SYT7	3'UTR	island
cg08575883	0.14	0.0027	0.68	0.82	0.14		IGR	opensea
cg10574566	0.14	0.0003	0.19	0.33	0.14		IGR	opensea
cg14189116	0.14	0.0240	0.77	0.91	0.14	CAPRIN2	Body	opensea
cg04637264	0.14	0.0034	0.48	0.62	0.14		IGR	opensea
cg02449762	0.14	0.0037	0.63	0.77	0.14		IGR	opensea
cg12804755	0.14	0.0003	0.50	0.64	0.14		IGR	opensea
cg13332142	0.14	0.0000	0.67	0.81	0.14		IGR	opensea
cg12559197	0.14	0.0005	0.40	0.54	0.14	PDE8B	Body	opensea
cg23530586	0.14	0.0035	0.39	0.54	0.14		IGR	opensea
cg23261319	0.14	0.0096	0.61	0.75	0.14		IGR	opensea
cg19321696	0.14	0.0000	0.52	0.66	0.14	NPS	3'UTR	opensea
cg26400954	0.14	0.0010	0.64	0.78	0.14	LMO7	Body	opensea
cg27049539	0.14	0.0008	0.56	0.70	0.14	USP40	Body	opensea
cg02714666	0.14	0.0047	0.50	0.64	0.14		IGR	opensea
cg04164584	0.14	0.0024	0.62	0.76	0.14	PHF12	Body	opensea
cg20740711	0.14	0.0070	0.38	0.52	0.14		IGR	opensea
cg11802797	0.14	0.0000	0.14	0.28	0.14	KIF1B	3'UTR	opensea
cg19628988	0.14	0.0006	0.29	0.43	0.14	CXXC5	5'UTR	island
cg13047869	0.14	0.0015	0.74	0.88	0.14	C3orf24	1stExon	island
cg01119374	0.14	0.0002	0.66	0.80	0.14		IGR	shore
cg10438089	0.14	0.0022	0.21	0.35	0.14	FAM117A	Body	opensea

cg18700744	0.14	0.0044	0.25	0.40	0.14	NAA25	Body	opensea
cg01882871	0.14	0.0000	0.51	0.66	0.14		IGR	opensea
cg19017553	0.14	0.0019	0.65	0.79	0.14	PARD3	Body	opensea
cg11960655	0.14	0.0061	0.50	0.64	0.14		IGR	opensea
cg01354782	0.14	0.0014	0.64	0.78	0.14	ITPR2	Body	opensea
cg04944393	0.14	0.0001	0.78	0.92	0.14	ATP6V1E2	TSS1500	opensea
cg14420953	0.14	0.0011	0.66	0.80	0.14		IGR	shelf
ch.3.3371471R	0.14	0.0032	0.18	0.32	0.14	NLGN1	5'UTR	opensea
cg23543318	0.14	0.0066	0.54	0.68	0.14	LOC100130872	Body	opensea
cg02952295	0.14	0.0059	0.63	0.78	0.14	TRPM8	Body	opensea
cg03327325	0.14	0.0052	0.26	0.40	0.14		IGR	island
cg15244778	0.14	0.0009	0.73	0.87	0.14		IGR	opensea
cg11235594	0.14	0.0007	0.52	0.66	0.14	NPHP1	Body	opensea
cg04254487	0.14	0.0082	0.71	0.86	0.14	TBPL1	Body	opensea
cg09467607	0.14	0.0394	0.43	0.57	0.14	FEZ2	TSS1500	shore
cg14977018	0.14	0.0001	0.74	0.89	0.14	TMEM44	Body	opensea
cg24680632	0.14	0.0012	0.37	0.52	0.14		IGR	opensea
cg01361499	0.14	0.0192	0.69	0.83	0.14		IGR	shelf
cg25913882	0.14	0.0070	0.61	0.76	0.14	CUBN	Body	opensea
cg27246744	0.14	0.0016	0.27	0.41	0.14	OSBPL10	Body	opensea
cg26856257	0.14	0.0051	0.54	0.69	0.14		IGR	shelf
cg19714737	0.14	0.0055	0.52	0.66	0.14		IGR	shelf
cg23733260	0.14	0.0063	0.65	0.79	0.14	BBX	5'UTR	opensea
cg02033582	0.14	0.0187	0.51	0.65	0.14		IGR	opensea
cg01862311	0.14	0.0014	0.60	0.75	0.14		IGR	shore
cg26300461	0.15	0.0010	0.46	0.61	0.15		IGR	shore
cg16210979	0.15	0.0007	0.50	0.64	0.15	YTHDC2	Body	opensea
cg08607018	0.15	0.0014	0.26	0.41	0.15		IGR	island
ch.2.11889418R	0.15	0.0001	0.39	0.54	0.15		IGR	opensea
cg04057161	0.15	0.0013	0.66	0.81	0.15		IGR	opensea
cg01412419	0.15	0.0089	0.61	0.76	0.15		IGR	opensea
cg17066594	0.15	0.0075	0.21	0.35	0.15	UNC93B1	Body	shore
cg26541218	0.15	0.0177	0.63	0.78	0.15	PKD1L1	Body	opensea
cg21814550	0.15	0.0137	0.45	0.60	0.15		IGR	shelf
cg23199907	0.15	0.0009	0.63	0.78	0.15	PDS5B	Body	opensea
cg01031032	0.15	0.0047	0.23	0.38	0.15	CD200	TSS200	island
cg17686260	0.15	0.0284	0.47	0.62	0.15	MGMT	Body	opensea
cg01238435	0.15	0.0047	0.42	0.57	0.15		IGR	opensea
cg17660833	0.15	0.0126	0.49	0.64	0.15	HRH1	5'UTR	opensea
cg05695699	0.15	0.0004	0.65	0.80	0.15	KIAA1522	Body	shore
cg11645556	0.15	0.0000	0.21	0.35	0.15	RRAS2	Body	opensea
cg09803959	0.15	0.0221	0.28	0.42	0.15	RPTOR	Body	shore
cg02524983	0.15	0.0012	0.33	0.48	0.15	LPP	5'UTR	opensea

cg19229692	0.15	0.0006	0.74	0.89	0.15	PLXNA4	Body	opensea
cg14115597	0.15	0.0001	0.27	0.42	0.15	SSBP2	Body	opensea
cg25173405	0.15	0.0039	0.11	0.25	0.15	C17orf57	5'UTR	shore
cg14737484	0.15	0.0002	0.48	0.63	0.15	TNXB	Body	shore
cg17349632	0.15	0.0013	0.70	0.85	0.15	PLXNA4	Body	opensea
cg16783186	0.15	0.0007	0.56	0.71	0.15		IGR	opensea
cg10764891	0.15	0.0017	0.68	0.83	0.15		IGR	shelf
cg06474225	0.15	0.0000	0.23	0.38	0.15	HTRA1	Body	opensea
cg23197236	0.15	0.0000	0.72	0.87	0.15	LIN7C	3'UTR	opensea
cg21796547	0.15	0.0072	0.26	0.41	0.15	PAPLN	5'UTR	shore
cg06595154	0.15	0.0001	0.49	0.64	0.15	MRVI1	TSS1500	opensea
cg03577157	0.15	0.0283	0.69	0.84	0.15	RNU5E	Body	opensea
cg16377679	0.15	0.0000	0.42	0.57	0.15	PDE4D	Body	opensea
cg26094842	0.15	0.0065	0.72	0.87	0.15	VTI1A	Body	opensea
cg15391590	0.15	0.0434	0.72	0.87	0.15	ICK	5'UTR	opensea
cg07964219	0.15	0.0015	0.37	0.52	0.15	COL18A1	Body	shore
cg17552333	0.15	0.0016	0.26	0.41	0.15		IGR	opensea
cg07008591	0.15	0.0069	0.67	0.82	0.15	TEAD1	Body	opensea
cg21708130	0.15	0.0107	0.29	0.44	0.15	LRRFIP1	Body	shelf
cg15633390	0.15	0.0042	0.39	0.54	0.15	EIF4E	5'UTR	shore
cg25123566	0.15	0.0080	0.69	0.84	0.15	FAM113B	5'UTR	opensea
cg20848291	0.15	0.0017	0.24	0.39	0.15	ZAN	Body	opensea
cg12427162	0.15	0.0000	0.57	0.72	0.15	SFT2D2	Body	shelf
cg18992848	0.15	0.0007	0.27	0.42	0.15	TPK1	Body	shore
cg06853894	0.15	0.0157	0.59	0.74	0.15	TNFRSF8	3'UTR	opensea
cg01649611	0.15	0.0201	0.55	0.71	0.15	THADA	Body	opensea
cg14753094	0.15	0.0017	0.69	0.84	0.15	HSD17B12	Body	opensea
cg25467833	0.15	0.0004	0.54	0.69	0.15		IGR	opensea
cg08928408	0.15	0.0048	0.69	0.84	0.15		IGR	opensea
cg05349016	0.15	0.0017	0.38	0.53	0.15	NMT1	Body	opensea
cg02832512	0.15	0.0057	0.60	0.76	0.15	FLJ22536	Body	opensea
cg08913523	0.15	0.0005	0.45	0.60	0.15		IGR	opensea
cg05762671	0.15	0.0055	0.58	0.74	0.15	KCTD19	Body	opensea
cg13052638	0.15	0.0059	0.30	0.46	0.15		IGR	shelf
cg23295647	0.15	0.0207	0.40	0.56	0.15	NPAS3	Body	island
cg04642300	0.15	0.0009	0.43	0.58	0.15	ARMC2	Body	opensea
cg13327911	0.15	0.0266	0.67	0.82	0.15	COL21A1	Body	opensea
cg26197915	0.15	0.0005	0.20	0.35	0.15	PTPRJ	Body	opensea
cg03354554	0.15	0.0067	0.29	0.44	0.15		IGR	shore
cg02573091	0.15	0.0096	0.26	0.42	0.15		IGR	shore
cg21369466	0.15	0.0073	0.65	0.81	0.15	LANCL2	Body	shelf
cg06570967	0.15	0.0034	0.76	0.91	0.15		IGR	opensea
cg06161600	0.15	0.0036	0.32	0.48	0.15	BAIAP3	Body	island

cg26177041	0.15	0.0000	0.20	0.35	0.15	CAMK2D	Body	opensea
cg22473770	0.15	0.0063	0.53	0.68	0.15	EVI2A	5'UTR	opensea
cg13419330	0.15	0.0019	0.56	0.71	0.15	IRAK2	Body	opensea
cg07664000	0.16	0.0053	0.53	0.69	0.16	TMIGD1	5'UTR	opensea
cg09228833	0.16	0.0201	0.48	0.64	0.16	ZNF217	TSS200	shore
cg00993830	0.16	0.0010	0.38	0.53	0.16	UBE2H	Body	opensea
cg25298319	0.16	0.0048	0.23	0.39	0.16		IGR	island
cg01993169	0.16	0.0006	0.73	0.89	0.16		IGR	opensea
cg08939850	0.16	0.0054	0.59	0.74	0.16	RPTOR	Body	shore
cg06478886	0.16	0.0324	0.32	0.48	0.16		IGR	shore
cg20968743	0.16	0.0079	0.31	0.46	0.16	TSPAN18	5'UTR	opensea
cg00223245	0.16	0.0014	0.41	0.57	0.16		IGR	opensea
cg25616869	0.16	0.0017	0.54	0.70	0.16		IGR	opensea
cg06691616	0.16	0.0006	0.51	0.67	0.16		IGR	opensea
cg18156592	0.16	0.0018	0.27	0.43	0.16	ARL6IP5	Body	opensea
cg13017929	0.16	0.0039	0.64	0.80	0.16		IGR	opensea
cg18456803	0.16	0.0004	0.40	0.56	0.16	ELF1	TSS200	opensea
cg00377497	0.16	0.0001	0.55	0.71	0.16	TRIM35	Body	shore
cg01116477	0.16	0.0000	0.59	0.75	0.16		IGR	opensea
cg05227773	0.16	0.0003	0.62	0.78	0.16	ZFHX3	3'UTR	shelf
cg24736734	0.16	0.0005	0.26	0.42	0.16		IGR	opensea
cg10732871	0.16	0.0016	0.29	0.45	0.16	GPX4	TSS1500	shore
cg03987648	0.16	0.0046	0.66	0.82	0.16	MAML3	Body	opensea
cg07285237	0.16	0.0178	0.69	0.85	0.16		IGR	opensea
cg10110335	0.16	0.0050	0.53	0.69	0.16	SYN2	Body	shelf
cg10557907	0.16	0.0013	0.43	0.59	0.16	PRDM16	Body	opensea
cg27310092	0.16	0.0042	0.30	0.46	0.16		IGR	opensea
cg07813142	0.16	0.0022	0.10	0.26	0.16	SP5	Body	island
cg14701867	0.16	0.0445	0.45	0.61	0.16	ZNF365	Body	opensea
cg13001142	0.16	0.0000	0.62	0.78	0.16	STXBP5	Body	shelf
cg09450153	0.16	0.0428	0.56	0.72	0.16	CREB5	Body	opensea
cg00754989	0.16	0.0281	0.53	0.69	0.16		IGR	opensea
cg24508426	0.16	0.0016	0.23	0.39	0.16		IGR	island
cg15852787	0.16	0.0000	0.68	0.84	0.16	FRMD6	5'UTR	opensea
cg01409343	0.16	0.0008	0.38	0.54	0.16	TMEM49	Body	opensea
cg05373263	0.16	0.0251	0.56	0.72	0.16		IGR	shore
cg26284735	0.16	0.0006	0.46	0.63	0.16		IGR	shelf
cg01412970	0.16	0.0335	0.28	0.44	0.16	PLD6	1stExon	island
cg06769820	0.16	0.0212	0.40	0.56	0.16		IGR	opensea
cg13315147	0.16	0.0343	0.13	0.30	0.16	CYP2E1	Body	island
cg02351277	0.16	0.0031	0.27	0.43	0.16		IGR	opensea
cg24314564	0.16	0.0312	0.56	0.72	0.16		IGR	shelf
cg09435170	0.16	0.0000	0.14	0.30	0.16		IGR	opensea

cg24475182	0.16	0.0007	0.22	0.38	0.16		IGR	opensea
cg20899781	0.16	0.0000	0.35	0.51	0.16		IGR	opensea
cg10763234	0.16	0.0011	0.22	0.38	0.16		IGR	island
cg02849956	0.16	0.0019	0.56	0.72	0.16		IGR	shore
cg11029367	0.16	0.0016	0.64	0.80	0.16	HEG1	Body	opensea
cg16429725	0.16	0.0001	0.52	0.68	0.16	KIFC3	Body	shelf
cg04760448	0.16	0.0046	0.49	0.65	0.16	COL18A1	Body	island
cg15418499	0.16	0.0007	0.41	0.57	0.16	IL18	5'UTR	opensea
cg23920246	0.16	0.0011	0.73	0.89	0.16		IGR	opensea
cg17611046	0.16	0.0008	0.61	0.77	0.16	FARS2	Body	opensea
cg16337566	0.16	0.0001	0.25	0.41	0.16	PINX1	Body	opensea
cg26361533	0.16	0.0000	0.15	0.31	0.16	CACNA1C	Body	opensea
cg21818891	0.17	0.0003	0.76	0.93	0.17	SLC1A2	Body	opensea
cg00960147	0.17	0.0011	0.67	0.84	0.17		IGR	opensea
cg01195564	0.17	0.0028	0.58	0.74	0.17		IGR	opensea
cg16911981	0.17	0.0005	0.21	0.38	0.17	AUTS2	Body	island
cg05875421	0.17	0.0015	0.69	0.86	0.17	GPR68	5'UTR	opensea
cg04926881	0.17	0.0000	0.19	0.36	0.17		IGR	opensea
cg14276379	0.17	0.0037	0.25	0.42	0.17	C9orf3	Body	opensea
cg05492387	0.17	0.0007	0.57	0.73	0.17	RAP1GDS1	Body	opensea
cg12024811	0.17	0.0000	0.60	0.77	0.17	KALRN	Body	opensea
cg09548403	0.17	0.0049	0.28	0.45	0.17		IGR	opensea
cg22274117	0.17	0.0279	0.41	0.58	0.17	ATXN1	5'UTR	opensea
cg00858840	0.17	0.0002	0.18	0.35	0.17	SP5	Body	island
cg26203572	0.17	0.0001	0.64	0.81	0.17		IGR	opensea
cg09966895	0.17	0.0092	0.66	0.83	0.17	ODZ4	Body	opensea
cg24178897	0.17	0.0024	0.57	0.74	0.17		IGR	opensea
cg20651995	0.17	0.0012	0.53	0.70	0.17		IGR	opensea
cg11562411	0.17	0.0001	0.24	0.41	0.17	SCUBE3	Body	opensea
cg00601450	0.17	0.0051	0.44	0.61	0.17		IGR	shore
cg26504263	0.17	0.0000	0.59	0.76	0.17	ANKRD6	5'UTR	opensea
cg24113973	0.17	0.0086	0.20	0.37	0.17		IGR	shelf
cg17800426	0.17	0.0000	0.66	0.83	0.17	MYOZ3	TSS1500	shelf
cg18263166	0.17	0.0104	0.57	0.74	0.17		IGR	opensea
cg11868461	0.17	0.0055	0.36	0.54	0.17		IGR	opensea
cg26893861	0.17	0.0337	0.21	0.39	0.17	DUSP3	3'UTR	opensea
cg06457736	0.17	0.0031	0.49	0.66	0.17	HRH1	TSS200	opensea
cg18700940	0.17	0.0000	0.12	0.30	0.17	MAP3K14	5'UTR	opensea
cg21335012	0.17	0.0000	0.69	0.87	0.17		IGR	opensea
cg16683060	0.18	0.0192	0.50	0.68	0.18	UBAC2	Body	opensea
cg14708411	0.18	0.0339	0.72	0.89	0.18	SLC12A7	Body	island
cg23821329	0.18	0.0069	0.44	0.62	0.18	VIM	TSS1500	shore
cg24772753	0.18	0.0008	0.17	0.35	0.18	SP5	Body	island

cg00773142	0.18	0.0036	0.38	0.55	0.18	PLCG2	Body	opensea
cg07805542	0.18	0.0015	0.48	0.66	0.18	PIK3CD	Body	shelf
cg02992067	0.18	0.0000	0.68	0.86	0.18	FTO	Body	opensea
cg24062389	0.18	0.0001	0.61	0.79	0.18	BIVM	Body	opensea
cg25773259	0.18	0.0002	0.61	0.79	0.18		IGR	opensea
cg11118962	0.18	0.0008	0.44	0.62	0.18		IGR	opensea
cg08426157	0.18	0.0004	0.64	0.82	0.18	HDAC9	Body	opensea
cg21341586	0.18	0.0027	0.44	0.62	0.18	EIF4E	5'UTR	shore
cg16002660	0.18	0.0001	0.42	0.60	0.18	LOC284009	Body	opensea
cg07986257	0.18	0.0000	0.63	0.81	0.18		IGR	opensea
cg21860675	0.18	0.0011	0.66	0.84	0.18	FOXP1	5'UTR	opensea
cg27640794	0.18	0.0102	0.43	0.61	0.18	PALLD	5'UTR	shelf
cg07677157	0.18	0.0328	0.62	0.80	0.18		IGR	opensea
cg12686055	0.18	0.0000	0.21	0.39	0.18	ANO6	Body	opensea
cg02500300	0.18	0.0001	0.17	0.35	0.18	STOX2	1stExon	island
cg10316899	0.18	0.0001	0.35	0.53	0.18	MACF1	Body	opensea
cg27549186	0.18	0.0015	0.62	0.81	0.18	TIMP2	Body	opensea
cg24693760	0.18	0.0309	0.35	0.53	0.18		IGR	opensea
cg03548415	0.18	0.0033	0.36	0.54	0.18		IGR	opensea
cg03839782	0.18	0.0004	0.62	0.80	0.18	FAM65B	5'UTR	opensea
cg10862468	0.18	0.0238	0.18	0.37	0.18	CYP2E1	Body	island
cg16336556	0.18	0.0034	0.55	0.73	0.18	LTBP1	Body	opensea
cg17178175	0.19	0.0026	0.34	0.52	0.19	NFE2L2	Body	opensea
cg21727223	0.19	0.0000	0.30	0.49	0.19		IGR	opensea
cg06060522	0.19	0.0029	0.40	0.59	0.19		IGR	island
cg01413054	0.19	0.0314	0.56	0.74	0.19	CAB39	Body	opensea
cg01923775	0.19	0.0001	0.40	0.59	0.19	PALLD	Body	opensea
cg02515217	0.19	0.0001	0.42	0.61	0.19	MIR21	TSS200	opensea
cg19770281	0.19	0.0094	0.59	0.78	0.19		IGR	opensea
cg11809668	0.19	0.0001	0.62	0.81	0.19	KIAA0922	Body	opensea
cg07093324	0.19	0.0134	0.63	0.82	0.19	ACTR3	Body	shelf
cg11445109	0.19	0.0184	0.10	0.29	0.19	CYP2E1	Body	shore
cg25015038	0.19	0.0033	0.38	0.57	0.19		IGR	opensea
cg11608150	0.19	0.0233	0.25	0.45	0.19		IGR	shore
cg12609785	0.19	0.0015	0.40	0.59	0.19		IGR	shore
cg23099839	0.20	0.0032	0.23	0.43	0.20		IGR	opensea
cg14986890	0.20	0.0015	0.49	0.69	0.20	RARRES1	Body	opensea
cg27149179	0.20	0.0000	0.34	0.53	0.20		IGR	opensea
cg21144063	0.20	0.0007	0.29	0.49	0.20		IGR	opensea
cg11906781	0.20	0.0022	0.31	0.51	0.20	BRE	Body	opensea
cg23201812	0.20	0.0012	0.30	0.50	0.20		IGR	opensea
cg16233797	0.20	0.0013	0.61	0.82	0.20		IGR	opensea
cg24367957	0.21	0.0066	0.33	0.54	0.21		IGR	opensea

cg20905796	0.21	0.0100	0.47	0.68	0.21		IGR	opensea
cg15694704	0.21	0.0003	0.45	0.67	0.21	RPTOR	Body	shelf
cg24921221	0.21	0.0000	0.16	0.37	0.21	LONRF1	Body	opensea
cg05469819	0.21	0.0001	0.56	0.78	0.21		IGR	opensea
cg08085267	0.22	0.0005	0.13	0.34	0.22	C17orf57	5'UTR	shore
cg02782634	0.22	0.0000	0.27	0.49	0.22	TMEM49	Body	opensea
cg10587082	0.22	0.0020	0.44	0.66	0.22	PLXNA2	Body	opensea
cg04236915	0.23	0.0000	0.42	0.65	0.23	ECE1	Body	opensea
cg12403162	0.23	0.0000	0.26	0.49	0.23	ABLIM1	Body	shelf
cg05194426	0.23	0.0205	0.25	0.48	0.23	CYP2E1	Body	shore
cg12592365	0.23	0.0004	0.36	0.60	0.23	RPTOR	Body	opensea
cg04470054	0.24	0.0031	0.42	0.66	0.24	RPTOR	Body	shore
cg25588844	0.24	0.0001	0.40	0.64	0.24	TAF1B	Body	opensea
cg27477494	0.24	0.0002	0.33	0.57	0.24		IGR	opensea
cg17311132	0.24	0.0005	0.57	0.81	0.24		IGR	opensea
cg19683494	0.25	0.0088	0.36	0.60	0.25		IGR	shore
cg17749961	0.28	0.0000	0.04	0.31	0.28	LCLAT1	TSS1500	shore
cg12454169	0.30	0.0000	0.12	0.42	0.30	LCLAT1	TSS1500	shore
cg22443212	0.36	0.0023	0.32	0.68	0.36	RNF213	Body	opensea
cg15652532	0.36	0.0001	0.16	0.53	0.36	LCLAT1	TSS1500	shore

Supplemental Table 3. 261 differentially methylated positions in Non-ischemic HF vs. Non-Failing (adjusted p-value <0.05, abs (delta beta) > 10%)

CpG Site	log FC	Adj p-val	ICM	NF	Delta B	Gene	Feature	CGI
cg05528899	0.30	0.0188	0.65	0.35	-0.30		IGR	island
cg26536949	0.25	0.0134	0.75	0.51	-0.25		IGR	island
cg10327440	0.21	0.0281	0.63	0.42	-0.21	CDC42BPA	3'UTR	opensea
cg22459517	0.21	0.0110	0.50	0.29	-0.21	EPS8L1	TSS200	opensea
cg07167872	0.17	0.0325	0.41	0.24	-0.17	PM20D1	TSS200	shore
cg02928365	0.17	0.0000	0.43	0.26	-0.17	HLX	Body	shore
cg13283845	0.16	0.0169	0.73	0.57	-0.16		IGR	shore
cg02497785	0.16	0.0004	0.66	0.51	-0.16	ABCA13	Body	island
cg10507304	0.15	0.0001	0.42	0.26	-0.15		IGR	opensea
cg22056094	0.15	0.0003	0.67	0.52	-0.15	PCDHGA2	1stExon	shore
cg05995465	0.15	0.0410	0.83	0.68	-0.15	HDAC4	5'UTR	opensea
cg19657945	0.15	0.0255	0.90	0.75	-0.15		IGR	shore
cg14318858	0.14	0.0000	0.74	0.60	-0.14	CPT1C	Body	island
cg13752114	0.14	0.0133	0.82	0.68	-0.14	MUC4	Body	island
cg04811114	0.14	0.0123	0.35	0.21	-0.14	LGR6	TSS200	opensea
cg04392266	0.14	0.0013	0.76	0.62	-0.14		IGR	island
cg20070837	0.14	0.0101	0.93	0.79	-0.14	C14orf79	Body	opensea
cg25538415	0.14	0.0157	0.56	0.43	-0.14	DCAKD	TSS1500	shore
cg15847845	0.14	0.0334	0.82	0.69	-0.14	PLEC1	TSS1500	shore
cg00986825	0.14	0.0024	0.94	0.80	-0.14	EPN3	Body	island
cg17436134	0.14	0.0011	0.43	0.30	-0.14		IGR	shore
cg03654560	0.13	0.0024	0.59	0.46	-0.13		IGR	opensea
cg13065507	0.13	0.0185	0.56	0.42	-0.13		IGR	opensea
cg12563372	0.13	0.0000	0.45	0.32	-0.13		IGR	shore
cg07045469	0.13	0.0005	0.80	0.68	-0.13	ABCA13	Body	island
cg07555084	0.13	0.0148	0.68	0.55	-0.13		IGR	opensea
cg07028950	0.13	0.0022	0.68	0.55	-0.13		IGR	opensea
cg17735593	0.12	0.0051	0.42	0.30	-0.12	PCDHB7	1stExon	shore
cg13857646	0.12	0.0254	0.73	0.60	-0.12	PNPLA7	Body	island
cg26648818	0.12	0.0000	0.43	0.31	-0.12	TOX3	TSS200	shore
cg20618651	0.12	0.0273	0.76	0.64	-0.12	EXOC1	TSS1500	shore
cg01228941	0.12	0.0008	0.57	0.45	-0.12	PCDHB7	1stExon	shore
cg20454002	0.12	0.0005	0.46	0.34	-0.12	HLX	Body	shore
cg08355045	0.12	0.0279	0.68	0.56	-0.12		IGR	opensea
cg12211856	0.12	0.0000	0.29	0.17	-0.12	SDCCAG8	Body	island
cg24441899	0.12	0.0255	0.33	0.21	-0.12	SDK1	Body	opensea
cg08274176	0.12	0.0003	0.80	0.68	-0.12		IGR	shore
cg14101117	0.12	0.0116	0.46	0.34	-0.12	PARK2	Body	opensea

cg12777520	0.12	0.0008	0.47	0.35	-0.12	LMX1B	Body	island
cg19689427	0.12	0.0001	0.44	0.32	-0.12	PCDHGA2	1stExon	shore
cg16049600	0.12	0.0013	0.35	0.23	-0.12	PCDHB11	TSS200	shore
cg16298867	0.12	0.0050	0.57	0.45	-0.12		IGR	opensea
cg03329019	0.12	0.0003	0.58	0.47	-0.12		IGR	shore
cg23058405	0.12	0.0015	0.30	0.19	-0.12		IGR	island
cg23596123	0.12	0.0016	0.38	0.27	-0.12	PCDHB6	1stExon	shore
cg00817765	0.12	0.0000	0.66	0.54	-0.12		IGR	shore
cg11350586	0.12	0.0000	0.52	0.41	-0.12	SOX9	TSS1500	shore
cg26884658	0.12	0.0000	0.69	0.57	-0.12		IGR	shelf
cg03924115	0.11	0.0000	0.81	0.69	-0.11	QRFP	1stExon	opensea
cg25720795	0.11	0.0016	0.53	0.42	-0.11		IGR	shore
cg26680989	0.11	0.0000	0.76	0.64	-0.11		IGR	opensea
cg23936410	0.11	0.0346	0.38	0.27	-0.11		IGR	opensea
cg01107874	0.11	0.0002	0.69	0.58	-0.11	C10orf41	Body	island
cg18559901	0.11	0.0285	0.51	0.40	-0.11	PNPLA7	Body	island
cg23283495	0.11	0.0008	0.51	0.40	-0.11	IRF6	TSS1500	shore
cg06347454	0.11	0.0000	0.88	0.77	-0.11	ABCA13	Body	island
cg26669793	0.11	0.0081	0.47	0.36	-0.11	PRRX1	Body	shelf
cg10388307	0.11	0.0000	0.72	0.61	-0.11		IGR	opensea
cg24127414	0.11	0.0037	0.63	0.51	-0.11	PCDHB11	1stExon	shore
cg13012494	0.11	0.0239	0.27	0.16	-0.11	C21orf56	TSS1500	shelf
cg18463607	0.11	0.0126	0.82	0.71	-0.11	EXOC1	TSS1500	shore
cg22029157	0.11	0.0014	0.39	0.28	-0.11	IRF6	TSS200	island
cg01000188	0.11	0.0012	0.70	0.59	-0.11	QRFP	1stExon	opensea
cg11471802	0.11	0.0412	0.59	0.48	-0.11		IGR	island
cg24062763	0.11	0.0019	0.70	0.59	-0.11		IGR	island
cg18713687	0.11	0.0276	0.80	0.69	-0.11	MUC4	Body	island
cg15415259	0.11	0.0002	0.54	0.43	-0.11	MGC34034	Body	shore
cg05283184	0.11	0.0001	0.64	0.53	-0.11		IGR	shore
cg23289079	0.11	0.0044	0.50	0.39	-0.11	PRDM6	Body	shore
cg11679455	0.11	0.0005	0.62	0.51	-0.11	GATA3	Body	island
cg09834706	0.11	0.0014	0.82	0.71	-0.11		IGR	island
cg07381872	0.11	0.0119	0.73	0.62	-0.11		IGR	opensea
cg27582059	0.11	0.0003	0.50	0.40	-0.11	PCDHB16	TSS1500	shore
cg23043544	0.11	0.0000	0.90	0.79	-0.11	MAD1L1	Body	opensea
cg01310397	0.11	0.0124	0.81	0.71	-0.11	MUC4	Body	shore
cg15829969	0.11	0.0300	0.57	0.46	-0.11		IGR	opensea
cg05826245	0.11	0.0403	0.42	0.32	-0.11	STYK1	TSS1500	shore
cg04154653	0.11	0.0185	0.91	0.80	-0.11	TTLL10	3'UTR	shore
cg14774440	0.11	0.0001	0.65	0.54	-0.11	RAB11FIP1	Body	opensea
cg18011273	0.11	0.0016	0.62	0.51	-0.11	SORCS2	Body	opensea
cg00877329	0.11	0.0000	0.45	0.35	-0.11	HPSE2	TSS1500	shelf

cg15359163	0.11	0.0024	0.33	0.23	-0.11	PRDM6	Body	shore
cg14141912	0.11	0.0000	0.55	0.44	-0.11	ATOH8	Body	opensea
cg14065590	0.10	0.0012	0.70	0.60	-0.10	PCDHB11	TSS200	shore
cg27353899	0.10	0.0339	0.41	0.31	-0.10	MUC4	Body	island
cg03776662	0.10	0.0003	0.28	0.18	-0.10	PRDM6	Body	island
cg14963928	0.10	0.0177	0.55	0.45	-0.10		IGR	opensea
cg16316993	0.10	0.0047	0.47	0.36	-0.10	C14orf79	Body	shelf
cg03729337	0.10	0.0000	0.55	0.44	-0.10	PRDM6	Body	shore
cg11948367	0.10	0.0015	0.62	0.52	-0.10		IGR	opensea
cg14493094	0.10	0.0394	0.68	0.58	-0.10		IGR	island
cg10070864	0.10	0.0399	0.65	0.55	-0.10		IGR	shelf
cg27067781	0.10	0.0002	0.49	0.38	-0.10	PRRT1	3'UTR	island
cg14497054	0.10	0.0065	0.26	0.15	-0.10		IGR	island
cg22025206	0.10	0.0002	0.49	0.39	-0.10	SLC9A3	Body	shore
cg07994661	0.10	0.0000	0.49	0.39	-0.10		IGR	shore
cg19349713	0.10	0.0012	0.73	0.62	-0.10	CPEB1	Body	opensea
cg03014829	0.10	0.0114	0.75	0.65	-0.10		IGR	island
cg11430077	0.10	0.0000	0.63	0.52	-0.10	GATA3	Body	shore
cg02793451	0.10	0.0001	0.58	0.48	-0.10	TOX3	TSS1500	shore
cg27005118	0.10	0.0026	0.57	0.46	-0.10	COX10	TSS1500	shore
cg13829104	0.10	0.0005	0.72	0.62	-0.10	TBX3	Body	shore
cg12864721	0.10	0.0001	0.52	0.42	-0.10	C10orf41	Body	island
cg16200531	0.10	0.0006	0.55	0.45	-0.10		IGR	opensea
cg07703976	0.10	0.0002	0.88	0.78	-0.10	ABCA13	Body	island
cg21283066	0.10	0.0010	0.45	0.35	-0.10		IGR	opensea
cg07741162	0.10	0.0001	0.47	0.36	-0.10	PRDM6	Body	shore
cg01535205	0.10	0.0006	0.55	0.45	-0.10		IGR	opensea
cg11197101	0.10	0.0151	0.34	0.24	-0.10	KIAA1522	Body	island
cg02707854	0.10	0.0107	0.66	0.56	-0.10	SLC27A1	Body	shelf
cg21951975	0.10	0.0003	0.35	0.25	-0.10	IRF6	TSS1500	shore
cg13683361	0.10	0.0019	0.53	0.43	-0.10	LPPR1	5'UTR	shore
cg22041228	0.10	0.0022	0.38	0.28	-0.10	HLX	Body	shore
cg22770911	0.10	0.0001	0.52	0.42	-0.10	GATA3	Body	shore
cg01845041	0.10	0.0001	0.60	0.50	-0.10	IGSF11	Body	shore
cg01404163	0.10	0.0002	0.45	0.35	-0.10	TOX3	TSS200	shore
cg04438997	0.10	0.0001	0.48	0.38	-0.10	SOX9	TSS1500	shore
cg25503149	-0.10	0.0262	0.12	0.22	0.10		IGR	opensea
cg06234051	-0.10	0.0003	0.55	0.65	0.10	SOX9	3'UTR	shore
cg11802797	-0.10	0.0000	0.18	0.28	0.10	KIF1B	3'UTR	opensea
cg00159243	-0.10	0.0000	0.37	0.47	0.10	SELPLG	5'UTR	opensea
cg14986890	-0.10	0.0031	0.59	0.69	0.10	RARRES1	Body	opensea
cg15690347	-0.10	0.0113	0.23	0.33	0.10	SPIB	Body	island
cg18700940	-0.10	0.0003	0.20	0.30	0.10	MAP3K14	5'UTR	opensea

cg05940691	-0.10	0.0178	0.62	0.72	0.10	WDR64	Body	opensea
cg08426157	-0.10	0.0002	0.72	0.82	0.10	HDAC9	Body	opensea
cg01412419	-0.10	0.0445	0.66	0.76	0.10		IGR	opensea
cg03665360	-0.10	0.0008	0.21	0.31	0.10		IGR	opensea
cg10296718	-0.10	0.0003	0.80	0.90	0.10	ARHGEF10	Body	opensea
cg13051700	-0.10	0.0000	0.53	0.63	0.10	MAGI2	Body	opensea
cg14387312	-0.10	0.0014	0.49	0.60	0.10		IGR	opensea
cg12868173	-0.10	0.0055	0.22	0.32	0.10		IGR	island
cg19590421	-0.10	0.0000	0.29	0.39	0.10		IGR	opensea
cg19017553	-0.10	0.0007	0.69	0.79	0.10	PARD3	Body	opensea
cg10973622	-0.10	0.0335	0.51	0.61	0.10	IMMT	TSS1500	shore
cg26937434	-0.10	0.0006	0.74	0.84	0.10	ANKS4B	1stExon	opensea
cg13221458	-0.10	0.0000	0.18	0.29	0.10	SOD2	Body	shore
cg09447675	-0.10	0.0383	0.45	0.56	0.10		IGR	opensea
cg23959772	-0.10	0.0227	0.33	0.43	0.10		IGR	opensea
cg10974479	-0.10	0.0007	0.72	0.83	0.10	MLN	TSS1500	opensea
cg07791418	-0.10	0.0002	0.54	0.65	0.10		IGR	opensea
cg13636014	-0.10	0.0000	0.44	0.54	0.10		IGR	opensea
cg13052638	-0.10	0.0107	0.35	0.46	0.10		IGR	shelf
cg02564299	-0.10	0.0000	0.64	0.74	0.10	ZBTB20	5'UTR	opensea
cg16377679	-0.10	0.0002	0.47	0.57	0.10	PDE4D	Body	opensea
cg17800426	-0.10	0.0001	0.73	0.83	0.10	MYOZ3	TSS1500	shelf
cg21335012	-0.10	0.0000	0.77	0.87	0.10		IGR	opensea
cg24475182	-0.10	0.0124	0.28	0.38	0.10		IGR	opensea
cg24772753	-0.10	0.0192	0.25	0.35	0.10	SP5	Body	island
cg10557907	-0.11	0.0102	0.48	0.59	0.11	PRDM16	Body	opensea
cg18942579	-0.11	0.0003	0.19	0.30	0.11	TMEM49	Body	opensea
cg14713217	-0.11	0.0005	0.20	0.31	0.11		IGR	opensea
cg10110335	-0.11	0.0038	0.58	0.69	0.11	SYN2	Body	shelf
cg24736734	-0.11	0.0051	0.31	0.42	0.11		IGR	opensea
cg24062389	-0.11	0.0003	0.68	0.79	0.11	BIVM	Body	opensea
cg05543593	-0.11	0.0000	0.56	0.67	0.11		IGR	opensea
cg11562411	-0.11	0.0001	0.31	0.41	0.11	SCUBE3	Body	opensea
cg25773259	-0.11	0.0008	0.68	0.79	0.11		IGR	opensea
cg24611970	-0.11	0.0002	0.60	0.71	0.11		IGR	opensea
cg07813142	-0.11	0.0186	0.15	0.26	0.11	SP5	Body	island
cg12751644	-0.11	0.0412	0.43	0.54	0.11		IGR	shelf
cg18363918	-0.11	0.0101	0.28	0.39	0.11	IGLON5	Body	shore
cg01589587	-0.11	0.0009	0.33	0.43	0.11	BATF	Body	opensea
cg04057161	-0.11	0.0052	0.70	0.81	0.11		IGR	opensea
cg26203572	-0.11	0.0004	0.70	0.81	0.11		IGR	opensea
cg04128967	-0.11	0.0001	0.63	0.74	0.11		IGR	opensea
cg20366549	-0.11	0.0000	0.20	0.31	0.11	SCNN1A	Body	opensea

cg11118962	-0.11	0.0026	0.51	0.62	0.11		IGR	opensea
cg15645888	-0.11	0.0002	0.64	0.75	0.11	FBXO16	3'UTR	opensea
cg02784232	-0.11	0.0001	0.61	0.72	0.11	PHC3	Body	shore
cg15226275	-0.11	0.0007	0.40	0.51	0.11	FRK	TSS200	opensea
cg21106486	-0.11	0.0016	0.09	0.20	0.11	CR1L	Body	island
cg04760448	-0.11	0.0091	0.54	0.65	0.11	COL18A1	Body	island
cg06938601	-0.11	0.0008	0.54	0.65	0.11	TCERG1L	Body	opensea
cg05577810	-0.11	0.0090	0.11	0.22	0.11		IGR	island
cg19777067	-0.11	0.0370	0.24	0.34	0.11		IGR	island
cg26284735	-0.11	0.0003	0.52	0.63	0.11		IGR	shelf
cg02351277	-0.11	0.0041	0.32	0.43	0.11		IGR	opensea
cg18842353	-0.11	0.0000	0.18	0.29	0.11		IGR	opensea
cg11452329	-0.11	0.0000	0.43	0.54	0.11		IGR	opensea
cg16085649	-0.11	0.0037	0.30	0.41	0.11	AKAP13	Body	opensea
cg11906781	-0.11	0.0148	0.39	0.51	0.11	BRE	Body	opensea
cg15852787	-0.11	0.0004	0.73	0.84	0.11	FRMD6	5'UTR	opensea
cg10082398	-0.11	0.0008	0.59	0.71	0.11	CLDN20	5'UTR	opensea
cg26504263	-0.11	0.0008	0.65	0.76	0.11	ANKRD6	5'UTR	opensea
cg27291468	-0.11	0.0004	0.21	0.32	0.11		IGR	opensea
cg12024811	-0.11	0.0000	0.65	0.77	0.11	KALRN	Body	opensea
cg01409343	-0.11	0.0004	0.43	0.54	0.11	TMEM49	Body	opensea
cg14977018	-0.11	0.0003	0.77	0.89	0.11	TMEM44	Body	opensea
cg08889114	-0.11	0.0011	0.81	0.92	0.11		IGR	opensea
cg17178175	-0.11	0.0031	0.41	0.52	0.11	NFE2L2	Body	opensea
cg16002660	-0.11	0.0000	0.49	0.60	0.11	LOC284009	Body	opensea
cg22430036	-0.11	0.0001	0.78	0.89	0.11	CALD1	Body	opensea
cg05469819	-0.11	0.0002	0.66	0.78	0.11		IGR	opensea
cg05227773	-0.11	0.0010	0.66	0.78	0.11	ZFHX3	3'UTR	shelf
cg06474225	-0.12	0.0000	0.27	0.38	0.12	HTRA1	Body	opensea
cg11809668	-0.12	0.0001	0.69	0.81	0.12	KIAA0922	Body	opensea
cg14111334	-0.12	0.0007	0.67	0.78	0.12		IGR	opensea
cg04414720	-0.12	0.0144	0.19	0.31	0.12	GOLPH3L	TSS1500	opensea
cg01195564	-0.12	0.0044	0.63	0.74	0.12		IGR	opensea
cg03052078	-0.12	0.0001	0.53	0.65	0.12	STXBP5	Body	shore
cg01351822	-0.12	0.0001	0.07	0.18	0.12	UNC45A	5'UTR	island
cg02515217	-0.12	0.0002	0.50	0.61	0.12	MIR21	TSS200	opensea
cg24367957	-0.12	0.0093	0.42	0.54	0.12		IGR	opensea
cg11235602	-0.12	0.0254	0.27	0.39	0.12	MOBP	Body	island
cg07506560	-0.12	0.0016	0.61	0.74	0.12		IGR	opensea
cg21727223	-0.12	0.0001	0.37	0.49	0.12		IGR	opensea
cg01993169	-0.12	0.0003	0.77	0.89	0.12		IGR	opensea
cg01412970	-0.12	0.0291	0.32	0.44	0.12	PLD6	1stExon	island
cg16233797	-0.12	0.0001	0.69	0.82	0.12		IGR	opensea

cg26177041	-0.12	0.0000	0.23	0.35	0.12	CAMK2D	Body	opensea
cg01116477	-0.12	0.0000	0.63	0.75	0.12		IGR	opensea
cg17311132	-0.12	0.0003	0.69	0.81	0.12		IGR	opensea
cg10587082	-0.12	0.0283	0.54	0.66	0.12	PLXNA2	Body	opensea
cg25173405	-0.12	0.0058	0.13	0.25	0.12	C17orf57	5'UTR	shore
cg04228083	-0.13	0.0011	0.69	0.81	0.13	LOC100130872-SPON2	TSS200	shore
cg24631102	-0.13	0.0001	0.61	0.74	0.13	NPS	Body	opensea
cg24921221	-0.13	0.0009	0.25	0.37	0.13	LONRF1	Body	opensea
cg04926881	-0.13	0.0000	0.23	0.36	0.13		IGR	opensea
cg23201812	-0.13	0.0043	0.38	0.50	0.13		IGR	opensea
cg02573091	-0.13	0.0160	0.29	0.42	0.13		IGR	shore
cg20848291	-0.13	0.0026	0.26	0.39	0.13	ZAN	Body	opensea
cg07986257	-0.13	0.0001	0.68	0.81	0.13		IGR	opensea
cg15694704	-0.13	0.0002	0.54	0.67	0.13	RPTOR	Body	shelf
cg27149179	-0.13	0.0001	0.40	0.53	0.13		IGR	opensea
cg02992067	-0.13	0.0000	0.72	0.86	0.13	FTO	Body	opensea
cg09435170	-0.13	0.0000	0.17	0.30	0.13		IGR	opensea
cg13001142	-0.13	0.0000	0.65	0.78	0.13	STXBP5	Body	shelf
cg24113973	-0.14	0.0149	0.23	0.37	0.14		IGR	shelf
cg01923775	-0.14	0.0003	0.45	0.59	0.14	PALLD	Body	opensea
cg03839782	-0.14	0.0003	0.66	0.80	0.14	FAM65B	5'UTR	opensea
cg06850787	-0.14	0.0009	0.42	0.56	0.14		IGR	opensea
cg21144063	-0.14	0.0135	0.35	0.49	0.14		IGR	opensea
cg03264133	-0.14	0.0319	0.35	0.49	0.14		IGR	island
cg16337566	-0.14	0.0001	0.27	0.41	0.14	PINX1	Body	opensea
cg13173567	-0.14	0.0002	0.22	0.37	0.14	DENND5A	Body	opensea
cg03517284	-0.14	0.0156	0.33	0.48	0.14		IGR	shore
cg12686055	-0.15	0.0000	0.24	0.39	0.15	ANO6	Body	opensea
cg10316899	-0.15	0.0001	0.38	0.53	0.15	MACF1	Body	opensea
cg04470054	-0.15	0.0018	0.51	0.66	0.15	RPTOR	Body	shore
cg12403162	-0.15	0.0004	0.34	0.49	0.15	ABLIM1	Body	shelf
cg04236915	-0.15	0.0000	0.49	0.65	0.15	ECE1	Body	opensea
cg00601450	-0.16	0.0020	0.45	0.61	0.16		IGR	shore
cg25588844	-0.16	0.0000	0.48	0.64	0.16	TAF1B	Body	opensea
cg25755428	-0.16	0.0080	0.10	0.26	0.16	MRI1	TSS1500	island
cg16474696	-0.17	0.0113	0.24	0.41	0.17	MRI1	TSS1500	shore
cg23099839	-0.17	0.0022	0.26	0.43	0.17		IGR	opensea
cg12592365	-0.17	0.0005	0.42	0.60	0.17	RPTOR	Body	opensea
cg08085267	-0.18	0.0011	0.16	0.34	0.18	C17orf57	5'UTR	shore
cg27477494	-0.18	0.0003	0.39	0.57	0.18		IGR	opensea
cg24693760	-0.19	0.0105	0.34	0.53	0.19		IGR	opensea
cg17749961	-0.20	0.0049	0.12	0.31	0.20	LCLAT1	TSS1500	shore
cg02782634	-0.20	0.0000	0.29	0.49	0.20	TMEM49	Body	opensea

cg07147204	-0.20	0.0001	0.75	0.95	0.20		IGR	opensea
cg12454169	-0.20	0.0208	0.22	0.42	0.20	LCLAT1	TSS1500	shore
cg15532640	-0.21	0.0058	0.20	0.41	0.21		IGR	opensea
cg20905796	-0.22	0.0022	0.46	0.68	0.22		IGR	opensea
cg19318364	-0.23	0.0035	0.65	0.87	0.23		IGR	opensea
cg19683494	-0.23	0.0029	0.37	0.60	0.23		IGR	shore
cg15652532	-0.24	0.0160	0.28	0.53	0.24	LCLAT1	TSS1500	shore
cg22443212	-0.31	0.0005	0.37	0.68	0.31	RNF213	Body	opensea

Supplemental Table 4. 192 differentially methylated positions common to Ischemic and Non-Ischemic HF (adjusted p-value <0.05, abs (delta beta) > 10%)

CpG Site	Delta ICM	Delta NICM	Methylation	Gene	Feature	CGI
cg22443212	-0.36	-0.31	Hypomethylated	RNF213	Body	opensea
cg15652532	-0.36	-0.24	Hypomethylated	LCLAT1	TSS1500	shore
cg12454169	-0.30	-0.20	Hypomethylated	LCLAT1	TSS1500	shore
cg19683494	-0.25	-0.23	Hypomethylated		IGR	shore
cg17749961	-0.28	-0.20	Hypomethylated	LCLAT1	TSS1500	shore
cg20905796	-0.21	-0.22	Hypomethylated		IGR	opensea
cg27477494	-0.24	-0.18	Hypomethylated		IGR	opensea
cg02782634	-0.22	-0.20	Hypomethylated	TMEM49	Body	opensea
cg12592365	-0.23	-0.17	Hypomethylated	RPTOR	Body	opensea
cg25588844	-0.24	-0.16	Hypomethylated	TAF1B	Body	opensea
cg08085267	-0.22	-0.18	Hypomethylated	C17orf57	5'UTR	shore
cg04470054	-0.24	-0.15	Hypomethylated	RPTOR	Body	shore
cg04236915	-0.23	-0.15	Hypomethylated	ECE1	Body	opensea
cg12403162	-0.23	-0.15	Hypomethylated	ABLIM1	Body	shelf
cg24693760	-0.18	-0.19	Hypomethylated		IGR	opensea
cg17311132	-0.24	-0.12	Hypomethylated		IGR	opensea
cg23099839	-0.20	-0.17	Hypomethylated		IGR	opensea
cg10587082	-0.22	-0.12	Hypomethylated	PLXNA2	Body	opensea
cg15694704	-0.21	-0.13	Hypomethylated	RPTOR	Body	shelf
cg24921221	-0.21	-0.13	Hypomethylated	LONRF1	Body	opensea
cg21144063	-0.20	-0.14	Hypomethylated		IGR	opensea
cg10316899	-0.18	-0.15	Hypomethylated	MACF1	Body	opensea
cg00601450	-0.17	-0.16	Hypomethylated		IGR	shore
cg12686055	-0.18	-0.15	Hypomethylated	ANO6	Body	opensea
cg27149179	-0.20	-0.13	Hypomethylated		IGR	opensea
cg05469819	-0.21	-0.11	Hypomethylated		IGR	opensea
cg23201812	-0.20	-0.13	Hypomethylated		IGR	opensea
cg24367957	-0.21	-0.12	Hypomethylated		IGR	opensea
cg01923775	-0.19	-0.14	Hypomethylated	PALLD	Body	opensea
cg16233797	-0.20	-0.12	Hypomethylated		IGR	opensea
cg03839782	-0.18	-0.14	Hypomethylated	FAM65B	5'UTR	opensea
cg11906781	-0.20	-0.11	Hypomethylated	BRE	Body	opensea
cg02992067	-0.18	-0.13	Hypomethylated	FTO	Body	opensea
cg07986257	-0.18	-0.13	Hypomethylated		IGR	opensea
cg24113973	-0.17	-0.14	Hypomethylated		IGR	shelf
cg16337566	-0.16	-0.14	Hypomethylated	PINX1	Body	opensea
cg21727223	-0.19	-0.12	Hypomethylated		IGR	opensea
cg02515217	-0.19	-0.12	Hypomethylated	MIR21	TSS200	opensea
cg11809668	-0.19	-0.12	Hypomethylated	KIAA0922	Body	opensea

cg17178175	-0.19	-0.11	Hypomethylated	NFE2L2	Body	opensea
cg14986890	-0.20	-0.10	Hypomethylated	RARRES1	Body	opensea
cg09435170	-0.16	-0.13	Hypomethylated		IGR	opensea
cg13001142	-0.16	-0.13	Hypomethylated	STXBP5	Body	shelf
cg04926881	-0.17	-0.13	Hypomethylated		IGR	opensea
cg16002660	-0.18	-0.11	Hypomethylated	LOC284009	Body	opensea
cg11118962	-0.18	-0.11	Hypomethylated		IGR	opensea
cg26504263	-0.17	-0.11	Hypomethylated	ANKRD6	5'UTR	opensea
cg25773259	-0.18	-0.11	Hypomethylated		IGR	opensea
cg24062389	-0.18	-0.11	Hypomethylated	BIVM	Body	opensea
cg01412970	-0.16	-0.12	Hypomethylated	PLD6	1stExon	island
cg01195564	-0.17	-0.12	Hypomethylated		IGR	opensea
cg24772753	-0.18	-0.10	Hypomethylated	SP5	Body	island
cg02573091	-0.15	-0.13	Hypomethylated		IGR	shore
cg01116477	-0.16	-0.12	Hypomethylated		IGR	opensea
cg12024811	-0.17	-0.11	Hypomethylated	KALRN	Body	opensea
cg08426157	-0.18	-0.10	Hypomethylated	HDAC9	Body	opensea
cg21335012	-0.17	-0.10	Hypomethylated		IGR	opensea
cg20848291	-0.15	-0.13	Hypomethylated	ZAN	Body	opensea
cg01993169	-0.16	-0.12	Hypomethylated		IGR	opensea
cg11562411	-0.17	-0.11	Hypomethylated	SCUBE3	Body	opensea
cg17800426	-0.17	-0.10	Hypomethylated	MYOZ3	TSS1500	shelf
cg26203572	-0.17	-0.11	Hypomethylated		IGR	opensea
cg26177041	-0.15	-0.12	Hypomethylated	CAMK2D	Body	opensea
cg18700940	-0.17	-0.10	Hypomethylated	MAP3K14	5'UTR	opensea
cg13173567	-0.13	-0.14	Hypomethylated	DENND5A	Body	opensea
cg01409343	-0.16	-0.11	Hypomethylated	TMEM49	Body	opensea
cg15852787	-0.16	-0.11	Hypomethylated	FRMD6	5'UTR	opensea
cg05227773	-0.16	-0.11	Hypomethylated	ZFHX3	3'UTR	shelf
cg02351277	-0.16	-0.11	Hypomethylated		IGR	opensea
cg04760448	-0.16	-0.11	Hypomethylated	COL18A1	Body	island
cg25173405	-0.15	-0.12	Hypomethylated	C17orf57	5'UTR	shore
cg26284735	-0.16	-0.11	Hypomethylated		IGR	shelf
cg07813142	-0.16	-0.11	Hypomethylated	SP5	Body	island
cg24475182	-0.16	-0.10	Hypomethylated		IGR	opensea
cg10557907	-0.16	-0.11	Hypomethylated	PRDM16	Body	opensea
cg10110335	-0.16	-0.11	Hypomethylated	SYN2	Body	shelf
cg24736734	-0.16	-0.11	Hypomethylated		IGR	opensea
cg06474225	-0.15	-0.12	Hypomethylated	HTRA1	Body	opensea
cg07506560	-0.14	-0.12	Hypomethylated		IGR	opensea
cg14977018	-0.14	-0.11	Hypomethylated	TMEM44	Body	opensea
cg13052638	-0.15	-0.10	Hypomethylated		IGR	shelf
cg03052078	-0.14	-0.12	Hypomethylated	STXBP5	Body	shore

cg16377679	-0.15	-0.10	Hypomethylated	PDE4D	Body	opensea
cg04057161	-0.15	-0.11	Hypomethylated		IGR	opensea
cg18842353	-0.14	-0.11	Hypomethylated		IGR	opensea
cg16085649	-0.14	-0.11	Hypomethylated	AKAP13	Body	opensea
cg01412419	-0.15	-0.10	Hypomethylated		IGR	opensea
cg06850787	-0.11	-0.14	Hypomethylated		IGR	opensea
cg05543593	-0.14	-0.11	Hypomethylated		IGR	opensea
cg08889114	-0.13	-0.11	Hypomethylated		IGR	opensea
cg19017553	-0.14	-0.10	Hypomethylated	PARD3	Body	opensea
cg15645888	-0.14	-0.11	Hypomethylated	FBXO16	3'UTR	opensea
cg13636014	-0.14	-0.10	Hypomethylated		IGR	opensea
cg01589587	-0.14	-0.11	Hypomethylated	BATF	Body	opensea
cg11802797	-0.14	-0.10	Hypomethylated	KIF1B	3'UTR	opensea
cg24631102	-0.11	-0.13	Hypomethylated	NPS	Body	opensea
cg01351822	-0.12	-0.12	Hypomethylated	UNC45A	5'UTR	island
cg12868173	-0.13	-0.10	Hypomethylated		IGR	island
cg13221458	-0.13	-0.10	Hypomethylated	SOD2	Body	shore
cg11452329	-0.13	-0.11	Hypomethylated		IGR	opensea
cg04228083	-0.11	-0.13	Hypomethylated	LOC100130872-SPON2	TSS200	shore
cg02564299	-0.13	-0.10	Hypomethylated	ZBTB20	5'UTR	opensea
cg14111334	-0.12	-0.12	Hypomethylated		IGR	opensea
cg18363918	-0.13	-0.11	Hypomethylated	IGLON5	Body	shore
cg14713217	-0.13	-0.11	Hypomethylated		IGR	opensea
cg04128967	-0.13	-0.11	Hypomethylated		IGR	opensea
cg24611970	-0.13	-0.11	Hypomethylated		IGR	opensea
cg13051700	-0.13	-0.10	Hypomethylated	MAGI2	Body	opensea
cg10082398	-0.12	-0.11	Hypomethylated	CLDN20	5'UTR	opensea
cg06938601	-0.12	-0.11	Hypomethylated	TCERG1L	Body	opensea
cg02784232	-0.12	-0.11	Hypomethylated	PHC3	Body	shore
cg14387312	-0.12	-0.10	Hypomethylated		IGR	opensea
cg07791418	-0.12	-0.10	Hypomethylated		IGR	opensea
cg18942579	-0.12	-0.11	Hypomethylated	TMEM49	Body	opensea
cg00159243	-0.12	-0.10	Hypomethylated	SELPLG	5'UTR	opensea
cg15690347	-0.12	-0.10	Hypomethylated	SPIB	Body	island
cg03665360	-0.12	-0.10	Hypomethylated		IGR	opensea
cg21106486	-0.11	-0.11	Hypomethylated	CR1L	Body	island
cg20366549	-0.11	-0.11	Hypomethylated	SCNN1A	Body	opensea
cg10974479	-0.11	-0.10	Hypomethylated	MLN	TSS1500	opensea
cg26937434	-0.11	-0.10	Hypomethylated	ANKS4B	1stExon	opensea
cg10296718	-0.11	-0.10	Hypomethylated	ARHGEF10	Body	opensea
cg15226275	-0.10	-0.11	Hypomethylated	FRK	TSS200	opensea
cg19590421	-0.10	-0.10	Hypomethylated		IGR	opensea

cg06234051	-0.10	-0.10	Hypomethylated	SOX9	3'UTR	shore
cg01404163	0.10	0.10	Hypermethylated	TOX3	TSS200	shore
cg22025206	0.10	0.10	Hypermethylated	SLC9A3	Body	shore
cg02793451	0.11	0.10	Hypermethylated	TOX3	TSS1500	shore
cg07741162	0.11	0.10	Hypermethylated	PRDM6	Body	shore
cg27353899	0.11	0.10	Hypermethylated	MUC4	Body	island
cg14065590	0.11	0.10	Hypermethylated	PCDHB11	TSS200	shore
cg11948367	0.11	0.10	Hypermethylated		IGR	opensea
cg03776662	0.11	0.10	Hypermethylated	PRDM6	Body	island
cg27067781	0.11	0.10	Hypermethylated	PRRT1	3'UTR	island
cg14141912	0.11	0.11	Hypermethylated	ATOH8	Body	opensea
cg16049600	0.10	0.12	Hypermethylated	PCDHB11	TSS200	shore
cg14497054	0.12	0.10	Hypermethylated		IGR	island
cg00877329	0.12	0.11	Hypermethylated	HPSE2	TSS1500	shelf
cg05826245	0.12	0.11	Hypermethylated	STYK1	TSS1500	shore
cg04438997	0.12	0.10	Hypermethylated	SOX9	TSS1500	shore
cg24127414	0.11	0.11	Hypermethylated	PCDHB11	1stExon	shore
cg12864721	0.12	0.10	Hypermethylated	C10orf41	Body	island
cg10388307	0.12	0.11	Hypermethylated		IGR	opensea
cg11430077	0.13	0.10	Hypermethylated	GATA3	Body	shore
cg22041228	0.13	0.10	Hypermethylated	HLX	Body	shore
cg15359163	0.12	0.11	Hypermethylated	PRDM6	Body	shore
cg04154653	0.13	0.11	Hypermethylated	TTLL10	3'UTR	shore
cg01310397	0.12	0.11	Hypermethylated	MUC4	Body	shore
cg18713687	0.12	0.11	Hypermethylated	MUC4	Body	island
cg12211856	0.11	0.12	Hypermethylated	SDCCAG8	Body	island
cg15415259	0.13	0.11	Hypermethylated	MGC34034	Body	shore
cg22770911	0.13	0.10	Hypermethylated	GATA3	Body	shore
cg18463607	0.13	0.11	Hypermethylated	EXOC1	TSS1500	shore
cg23596123	0.12	0.12	Hypermethylated	PCDHB6	1stExon	shore
cg25720795	0.13	0.11	Hypermethylated		IGR	shore
cg14774440	0.14	0.11	Hypermethylated	RAB11FIP1	Body	opensea
cg01535205	0.14	0.10	Hypermethylated		IGR	opensea
cg11350586	0.13	0.12	Hypermethylated	SOX9	TSS1500	shore
cg26884658	0.13	0.12	Hypermethylated		IGR	shelf
cg23289079	0.14	0.11	Hypermethylated	PRDM6	Body	shore
cg13829104	0.14	0.10	Hypermethylated	TBX3	Body	shore
cg16298867	0.13	0.12	Hypermethylated		IGR	opensea
cg26680989	0.14	0.11	Hypermethylated		IGR	opensea
cg07028950	0.12	0.13	Hypermethylated		IGR	opensea
cg19689427	0.13	0.12	Hypermethylated	PCDHGA2	1stExon	shore
cg24441899	0.13	0.12	Hypermethylated	SDK1	Body	opensea
cg26648818	0.13	0.12	Hypermethylated	TOX3	TSS200	shore

cg21283066	0.15	0.10	Hypermethylated		IGR	opensea
cg16200531	0.16	0.10	Hypermethylated		IGR	opensea
cg02497785	0.11	0.16	Hypermethylated	ABCA13	Body	island
cg04392266	0.13	0.14	Hypermethylated		IGR	island
cg20618651	0.15	0.12	Hypermethylated	EXOC1	TSS1500	shore
cg01107874	0.16	0.11	Hypermethylated	C10orf41	Body	island
cg20454002	0.15	0.12	Hypermethylated	HLX	Body	shore
cg03329019	0.15	0.12	Hypermethylated		IGR	shore
cg11679455	0.16	0.11	Hypermethylated	GATA3	Body	island
cg23936410	0.16	0.11	Hypermethylated		IGR	opensea
cg10070864	0.17	0.10	Hypermethylated		IGR	shelf
cg10507304	0.14	0.15	Hypermethylated		IGR	opensea
cg22056094	0.14	0.15	Hypermethylated	PCDHGA2	1stExon	shore
cg13752114	0.16	0.14	Hypermethylated	MUC4	Body	island
cg17436134	0.17	0.14	Hypermethylated		IGR	shore
cg19657945	0.17	0.15	Hypermethylated		IGR	shore
cg12563372	0.18	0.13	Hypermethylated		IGR	shore
cg14318858	0.17	0.14	Hypermethylated	CPT1C	Body	island
cg12777520	0.20	0.12	Hypermethylated	LMX1B	Body	island
cg02928365	0.18	0.17	Hypermethylated	HLX	Body	shore
cg13283845	0.20	0.16	Hypermethylated		IGR	shore
cg04811114	0.26	0.14	Hypermethylated	LGR6	TSS200	opensea
cg26536949	0.25	0.25	Hypermethylated		IGR	island
cg07167872	0.34	0.17	Hypermethylated	PM20D1	TSS200	shore
cg05528899	0.33	0.30	Hypermethylated		IGR	island

Supplemental Table 5. 1075 differentially methylated positions in Pre-LVAD vs. Non-Failing (adjusted p-value <0.05, abs (delta beta) > 10%)

CpG Site	log FC	Adj p-val	ICM	NF	Delta B	Gene	Feature	CGI
cg07167872	0.23	0.0202	0.47	0.24	-0.23	PM20D1	TSS200	shore
cg14159672	0.21	0.0486	0.43	0.23	-0.21	PM20D1	1stExon	island
cg04811114	0.20	0.0026	0.41	0.21	-0.20	LGR6	TSS200	opensea
cg10507304	0.18	0.0003	0.44	0.26	-0.18		IGR	opensea
cg02928365	0.18	0.0007	0.44	0.26	-0.18	HLX	Body	shore
cg12777520	0.17	0.0013	0.52	0.35	-0.17	LMX1B	Body	island
cg17436134	0.17	0.0024	0.47	0.30	-0.17		IGR	shore
cg14318858	0.17	0.0003	0.77	0.60	-0.17	CPT1C	Body	island
cg11679455	0.16	0.0001	0.68	0.51	-0.16	GATA3	Body	island
cg12563372	0.16	0.0004	0.48	0.32	-0.16		IGR	shore
cg24503407	0.16	0.0323	0.56	0.40	-0.16	PM20D1	TSS1500	shore
cg03329019	0.16	0.0001	0.62	0.47	-0.16		IGR	shore
cg09559189	0.16	0.0012	0.37	0.22	-0.16	EBF2	Body	shore
cg22280475	0.16	0.0017	0.34	0.18	-0.16	EBF2	Body	island
cg12583076	0.15	0.0218	0.54	0.39	-0.15	RASSF3	Body	opensea
cg03731740	0.15	0.0025	0.54	0.39	-0.15	YTHDF2	TSS1500	shore
cg04492228	0.15	0.0000	0.57	0.42	-0.15	GATA3	Body	shore
cg01190989	0.15	0.0013	0.45	0.30	-0.15	GPR125	Body	opensea
cg11936410	0.15	0.0082	0.61	0.46	-0.15	TPM1	Body	shelf
cg04290171	0.14	0.0018	0.64	0.49	-0.14	CD46	TSS1500	shore
cg01107874	0.14	0.0033	0.72	0.58	-0.14	C10orf41	Body	island
cg01900030	0.14	0.0060	0.71	0.57	-0.14	CDK6	Body	opensea
cg02455346	0.14	0.0002	0.66	0.52	-0.14	HLX	Body	shore
cg05748163	0.14	0.0017	0.32	0.18	-0.14	EBF2	Body	shore
cg08070200	0.14	0.0099	0.51	0.36	-0.14	FAT1	Body	opensea
cg12930727	0.14	0.0190	0.44	0.30	-0.14	HYAL1	TSS1500	opensea
cg05214708	0.14	0.0039	0.45	0.31	-0.14	KLF7	Body	opensea
cg02497785	0.14	0.0009	0.65	0.51	-0.14	ABCA13	Body	island
cg10653297	0.14	0.0134	0.67	0.53	-0.14	LOC285033	TSS200	opensea
cg14204784	0.14	0.0051	0.49	0.35	-0.14	LMX1B	Body	island
cg11350586	0.14	0.0001	0.55	0.41	-0.14	SOX9	TSS1500	shore
cg07555084	0.14	0.0346	0.69	0.55	-0.14		IGR	opensea
cg22770911	0.14	0.0000	0.56	0.42	-0.14	GATA3	Body	shore
cg01525538	0.14	0.0052	0.43	0.30	-0.14	DUSP5P	Body	island
cg20454002	0.14	0.0046	0.47	0.34	-0.14	HLX	Body	shore
cg22946147	0.14	0.0148	0.61	0.48	-0.14	ZNF804B	Body	opensea
cg08370082	0.14	0.0118	0.59	0.45	-0.14	SENP5	Body	opensea
cg15415259	0.14	0.0002	0.56	0.43	-0.14	MGC34034	Body	shore
cg18242288	0.14	0.0062	0.81	0.67	-0.14		IGR	opensea

cg26481249	0.14	0.0125	0.48	0.34	-0.14	SORBS2	5'UTR	opensea
cg04160030	0.14	0.0081	0.57	0.43	-0.14	FUCA1	TSS1500	shore
cg23080355	0.14	0.0207	0.71	0.57	-0.14		IGR	opensea
cg07381872	0.14	0.0381	0.76	0.62	-0.14		IGR	opensea
cg02723558	0.14	0.0085	0.53	0.40	-0.14	BDNFOS	Body	opensea
cg19689427	0.13	0.0001	0.45	0.32	-0.13	PCDHGA2	1stExon	shore
cg06317507	0.13	0.0011	0.41	0.27	-0.13	MGC34034	Body	island
cg24060730	0.13	0.0185	0.68	0.54	-0.13	CAV1	Body	opensea
cg12211856	0.13	0.0000	0.30	0.17	-0.13	SDCCAG8	Body	island
cg14774440	0.13	0.0006	0.67	0.54	-0.13	RAB11FIP1	Body	opensea
cg25451765	0.13	0.0107	0.58	0.45	-0.13	SDK1	Body	opensea
cg26680989	0.13	0.0002	0.77	0.64	-0.13		IGR	opensea
cg06118122	0.13	0.0115	0.56	0.43	-0.13	LGALS8	Body	opensea
cg01300495	0.13	0.0098	0.70	0.58	-0.13	LHFP	Body	opensea
cg12910268	0.13	0.0039	0.65	0.52	-0.13	DNAJB6	Body	opensea
cg18239431	0.13	0.0025	0.28	0.16	-0.13	EBF2	Body	shore
cg23387597	0.13	0.0067	0.60	0.47	-0.13	ITPRIP	TSS200	shelf
cg01285926	0.13	0.0109	0.36	0.23	-0.13		IGR	island
cg01302853	0.13	0.0119	0.72	0.59	-0.13	MAD1L1	Body	opensea
cg10902396	0.13	0.0159	0.49	0.36	-0.13	CHN2	Body	opensea
cg16732787	0.13	0.0074	0.54	0.41	-0.13	DUSP5P	Body	island
cg10248878	0.13	0.0007	0.44	0.32	-0.13	GSX2	TSS1500	shore
cg15273575	0.13	0.0000	0.87	0.75	-0.13	UTRN	Body	opensea
cg05357209	0.13	0.0035	0.66	0.54	-0.13	UNC84A	5'UTR	opensea
cg01228941	0.13	0.0030	0.57	0.45	-0.13	PCDHB7	1stExon	shore
cg10070864	0.13	0.0391	0.67	0.55	-0.13		IGR	shelf
cg00991744	0.13	0.0228	0.62	0.49	-0.13	PDZRN4	TSS1500	shore
cg07226964	0.13	0.0233	0.48	0.36	-0.13	PPARGC1B	Body	opensea
cg21283066	0.12	0.0016	0.47	0.35	-0.12		IGR	opensea
cg06410057	0.12	0.0040	0.31	0.19	-0.12		IGR	shore
cg24953078	0.12	0.0053	0.51	0.39	-0.12	NTM	Body	opensea
cg12334013	0.12	0.0008	0.47	0.35	-0.12	EEPD1	Body	shelf
cg27067781	0.12	0.0006	0.51	0.38	-0.12	PRRT1	3'UTR	island
cg13563298	0.12	0.0385	0.52	0.40	-0.12	WNK2	Body	shore
cg14855519	0.12	0.0035	0.39	0.26	-0.12	EBF2	Body	shore
cg24127414	0.12	0.0121	0.64	0.51	-0.12	PCDHB11	1stExon	shore
cg22056094	0.12	0.0124	0.65	0.52	-0.12	PCDHGA2	1stExon	shore
cg11430077	0.12	0.0002	0.65	0.52	-0.12	GATA3	Body	shore
cg00630991	0.12	0.0192	0.56	0.44	-0.12	BCL2L14	3'UTR	opensea
cg01535205	0.12	0.0038	0.57	0.45	-0.12		IGR	opensea
cg20494563	0.12	0.0033	0.39	0.27	-0.12		IGR	opensea
cg13997553	0.12	0.0051	0.60	0.48	-0.12	TNC	Body	opensea
cg05290820	0.12	0.0034	0.38	0.26	-0.12		IGR	shore

cg22822630	0.12	0.0076	0.57	0.45	-0.12	USP12	Body	opensea
cg10207609	0.12	0.0179	0.48	0.36	-0.12	CD36	TSS1500	opensea
cg26484001	0.12	0.0175	0.46	0.34	-0.12		IGR	shelf
cg08005692	0.12	0.0149	0.32	0.20	-0.12	ASH1L	5'UTR	opensea
cg19306970	0.12	0.0036	0.48	0.36	-0.12	HLX	Body	shore
cg06206957	0.12	0.0097	0.54	0.42	-0.12	NT5C3	TSS200	opensea
cg27625491	0.12	0.0115	0.40	0.28	-0.12	CD36	TSS1500	opensea
cg14101117	0.12	0.0338	0.46	0.34	-0.12	PARK2	Body	opensea
cg17083209	0.12	0.0263	0.35	0.23	-0.12	LPHN2	5'UTR	opensea
cg13475333	0.12	0.0141	0.38	0.26	-0.12		IGR	shore
cg24838345	0.12	0.0414	0.65	0.53	-0.12	MTSS1	Body	shelf
cg23289079	0.12	0.0095	0.51	0.39	-0.12	PRDM6	Body	shore
cg26067760	0.12	0.0000	0.45	0.33	-0.12	HPSE2	TSS1500	shelf
cg16200531	0.12	0.0074	0.57	0.45	-0.12		IGR	opensea
cg07045469	0.12	0.0008	0.80	0.68	-0.12	ABCA13	Body	island
cg26393261	0.12	0.0010	0.69	0.57	-0.12	ATXN1	5'UTR	opensea
cg20570797	0.12	0.0046	0.38	0.26	-0.12	PXDN	Body	opensea
cg07050692	0.12	0.0253	0.40	0.28	-0.12	CHD7	Body	opensea
cg22897615	0.12	0.0013	0.33	0.21	-0.12	PRRT1	Body	island
cg24382823	0.12	0.0125	0.38	0.26	-0.12	LMX1B	Body	island
cg10512202	0.12	0.0198	0.61	0.49	-0.12	LIMD1	Body	opensea
cg01581084	0.12	0.0102	0.38	0.26	-0.12	OSR2	Body	shore
cg04322298	0.12	0.0317	0.60	0.48	-0.12	ORMDL1	3'UTR	opensea
cg11736230	0.12	0.0151	0.35	0.23	-0.12	PPP1R13B	Body	shore
cg17117243	0.12	0.0145	0.61	0.49	-0.12	SESN1	Body	opensea
cg16298867	0.12	0.0444	0.57	0.45	-0.12		IGR	opensea
cg04244354	0.12	0.0092	0.34	0.22	-0.12		IGR	shore
cg25979526	0.12	0.0171	0.69	0.58	-0.12	MYO5A	Body	opensea
cg23246095	0.12	0.0272	0.64	0.52	-0.12	DPP4	TSS1500	shore
cg17489908	0.12	0.0025	0.65	0.53	-0.12	GATA3	Body	shore
cg17735593	0.12	0.0077	0.41	0.30	-0.12	PCDHB7	1stExon	shore
cg07000514	0.12	0.0059	0.75	0.63	-0.12	C7orf50	Body	island
cg15359163	0.12	0.0093	0.35	0.23	-0.12	PRDM6	Body	shore
cg16310415	0.12	0.0001	0.33	0.21	-0.12	EBF2	Body	shore
cg11024506	0.12	0.0136	0.59	0.47	-0.12	RHOBTB1	Body	opensea
cg12190994	0.12	0.0002	0.71	0.59	-0.12	SDK1	Body	island
cg04839409	0.12	0.0118	0.55	0.44	-0.12	CTBP2	Body	opensea
cg08210507	0.12	0.0050	0.88	0.77	-0.12	MAD1L1	Body	shore
cg04471192	0.12	0.0040	0.78	0.66	-0.12		IGR	opensea
cg00914726	0.12	0.0491	0.44	0.32	-0.12	C1orf87	1stExon	island
cg08559364	0.12	0.0186	0.32	0.20	-0.12	VGLL4	Body	opensea
cg16725974	0.12	0.0178	0.41	0.29	-0.12	SYNE2	5'UTR	opensea
cg27065003	0.12	0.0032	0.43	0.32	-0.12	PRDM6	Body	shore

cg03776662	0.12	0.0004	0.30	0.18	-0.12	PRDM6	Body	island
cg26648818	0.12	0.0005	0.42	0.31	-0.12	TOX3	TSS200	shore
cg13466694	0.12	0.0058	0.47	0.35	-0.12	LMX1B	Body	shelf
cg21899520	0.12	0.0029	0.57	0.45	-0.12	CYB5A	Body	shore
cg23077820	0.12	0.0010	0.40	0.29	-0.12	PAX3	Body	shore
cg03080147	0.12	0.0007	0.84	0.72	-0.12	MTMR7	Body	shore
cg13829104	0.12	0.0002	0.73	0.62	-0.12	TBX3	Body	shore
cg02427468	0.12	0.0137	0.37	0.26	-0.12	ESYT2	Body	opensea
cg10163955	0.12	0.0003	0.85	0.73	-0.12	GATA3	Body	shore
cg20178924	0.12	0.0276	0.71	0.59	-0.12	ANKRD1	Body	opensea
cg15580052	0.12	0.0127	0.33	0.21	-0.12	B4GALNT3	Body	opensea
cg18011273	0.11	0.0162	0.62	0.51	-0.11	SORCS2	Body	opensea
cg26884658	0.11	0.0006	0.69	0.57	-0.11		IGR	shelf
cg18516067	0.11	0.0023	0.57	0.45	-0.11		IGR	opensea
cg02762475	0.11	0.0393	0.35	0.24	-0.11	SLC12A7	Body	shelf
cg26500142	0.11	0.0016	0.55	0.44	-0.11		IGR	shore
cg03348100	0.11	0.0001	0.70	0.59	-0.11		IGR	shelf
cg07746514	0.11	0.0239	0.72	0.61	-0.11	MLEC	3'UTR	opensea
cg00095431	0.11	0.0030	0.48	0.36	-0.11		IGR	opensea
cg26669793	0.11	0.0131	0.47	0.36	-0.11	PRRX1	Body	shelf
cg04982834	0.11	0.0004	0.43	0.32	-0.11	EVI5	TSS1500	opensea
cg26312807	0.11	0.0313	0.36	0.25	-0.11		IGR	island
cg00765233	0.11	0.0005	0.84	0.72	-0.11	SH3BP4	5'UTR	opensea
cg21474247	0.11	0.0089	0.74	0.63	-0.11		IGR	opensea
cg04438997	0.11	0.0010	0.49	0.38	-0.11	SOX9	TSS1500	shore
cg05427639	0.11	0.0037	0.51	0.40	-0.11	NT5C3	1stExon	opensea
cg05146756	0.11	0.0227	0.47	0.35	-0.11	LOC554202	Body	shore
cg12302647	0.11	0.0028	0.52	0.41	-0.11	ABLIM3	Body	opensea
cg16907496	0.11	0.0020	0.60	0.49	-0.11	CNTN4	Body	opensea
cg01262952	0.11	0.0051	0.53	0.41	-0.11	ANKRD1	1stExon	opensea
cg14959425	0.11	0.0182	0.59	0.48	-0.11	ITGB8	Body	opensea
cg00297075	0.11	0.0015	0.58	0.47	-0.11		IGR	shore
cg17230535	0.11	0.0082	0.67	0.56	-0.11		IGR	opensea
cg08785724	0.11	0.0000	0.41	0.30	-0.11		IGR	opensea
cg02128244	0.11	0.0007	0.41	0.30	-0.11		IGR	island
cg16049600	0.11	0.0099	0.34	0.23	-0.11	PCDHB11	TSS200	shore
cg03329576	0.11	0.0007	0.49	0.38	-0.11		IGR	opensea
cg23490829	0.11	0.0128	0.56	0.44	-0.11	PCDHA1	Body	shelf
cg02848875	0.11	0.0453	0.59	0.47	-0.11	SNX1	TSS1500	shore
cg00519208	0.11	0.0013	0.63	0.52	-0.11	EVI5	TSS1500	opensea
cg19763428	0.11	0.0042	0.37	0.26	-0.11	PDE1C	Body	shore
cg00923880	0.11	0.0007	0.58	0.47	-0.11		IGR	shore
cg06347454	0.11	0.0008	0.88	0.77	-0.11	ABCA13	Body	island

cg25720795	0.11	0.0165	0.53	0.42	-0.11		IGR	shore
cg00877329	0.11	0.0002	0.46	0.35	-0.11	HPSE2	TSS1500	shelf
cg07046818	0.11	0.0076	0.39	0.28	-0.11	GDF6	Body	shelf
cg09378783	0.11	0.0071	0.60	0.48	-0.11	EGFR	Body	opensea
cg24436715	0.11	0.0022	0.27	0.16	-0.11		IGR	island
cg14612733	0.11	0.0019	0.61	0.50	-0.11	EFNA1	Body	shelf
cg09936799	0.11	0.0053	0.44	0.33	-0.11	PCDHA6	Body	shore
cg18044113	0.11	0.0004	0.70	0.59	-0.11	MAD1L1	Body	opensea
cg16530086	0.11	0.0018	0.44	0.33	-0.11	CUGBP2	Body	shelf
cg25887789	0.11	0.0096	0.71	0.60	-0.11	PDZRN4	TSS1500	shore
cg03061778	0.11	0.0045	0.75	0.64	-0.11		IGR	opensea
cg12587213	0.11	0.0402	0.47	0.36	-0.11	SRGAP2	Body	opensea
cg19699264	0.11	0.0017	0.89	0.78	-0.11	SDPR	Body	opensea
cg19780570	0.11	0.0069	0.61	0.50	-0.11		IGR	opensea
cg24760869	0.11	0.0041	0.41	0.30	-0.11	ABLIM1	Body	opensea
cg19242851	0.11	0.0181	0.74	0.63	-0.11	RPH3AL	Body	opensea
cg04657684	0.11	0.0053	0.54	0.43	-0.11	ELMOD1	Body	opensea
cg12392104	0.11	0.0064	0.38	0.27	-0.11	CCPG1	Body	opensea
cg26919014	0.11	0.0114	0.69	0.58	-0.11	MMP15	Body	shore
cg22041228	0.11	0.0065	0.39	0.28	-0.11	HLX	Body	shore
cg19561274	0.11	0.0275	0.70	0.59	-0.11	LOC285033	TSS1500	opensea
cg06595211	0.11	0.0415	0.68	0.57	-0.11		IGR	opensea
cg26926765	0.11	0.0349	0.46	0.35	-0.11	C6orf142	Body	opensea
cg02551980	0.11	0.0449	0.42	0.31	-0.11	MCC	Body	shore
cg11223622	0.11	0.0001	0.38	0.27	-0.11	ITGB4	Body	island
cg24699699	0.11	0.0010	0.61	0.50	-0.11	EVI5	TSS1500	opensea
cg11276172	0.11	0.0074	0.61	0.50	-0.11	ITPRIP	TSS200	shelf
cg07145988	0.11	0.0152	0.57	0.46	-0.11	RERE	Body	opensea
cg17936488	0.11	0.0157	0.40	0.29	-0.11	FAM78A	1stExon	shore
cg09430976	0.11	0.0024	0.35	0.24	-0.11		IGR	shore
cg11661493	0.11	0.0032	0.71	0.60	-0.11	UBE2O	Body	opensea
cg18500988	0.11	0.0033	0.54	0.43	-0.11		IGR	shore
cg10362869	0.11	0.0026	0.47	0.36	-0.11	NCKAP5	TSS1500	opensea
cg06076692	0.11	0.0430	0.68	0.57	-0.11	ATXN1	5'UTR	opensea
cg06013117	0.11	0.0080	0.74	0.63	-0.11	MSX2	Body	shore
cg06704455	0.11	0.0053	0.41	0.31	-0.11	ELFN2	5'UTR	shore
cg14065590	0.11	0.0038	0.70	0.60	-0.11	PCDHB11	TSS200	shore
cg24125710	0.11	0.0031	0.46	0.35	-0.11		IGR	opensea
cg13687594	0.11	0.0188	0.51	0.41	-0.11	CAMTA1	Body	shore
cg14141912	0.11	0.0026	0.55	0.44	-0.11	ATOH8	Body	opensea
cg04213746	0.11	0.0028	0.80	0.69	-0.11	GATA3	Body	opensea
cg22480109	0.11	0.0252	0.36	0.25	-0.11	ESYT2	Body	opensea
cg08554603	0.11	0.0026	0.62	0.51	-0.11	C7orf58	Body	opensea

cg17160666	0.11	0.0084	0.68	0.57	-0.11		IGR	opensea
cg09122414	0.11	0.0166	0.41	0.30	-0.11	INPP5B	Body	opensea
cg07703976	0.11	0.0001	0.89	0.78	-0.11	ABCA13	Body	island
cg11236452	0.11	0.0020	0.46	0.35	-0.11	PCDHGA2	Body	shore
cg26819695	0.11	0.0038	0.40	0.30	-0.11	ASAM	TSS1500	island
cg26196860	0.11	0.0124	0.77	0.66	-0.11		IGR	opensea
cg11842367	0.11	0.0002	0.62	0.51	-0.11	MGC27382	Body	opensea
cg13250209	0.11	0.0083	0.76	0.65	-0.11	CCDC85C	Body	opensea
cg11125249	0.11	0.0090	0.58	0.48	-0.11	GYG1	Body	opensea
cg12864721	0.11	0.0058	0.53	0.42	-0.11	C10orf41	Body	island
cg24677093	0.11	0.0010	0.77	0.66	-0.11	C10orf41	Body	island
cg10180052	0.11	0.0040	0.64	0.53	-0.11	RNF151	Body	island
cg14197071	0.11	0.0012	0.64	0.53	-0.11	PRDM8	5'UTR	island
cg18645081	0.11	0.0010	0.44	0.33	-0.11	HLX	TSS1500	shore
cg22332722	0.11	0.0188	0.46	0.36	-0.11	CDH2	Body	shore
cg19494574	0.11	0.0148	0.54	0.44	-0.11		IGR	opensea
cg24807169	0.11	0.0078	0.41	0.30	-0.11	EMCN	TSS1500	opensea
cg02081006	0.11	0.0093	0.41	0.30	-0.11	PRDM6	Body	shore
cg13761419	0.11	0.0054	0.44	0.33	-0.11		IGR	shelf
cg14871588	0.11	0.0013	0.47	0.36	-0.11		IGR	shore
cg05044291	0.11	0.0011	0.46	0.35	-0.11	LGR6	TSS200	opensea
cg18004701	0.11	0.0168	0.26	0.15	-0.11	PTPRN2	Body	island
cg04566694	0.11	0.0205	0.44	0.33	-0.11	H6PD	Body	opensea
cg09672452	0.11	0.0010	0.33	0.23	-0.11	CCPG1	Body	opensea
cg10786043	0.11	0.0087	0.53	0.43	-0.11	TACC1	TSS1500	shore
cg11190278	0.11	0.0096	0.41	0.30	-0.11	NIN	Body	opensea
cg24750887	0.11	0.0127	0.49	0.39	-0.11	HERC3	Body	opensea
cg12354192	0.11	0.0203	0.51	0.40	-0.11	TNIK	Body	opensea
cg12161848	0.11	0.0016	0.76	0.65	-0.11	RAPGEF5	Body	opensea
cg10401362	0.11	0.0010	0.73	0.63	-0.11	DNAJB6	Body	opensea
cg03729337	0.11	0.0001	0.55	0.44	-0.11	PRDM6	Body	shore
cg22655696	0.11	0.0012	0.20	0.09	-0.11	EBF3	Body	island
cg05578480	0.11	0.0024	0.70	0.60	-0.11	FAM89A	Body	shore
cg01837362	0.11	0.0152	0.30	0.19	-0.11		IGR	shore
cg01185754	0.11	0.0019	0.50	0.39	-0.11	F3	Body	shore
cg04388989	0.11	0.0453	0.35	0.24	-0.11		IGR	island
cg05914150	0.11	0.0397	0.49	0.38	-0.11	PIK3R1	TSS200	opensea
cg25513433	0.11	0.0001	0.50	0.39	-0.11	SBF2	TSS1500	shore
cg27338607	0.10	0.0019	0.35	0.24	-0.10		IGR	shore
cg00603371	0.10	0.0061	0.27	0.16	-0.10	EMX2	TSS1500	shore
cg09491120	0.10	0.0047	0.69	0.58	-0.10	FOXF2	Body	shore
cg06220112	0.10	0.0184	0.55	0.44	-0.10		IGR	opensea
cg06360465	0.10	0.0207	0.48	0.37	-0.10	HYAL1	TSS200	opensea

cg05066959	0.10	0.0112	0.80	0.69	-0.10	ANK1	Body	opensea
cg15882591	0.10	0.0203	0.54	0.44	-0.10	LATS2	Body	opensea
cg14027333	0.10	0.0006	0.44	0.33	-0.10	PRRT1	3'UTR	shore
cg01114405	0.10	0.0166	0.79	0.68	-0.10	EXOC2	Body	opensea
cg22251569	0.10	0.0008	0.44	0.34	-0.10		IGR	shore
cg09703114	0.10	0.0119	0.64	0.54	-0.10	EP400	Body	shore
cg17120578	0.10	0.0230	0.51	0.40	-0.10		IGR	shelf
cg00179374	0.10	0.0000	0.76	0.66	-0.10	PLXNA4	5'UTR	shore
cg23500537	0.10	0.0219	0.44	0.33	-0.10		IGR	opensea
cg12881854	0.10	0.0065	0.41	0.31	-0.10		IGR	shelf
cg05569742	0.10	0.0022	0.29	0.18	-0.10	PRDM6	Body	shore
cg18583931	0.10	0.0005	0.81	0.70	-0.10		IGR	opensea
cg06620723	0.10	0.0292	0.41	0.30	-0.10	VPS13D	Body	opensea
cg12386721	0.10	0.0223	0.38	0.28	-0.10	SAPS3	5'UTR	opensea
cg14757228	0.10	0.0069	0.37	0.27	-0.10	PRRT1	3'UTR	island
cg13220123	0.10	0.0001	0.71	0.60	-0.10		IGR	island
cg24806326	0.10	0.0138	0.58	0.48	-0.10	PLCD3	Body	shore
cg01799521	0.10	0.0234	0.55	0.45	-0.10	RRAGC	3'UTR	opensea
cg22867629	0.10	0.0007	0.66	0.56	-0.10	BDKRB2	5'UTR	opensea
cg00748640	0.10	0.0007	0.89	0.78	-0.10	GPRC5B	5'UTR	opensea
cg06656005	0.10	0.0102	0.65	0.55	-0.10	NUAK1	TSS1500	shore
cg13213165	0.10	0.0011	0.82	0.72	-0.10	SGIP1	Body	opensea
cg01255894	0.10	0.0006	0.38	0.27	-0.10	GATA3	Body	shore
cg19186105	0.10	0.0174	0.28	0.18	-0.10		IGR	shelf
cg26396815	0.10	0.0000	0.80	0.70	-0.10	BANK1	Body	opensea
cg02861260	0.10	0.0018	0.68	0.58	-0.10		IGR	opensea
cg18085683	0.10	0.0028	0.81	0.71	-0.10		IGR	opensea
cg02143988	0.10	0.0144	0.72	0.62	-0.10		IGR	opensea
cg16412772	0.10	0.0013	0.72	0.62	-0.10		IGR	island
cg06881898	0.10	0.0134	0.46	0.36	-0.10	F3	Body	shore
cg21887023	0.10	0.0004	0.49	0.38	-0.10		IGR	opensea
cg04075986	0.10	0.0059	0.44	0.34	-0.10		IGR	shore
cg01912040	0.10	0.0067	0.48	0.38	-0.10		IGR	shore
cg12451671	0.10	0.0049	0.58	0.47	-0.10	ATXN1	3'UTR	opensea
cg12080306	0.10	0.0000	0.60	0.50	-0.10	YPEL2	TSS1500	shore
cg03476007	0.10	0.0055	0.58	0.47	-0.10	INPP4B	TSS1500	shore
cg27069285	0.10	0.0051	0.70	0.60	-0.10	GIT2	Body	opensea
cg07684068	0.10	0.0013	0.83	0.72	-0.10	HIF3A	Body	shore
cg23596123	0.10	0.0077	0.37	0.27	-0.10	PCDHB6	1stExon	shore
cg01815090	0.10	0.0416	0.55	0.45	-0.10	MB	TSS1500	opensea
cg18433146	0.10	0.0252	0.67	0.57	-0.10	CD36	1stExon	opensea
cg05845376	0.10	0.0267	0.29	0.19	-0.10	SLC25A2	TSS200	island
cg09271709	0.10	0.0108	0.75	0.65	-0.10	NRXN1	Body	opensea

cg13392957	0.10	0.0031	0.36	0.26	-0.10		IGR	island
cg01301252	0.10	0.0014	0.71	0.61	-0.10	PCDHGA2	Body	shore
cg00133624	0.10	0.0017	0.40	0.30	-0.10	EFCAB4B	Body	opensea
cg04304338	0.10	0.0005	0.72	0.62	-0.10	ANKRD33B	Body	opensea
cg16512163	0.10	0.0115	0.78	0.68	-0.10	RASA3	Body	opensea
cg25140419	0.10	0.0013	0.41	0.31	-0.10		IGR	island
cg18405631	0.10	0.0060	0.65	0.55	-0.10	TNIK	Body	opensea
cg05426299	0.10	0.0056	0.73	0.63	-0.10	C6orf195	TSS1500	shore
cg08234418	0.10	0.0011	0.79	0.68	-0.10	SFMBT2	Body	opensea
cg17955729	0.10	0.0014	0.46	0.36	-0.10	PRRT1	Body	island
cg25495534	0.10	0.0141	0.49	0.39	-0.10		IGR	opensea
cg02417084	0.10	0.0019	0.71	0.61	-0.10	SALL1	Body	shore
cg09782912	0.10	0.0010	0.45	0.35	-0.10		IGR	shore
cg15061330	0.10	0.0444	0.51	0.41	-0.10		IGR	opensea
cg11948367	0.10	0.0109	0.62	0.52	-0.10		IGR	opensea
cg21059392	0.10	0.0019	0.52	0.42	-0.10		IGR	shore
cg13288164	0.10	0.0421	0.38	0.28	-0.10	PCDHB4	TSS200	shore
cg03924115	0.10	0.0012	0.79	0.69	-0.10	QRFP	1stExon	opensea
cg04231085	0.10	0.0029	0.82	0.72	-0.10		IGR	opensea
cg00551647	0.10	0.0002	0.72	0.62	-0.10	CYYR1	Body	shore
cg08125574	0.10	0.0416	0.56	0.46	-0.10		IGR	opensea
cg07741162	0.10	0.0012	0.47	0.36	-0.10	PRDM6	Body	shore
cg11011736	0.10	0.0399	0.59	0.49	-0.10	AP2B1	Body	opensea
cg22122862	0.10	0.0298	0.42	0.32	-0.10	PRDM16	Body	island
cg15948785	0.10	0.0139	0.80	0.70	-0.10	PTPRN2	Body	opensea
cg26769984	0.10	0.0108	0.83	0.73	-0.10	C7orf50	Body	island
cg01044293	0.10	0.0002	0.44	0.34	-0.10	ITGA6	Body	shelf
cg11713064	0.10	0.0222	0.37	0.27	-0.10		IGR	opensea
cg04105726	0.10	0.0255	0.72	0.62	-0.10	LOC728661	Body	island
cg02564291	0.10	0.0021	0.45	0.35	-0.10	PRDM6	Body	shore
cg20505728	0.10	0.0177	0.58	0.48	-0.10		IGR	opensea
cg27225309	0.10	0.0082	0.38	0.28	-0.10	IKZF2	Body	opensea
cg13916117	0.10	0.0031	0.68	0.58	-0.10	CNTN4	Body	opensea
cg20000805	0.10	0.0003	0.56	0.46	-0.10		IGR	opensea
cg07958502	-0.10	0.0262	0.59	0.69	0.10	ITGA11	Body	opensea
cg23662097	-0.10	0.0328	0.44	0.54	0.10	ITPR1	Body	opensea
cg23173402	-0.10	0.0116	0.41	0.51	0.10		IGR	opensea
cg16085649	-0.10	0.0169	0.31	0.41	0.10	AKAP13	Body	opensea
cg08189198	-0.10	0.0066	0.47	0.57	0.10	NIN	5'UTR	opensea
cg03665360	-0.10	0.0030	0.21	0.31	0.10		IGR	opensea
cg25324164	-0.10	0.0139	0.33	0.43	0.10	FADS2	Body	shore
cg03078486	-0.10	0.0218	0.54	0.64	0.10		IGR	island
cg17452301	-0.10	0.0027	0.28	0.38	0.10	PWWP2B	Body	shore

cg00464046	-0.10	0.0095	0.59	0.69	0.10	C7orf49	Body	shore
cg18942579	-0.10	0.0093	0.20	0.30	0.10	TMEM49	Body	opensea
cg14479377	-0.10	0.0047	0.49	0.59	0.10	CAMSAP1	Body	opensea
cg01561304	-0.10	0.0252	0.34	0.44	0.10	COL23A1	Body	opensea
cg14527262	-0.10	0.0099	0.76	0.86	0.10	LOC100130872-SPON2	TSS200	shore
cg16566943	-0.10	0.0080	0.69	0.79	0.10	MUC4	Body	opensea
cg14502847	-0.10	0.0035	0.72	0.82	0.10	ADAMTSL1	Body	opensea
cg09929879	-0.10	0.0155	0.59	0.69	0.10	DIP2C	Body	shelf
cg10947408	-0.10	0.0071	0.23	0.33	0.10		IGR	island
cg03511628	-0.10	0.0016	0.64	0.74	0.10	NFATC1	Body	shore
cg27549208	-0.10	0.0316	0.49	0.59	0.10	URGCP	5'UTR	shore
cg22760287	-0.10	0.0320	0.67	0.77	0.10		IGR	opensea
cg16364066	-0.10	0.0021	0.58	0.68	0.10		IGR	opensea
cg17871621	-0.10	0.0134	0.20	0.30	0.10		IGR	island
cg18700744	-0.10	0.0257	0.30	0.40	0.10	NAA25	Body	opensea
cg14380217	-0.10	0.0056	0.53	0.63	0.10		IGR	opensea
cg14709479	-0.10	0.0088	0.21	0.31	0.10	TTLL10	TSS1500	shore
cg02146383	-0.10	0.0034	0.71	0.81	0.10	C16orf52	Body	opensea
cg10574006	-0.10	0.0072	0.62	0.72	0.10		IGR	opensea
cg24427850	-0.10	0.0240	0.59	0.69	0.10		IGR	island
cg27171704	-0.10	0.0064	0.65	0.75	0.10	TBKBP1	Body	shore
cg22816091	-0.10	0.0081	0.37	0.47	0.10		IGR	island
cg24597774	-0.10	0.0058	0.70	0.80	0.10	SPSB1	5'UTR	opensea
cg21818807	-0.10	0.0022	0.72	0.82	0.10	RPTOR	Body	shore
cg23261319	-0.10	0.0416	0.65	0.75	0.10		IGR	opensea
cg09449012	-0.10	0.0016	0.80	0.90	0.10		IGR	opensea
cg03926751	-0.10	0.0441	0.65	0.75	0.10	KLHL8	5'UTR	shore
cg14280382	-0.10	0.0063	0.75	0.85	0.10	NTN4	Body	opensea
cg01433955	-0.10	0.0074	0.70	0.80	0.10		IGR	shore
cg27312798	-0.10	0.0122	0.69	0.79	0.10	ADSSL1	Body	opensea
cg25053413	-0.10	0.0027	0.18	0.28	0.10	C14orf182	Body	shelf
cg15332978	-0.10	0.0011	0.51	0.61	0.10	C11orf24	5'UTR	shore
cg25579180	-0.10	0.0223	0.08	0.18	0.10	WBSCR17	Body	opensea
cg24475171	-0.10	0.0240	0.45	0.55	0.10	C9orf78	TSS1500	shore
cg23482132	-0.10	0.0136	0.81	0.91	0.10	FMNL2	Body	opensea
cg22214349	-0.10	0.0104	0.45	0.55	0.10		IGR	opensea
cg10397932	-0.10	0.0363	0.35	0.46	0.10	SKI	Body	opensea
cg06050964	-0.10	0.0099	0.68	0.78	0.10		IGR	opensea
cg14713217	-0.10	0.0020	0.21	0.31	0.10		IGR	opensea
cg26245202	-0.10	0.0222	0.73	0.83	0.10	ELK4	TSS1500	shore
cg12461141	-0.10	0.0106	0.63	0.73	0.10	TRIM22	TSS1500	opensea
cg19900989	-0.10	0.0420	0.57	0.67	0.10	SETBP1	Body	shelf

cg14606478	-0.10	0.0032	0.68	0.78	0.10	TRIM26	5'UTR	opensea
cg19909658	-0.10	0.0228	0.53	0.63	0.10	CDC6	Body	shelf
cg23091302	-0.10	0.0119	0.48	0.59	0.10	KCNH8	Body	opensea
cg03929570	-0.10	0.0208	0.45	0.56	0.10		IGR	shelf
cg21399203	-0.10	0.0144	0.80	0.90	0.10		IGR	opensea
cg07181702	-0.10	0.0076	0.43	0.53	0.10	MIR21	Body	opensea
cg08206092	-0.10	0.0135	0.66	0.76	0.10	SYT17	Body	opensea
cg18577239	-0.10	0.0080	0.79	0.89	0.10	ATF7	Body	opensea
cg18907942	-0.10	0.0139	0.75	0.85	0.10		IGR	opensea
cg08748308	-0.10	0.0256	0.52	0.62	0.10	LCP2	Body	opensea
cg19319037	-0.10	0.0216	0.75	0.86	0.10	TTF2	Body	shore
cg17047222	-0.10	0.0005	0.70	0.80	0.10	GIGYF1	3'UTR	shelf
cg01862311	-0.10	0.0096	0.64	0.75	0.10		IGR	shore
cg23014871	-0.10	0.0302	0.74	0.84	0.10	CSGALNACT2	Body	opensea
cg10736330	-0.10	0.0012	0.73	0.83	0.10	GPR183	TSS1500	opensea
cg27262041	-0.10	0.0037	0.36	0.46	0.10	NAV2	Body	opensea
cg25642955	-0.10	0.0168	0.58	0.68	0.10		IGR	opensea
cg15972148	-0.10	0.0135	0.70	0.80	0.10	RASA3	Body	shore
cg01135546	-0.10	0.0187	0.29	0.39	0.10		IGR	island
cg06737942	-0.10	0.0038	0.72	0.82	0.10	ESYT2	Body	opensea
cg13503148	-0.10	0.0269	0.56	0.66	0.10	TMEM212	TSS200	opensea
cg26549084	-0.10	0.0023	0.68	0.79	0.10	FNDC3B	5'UTR	shore
cg15989892	-0.10	0.0043	0.53	0.63	0.10	APBB2	5'UTR	opensea
cg08133496	-0.10	0.0001	0.34	0.44	0.10	EXT2	TSS200	shore
cg06162751	-0.10	0.0003	0.55	0.66	0.10		IGR	opensea
cg19277884	-0.10	0.0037	0.70	0.80	0.10	SPATA2	5'UTR	shore
cg02968606	-0.10	0.0070	0.76	0.87	0.10	BRMS1L	Body	shelf
cg24050474	-0.10	0.0027	0.58	0.68	0.10	MOBP	Body	shore
cg06160973	-0.10	0.0048	0.27	0.37	0.10	LRP1	1stExon	shore
cg05893709	-0.10	0.0468	0.58	0.68	0.10	IDO2	Body	opensea
cg19628988	-0.10	0.0071	0.33	0.43	0.10	CXXC5	5'UTR	island
cg17714703	-0.10	0.0256	0.37	0.47	0.10	UHRF1	Body	shore
cg14659930	-0.10	0.0140	0.43	0.53	0.10	ZBTB20	5'UTR	opensea
cg19835796	-0.10	0.0053	0.61	0.71	0.10		IGR	opensea
cg22110158	-0.10	0.0123	0.55	0.65	0.10	ST14	Body	opensea
cg20559217	-0.10	0.0012	0.31	0.42	0.10	DCPS	TSS200	shore
cg18581950	-0.10	0.0011	0.24	0.34	0.10	GPBAR1	TSS1500	opensea
cg01356872	-0.10	0.0227	0.74	0.84	0.10		IGR	opensea
cg15226275	-0.10	0.0073	0.41	0.51	0.10	FRK	TSS200	opensea
cg06234051	-0.10	0.0049	0.55	0.65	0.10	SOX9	3'UTR	shore
cg01700683	-0.10	0.0023	0.35	0.46	0.10	RAB8B	Body	opensea
cg22699314	-0.10	0.0082	0.64	0.74	0.10		IGR	opensea
cg22050733	-0.10	0.0403	0.14	0.24	0.10	CROCC	Body	shore

cg10442157	-0.10	0.0189	0.28	0.39	0.10	SYT7	3'UTR	island
cg07067189	-0.10	0.0053	0.43	0.54	0.10		IGR	opensea
cg13828068	-0.10	0.0013	0.82	0.92	0.10	RMI1	5'UTR	opensea
cg19335412	-0.10	0.0033	0.59	0.69	0.10	ACTA2	3'UTR	opensea
cg05124918	-0.10	0.0205	0.57	0.68	0.10		IGR	island
cg12999120	-0.10	0.0013	0.74	0.85	0.10		IGR	opensea
cg11560600	-0.10	0.0301	0.70	0.80	0.10		IGR	opensea
cg19962424	-0.10	0.0128	0.43	0.54	0.10		IGR	opensea
cg10817669	-0.10	0.0147	0.70	0.80	0.10		IGR	opensea
cg11645556	-0.10	0.0043	0.25	0.35	0.10	RRAS2	Body	opensea
cg24597131	-0.10	0.0011	0.72	0.82	0.10	KIAA1026	Body	opensea
cg04619120	-0.10	0.0036	0.63	0.74	0.10	ASPH	Body	opensea
cg06676119	-0.10	0.0119	0.70	0.80	0.10	PDE1A	Body	opensea
cg16962115	-0.10	0.0048	0.54	0.64	0.10	LYST	5'UTR	opensea
cg01280881	-0.10	0.0367	0.26	0.37	0.10		IGR	opensea
cg25298596	-0.10	0.0013	0.73	0.83	0.10	LUM	Body	opensea
cg26313599	-0.10	0.0499	0.67	0.78	0.10	CCKBR	Body	shore
cg20325547	-0.10	0.0082	0.64	0.74	0.10		IGR	shelf
cg08717751	-0.10	0.0159	0.75	0.86	0.10		IGR	opensea
cg16608498	-0.10	0.0095	0.38	0.49	0.10	RNF145	Body	opensea
cg15376097	-0.10	0.0007	0.15	0.25	0.10	MPZL2	TSS1500	opensea
cg21104965	-0.10	0.0007	0.37	0.47	0.10	DCBLD1	Body	island
cg05590451	-0.10	0.0031	0.41	0.51	0.10	DCUN1D3	5'UTR	opensea
cg05499012	-0.10	0.0070	0.70	0.81	0.10		IGR	opensea
cg16231917	-0.10	0.0383	0.38	0.48	0.10	PVT1	Body	opensea
cg17727795	-0.10	0.0023	0.67	0.77	0.10		IGR	opensea
cg09217157	-0.10	0.0240	0.48	0.58	0.10	ENTPD1	Body	opensea
cg10489463	-0.10	0.0196	0.70	0.80	0.10	LTBP1	Body	opensea
cg19031575	-0.10	0.0141	0.25	0.35	0.10	RTN2	5'UTR	shore
cg07450552	-0.10	0.0011	0.14	0.24	0.10	DNAJC18	TSS1500	opensea
cg09510531	-0.10	0.0468	0.31	0.41	0.10	AIRE	TSS200	island
cg27220401	-0.10	0.0005	0.68	0.78	0.10	HDAC7	Body	shore
cg04057956	-0.10	0.0426	0.53	0.63	0.10	CD9	Body	opensea
cg21829923	-0.10	0.0066	0.28	0.38	0.10	SP5	Body	island
cg09782889	-0.10	0.0063	0.13	0.23	0.10		IGR	shore
cg14706297	-0.10	0.0145	0.23	0.33	0.10		IGR	island
cg25335841	-0.10	0.0120	0.25	0.36	0.10		IGR	island
cg11230112	-0.10	0.0072	0.68	0.79	0.10	SYNJ2	Body	opensea
cg20765408	-0.10	0.0316	0.61	0.71	0.10	PARP4	5'UTR	shore
cg23019886	-0.10	0.0284	0.24	0.34	0.10		IGR	opensea
cg19591417	-0.10	0.0026	0.59	0.69	0.10		IGR	opensea
cg17092065	-0.10	0.0083	0.62	0.72	0.10	SMAD3	Body	opensea
cg05543593	-0.10	0.0033	0.56	0.67	0.10		IGR	opensea

cg18156592	-0.10	0.0335	0.32	0.43	0.10	ARL6IP5	Body	opensea
cg11231735	-0.10	0.0305	0.51	0.61	0.10		IGR	opensea
cg00296578	-0.10	0.0098	0.65	0.75	0.10	CRIM1	Body	opensea
cg20742298	-0.10	0.0117	0.73	0.83	0.10		IGR	opensea
cg14209518	-0.10	0.0194	0.48	0.59	0.10	NNMT	5'UTR	opensea
cg25542041	-0.10	0.0018	0.15	0.26	0.10	LHX6	Body	island
cg27492102	-0.10	0.0281	0.38	0.49	0.10		IGR	shore
cg21796547	-0.10	0.0410	0.30	0.41	0.10	PAPLN	5'UTR	shore
cg20788020	-0.10	0.0125	0.67	0.78	0.10		IGR	opensea
cg17552333	-0.10	0.0261	0.31	0.41	0.10		IGR	opensea
cg06654691	-0.11	0.0020	0.18	0.29	0.11	PLEKHH3	Body	shore
cg22708961	-0.11	0.0130	0.62	0.73	0.11	MORN1	Body	island
cg14872952	-0.11	0.0014	0.23	0.33	0.11		IGR	shore
cg18150247	-0.11	0.0472	0.45	0.55	0.11	ADSSL1	Body	opensea
cg24166450	-0.11	0.0104	0.34	0.45	0.11		IGR	opensea
cg05622577	-0.11	0.0182	0.66	0.77	0.11		IGR	opensea
cg25551551	-0.11	0.0038	0.78	0.89	0.11		IGR	shelf
cg03354616	-0.11	0.0334	0.54	0.65	0.11	BCKDHB	Body	opensea
cg23853861	-0.11	0.0053	0.38	0.48	0.11		IGR	shelf
cg04794141	-0.11	0.0455	0.30	0.41	0.11	TC2N	5'UTR	opensea
cg19409133	-0.11	0.0246	0.72	0.83	0.11	SNHG3-RCC1	Body	shore
cg17097293	-0.11	0.0011	0.68	0.78	0.11	PIP5K1C	Body	shelf
cg07267600	-0.11	0.0062	0.16	0.27	0.11	CACNA1C	Body	opensea
cg00598204	-0.11	0.0123	0.62	0.73	0.11	ASPSCR1	Body	shore
cg08889114	-0.11	0.0024	0.82	0.92	0.11		IGR	opensea
cg19350115	-0.11	0.0334	0.74	0.85	0.11		IGR	opensea
cg06182018	-0.11	0.0093	0.60	0.71	0.11	LIFR	5'UTR	shore
cg15082166	-0.11	0.0392	0.82	0.93	0.11	ZNF608	Body	opensea
cg01550348	-0.11	0.0141	0.70	0.81	0.11	DDAH1	Body	opensea
cg09173378	-0.11	0.0425	0.58	0.69	0.11	KCNT2	Body	opensea
cg17737409	-0.11	0.0055	0.52	0.62	0.11	GALNT2	Body	opensea
cg07025785	-0.11	0.0411	0.14	0.25	0.11		IGR	opensea
cg25467833	-0.11	0.0043	0.59	0.69	0.11		IGR	opensea
cg15985132	-0.11	0.0235	0.76	0.87	0.11		IGR	opensea
cg00377497	-0.11	0.0068	0.61	0.71	0.11	TRIM35	Body	shore
cg01357892	-0.11	0.0118	0.30	0.41	0.11	ZXDC	Body	shelf
cg02516419	-0.11	0.0102	0.34	0.44	0.11		IGR	opensea
cg16260126	-0.11	0.0113	0.45	0.56	0.11		IGR	opensea
cg14361033	-0.11	0.0018	0.23	0.34	0.11	LHX6	Body	shore
cg12973803	-0.11	0.0029	0.70	0.80	0.11		IGR	opensea
cg21112099	-0.11	0.0143	0.58	0.68	0.11	COL12A1	5'UTR	shore
cg05476182	-0.11	0.0008	0.46	0.56	0.11	PHF15	Body	opensea
cg00159243	-0.11	0.0003	0.36	0.47	0.11	SELPLG	5'UTR	opensea

cg13125510	-0.11	0.0020	0.50	0.61	0.11		IGR	opensea
cg17504394	-0.11	0.0231	0.54	0.65	0.11		IGR	opensea
cg04367486	-0.11	0.0158	0.31	0.42	0.11	CD200	1stExon	island
cg20119891	-0.11	0.0197	0.68	0.78	0.11	FIBIN	1stExon	opensea
cg21106486	-0.11	0.0257	0.09	0.20	0.11	CR1L	Body	island
cg17411016	-0.11	0.0053	0.26	0.37	0.11		IGR	opensea
cg27049539	-0.11	0.0166	0.59	0.70	0.11	USP40	Body	opensea
cg02107844	-0.11	0.0167	0.50	0.61	0.11	SLCO3A1	Body	opensea
cg16215913	-0.11	0.0032	0.28	0.39	0.11		IGR	opensea
cg18966688	-0.11	0.0129	0.61	0.72	0.11	AUTS2	Body	island
cg09862193	-0.11	0.0349	0.29	0.40	0.11		IGR	opensea
cg26147311	-0.11	0.0181	0.75	0.85	0.11		IGR	opensea
cg24116317	-0.11	0.0221	0.63	0.73	0.11	STK10	Body	opensea
cg26197915	-0.11	0.0183	0.24	0.35	0.11	PTPRJ	Body	opensea
cg03272066	-0.11	0.0151	0.56	0.66	0.11		IGR	opensea
cg16018921	-0.11	0.0019	0.74	0.85	0.11	PALLD	Body	opensea
cg13414270	-0.11	0.0026	0.43	0.54	0.11		IGR	opensea
cg15867652	-0.11	0.0297	0.63	0.74	0.11	BAIAP2	Body	shore
cg25216704	-0.11	0.0093	0.18	0.29	0.11	DOK7	Body	shore
cg08636385	-0.11	0.0292	0.55	0.66	0.11		IGR	opensea
cg19028997	-0.11	0.0218	0.72	0.82	0.11	ELK4	TSS1500	shore
cg05348875	-0.11	0.0093	0.50	0.60	0.11	NRP2	Body	opensea
cg04118124	-0.11	0.0389	0.43	0.53	0.11	LEPRE1	Body	shore
cg01178099	-0.11	0.0059	0.39	0.50	0.11		IGR	opensea
ch.11.1980478F	-0.11	0.0061	0.13	0.24	0.11	AMOTL1	Body	opensea
cg26937434	-0.11	0.0042	0.73	0.84	0.11	ANKS4B	1stExon	opensea
cg04585669	-0.11	0.0070	0.66	0.77	0.11	VGLL4	3'UTR	opensea
cg05197667	-0.11	0.0148	0.71	0.81	0.11	SRGAP1	Body	opensea
cg00633768	-0.11	0.0045	0.58	0.69	0.11	GPR133	Body	opensea
cg00731232	-0.11	0.0051	0.30	0.41	0.11		IGR	shore
cg17694130	-0.11	0.0374	0.36	0.46	0.11		IGR	opensea
cg26893134	-0.11	0.0062	0.52	0.63	0.11	FRK	1stExon	opensea
cg06933370	-0.11	0.0038	0.17	0.27	0.11	MEIS2	Body	island
cg13341982	-0.11	0.0004	0.49	0.60	0.11		IGR	opensea
cg02638523	-0.11	0.0034	0.79	0.90	0.11	AGAP1	Body	opensea
cg23670353	-0.11	0.0421	0.59	0.70	0.11	FGGY	Body	opensea
cg19758859	-0.11	0.0079	0.60	0.71	0.11	SASH1	Body	opensea
cg03950253	-0.11	0.0426	0.54	0.65	0.11	BPGM	5'UTR	opensea
cg23202253	-0.11	0.0341	0.78	0.89	0.11	ITPR2	Body	opensea
cg16497661	-0.11	0.0016	0.60	0.70	0.11	CKB	Body	shore
cg10205038	-0.11	0.0345	0.60	0.71	0.11	RIN3	Body	opensea
cg03675258	-0.11	0.0090	0.59	0.70	0.11	NFIX	Body	island
cg10995873	-0.11	0.0038	0.70	0.80	0.11	ZBTB20	5'UTR	opensea

cg14571813	-0.11	0.0302	0.37	0.48	0.11		IGR	island
cg20274304	-0.11	0.0006	0.64	0.75	0.11	PNOC	5'UTR	opensea
cg26361533	-0.11	0.0072	0.20	0.31	0.11	CACNA1C	Body	opensea
cg06740142	-0.11	0.0051	0.62	0.73	0.11	NGFR	Body	shore
cg17349632	-0.11	0.0166	0.74	0.85	0.11	PLXNA4	Body	opensea
cg05567440	-0.11	0.0078	0.64	0.75	0.11		IGR	opensea
cg11493661	-0.11	0.0008	0.25	0.35	0.11	TNXB	Body	opensea
cg10888281	-0.11	0.0010	0.43	0.54	0.11	CEP68	Body	opensea
cg23793686	-0.11	0.0138	0.65	0.76	0.11		IGR	shore
cg15572907	-0.11	0.0219	0.38	0.49	0.11	SPTBN4	Body	island
cg13153708	-0.11	0.0179	0.59	0.70	0.11		IGR	opensea
cg01031032	-0.11	0.0438	0.27	0.38	0.11	CD200	TSS200	island
cg19321696	-0.11	0.0027	0.55	0.66	0.11	NPS	3'UTR	opensea
cg07041720	-0.11	0.0001	0.61	0.72	0.11	LFNG	Body	shore
cg06938601	-0.11	0.0003	0.54	0.65	0.11	TCERG1L	Body	opensea
cg23199907	-0.11	0.0158	0.67	0.78	0.11	PDS5B	Body	opensea
cg08305942	-0.11	0.0484	0.77	0.88	0.11		IGR	opensea
cg06871529	-0.11	0.0094	0.60	0.71	0.11	FRY	Body	opensea
cg21394171	-0.11	0.0093	0.35	0.46	0.11		IGR	opensea
cg18418335	-0.11	0.0271	0.19	0.30	0.11	MKNK2	Body	island
cg17516247	-0.11	0.0195	0.60	0.71	0.11		IGR	opensea
cg04899656	-0.11	0.0243	0.70	0.81	0.11		IGR	opensea
cg16385330	-0.11	0.0185	0.63	0.74	0.11	LRBA	Body	shore
cg18751133	-0.11	0.0017	0.67	0.77	0.11	COQ3	TSS1500	shore
cg13627062	-0.11	0.0048	0.16	0.27	0.11		IGR	shore
cg07964219	-0.11	0.0244	0.41	0.52	0.11	COL18A1	Body	shore
ch.14.1488981R	-0.11	0.0033	0.39	0.50	0.11	RIN3	Body	opensea
cg20493497	-0.11	0.0142	0.31	0.42	0.11		IGR	opensea
cg02637282	-0.11	0.0406	0.56	0.67	0.11		IGR	opensea
cg26828017	-0.11	0.0046	0.79	0.90	0.11		IGR	opensea
cg00954105	-0.11	0.0140	0.67	0.77	0.11	CCDC46	Body	opensea
cg04604708	-0.11	0.0064	0.70	0.80	0.11	CREB3L2	Body	opensea
cg14455403	-0.11	0.0027	0.70	0.80	0.11	LMCD1	Body	opensea
cg24112733	-0.11	0.0107	0.14	0.25	0.11	STARD3NL	Body	opensea
cg06096901	-0.11	0.0024	0.66	0.77	0.11	RPTOR	Body	shelf
cg15945235	-0.11	0.0092	0.49	0.60	0.11	ANKRD22	1stExon	opensea
cg20454073	-0.11	0.0067	0.58	0.69	0.11	NFIX	Body	island
cg09015246	-0.11	0.0208	0.52	0.62	0.11	CIITA	1stExon	shore
cg03097134	-0.11	0.0095	0.58	0.68	0.11		IGR	opensea
cg16786808	-0.11	0.0208	0.51	0.62	0.11	EPHA4	Body	shore
cg10728351	-0.11	0.0007	0.76	0.87	0.11		IGR	opensea
cg02279108	-0.11	0.0132	0.67	0.77	0.11		IGR	shelf
cg10733123	-0.11	0.0026	0.72	0.83	0.11	RAD51L1	Body	opensea

cg20765267	-0.11	0.0031	0.13	0.23	0.11	DCAF13	Body	shore
cg04645534	-0.11	0.0075	0.32	0.43	0.11	STC1	TSS1500	opensea
cg07810677	-0.11	0.0015	0.61	0.71	0.11	COL25A1	Body	opensea
cg19774788	-0.11	0.0008	0.61	0.72	0.11	WDR51A	Body	opensea
cg02793828	-0.11	0.0017	0.30	0.41	0.11		IGR	shore
cg00288598	-0.11	0.0377	0.63	0.74	0.11	EIF2C2	Body	shelf
cg21453378	-0.11	0.0027	0.76	0.87	0.11	GAPDHS	TSS200	island
cg18842353	-0.11	0.0009	0.18	0.29	0.11		IGR	opensea
cg13531667	-0.11	0.0500	0.56	0.66	0.11	MCC	TSS1500	shore
cg14157435	-0.11	0.0241	0.30	0.41	0.11	NRP2	Body	opensea
cg04527220	-0.11	0.0067	0.49	0.60	0.11	GPR132	5'UTR	opensea
cg23654401	-0.11	0.0214	0.72	0.83	0.11	VOPP1	Body	opensea
cg02184280	-0.11	0.0103	0.18	0.29	0.11	PIH1D1	Body	shore
cg23654821	-0.11	0.0449	0.26	0.37	0.11	CSRNP1	5'UTR	shore
cg12229979	-0.11	0.0005	0.50	0.61	0.11	MYO9B	5'UTR	shelf
cg24334149	-0.11	0.0140	0.49	0.60	0.11	ATP6V1B1	Body	opensea
cg22377643	-0.11	0.0070	0.31	0.42	0.11		IGR	shore
cg16558432	-0.11	0.0080	0.25	0.36	0.11	LOC728392	1stExon	island
cg13794390	-0.11	0.0000	0.75	0.86	0.11	FOXJ3	Body	opensea
cg12235073	-0.11	0.0080	0.49	0.60	0.11	PFKFB3	Body	opensea
cg21994818	-0.11	0.0005	0.49	0.60	0.11		IGR	opensea
cg06686742	-0.11	0.0029	0.31	0.42	0.11	LASS4	TSS1500	shore
cg23093090	-0.11	0.0200	0.57	0.67	0.11	C10orf26	3'UTR	opensea
cg02153814	-0.11	0.0040	0.53	0.64	0.11		IGR	island
cg15324723	-0.11	0.0154	0.66	0.77	0.11	VPS13B	Body	opensea
cg14565465	-0.11	0.0336	0.59	0.70	0.11	TMEM2	3'UTR	opensea
cg14462670	-0.11	0.0006	0.13	0.24	0.11		IGR	shelf
cg01135464	-0.11	0.0008	0.71	0.82	0.11		IGR	opensea
cg10115368	-0.11	0.0077	0.73	0.84	0.11		IGR	shore
cg06680511	-0.11	0.0474	0.50	0.61	0.11		IGR	shore
cg19017553	-0.11	0.0083	0.68	0.79	0.11	PARD3	Body	opensea
cg19714737	-0.11	0.0302	0.55	0.66	0.11		IGR	shelf
cg10468535	-0.11	0.0058	0.70	0.81	0.11	PTPRN2	Body	opensea
cg11942594	-0.11	0.0237	0.65	0.76	0.11		IGR	opensea
cg07830160	-0.11	0.0037	0.20	0.31	0.11		IGR	opensea
cg11029367	-0.11	0.0136	0.69	0.80	0.11	HEG1	Body	opensea
cg09179646	-0.11	0.0247	0.52	0.63	0.11		IGR	island
cg07985116	-0.11	0.0188	0.52	0.63	0.11	LRP5	Body	shore
cg23375169	-0.11	0.0005	0.64	0.75	0.11		IGR	opensea
cg10134833	-0.11	0.0112	0.24	0.35	0.11		IGR	shore
cg09092280	-0.11	0.0206	0.31	0.42	0.11		IGR	opensea
cg01065822	-0.11	0.0003	0.61	0.72	0.11		IGR	opensea
cg19830657	-0.11	0.0013	0.13	0.24	0.11		IGR	island

cg07804749	-0.11	0.0245	0.62	0.73	0.11		IGR	opensea
cg20366549	-0.11	0.0028	0.20	0.31	0.11	SCNN1A	Body	opensea
cg07791418	-0.11	0.0017	0.54	0.65	0.11		IGR	opensea
cg19249516	-0.11	0.0064	0.71	0.82	0.11		IGR	opensea
cg06224587	-0.11	0.0044	0.55	0.66	0.11		IGR	opensea
cg19757382	-0.11	0.0020	0.31	0.42	0.11	MYEOV2	TSS1500	shore
cg07229001	-0.11	0.0045	0.51	0.62	0.11		IGR	opensea
cg13911697	-0.11	0.0067	0.66	0.77	0.11		IGR	opensea
ch.6.2925136R	-0.11	0.0097	0.26	0.37	0.11	MTHFD1L	Body	opensea
cg15394255	-0.11	0.0034	0.53	0.64	0.11	MGAT4C	TSS200	opensea
cg19609438	-0.11	0.0074	0.77	0.88	0.11	VPS13B	Body	opensea
cg23196346	-0.11	0.0088	0.44	0.55	0.11	KIAA1217	Body	opensea
cg14414911	-0.11	0.0025	0.51	0.62	0.11		IGR	opensea
cg10438089	-0.11	0.0082	0.24	0.35	0.11	FAM117A	Body	opensea
cg07126783	-0.11	0.0260	0.66	0.77	0.11	RPTOR	Body	shore
cg12804755	-0.11	0.0052	0.53	0.64	0.11		IGR	opensea
ch.3.3371471R	-0.11	0.0350	0.21	0.32	0.11	NLGN1	5'UTR	opensea
cg11235594	-0.11	0.0187	0.55	0.66	0.11	NPHP1	Body	opensea
cg16451306	-0.11	0.0016	0.56	0.67	0.11	MAP1B	Body	opensea
cg25139649	-0.11	0.0252	0.43	0.55	0.11	SKI	Body	opensea
cg26541218	-0.11	0.0451	0.66	0.78	0.11	PKD1L1	Body	opensea
cg16550264	-0.11	0.0009	0.31	0.42	0.11		IGR	opensea
cg22926842	-0.11	0.0075	0.60	0.71	0.11	CENPM	TSS1500	opensea
cg06055229	-0.11	0.0044	0.42	0.53	0.11	ZNF608	Body	opensea
cg14157107	-0.11	0.0137	0.57	0.68	0.11	BNC2	Body	opensea
cg25503149	-0.11	0.0220	0.11	0.22	0.11		IGR	opensea
cg26988138	-0.11	0.0189	0.46	0.57	0.11	GNG7	5'UTR	shelf
cg26806779	-0.11	0.0318	0.59	0.70	0.11	KLRG1	TSS1500	opensea
cg26180080	-0.11	0.0359	0.14	0.25	0.11		IGR	opensea
cg15922174	-0.11	0.0146	0.21	0.32	0.11	CRB2	Body	island
cg20744756	-0.11	0.0163	0.65	0.77	0.11		IGR	opensea
cg01704698	-0.11	0.0179	0.59	0.70	0.11	CRISPLD2	3'UTR	opensea
cg03987648	-0.11	0.0393	0.71	0.82	0.11	MAML3	Body	opensea
cg01292810	-0.11	0.0452	0.15	0.26	0.11		IGR	shore
cg14608275	-0.11	0.0041	0.48	0.59	0.11	NCEH1	TSS1500	shore
cg02564299	-0.11	0.0002	0.63	0.74	0.11	ZBTB20	5'UTR	opensea
cg11894504	-0.11	0.0308	0.21	0.33	0.11	MKNK2	Body	island
cg13104185	-0.11	0.0104	0.38	0.50	0.11	RERE	Body	opensea
cg19223064	-0.11	0.0105	0.67	0.79	0.11		IGR	shelf
cg11084266	-0.11	0.0012	0.34	0.45	0.11	PMF1	Body	shore
cg21074766	-0.11	0.0018	0.56	0.68	0.11		IGR	opensea
cg06160606	-0.11	0.0068	0.81	0.93	0.11	PDE4B	Body	opensea
cg11537406	-0.11	0.0114	0.43	0.54	0.11	SLC37A2	TSS1500	shore

cg03873153	-0.11	0.0131	0.28	0.39	0.11		IGR	shelf
cg08575883	-0.11	0.0171	0.71	0.82	0.11		IGR	opensea
cg16046375	-0.11	0.0443	0.43	0.54	0.11	RASA3	Body	shore
cg11916478	-0.11	0.0452	0.57	0.68	0.11	IQSEC3	Body	island
cg26668675	-0.11	0.0254	0.45	0.57	0.11		IGR	island
cg17227257	-0.11	0.0370	0.49	0.61	0.11	LOC100130872-SPON2	TSS200	shore
cg06633978	-0.11	0.0346	0.58	0.69	0.11	CFDP1	Body	opensea
cg05657292	-0.11	0.0123	0.42	0.53	0.11	CECR2	Body	opensea
cg15406952	-0.11	0.0010	0.54	0.65	0.11		IGR	opensea
ch.8.18329843R	-0.11	0.0050	0.15	0.26	0.11		IGR	opensea
cg07740894	-0.11	0.0134	0.69	0.81	0.11	CCNY	Body	opensea
cg14111334	-0.11	0.0081	0.67	0.78	0.11		IGR	opensea
cg11174786	-0.11	0.0033	0.49	0.60	0.11		IGR	opensea
cg04254487	-0.11	0.0324	0.74	0.86	0.11	TBPL1	Body	opensea
cg01323777	-0.11	0.0089	0.51	0.63	0.11	KCNAB3	TSS200	island
cg11970797	-0.11	0.0036	0.78	0.89	0.11	CRYL1	Body	opensea
cg14398228	-0.11	0.0231	0.61	0.73	0.11	VRK1	Body	opensea
cg24886867	-0.11	0.0265	0.68	0.80	0.11	RIPK1	TSS1500	opensea
cg04506114	-0.11	0.0055	0.62	0.73	0.11	SOAT1	Body	opensea
cg20968743	-0.11	0.0159	0.35	0.46	0.11	TSPAN18	5'UTR	opensea
cg27310092	-0.11	0.0065	0.34	0.46	0.11		IGR	opensea
cg08348121	-0.11	0.0022	0.64	0.75	0.11	SKIL	TSS1500	shore
cg10328047	-0.11	0.0060	0.69	0.80	0.11	PLEC1	Body	island
cg07034563	-0.11	0.0230	0.69	0.80	0.11	PDLIM7	Body	shore
cg03517250	-0.11	0.0031	0.46	0.57	0.11		IGR	shore
cg19590421	-0.11	0.0022	0.28	0.39	0.11		IGR	opensea
cg03624316	-0.11	0.0076	0.41	0.52	0.11	PFDN5	Body	shore
cg03310937	-0.11	0.0011	0.50	0.62	0.11		IGR	opensea
cg21368566	-0.11	0.0085	0.69	0.80	0.11	AGAP1	Body	opensea
ch.2.16090152F	-0.11	0.0110	0.19	0.30	0.11		IGR	opensea
cg15964672	-0.11	0.0229	0.76	0.87	0.11		IGR	opensea
cg12868173	-0.11	0.0302	0.21	0.32	0.11		IGR	island
cg05376465	-0.11	0.0319	0.12	0.23	0.11		IGR	opensea
cg01636873	-0.11	0.0036	0.52	0.63	0.11	CARKD	Body	shelf
cg23683800	-0.11	0.0018	0.15	0.26	0.11		IGR	opensea
cg03354554	-0.11	0.0161	0.33	0.44	0.11		IGR	shore
cg15972264	-0.11	0.0149	0.77	0.89	0.11	NEDD9	Body	opensea
cg19964491	-0.11	0.0000	0.19	0.30	0.11	TNXB	Body	opensea
cg13706058	-0.11	0.0000	0.44	0.56	0.11		IGR	opensea
cg01005968	-0.11	0.0316	0.24	0.35	0.11	RTN2	5'UTR	shore
cg10011495	-0.11	0.0045	0.67	0.78	0.11		IGR	shore
cg22701603	-0.11	0.0129	0.53	0.65	0.11		IGR	island

cg16429725	-0.11	0.0057	0.57	0.68	0.11	KIFC3	Body	shelf
cg25799589	-0.11	0.0048	0.52	0.64	0.11	ZNF827	Body	opensea
cg18992848	-0.11	0.0182	0.31	0.42	0.11	TPK1	Body	shore
ch.22.528917R	-0.11	0.0005	0.15	0.27	0.11	HMGXB4	Body	opensea
cg07388969	-0.11	0.0006	0.57	0.69	0.11		IGR	opensea
cg06595154	-0.11	0.0049	0.52	0.64	0.11	MRVI1	TSS1500	opensea
cg24611970	-0.11	0.0040	0.60	0.71	0.11		IGR	opensea
cg23679344	-0.11	0.0005	0.80	0.91	0.11	MED1	3'UTR	shore
cg03517776	-0.11	0.0323	0.56	0.68	0.11		IGR	shore
cg05695699	-0.12	0.0053	0.68	0.80	0.12	KIAA1522	Body	shore
cg04964617	-0.12	0.0051	0.56	0.68	0.12		IGR	opensea
cg06570967	-0.12	0.0291	0.80	0.91	0.12		IGR	opensea
cg05228359	-0.12	0.0253	0.35	0.46	0.12		IGR	opensea
cg07664000	-0.12	0.0114	0.57	0.69	0.12	TMIGD1	5'UTR	opensea
cg23733260	-0.12	0.0312	0.68	0.79	0.12	BBX	5'UTR	opensea
cg15735157	-0.12	0.0005	0.33	0.45	0.12		IGR	opensea
cg20221591	-0.12	0.0048	0.38	0.50	0.12	MYEOV2	TSS1500	shore
cg22400059	-0.12	0.0069	0.71	0.82	0.12	PEX14	Body	opensea
cg15012161	-0.12	0.0092	0.70	0.81	0.12		IGR	opensea
cg20388732	-0.12	0.0070	0.32	0.44	0.12	STAT5A	TSS200	shore
cg26870745	-0.12	0.0042	0.60	0.71	0.12		IGR	opensea
cg01238435	-0.12	0.0319	0.45	0.57	0.12		IGR	opensea
cg14520913	-0.12	0.0174	0.66	0.77	0.12	NNMT	TSS200	opensea
cg10110335	-0.12	0.0300	0.57	0.69	0.12	SYN2	Body	shelf
cg02714666	-0.12	0.0128	0.52	0.64	0.12		IGR	opensea
cg05762671	-0.12	0.0297	0.62	0.74	0.12	KCTD19	Body	opensea
cg16453056	-0.12	0.0322	0.40	0.51	0.12	UPK3B	3'UTR	island
cg19521832	-0.12	0.0163	0.17	0.28	0.12	KCNE1	TSS200	island
cg01354782	-0.12	0.0109	0.66	0.78	0.12	ITPR2	Body	opensea
cg15032314	-0.12	0.0078	0.21	0.32	0.12	PRR12	Body	island
cg15931471	-0.12	0.0108	0.54	0.66	0.12	SMAD6	Body	opensea
cg04637264	-0.12	0.0106	0.50	0.62	0.12		IGR	opensea
cg02832512	-0.12	0.0301	0.64	0.76	0.12	FLJ22536	Body	opensea
cg13636014	-0.12	0.0012	0.43	0.54	0.12		IGR	opensea
cg08888203	-0.12	0.0315	0.73	0.85	0.12	C3orf24	TSS200	island
cg06457736	-0.12	0.0359	0.55	0.66	0.12	HRH1	TSS200	opensea
cg26856257	-0.12	0.0096	0.57	0.69	0.12		IGR	shelf
cg06342072	-0.12	0.0055	0.49	0.61	0.12		IGR	opensea
cg24116380	-0.12	0.0036	0.22	0.33	0.12		IGR	opensea
cg23197236	-0.12	0.0007	0.75	0.87	0.12	LIN7C	3'UTR	opensea
cg19798881	-0.12	0.0049	0.51	0.63	0.12	BMP6	Body	opensea
cg04156483	-0.12	0.0185	0.62	0.74	0.12	MORN1	Body	island
cg19734190	-0.12	0.0062	0.64	0.76	0.12	TBC1D14	Body	opensea

cg13419330	-0.12	0.0103	0.60	0.71	0.12	IRAK2	Body	opensea
cg10729496	-0.12	0.0050	0.73	0.84	0.12	C3orf24	TSS200	island
cg04095069	-0.12	0.0035	0.69	0.81	0.12	NUB1	Body	opensea
cg18108818	-0.12	0.0036	0.62	0.74	0.12	FBXL13	Body	opensea
cg20236089	-0.12	0.0097	0.75	0.87	0.12	DTNBP1	Body	opensea
cg02598079	-0.12	0.0159	0.58	0.70	0.12		IGR	shelf
cg23780146	-0.12	0.0002	0.57	0.69	0.12		IGR	opensea
cg04456219	-0.12	0.0419	0.34	0.46	0.12		IGR	opensea
cg20899781	-0.12	0.0043	0.39	0.51	0.12		IGR	opensea
cg10974479	-0.12	0.0018	0.71	0.83	0.12	MLN	TSS1500	opensea
cg23530586	-0.12	0.0068	0.42	0.54	0.12		IGR	opensea
cg25153741	-0.12	0.0089	0.47	0.59	0.12	COL23A1	Body	opensea
cg02019444	-0.12	0.0025	0.38	0.50	0.12	ITSN1	5'UTR	shore
ch.10.2563868F	-0.12	0.0011	0.13	0.24	0.12	TIAL1	Body	opensea
cg26177041	-0.12	0.0006	0.23	0.35	0.12	CAMK2D	Body	opensea
cg25453625	-0.12	0.0077	0.41	0.53	0.12		IGR	island
cg17611046	-0.12	0.0078	0.66	0.77	0.12	FARS2	Body	opensea
cg25838968	-0.12	0.0359	0.54	0.65	0.12	PLXNA2	Body	opensea
cg14824921	-0.12	0.0105	0.48	0.60	0.12	IGFBP7	Body	opensea
cg01649611	-0.12	0.0350	0.59	0.71	0.12	THADA	Body	opensea
cg06189394	-0.12	0.0064	0.50	0.62	0.12	LRIG1	Body	opensea
cg13221458	-0.12	0.0000	0.17	0.29	0.12	SOD2	Body	shore
cg25913882	-0.12	0.0166	0.64	0.76	0.12	CUBN	Body	opensea
cg16476991	-0.12	0.0061	0.50	0.62	0.12	RASA3	Body	opensea
cg18045100	-0.12	0.0012	0.37	0.49	0.12		IGR	opensea
cg13544946	-0.12	0.0053	0.39	0.50	0.12	TLN1	5'UTR	shelf
cg15723028	-0.12	0.0192	0.58	0.70	0.12	RIPK2	Body	opensea
cg13780718	-0.12	0.0009	0.47	0.59	0.12	ZSWIM1	3'UTR	opensea
cg24587080	-0.12	0.0093	0.65	0.77	0.12		IGR	opensea
cg10763234	-0.12	0.0246	0.26	0.38	0.12		IGR	island
cg02196592	-0.12	0.0007	0.51	0.63	0.12		IGR	opensea
cg08607018	-0.12	0.0040	0.29	0.41	0.12		IGR	island
cg00993830	-0.12	0.0050	0.41	0.53	0.12	UBE2H	Body	opensea
cg01265860	-0.12	0.0041	0.67	0.79	0.12	RUNX1	Body	shelf
cg01119374	-0.12	0.0024	0.68	0.80	0.12		IGR	shore
cg26177311	-0.12	0.0082	0.72	0.84	0.12	PEMT	Body	opensea
cg25104233	-0.12	0.0142	0.56	0.68	0.12		IGR	opensea
cg10732611	-0.12	0.0010	0.19	0.31	0.12	STARD13	Body	opensea
cg02952295	-0.12	0.0315	0.66	0.78	0.12	TRPM8	Body	opensea
cg13332142	-0.12	0.0001	0.70	0.81	0.12		IGR	opensea
cg02681842	-0.12	0.0044	0.61	0.73	0.12	PLEC1	Body	opensea
cg18710053	-0.12	0.0261	0.24	0.36	0.12	FLJ44606	5'UTR	island
cg06685282	-0.12	0.0022	0.12	0.24	0.12		IGR	opensea

cg26828599	-0.12	0.0129	0.73	0.85	0.12	TPCN2	Body	opensea
cg14200368	-0.12	0.0011	0.65	0.77	0.12	THSD7A	Body	opensea
ch.2.11889418R	-0.12	0.0030	0.42	0.54	0.12		IGR	opensea
cg10423842	-0.12	0.0106	0.58	0.70	0.12		IGR	opensea
cg01199603	-0.12	0.0009	0.77	0.89	0.12	MSRB3	Body	opensea
cg03687532	-0.12	0.0042	0.43	0.55	0.12		IGR	island
cg09782560	-0.12	0.0124	0.20	0.32	0.12		IGR	opensea
cg07008591	-0.12	0.0219	0.70	0.82	0.12	TEAD1	Body	opensea
cg10840277	-0.12	0.0148	0.58	0.70	0.12		IGR	opensea
cg16377679	-0.12	0.0016	0.45	0.57	0.12	PDE4D	Body	opensea
cg26314722	-0.12	0.0145	0.67	0.79	0.12		IGR	opensea
ch.10.2760753F	-0.12	0.0033	0.16	0.28	0.12		IGR	shore
cg09807875	-0.12	0.0025	0.43	0.55	0.12	BMPER	Body	opensea
cg08913523	-0.12	0.0078	0.48	0.60	0.12		IGR	opensea
cg04703620	-0.12	0.0004	0.59	0.71	0.12		IGR	opensea
cg23462772	-0.12	0.0154	0.51	0.63	0.12		IGR	opensea
cg02847220	-0.12	0.0066	0.59	0.71	0.12	PLD1	5'UTR	opensea
cg25616869	-0.12	0.0158	0.57	0.70	0.12		IGR	opensea
cg07059402	-0.12	0.0055	0.35	0.47	0.12	MIR659	TSS1500	shore
cg22905511	-0.12	0.0345	0.16	0.28	0.12	FLJ44606	TSS200	island
cg20658466	-0.12	0.0014	0.36	0.48	0.12		IGR	opensea
cg18621852	-0.12	0.0279	0.63	0.75	0.12	C3orf24	TSS200	island
cg15418499	-0.12	0.0102	0.45	0.57	0.12	IL18	5'UTR	opensea
cg19048331	-0.12	0.0004	0.56	0.69	0.12	BTBD3	5'UTR	opensea
cg22631616	-0.12	0.0050	0.69	0.82	0.12	TBC1D9	Body	opensea
cg12559197	-0.12	0.0089	0.42	0.54	0.12	PDE8B	Body	opensea
cg22319784	-0.12	0.0069	0.32	0.44	0.12	BTNL9	3'UTR	island
cg07201475	-0.12	0.0217	0.68	0.80	0.12		IGR	opensea
cg03628053	-0.12	0.0082	0.65	0.77	0.12	FBN1	Body	shelf
cg25954269	-0.12	0.0292	0.68	0.80	0.12		IGR	opensea
cg06786153	-0.12	0.0263	0.33	0.45	0.12	TBC1D2B	Body	opensea
cg22550815	-0.12	0.0342	0.62	0.75	0.12	KANK2	3'UTR	opensea
cg02750792	-0.12	0.0024	0.22	0.34	0.12	MOBP	Body	island
cg06405186	-0.12	0.0072	0.41	0.53	0.12	SYT7	3'UTR	island
cg08382534	-0.12	0.0014	0.77	0.90	0.12	SLC38A8	Body	opensea
cg02849956	-0.12	0.0098	0.60	0.72	0.12		IGR	shore
cg05876246	-0.12	0.0206	0.30	0.42	0.12		IGR	opensea
cg09966895	-0.12	0.0292	0.71	0.83	0.12	ODZ4	Body	opensea
cg06159404	-0.12	0.0258	0.20	0.32	0.12		IGR	island
cg00858840	-0.12	0.0139	0.23	0.35	0.12	SP5	Body	island
cg03001305	-0.12	0.0034	0.43	0.55	0.12	STAT5A	TSS200	shore
cg15645888	-0.12	0.0004	0.63	0.75	0.12	FBXO16	3'UTR	opensea

cg04228083	-0.12	0.0113	0.69	0.81	0.12	LOC100130872-SPON2	TSS200	shore
cg13088471	-0.12	0.0026	0.49	0.62	0.12	ASAP1	Body	opensea
cg15174393	-0.12	0.0187	0.37	0.49	0.12		IGR	opensea
cg13173567	-0.12	0.0085	0.25	0.37	0.12	DENND5A	Body	opensea
cg02784232	-0.12	0.0002	0.59	0.72	0.12	PHC3	Body	shore
cg04944393	-0.12	0.0011	0.80	0.92	0.12	ATP6V1E2	TSS1500	opensea
cg24671734	-0.12	0.0110	0.44	0.56	0.12	BTBD11	Body	opensea
cg10574566	-0.12	0.0035	0.20	0.33	0.12		IGR	opensea
cg01284595	-0.12	0.0001	0.59	0.72	0.12	ZFAND3	Body	opensea
cg21274067	-0.12	0.0396	0.27	0.39	0.12	WASH2P	TSS200	shore
cg06474225	-0.12	0.0002	0.26	0.38	0.12	HTRA1	Body	opensea
cg12939390	-0.12	0.0023	0.62	0.75	0.12	SRBD1	Body	opensea
cg12427162	-0.12	0.0031	0.59	0.72	0.12	SFT2D2	Body	shelf
cg21144009	-0.12	0.0372	0.41	0.54	0.12	PLXNA2	Body	opensea
cg20740711	-0.12	0.0158	0.40	0.52	0.12		IGR	opensea
cg00741954	-0.12	0.0022	0.55	0.67	0.12	GMDS	Body	opensea
cg06018119	-0.12	0.0071	0.38	0.50	0.12	MAPK11	Body	shore
cg13490403	-0.12	0.0014	0.17	0.30	0.12	LHX6	Body	island
cg15633390	-0.12	0.0186	0.42	0.54	0.12	EIF4E	5'UTR	shore
cg18919017	-0.12	0.0006	0.47	0.59	0.12	DPYSL3	Body	opensea
cg20979153	-0.12	0.0099	0.38	0.50	0.12	ZNF217	TSS200	shore
cg24736734	-0.12	0.0076	0.29	0.42	0.12		IGR	opensea
cg02246055	-0.12	0.0468	0.35	0.48	0.12	ZFHX3	Body	island
cg02524983	-0.13	0.0030	0.35	0.48	0.13	LPP	5'UTR	opensea
cg20073153	-0.13	0.0181	0.60	0.72	0.13		IGR	opensea
cg23732024	-0.13	0.0076	0.60	0.72	0.13	LY96	Body	opensea
cg14753094	-0.13	0.0101	0.72	0.84	0.13	HSD17B12	Body	opensea
cg15443403	-0.13	0.0017	0.15	0.27	0.13	THUMPD3	TSS1500	shore
cg10764891	-0.13	0.0083	0.70	0.83	0.13		IGR	shelf
cg02304092	-0.13	0.0207	0.16	0.29	0.13	FLJ44606	TSS200	island
cg16638092	-0.13	0.0210	0.68	0.80	0.13	RPTOR	Body	shore
cg00311883	-0.13	0.0317	0.40	0.52	0.13		IGR	opensea
cg21369466	-0.13	0.0038	0.68	0.81	0.13	LANCL2	Body	shelf
cg04128967	-0.13	0.0005	0.61	0.74	0.13		IGR	opensea
cg27638217	-0.13	0.0147	0.40	0.52	0.13		IGR	shore
cg07493197	-0.13	0.0087	0.40	0.52	0.13		IGR	opensea
cg02683114	-0.13	0.0022	0.71	0.83	0.13	C2orf84	Body	island
cg08651590	-0.13	0.0036	0.65	0.78	0.13	SPRED2	Body	shelf
cg25123566	-0.13	0.0171	0.71	0.84	0.13	FAM113B	5'UTR	opensea
cg17800426	-0.13	0.0018	0.70	0.83	0.13	MYOZ3	TSS1500	shelf
cg08312765	-0.13	0.0007	0.43	0.56	0.13		IGR	opensea
cg09435170	-0.13	0.0046	0.17	0.30	0.13		IGR	opensea

cg24680632	-0.13	0.0028	0.39	0.52	0.13		IGR	opensea
cg27549186	-0.13	0.0213	0.68	0.81	0.13	TIMP2	Body	opensea
cg16763089	-0.13	0.0400	0.25	0.37	0.13	LOC149837	TSS200	opensea
cg01116477	-0.13	0.0004	0.62	0.75	0.13		IGR	opensea
cg11802797	-0.13	0.0002	0.15	0.28	0.13	KIF1B	3'UTR	opensea
cg07600533	-0.13	0.0067	0.21	0.34	0.13	KLHDC7B	TSS1500	island
cg11947187	-0.13	0.0067	0.26	0.39	0.13	TG	Body	opensea
cg09450153	-0.13	0.0062	0.59	0.72	0.13	CREB5	Body	opensea
cg14553740	-0.13	0.0381	0.45	0.58	0.13	FAM154A	Body	opensea
cg01412419	-0.13	0.0386	0.63	0.76	0.13		IGR	opensea
cg26575450	-0.13	0.0164	0.55	0.68	0.13		IGR	shore
cg04482110	-0.13	0.0284	0.11	0.24	0.13	TMEM106A	5'UTR	island
cg15244778	-0.13	0.0018	0.74	0.87	0.13		IGR	opensea
cg00960147	-0.13	0.0112	0.71	0.84	0.13		IGR	opensea
cg26094842	-0.13	0.0132	0.74	0.87	0.13	VTI1A	Body	opensea
cg09728393	-0.13	0.0085	0.64	0.77	0.13	KIF13A	Body	opensea
cg01589587	-0.13	0.0003	0.30	0.43	0.13	BATF	Body	opensea
cg10907148	-0.13	0.0004	0.54	0.67	0.13	C17orf28	Body	opensea
cg09251959	-0.13	0.0325	0.42	0.55	0.13	COL16A1	TSS1500	shore
cg06691616	-0.13	0.0149	0.54	0.67	0.13		IGR	opensea
cg25538571	-0.13	0.0084	0.60	0.73	0.13		IGR	opensea
cg19089701	-0.13	0.0177	0.46	0.59	0.13		IGR	opensea
cg07219303	-0.13	0.0343	0.49	0.62	0.13	ADH6	TSS1500	opensea
cg03052078	-0.13	0.0011	0.52	0.65	0.13	STXBP5	Body	shore
cg14977018	-0.13	0.0018	0.75	0.89	0.13	TMEM44	Body	opensea
cg21664909	-0.13	0.0005	0.55	0.68	0.13	PXDN	Body	opensea
cg01466164	-0.13	0.0008	0.44	0.57	0.13		IGR	shore
cg01351822	-0.13	0.0065	0.05	0.18	0.13	UNC45A	5'UTR	island
cg19784903	-0.13	0.0063	0.59	0.72	0.13	TBKBP1	Body	island
cg26300461	-0.13	0.0008	0.48	0.61	0.13		IGR	shore
cg27520536	-0.13	0.0132	0.52	0.65	0.13		IGR	island
cg17660833	-0.13	0.0277	0.51	0.64	0.13	HRH1	5'UTR	opensea
cg13051700	-0.13	0.0002	0.50	0.63	0.13	MAGI2	Body	opensea
cg05963604	-0.13	0.0105	0.36	0.49	0.13	APPL1	Body	shore
cg00223245	-0.13	0.0014	0.44	0.57	0.13		IGR	opensea
cg05492387	-0.13	0.0141	0.60	0.73	0.13	RAP1GDS1	Body	opensea
cg09530108	-0.13	0.0068	0.68	0.81	0.13	CRIM1	Body	shore
cg11811828	-0.13	0.0375	0.53	0.66	0.13		IGR	shore
cg05227773	-0.13	0.0054	0.64	0.78	0.13	ZFHX3	3'UTR	shelf
cg19229692	-0.13	0.0026	0.75	0.89	0.13	PLXNA4	Body	opensea
cg18700940	-0.13	0.0019	0.16	0.30	0.13	MAP3K14	5'UTR	opensea
cg04642300	-0.13	0.0034	0.45	0.58	0.13	ARMC2	Body	opensea
cg15852787	-0.13	0.0032	0.70	0.84	0.13	FRMD6	5'UTR	opensea

cg26877720	-0.13	0.0046	0.64	0.77	0.13	FAM107B	Body	shore
cg04164584	-0.13	0.0056	0.63	0.76	0.13	PHF12	Body	opensea
cg14276379	-0.13	0.0266	0.29	0.42	0.13	C9orf3	Body	opensea
cg13805761	-0.14	0.0017	0.49	0.63	0.14	DCLK1	Body	opensea
cg11868461	-0.14	0.0211	0.40	0.54	0.14		IGR	opensea
cg16911981	-0.14	0.0018	0.24	0.38	0.14	AUTS2	Body	island
cg06495631	-0.14	0.0062	0.24	0.37	0.14	ADAMTS2	Body	opensea
cg24314564	-0.14	0.0326	0.58	0.72	0.14		IGR	shelf
cg01063579	-0.14	0.0114	0.62	0.76	0.14		IGR	island
cg26504263	-0.14	0.0023	0.63	0.76	0.14	ANKRD6	5'UTR	opensea
cg08314176	-0.14	0.0054	0.27	0.40	0.14		IGR	opensea
cg03044684	-0.14	0.0386	0.48	0.61	0.14	HUNK	Body	shore
cg23543318	-0.14	0.0067	0.55	0.68	0.14	LOC100130872	Body	opensea
cg06161600	-0.14	0.0078	0.34	0.48	0.14	BAIAP3	Body	island
cg18877271	-0.14	0.0011	0.37	0.51	0.14	TNNT3	Body	shore
cg13017929	-0.14	0.0082	0.66	0.80	0.14		IGR	opensea
cg09447675	-0.14	0.0201	0.42	0.56	0.14		IGR	opensea
cg00166722	-0.14	0.0081	0.73	0.87	0.14	C3orf24	TSS200	island
cg06363692	-0.14	0.0089	0.61	0.75	0.14	RNF121	Body	opensea
cg20651995	-0.14	0.0047	0.57	0.70	0.14		IGR	opensea
cg26284735	-0.14	0.0035	0.49	0.63	0.14		IGR	shelf
cg01610979	-0.14	0.0039	0.28	0.41	0.14		IGR	opensea
cg23920246	-0.14	0.0066	0.75	0.89	0.14		IGR	opensea
cg09233395	-0.14	0.0125	0.43	0.56	0.14	TRPS1	5'UTR	shore
cg11452329	-0.14	0.0000	0.41	0.54	0.14		IGR	opensea
cg23252259	-0.14	0.0187	0.46	0.60	0.14		IGR	shore
cg24631102	-0.14	0.0000	0.60	0.74	0.14	NPS	Body	opensea
cg14640149	-0.14	0.0059	0.64	0.78	0.14		IGR	opensea
cg21727223	-0.14	0.0008	0.35	0.49	0.14		IGR	opensea
cg13001142	-0.14	0.0007	0.64	0.78	0.14	STXBP5	Body	shelf
cg09548403	-0.14	0.0081	0.31	0.45	0.14		IGR	opensea
cg24062389	-0.14	0.0031	0.65	0.79	0.14	BIVM	Body	opensea
cg18456803	-0.14	0.0011	0.42	0.56	0.14	ELF1	TSS200	opensea
cg24178897	-0.14	0.0082	0.60	0.74	0.14		IGR	opensea
cg01409343	-0.14	0.0080	0.40	0.54	0.14	TMEM49	Body	opensea
cg21335012	-0.14	0.0022	0.73	0.87	0.14		IGR	opensea
cg23186333	-0.14	0.0032	0.75	0.89	0.14	CD44	Body	shore
cg26235990	-0.14	0.0027	0.54	0.68	0.14		IGR	opensea
cg02992067	-0.14	0.0008	0.72	0.86	0.14	FTO	Body	opensea
cg13941978	-0.14	0.0020	0.50	0.64	0.14	PLEKHO2	Body	opensea
cg01993169	-0.14	0.0038	0.75	0.89	0.14		IGR	opensea
cg02033582	-0.14	0.0047	0.51	0.65	0.14		IGR	opensea
cg04926881	-0.14	0.0000	0.22	0.36	0.14		IGR	opensea

cg18117669	-0.14	0.0038	0.64	0.79	0.14	TCF12	TSS1500	opensea
cg22473770	-0.14	0.0106	0.54	0.68	0.14	EVI2A	5'UTR	opensea
cg09228833	-0.14	0.0128	0.50	0.64	0.14	ZNF217	TSS200	shore
cg05940691	-0.14	0.0143	0.58	0.72	0.14	WDR64	Body	opensea
cg04057161	-0.14	0.0086	0.67	0.81	0.14		IGR	opensea
cg10732871	-0.14	0.0065	0.31	0.45	0.14	GPX4	TSS1500	shore
cg05875421	-0.14	0.0069	0.71	0.86	0.14	GPR68	5'UTR	opensea
cg19770281	-0.14	0.0217	0.64	0.78	0.14		IGR	opensea
cg24475182	-0.14	0.0080	0.24	0.38	0.14		IGR	opensea
cg15690347	-0.14	0.0027	0.18	0.33	0.14	SPIB	Body	island
cg07813142	-0.14	0.0193	0.11	0.26	0.14	SP5	Body	island
cg11118962	-0.14	0.0082	0.48	0.62	0.14		IGR	opensea
cg13047869	-0.14	0.0160	0.74	0.88	0.14	C3orf24	1stExon	island
cg14986890	-0.14	0.0117	0.55	0.69	0.14	RARRES1	Body	opensea
cg20848291	-0.14	0.0001	0.25	0.39	0.14	ZAN	Body	opensea
cg06850787	-0.14	0.0052	0.41	0.56	0.14		IGR	opensea
cg18110333	-0.14	0.0096	0.30	0.44	0.14	DUSP22	1stExon	island
cg21708130	-0.14	0.0066	0.29	0.44	0.14	LRRFIP1	Body	shelf
cg21818891	-0.14	0.0009	0.78	0.93	0.14	SLC1A2	Body	opensea
cg08939850	-0.15	0.0178	0.60	0.74	0.15	RPTOR	Body	shore
cg12024811	-0.15	0.0002	0.62	0.77	0.15	KALRN	Body	opensea
cg01195564	-0.15	0.0084	0.60	0.74	0.15		IGR	opensea
cg08426157	-0.15	0.0017	0.67	0.82	0.15	HDAC9	Body	opensea
cg11562411	-0.15	0.0005	0.27	0.41	0.15	SCUBE3	Body	opensea
cg14733031	-0.15	0.0018	0.51	0.66	0.15	B3GNT3	TSS1500	shore
cg07805542	-0.15	0.0065	0.51	0.66	0.15	PIK3CD	Body	shelf
cg21341586	-0.15	0.0083	0.47	0.62	0.15	EIF4E	5'UTR	shore
cg21860675	-0.15	0.0096	0.70	0.84	0.15	FOXP1	5'UTR	opensea
cg02515217	-0.15	0.0007	0.46	0.61	0.15	MIR21	TSS200	opensea
cg10316899	-0.15	0.0031	0.38	0.53	0.15	MACF1	Body	opensea
cg22305268	-0.15	0.0052	0.54	0.69	0.15		IGR	shore
cg18363918	-0.15	0.0016	0.24	0.39	0.15	IGLON5	Body	shore
cg16002660	-0.15	0.0018	0.45	0.60	0.15	LOC284009	Body	opensea
cg07986257	-0.15	0.0004	0.66	0.81	0.15		IGR	opensea
cg25173405	-0.15	0.0102	0.10	0.25	0.15	C17orf57	5'UTR	shore
cg25015038	-0.15	0.0177	0.42	0.57	0.15		IGR	opensea
cg25773259	-0.15	0.0016	0.64	0.79	0.15		IGR	opensea
cg18263166	-0.15	0.0084	0.59	0.74	0.15		IGR	opensea
cg26203572	-0.15	0.0007	0.66	0.81	0.15		IGR	opensea
cg24772753	-0.15	0.0073	0.20	0.35	0.15	SP5	Body	island
cg10557907	-0.15	0.0080	0.44	0.59	0.15	PRDM16	Body	opensea
cg11235602	-0.15	0.0007	0.24	0.39	0.15	MOBP	Body	island
cg11809668	-0.15	0.0030	0.65	0.81	0.15	KIAA0922	Body	opensea

cg16683060	-0.15	0.0191	0.52	0.68	0.15	UBAC2	Body	opensea
cg16337566	-0.15	0.0002	0.26	0.41	0.15	PINX1	Body	opensea
cg00117018	-0.16	0.0165	0.50	0.65	0.16	ZNF251	Body	island
cg03839782	-0.16	0.0021	0.64	0.80	0.16	FAM65B	5'UTR	opensea
cg13052638	-0.16	0.0060	0.30	0.46	0.16		IGR	shelf
cg08159989	-0.16	0.0036	0.50	0.66	0.16	KLHDC8A	TSS200	opensea
cg04760448	-0.16	0.0078	0.50	0.65	0.16	COL18A1	Body	island
cg07677157	-0.16	0.0323	0.64	0.80	0.16		IGR	opensea
cg03548415	-0.16	0.0068	0.39	0.54	0.16		IGR	opensea
cg07506560	-0.16	0.0015	0.58	0.74	0.16		IGR	opensea
cg24921221	-0.16	0.0014	0.21	0.37	0.16	LONRF1	Body	opensea
cg12686055	-0.16	0.0001	0.23	0.39	0.16	ANO6	Body	opensea
cg16233797	-0.16	0.0060	0.66	0.82	0.16		IGR	opensea
cg02351277	-0.16	0.0031	0.28	0.43	0.16		IGR	opensea
cg27149179	-0.16	0.0013	0.37	0.53	0.16		IGR	opensea
cg06060522	-0.16	0.0288	0.43	0.59	0.16		IGR	island
cg05469819	-0.16	0.0043	0.62	0.78	0.16		IGR	opensea
cg04236915	-0.16	0.0014	0.48	0.65	0.16	ECE1	Body	opensea
cg07147204	-0.16	0.0054	0.78	0.95	0.16		IGR	opensea
cg11906781	-0.17	0.0067	0.34	0.51	0.17	BRE	Body	opensea
cg02573091	-0.17	0.0158	0.25	0.42	0.17		IGR	shore
cg24367957	-0.17	0.0122	0.37	0.54	0.17		IGR	opensea
cg17178175	-0.17	0.0041	0.35	0.52	0.17	NFE2L2	Body	opensea
cg16336556	-0.17	0.0029	0.56	0.73	0.17	LTBP1	Body	opensea
cg23201812	-0.17	0.0034	0.33	0.50	0.17		IGR	opensea
cg02500300	-0.18	0.0002	0.18	0.35	0.18	STOX2	1stExon	island
cg04470054	-0.18	0.0185	0.48	0.66	0.18	RPTOR	Body	shore
cg23821329	-0.18	0.0067	0.44	0.62	0.18	VIM	TSS1500	shore
cg21144063	-0.18	0.0042	0.31	0.49	0.18		IGR	opensea
cg12403162	-0.19	0.0014	0.31	0.49	0.19	ABLIM1	Body	shelf
cg01923775	-0.19	0.0002	0.40	0.59	0.19	PALLD	Body	opensea
cg17311132	-0.19	0.0022	0.62	0.81	0.19		IGR	opensea
cg15694704	-0.19	0.0015	0.48	0.67	0.19	RPTOR	Body	shelf
cg15532640	-0.19	0.0470	0.22	0.41	0.19		IGR	opensea
cg01412970	-0.19	0.0048	0.25	0.44	0.19	PLD6	1stExon	island
cg10587082	-0.20	0.0113	0.47	0.66	0.20	PLXNA2	Body	opensea
cg00601450	-0.20	0.0059	0.41	0.61	0.20		IGR	shore
cg23099839	-0.20	0.0092	0.23	0.43	0.20		IGR	opensea
cg02782634	-0.20	0.0001	0.28	0.49	0.20	TMEM49	Body	opensea
cg17749961	-0.20	0.0280	0.11	0.31	0.20	LCLAT1	TSS1500	shore
cg24693760	-0.20	0.0270	0.32	0.53	0.20		IGR	opensea
cg27477494	-0.21	0.0017	0.36	0.57	0.21		IGR	opensea
cg20905796	-0.21	0.0366	0.47	0.68	0.21		IGR	opensea

cg24113973	-0.21	0.0028	0.16	0.37	0.21		IGR	shelf
cg25588844	-0.21	0.0004	0.43	0.64	0.21	TAF1B	Body	opensea
cg08085267	-0.22	0.0018	0.13	0.34	0.22	C17orf57	5'UTR	shore
cg12592365	-0.22	0.0021	0.37	0.60	0.22	RPTOR	Body	opensea
cg19318364	-0.24	0.0012	0.63	0.87	0.24		IGR	opensea
cg19683494	-0.25	0.0183	0.36	0.60	0.25		IGR	shore
cg15652532	-0.27	0.0398	0.26	0.53	0.27	LCLAT1	TSS1500	shore
cg22443212	-0.44	0.0002	0.24	0.68	0.44	RNF213	Body	opensea

Supplemental Table 6. 130 differentially methylated positions in Pre-LVAD vs. Post-LVAD (Reverse Remodeling) (adjusted p-value <0.05, abs (delta beta) > 10%)

CpG Site	log FC	Adj. p-val	Pre-LVAD	Post-LVAD	Delta B	Gene	Feature	CGI
cg09559189	0.17	0.0001	0.37	0.20	-0.17	EBF2	Body	shore
cg22280475	0.15	0.0033	0.34	0.19	-0.15	EBF2	Body	island
cg14855519	0.14	0.0004	0.39	0.25	-0.14	EBF2	Body	shore
cg18239431	0.14	0.0005	0.28	0.14	-0.14	EBF2	Body	shore
cg03795776	0.14	0.0020	0.43	0.29	-0.14	BACH2	Body	opensea
cg16310415	0.14	0.0001	0.33	0.19	-0.14	EBF2	Body	shore
cg17902947	0.13	0.0090	0.63	0.50	-0.13	PPAP2B	Body	shelf
cg12536527	0.13	0.0022	0.32	0.19	-0.13	CACNA2D1	Body	opensea
cg05748163	0.13	0.0030	0.32	0.19	-0.13	EBF2	Body	shore
cg05996789	0.12	0.0057	0.74	0.62	-0.12	SLC25A4	TSS1500	shore
cg25892587	0.12	0.0060	0.42	0.30	-0.12		IGR	opensea
cg04657684	0.12	0.0011	0.54	0.42	-0.12	ELMOD1	Body	opensea
cg12149795	0.12	0.0003	0.27	0.16	-0.12	DIP2A	Body	shelf
cg16613240	0.12	0.0044	0.38	0.27	-0.12		IGR	opensea
cg01386185	0.12	0.0002	0.74	0.63	-0.12		IGR	opensea
cg00066750	0.12	0.0311	0.75	0.64	-0.12	HEY2	Body	shore
cg24881607	0.11	0.0289	0.79	0.68	-0.11	HEY2	Body	island
cg02371631	0.11	0.0010	0.55	0.44	-0.11	CDK15	Body	opensea
cg17936488	0.11	0.0045	0.40	0.28	-0.11	FAM78A	1stExon	shore
cg00730887	0.11	0.0008	0.27	0.16	-0.11		IGR	opensea
cg14787880	0.11	0.0006	0.32	0.21	-0.11	BMPR2	Body	shore
cg13425294	0.11	0.0058	0.31	0.19	-0.11		IGR	opensea
cg15580052	0.11	0.0086	0.33	0.22	-0.11	B4GALNT3	Body	opensea
cg09901201	0.11	0.0201	0.74	0.63	-0.11	SCP2	3'UTR	opensea
cg10378032	0.11	0.0043	0.59	0.48	-0.11	RAVER2	Body	shelf
cg26874542	0.11	0.0031	0.55	0.44	-0.11	FGF18	Body	shelf
cg03731740	0.11	0.0107	0.54	0.43	-0.11	YTHDF2	TSS1500	shore
cg03077077	0.11	0.0304	0.77	0.66	-0.11	TMEM155	5'UTR	shore
cg04492228	0.11	0.0000	0.57	0.47	-0.11	GATA3	Body	shore
cg16411101	0.11	0.0020	0.79	0.68	-0.11	SSBP3	Body	opensea
cg22783664	0.11	0.0014	0.24	0.13	-0.11	STX11	5'UTR	opensea
cg19343518	0.11	0.0047	0.39	0.28	-0.11	ARID1B	Body	opensea
cg01900030	0.11	0.0064	0.71	0.60	-0.11	CDK6	Body	opensea
cg06734510	0.11	0.0138	0.78	0.67	-0.11	PLCL1	Body	shelf
cg01190989	0.11	0.0025	0.45	0.34	-0.11	GPR125	Body	opensea
cg06115614	0.11	0.0200	0.55	0.45	-0.11	GATA2	Body	shore
cg05980083	0.11	0.0032	0.92	0.81	-0.11	PTPN3	5'UTR	opensea
cg11577329	0.10	0.0062	0.77	0.67	-0.10	SLC12A7	Body	island
cg12251895	0.10	0.0004	0.24	0.13	-0.10	KLF7	Body	opensea

cg14362113	0.10	0.0032	0.70	0.59	-0.10		IGR	opensea
cg11421073	0.10	0.0022	0.49	0.38	-0.10	ARL6IP5	3'UTR	opensea
cg27078890	0.10	0.0021	0.28	0.18	-0.10	ETS1	TSS200	opensea
cg17065262	0.10	0.0002	0.38	0.27	-0.10	MYO18A	5'UTR	shore
cg08559364	0.10	0.0072	0.32	0.22	-0.10	VGLL4	Body	opensea
cg05152903	0.10	0.0179	0.36	0.25	-0.10	CSDA	Body	shelf
cg05648629	0.10	0.0014	0.59	0.49	-0.10	ADCY9	Body	shelf
cg00652274	0.10	0.0001	0.83	0.73	-0.10	LSAMP	Body	opensea
cg05634495	0.10	0.0044	0.36	0.26	-0.10		IGR	opensea
cg07263393	0.10	0.0495	0.46	0.36	-0.10	GATA2	Body	shore
cg12962542	-0.10	0.0132	0.50	0.60	0.10	MECOM	Body	opensea
cg09368075	-0.10	0.0019	0.47	0.57	0.10		IGR	shore
cg02829601	-0.10	0.0037	0.34	0.44	0.10	SYTL3	TSS200	opensea
cg11552287	-0.10	0.0167	0.37	0.47	0.10	LANCL2	TSS1500	shore
cg12810626	-0.10	0.0048	0.79	0.89	0.10	LY9	TSS1500	opensea
cg23404248	-0.10	0.0460	0.11	0.21	0.10		IGR	island
cg25391092	-0.10	0.0364	0.58	0.68	0.10	C10orf11	Body	opensea
cg21350115	-0.10	0.0044	0.26	0.36	0.10	CALCRL	1stExon	opensea
cg02719154	-0.10	0.0400	0.17	0.27	0.10		IGR	shore
cg14511677	-0.10	0.0157	0.39	0.49	0.10	ST14	Body	island
cg11036041	-0.10	0.0098	0.31	0.41	0.10	LIMCH1	Body	shore
cg23557926	-0.10	0.0243	0.30	0.40	0.10	CFH	TSS200	opensea
cg01552197	-0.10	0.0240	0.46	0.56	0.10		IGR	opensea
cg22817650	-0.10	0.0013	0.47	0.57	0.10	CDC73	Body	opensea
cg23044178	-0.10	0.0232	0.52	0.62	0.10	MICAL2	5'UTR	shelf
cg09255732	-0.10	0.0107	0.37	0.47	0.10	COL16A1	TSS1500	shore
cg25095814	-0.10	0.0038	0.37	0.47	0.10	CASP8	TSS200	opensea
cg01245224	-0.10	0.0240	0.73	0.83	0.10		IGR	opensea
cg00960700	-0.10	0.0075	0.16	0.26	0.10	TBCD	TSS1500	island
cg15521792	-0.10	0.0000	0.53	0.63	0.10	NUDT17	TSS1500	shore
cg08711175	-0.10	0.0108	0.23	0.33	0.10	NXPH4	Body	shelf
cg11217865	-0.10	0.0040	0.39	0.49	0.10	NRG2	3'UTR	shore
cg16336556	-0.10	0.0353	0.56	0.67	0.10	LTBP1	Body	opensea
cg09501687	-0.10	0.0141	0.41	0.51	0.10	C5orf62	Body	opensea
cg00067758	-0.10	0.0167	0.42	0.53	0.10		IGR	opensea
cg07113653	-0.10	0.0204	0.74	0.85	0.10	GFOD1	Body	opensea
cg23806894	-0.10	0.0174	0.13	0.23	0.10		IGR	shelf
cg05307752	-0.10	0.0323	0.59	0.70	0.10	ARHGAP15	Body	opensea
cg26575450	-0.10	0.0127	0.55	0.66	0.10		IGR	shore
cg21015470	-0.10	0.0087	0.60	0.70	0.10	OPCML	Body	opensea
cg01232969	-0.10	0.0224	0.43	0.53	0.10		IGR	opensea
cg02500300	-0.10	0.0040	0.18	0.28	0.10	STOX2	1stExon	island
cg03926751	-0.11	0.0138	0.65	0.75	0.11	KLHL8	5'UTR	shore

cg12590005	-0.11	0.0044	0.18	0.28	0.11	MAPK14	Body	shore
cg23618477	-0.11	0.0015	0.55	0.66	0.11		IGR	shelf
cg15174393	-0.11	0.0256	0.37	0.48	0.11		IGR	opensea
cg13959207	-0.11	0.0020	0.57	0.68	0.11	ATP13A3	TSS200	opensea
cg25838968	-0.11	0.0295	0.54	0.64	0.11	PLXNA2	Body	opensea
cg09233395	-0.11	0.0162	0.43	0.53	0.11	TRPS1	5'UTR	shore
cg01180552	-0.11	0.0384	0.62	0.73	0.11		IGR	island
cg27520536	-0.11	0.0280	0.52	0.63	0.11		IGR	island
cg04067806	-0.11	0.0002	0.68	0.78	0.11		IGR	shore
cg09217350	-0.11	0.0060	0.43	0.53	0.11		IGR	opensea
cg15931471	-0.11	0.0097	0.54	0.65	0.11	SMAD6	Body	opensea
cg07034563	-0.11	0.0091	0.69	0.80	0.11	PDLIM7	Body	shore
cg26877720	-0.11	0.0017	0.64	0.75	0.11	FAM107B	Body	shore
cg08750534	-0.11	0.0007	0.24	0.35	0.11	SPRED2	Body	opensea
cg20765408	-0.11	0.0088	0.61	0.71	0.11	PARP4	5'UTR	shore
cg07777652	-0.11	0.0039	0.71	0.82	0.11	WRB	Body	shore
cg08141395	-0.11	0.0060	0.18	0.29	0.11	MAML2	Body	opensea
cg09275704	-0.11	0.0331	0.46	0.57	0.11		IGR	island
cg11509088	-0.11	0.0000	0.53	0.63	0.11	WDFY4	Body	opensea
cg22184990	-0.11	0.0041	0.39	0.50	0.11	ARHGAP26	Body	opensea
cg19541865	-0.11	0.0107	0.69	0.80	0.11	ACPP	TSS1500	opensea
cg10229594	-0.11	0.0051	0.25	0.36	0.11		IGR	opensea
cg24166450	-0.11	0.0027	0.34	0.45	0.11		IGR	opensea
cg16867680	-0.11	0.0047	0.30	0.41	0.11		IGR	opensea
cg21994818	-0.11	0.0002	0.49	0.60	0.11		IGR	opensea
cg21594702	-0.11	0.0235	0.36	0.47	0.11	VCAN	5'UTR	shore
cg23821329	-0.11	0.0331	0.44	0.55	0.11	VIM	TSS1500	shore
cg27285720	-0.11	0.0002	0.36	0.48	0.11	GBP4	TSS200	opensea
cg14553740	-0.11	0.0190	0.45	0.56	0.11	FAM154A	Body	opensea
cg16496024	-0.11	0.0023	0.35	0.46	0.11	PCP4	TSS1500	opensea
cg17941330	-0.11	0.0161	0.23	0.34	0.11	GJA5	TSS200	opensea
cg09251959	-0.11	0.0051	0.42	0.53	0.11	COL16A1	TSS1500	shore
cg10699857	-0.11	0.0376	0.11	0.22	0.11		IGR	island
cg11074047	-0.12	0.0013	0.52	0.64	0.12	NFATC2	Body	shore
cg05033239	-0.12	0.0079	0.36	0.47	0.12	GPR98	Body	opensea
cg04456219	-0.12	0.0292	0.34	0.46	0.12		IGR	opensea
cg22305268	-0.12	0.0008	0.54	0.66	0.12		IGR	shore
cg10308253	-0.12	0.0050	0.30	0.42	0.12	ZC3H12D	5'UTR	opensea
cg01973456	-0.12	0.0386	0.53	0.65	0.12		IGR	shelf
cg06088745	-0.12	0.0298	0.47	0.60	0.12	IRX3	3'UTR	shore
cg18142262	-0.12	0.0116	0.18	0.31	0.12		IGR	opensea
cg26639076	-0.13	0.0047	0.46	0.59	0.13	RIF1	3'UTR	opensea
cg18693345	-0.13	0.0144	0.25	0.38	0.13	C5orf38	Body	island

cg03044684	-0.13	0.0214	0.48	0.61	0.13	HUNK	Body	shore
cg13064658	-0.14	0.0057	0.07	0.21	0.14	LPGAT1	5'UTR	island
cg15945235	-0.15	0.0008	0.49	0.64	0.15	ANKRD22	1stExon	opensea
cg07904452	-0.15	0.0008	0.36	0.51	0.15		IGR	opensea
cg19611616	-0.22	0.0293	0.03	0.25	0.22	STK38L	5'UTR	shore

Supplemental Table 7. 35 LVAD Responsive HF associated differentially methylated positions (adjusted p-value <0.05, abs (delta beta) > 10%)

CpG Site	Non-Failing	Pre-LVAD	Post-LVAD	Delta HF	Delta LVAD	Gene	Feature	CGI
cg23821329	0.62	0.44	0.55	-0.18	0.11	VIM	TSS1500	shore
cg02500300	0.35	0.18	0.28	-0.18	0.10	STOX2	1stExon	island
cg16336556	0.73	0.56	0.67	-0.17	0.10	LTBP1	Body	opensea
cg22305268	0.69	0.54	0.66	-0.15	0.12		IGR	shore
cg09233395	0.56	0.43	0.53	-0.14	0.11	TRPS1	5'UTR	shore
cg03044684	0.61	0.48	0.61	-0.14	0.13	HUNK	Body	shore
cg26877720	0.77	0.64	0.75	-0.13	0.11	FAM107B	Body	shore
cg27520536	0.65	0.52	0.63	-0.13	0.11		IGR	island
cg09251959	0.55	0.42	0.53	-0.13	0.11	COL16A1	TSS1500	shore
cg26575450	0.68	0.55	0.66	-0.13	0.10		IGR	shore
cg14553740	0.58	0.45	0.56	-0.13	0.11	FAM154A	Body	opensea
cg15174393	0.49	0.37	0.48	-0.12	0.11		IGR	opensea
cg25838968	0.65	0.54	0.64	-0.12	0.11	PLXNA2	Body	opensea
cg04456219	0.46	0.34	0.46	-0.12	0.12		IGR	opensea
cg15931471	0.66	0.54	0.65	-0.12	0.11	SMAD6	Body	opensea
cg07034563	0.80	0.69	0.80	-0.11	0.11	PDLIM7	Body	shore
cg21994818	0.60	0.49	0.60	-0.11	0.11		IGR	opensea
cg15945235	0.60	0.49	0.64	-0.11	0.15	ANKRD22	1stExon	opensea
cg24166450	0.45	0.34	0.45	-0.11	0.11		IGR	opensea
cg20765408	0.71	0.61	0.71	-0.10	0.11	PARP4	5'UTR	shore
cg03926751	0.75	0.65	0.75	-0.10	0.11	KLHL8	5'UTR	shore
cg17936488	0.29	0.40	0.28	0.11	-0.11	FAM78A	1stExon	shore
cg04657684	0.43	0.54	0.42	0.11	-0.12	ELMOD1	Body	opensea
cg15580052	0.21	0.33	0.22	0.12	-0.11	B4GALNT3	Body	opensea
cg08559364	0.20	0.32	0.22	0.12	-0.10	VGLL4	Body	opensea
cg16310415	0.21	0.33	0.19	0.12	-0.14	EBF2	Body	shore
cg14855519	0.26	0.39	0.25	0.12	-0.14	EBF2	Body	shore
cg18239431	0.16	0.28	0.14	0.13	-0.14	EBF2	Body	shore
cg05748163	0.18	0.32	0.19	0.14	-0.13	EBF2	Body	shore
cg01900030	0.57	0.71	0.60	0.14	-0.11	CDK6	Body	opensea
cg01190989	0.30	0.45	0.34	0.15	-0.11	GPR125	Body	opensea
cg04492228	0.42	0.57	0.47	0.15	-0.11	GATA3	Body	shore
cg03731740	0.39	0.54	0.43	0.15	-0.11	YTHDF2	TSS1500	shore
cg22280475	0.18	0.34	0.19	0.16	-0.15	EBF2	Body	island
cg09559189	0.22	0.37	0.20	0.16	-0.17	EBF2	Body	shore

Supplemental Table 8. DNA Methylated CpG Sites that are located within or near (<10 kb) human long-non-coding RNA (lncRNA) genes

Heart Failure DMPs	lncRNA Name	lncRNA Type	DNA Strand	Genomic Location	Cardiac Expression GTEX (TPM)
**Ischemic and Non-Ischemic Cardiomyopathy DMPs**					
cg23099839	GS1-57L11.1	Intergenic	+	chr8:2584858-2680004	0.000
cg23099839	RP11-134O21.1	Intergenic	-	chr8:2523591-2585991	0.000
cg27149179	CTD-2005H7.2	Intergenic	+	chr11:86438397-86476307	0.078
cg07986257	RP3-404K8.2	Antisense	+	chr6:22260653-22318027	-
cg21727223	LINC01482	Intergenic	+	chr17:66587980-66746693	0.001
cg21727223	RP11-118B18.2	Intergenic	-	chr17:66789690-66793963	0.000
cg04926881	CTB-113D17.1	Antisense	+	chr7:29019583-29052983	0.198
cg04926881	AC005162.5	Antisense	+	chr7:29026644-29028515	0.000
cg01195564	LINC01331	Intergenic	-	chr5:73407515-73832649	0.000
cg26203572	LINC00525	Intergenic	+	chr7:47801074-47806370	0.168
cg02351277	LINC00578	Intergenic	+	chr3:177159709-177469882	0.000
cg02351277	RP11-114M1.1	Intergenic	+	chr3:177401415-177409038	0.000
cg24475182	RP11-134O21.1	Intergenic	-	chr8:2523591-2585991	0.000
cg24475182	GS1-57L11.1	Intergenic	+	chr8:2584858-2680004	0.000
cg07506560	AC093802.1	Intergenic	+	chr2:240684554-240724577	0.000
cg13052638	RP11-534L20.5	Intergenic	+	chr1:206677281-206677789	0.000
cg04057161	RP11-317M11.1	Sense	+	chr4:54525375-54603323	0.000
cg01412419	RP4-536B24.3	Intergenic	-	chr16:87813740-87840082	0.000
cg01412419	RP4-536B24.2	Antisense	+	chr16:87870138-87871269	0.000
cg08889114	LMCD1-AS1	Antisense	-	chr2:21444047-22193831	0.054
cg08889114	AC034187.2	Intergenic	-	chr3:8615412-8634810	0.000
cg14111334	RP11-626H12.1	Intergenic	+	chr11:69831982-69861921	0.070
cg14111334	RP11-626H12.2	Intergenic	-	chr11:69860964-69867165	0.113
cg14387312	LINC01618	Intergenic	+	chr4:53578561-53732988	0.000
cg19590421	RP11-218E20.2	Antisense	+	chr14:51314840-51332377	0.000
cg25720795	HOTTIP	Antisense	+	chr7:27241461-27246878	0.000
cg01535205	LINC00880	Intergenic	-	chr3:156799456-156840793	0.031
cg01535205	LINC00881	Intergenic	+	chr3:156807670-156818924	35.17
cg16200531	CCNT2-AS1	Antisense	-	chr2:135493034-135676240	0.444
cg03329019	HLX-AS1	Antisense	-	chr1:221006105-221053482	0.053
cg19657945	MINCR	Antisense	-	chr8:144362336-144363830	2.42
cg13283845	MINCR	Antisense	-	chr8:144362336-144363830	2.42
cg26536949	AC108004.2	Intergenic	-	chr17:33615-41378	0.068
cg05528899	AC108004.2	Intergenic	-	chr17:33615-41378	0.068
**LVAD Responsive HF DMPs**					
cg26575450	LINC02579	Intergenic	+	chr2:64834446-64843616	0.000
cg21994818	C8orf37-AS1	Antisense	+	chr8:96281064-96822371	0.000

Supplemental Table 9. Top 100 mRNAs that are regulated by LINC00881 plasmid overexpression in the beating human iPS cell derived cardiomyocytes by Deseq2

Gene ID	Base	Log2 FC	lfc SE	stat	p value	p adj
LINC00881	3323.2	2.07	0.18	11.38	0.0000	0.0000
MYH6	58976.6	0.35	0.07	4.98	0.0000	0.0105
FXYD6	608.8	0.41	0.08	4.84	0.0000	0.0143
DNTTIP2	1686.7	-0.33	0.07	-4.66	0.0000	0.0253
ARL6IP1	1427.0	-0.29	0.06	-4.55	0.0000	0.0350
EIF2S2	3515.4	-0.24	0.05	-4.47	0.0000	0.0428
CACNA1C	1375.7	0.36	0.08	4.39	0.0000	0.0461
HSPG2	1642.4	0.29	0.07	4.36	0.0000	0.0461
SYF2	532.0	-0.36	0.08	-4.36	0.0000	0.0461
POLR1F	592.9	-0.38	0.09	-4.21	0.0000	0.0797
RIOK2	1058.1	-0.30	0.07	-4.19	0.0000	0.0797
ZNF146	1964.0	-0.25	0.06	-4.16	0.0000	0.0797
MT-RNR2	101974.6	-0.29	0.07	-4.16	0.0000	0.0797
NFE2L2	1280.6	-0.26	0.06	-4.14	0.0000	0.0808
MYBPC3	12012.0	0.35	0.09	4.08	0.0000	0.0966
CEBPZ	1638.6	-0.23	0.06	-4.01	0.0001	0.1251
CALR	3952.5	0.31	0.08	3.99	0.0001	0.1276
NID2	3748.1	0.30	0.07	3.97	0.0001	0.1276
LDLR	1381.0	0.32	0.08	3.95	0.0001	0.1310
A2M	8017.2	0.36	0.09	3.94	0.0001	0.1310
DYSF	1200.8	0.28	0.07	3.92	0.0001	0.1386
CNN2	1355.3	0.30	0.08	3.88	0.0001	0.1474
UNC5B	1389.8	0.26	0.07	3.88	0.0001	0.1474
RGS4	696.6	-0.54	0.14	-3.87	0.0001	0.1476
ACACB	981.4	0.26	0.07	3.85	0.0001	0.1476
EMC2	728.0	-0.30	0.08	-3.85	0.0001	0.1476
TMEM167A	4189.5	-0.25	0.07	-3.77	0.0002	0.1808
CACNA1D	591.3	0.35	0.09	3.76	0.0002	0.1808
ZFAND5	3454.4	-0.23	0.06	-3.75	0.0002	0.1808
VCAN	4920.5	0.21	0.06	3.75	0.0002	0.1808
PHAX	600.5	-0.28	0.08	-3.75	0.0002	0.1808
SVIL	9311.4	0.19	0.05	3.74	0.0002	0.1808
SEC62	2171.8	-0.31	0.08	-3.74	0.0002	0.1808
TM4SF1	118.4	-0.54	0.15	-3.72	0.0002	0.1888
CDC42EP3	3137.1	-0.31	0.08	-3.71	0.0002	0.1902
RPL14	3635.7	-0.25	0.07	-3.70	0.0002	0.1954
COL6A1	751.1	0.44	0.12	3.68	0.0002	0.2014
SYNE2	814.5	0.25	0.07	3.67	0.0002	0.2014
OARD1	618.8	-0.30	0.08	-3.67	0.0002	0.2014

CIR1	624.8	-0.29	0.08	-3.66	0.0002	0.2016
ESF1	641.2	-0.32	0.09	-3.65	0.0003	0.2083
GNAI3	1217.0	-0.24	0.07	-3.63	0.0003	0.2146
MT-CO2	63460.8	-0.22	0.06	-3.63	0.0003	0.2146
MT-RNR1	13798.2	-0.24	0.07	-3.62	0.0003	0.2146
C16orf72	1744.5	-0.25	0.07	-3.61	0.0003	0.2199
PYROXD2	293.7	0.42	0.12	3.60	0.0003	0.2199
ZNF800	265.3	-0.44	0.12	-3.60	0.0003	0.2199
BNIP2	1981.3	-0.24	0.07	-3.59	0.0003	0.2249
CFAP97	1046.6	-0.28	0.08	-3.57	0.0004	0.2249
GAA	288.3	0.39	0.11	3.57	0.0004	0.2249
ACTA2	4114.2	0.33	0.09	3.56	0.0004	0.2249
SLK	3830.9	-0.24	0.07	-3.56	0.0004	0.2249
PXDN	1313.2	0.22	0.06	3.56	0.0004	0.2249
PDIA4	1199.4	0.31	0.09	3.55	0.0004	0.2249
TAF1D	747.3	-0.33	0.09	-3.54	0.0004	0.2249
ZNF281	1022.4	-0.28	0.08	-3.53	0.0004	0.2249
LSS	897.1	0.37	0.11	3.53	0.0004	0.2249
EIF4A2	7267.6	-0.18	0.05	-3.52	0.0004	0.2249
PIK3CA	1201.6	-0.30	0.08	-3.52	0.0004	0.2249
TMEM167B	994.4	-0.25	0.07	-3.52	0.0004	0.2249
PLEKHA6	575.9	0.32	0.09	3.52	0.0004	0.2249
DNAJC2	660.2	-0.30	0.09	-3.51	0.0004	0.2249
PPP4R2	2391.2	-0.28	0.08	-3.51	0.0004	0.2249
RAD17	1171.7	-0.24	0.07	-3.51	0.0005	0.2249
CAMK2A	243.5	0.37	0.11	3.50	0.0005	0.2249
ADGRB2	727.1	0.39	0.11	3.50	0.0005	0.2249
AFTPH	449.7	-0.31	0.09	-3.50	0.0005	0.2249
SH3GLB2	417.9	0.34	0.10	3.49	0.0005	0.2339
RAB22A	499.2	-0.29	0.08	-3.48	0.0005	0.2339
KCNH7	131.9	0.50	0.14	3.48	0.0005	0.2339
SLU7	1103.9	-0.26	0.08	-3.46	0.0005	0.2339
RPL23A	3334.9	-0.25	0.07	-3.46	0.0005	0.2339
GREB1	1164.5	0.22	0.06	3.46	0.0005	0.2339
ADAM11	321.3	0.40	0.12	3.46	0.0005	0.2339
KLHL41	1289.4	-0.24	0.07	-3.46	0.0005	0.2339
SCUBE3	1507.6	0.24	0.07	3.46	0.0005	0.2339
NMD3	1603.0	-0.24	0.07	-3.44	0.0006	0.2361
UBE3A	3478.2	-0.21	0.06	-3.44	0.0006	0.2361
KLHL9	1370.4	-0.24	0.07	-3.44	0.0006	0.2361
POT1	467.7	-0.28	0.08	-3.44	0.0006	0.2361
DMAP1	306.1	0.32	0.09	3.43	0.0006	0.2361
MYH7B	782.1	0.32	0.09	3.43	0.0006	0.2361

UBE2V2	1054.1	-0.27	0.08	-3.42	0.0006	0.2430
OBI1	1128.3	-0.29	0.08	-3.42	0.0006	0.2430
CCT8	4009.7	-0.22	0.07	-3.42	0.0006	0.2430
RAD23B	3510.6	-0.23	0.07	-3.41	0.0006	0.2434
LAMB2	3760.3	0.31	0.09	3.41	0.0007	0.2456
AP5M1	1967.0	-0.24	0.07	-3.39	0.0007	0.2504
SERP1	620.1	-0.28	0.08	-3.39	0.0007	0.2504
TENM4	687.6	0.27	0.08	3.39	0.0007	0.2504
FAM107B	271.4	-0.33	0.10	-3.39	0.0007	0.2513
ZNF639	787.2	-0.26	0.08	-3.38	0.0007	0.2526
RBAK	821.8	-0.31	0.09	-3.38	0.0007	0.2554
COL5A1	352.3	0.33	0.10	3.37	0.0007	0.2566
CHD1	990.2	-0.25	0.07	-3.35	0.0008	0.2802
MSANTD4	1268.7	-0.26	0.08	-3.34	0.0009	0.2837
LRP1	1043.1	0.30	0.09	3.33	0.0009	0.2837
MYOM2	107.3	0.51	0.15	3.33	0.0009	0.2837
EMILIN2	3342.9	0.20	0.06	3.33	0.0009	0.2837
ATP2B4	3356.0	0.23	0.07	3.33	0.0009	0.2837

Supplemental Table 10. Top 100 mRNAs that are regulated by LINC00881 GapmeR knockdown in the beating human iPS cell derived cardiomyocytes by Deseq2

Gene ID	Base	log2 FC	lfc SE	stat	p value	p adj
DTNA	1940.6	-1.08	0.17	-6.19	0.0000	0.0000
LINC00881	1907.5	-2.27	0.39	-5.84	0.0000	0.0000
ERRFI1	605.7	1.21	0.23	5.29	0.0000	0.0007
THADA	523.8	-1.32	0.25	-5.26	0.0000	0.0007
FOSL1	75.1	2.10	0.41	5.11	0.0000	0.0012
F2RL2	17.3	6.88	1.36	5.07	0.0000	0.0012
FAM53C	3717.1	0.71	0.14	5.03	0.0000	0.0013
VPS45	2903.3	-0.89	0.18	-4.98	0.0000	0.0015
RP11-419I17.1	167.8	-1.17	0.24	-4.80	0.0000	0.0032
CYB561	598.2	1.38	0.29	4.78	0.0000	0.0032
CCNG1	5904.2	0.62	0.13	4.76	0.0000	0.0032
NUS1	513.9	0.76	0.16	4.71	0.0000	0.0034
FARP1	859.5	-0.85	0.18	-4.70	0.0000	0.0034
GALNT11	1152.6	-0.61	0.13	-4.70	0.0000	0.0034
RSU1	3123.8	-0.82	0.18	-4.65	0.0000	0.0041
TBCD	875.3	-1.04	0.22	-4.62	0.0000	0.0042
NOCT	172.8	1.04	0.23	4.62	0.0000	0.0042
KLF12	667.5	-1.04	0.23	-4.60	0.0000	0.0043
MYLIP	1137.7	1.04	0.23	4.58	0.0000	0.0045
KIF26B	1749.9	-1.01	0.22	-4.53	0.0000	0.0048
PMAIP1	386.9	1.34	0.30	4.53	0.0000	0.0048
CDH4	136.8	-1.51	0.33	-4.53	0.0000	0.0048
PLCXD2	63.3	2.00	0.44	4.53	0.0000	0.0048
SLC16A14	186.9	1.84	0.41	4.50	0.0000	0.0051
RP11-556O5.7	50.1	-2.55	0.57	-4.47	0.0000	0.0058
TRAPPC9	349.1	-1.08	0.24	-4.44	0.0000	0.0064
CDC42EP1	325.5	1.53	0.35	4.43	0.0000	0.0064
ATF6	1325.9	-0.81	0.18	-4.42	0.0000	0.0064
ADAM23	553.6	-1.76	0.40	-4.40	0.0000	0.0070
FBXW8	845.3	-0.71	0.16	-4.39	0.0000	0.0070
SLC12A7	1981.0	-0.81	0.19	-4.38	0.0000	0.0070
DOCK1	490.8	-1.15	0.26	-4.37	0.0000	0.0070
NDUFV2	56.8	1.68	0.38	4.37	0.0000	0.0070
KLF10	842.4	2.39	0.55	4.36	0.0000	0.0072
SIX4	328.9	0.98	0.22	4.35	0.0000	0.0072
WDR59	471.3	-1.43	0.33	-4.34	0.0000	0.0072
DUSP1	1488.4	0.74	0.17	4.33	0.0000	0.0073
PRKCI	1235.1	0.63	0.14	4.33	0.0000	0.0073
SLC3A2	6297.2	0.79	0.18	4.32	0.0000	0.0073

CRADD	661.9	-1.27	0.30	-4.29	0.0000	0.0082
ACTBL2	15.4	7.44	1.74	4.29	0.0000	0.0082
BCLAF1	5568.3	0.73	0.17	4.28	0.0000	0.0083
FOXH1	175.8	2.99	0.70	4.27	0.0000	0.0084
LZTS3	352.8	1.01	0.24	4.26	0.0000	0.0084
GPR3	126.3	1.63	0.38	4.26	0.0000	0.0084
PIK3C3	937.4	-0.96	0.23	-4.24	0.0000	0.0089
FBXO2	1219.5	0.61	0.14	4.24	0.0000	0.0089
CYB5RL	300.8	-0.88	0.21	-4.23	0.0000	0.0089
CAMLG	1914.9	0.59	0.14	4.22	0.0000	0.0094
FSTL3	573.8	1.07	0.25	4.21	0.0000	0.0096
RCC2	1164.4	0.80	0.19	4.19	0.0000	0.0100
EDA	24.8	-3.68	0.88	-4.19	0.0000	0.0100
RALYL	99.1	-1.78	0.43	-4.18	0.0000	0.0101
CHST9	18.1	-4.99	1.20	-4.16	0.0000	0.0110
MARCHF11	387.0	-1.65	0.40	-4.15	0.0000	0.0113
ACVR2B	1213.5	0.65	0.16	4.14	0.0000	0.0115
RP11-110G21.1	254.9	-0.82	0.20	-4.12	0.0000	0.0124
SERP1	2751.2	0.62	0.15	4.11	0.0000	0.0125
JUND	235.7	1.16	0.28	4.09	0.0000	0.0135
GPER1	55.9	1.72	0.42	4.09	0.0000	0.0135
MAFF	84.7	1.28	0.31	4.07	0.0000	0.0139
DISP1	336.4	-0.88	0.22	-4.06	0.0000	0.0139
TSPYL2	1481.3	1.42	0.35	4.06	0.0000	0.0139
MAPKAP1	2286.2	-0.72	0.18	-4.06	0.0000	0.0139
RP11-74J13.8	39.7	-1.97	0.49	-4.06	0.0000	0.0139
DYM	1056.3	-0.68	0.17	-4.06	0.0000	0.0139
AP4S1	266.8	-0.91	0.23	-4.04	0.0001	0.0148
YOD1	947.7	0.59	0.15	4.04	0.0001	0.0148
CACNA1C	898.0	-1.17	0.29	-4.03	0.0001	0.0148
ERI3	2031.7	-0.70	0.17	-4.02	0.0001	0.0155
PDLIM3	955.6	1.14	0.28	4.02	0.0001	0.0155
LINC00638	47.5	-2.24	0.56	-4.01	0.0001	0.0159
IMPG2	44.7	-5.04	1.26	-3.99	0.0001	0.0164
FTL	74039.0	1.26	0.31	3.99	0.0001	0.0165
P3H1	749.0	0.92	0.23	3.99	0.0001	0.0166
PHKB	4104.6	-0.60	0.15	-3.98	0.0001	0.0171
SOX9	141.1	1.44	0.36	3.97	0.0001	0.0173
CIB1	1466.3	0.57	0.14	3.97	0.0001	0.0174
RP11-274B21.4	13.4	-3.86	0.98	-3.96	0.0001	0.0178
NR4A1	237.8	1.14	0.29	3.95	0.0001	0.0179
ALB	73.1	-2.48	0.63	-3.94	0.0001	0.0181
CIDEC	10.7	-4.38	1.11	-3.93	0.0001	0.0181

AD000864.6	14.5	3.63	0.92	3.93	0.0001	0.0181
NAPG	1015.4	0.69	0.17	3.93	0.0001	0.0181
FTLP3	511.8	1.42	0.36	3.93	0.0001	0.0181
COBLL1	372.4	-0.92	0.23	-3.93	0.0001	0.0181
HECW2	157.8	-1.08	0.28	-3.93	0.0001	0.0181
DUSP3	8306.9	0.60	0.15	3.92	0.0001	0.0181
CSGALNACT2	685.9	0.60	0.15	3.92	0.0001	0.0181
LONRF1	991.1	0.83	0.21	3.92	0.0001	0.0181
HPR	9.8	5.29	1.35	3.92	0.0001	0.0181
CCDC117	2648.7	0.65	0.16	3.91	0.0001	0.0183
STIM2	736.4	-0.71	0.18	-3.90	0.0001	0.0189
MARK3	3086.1	-0.60	0.16	-3.89	0.0001	0.0197
UGT2B7	7.8	-4.92	1.27	-3.88	0.0001	NA
LL0XNC01-7P3.1	38.1	1.47	0.38	3.88	0.0001	0.0204
ARRDC4	2419.3	1.03	0.27	3.87	0.0001	0.0207
GHR	121.1	-1.16	0.30	-3.87	0.0001	0.0210
ITPKB	42.5	-1.92	0.50	-3.86	0.0001	0.0213

Supplemental Table 11. Genes significantly regulated by both LINC00881 overexpression and LINC0881 knockdown in human iPS cell derived cardiomyocytes

Gene ID	log2FC (OE)	p val (OE)	log2FC (KD)	p val (KD)	Direction
LINC00881	2.07	0.0000	-2.27	0.0000	Up-Down
SLC16A13	1.07	0.0177	-1.76	0.0244	Up-Down
DOC2A	1.34	0.0183	-1.67	0.0131	Up-Down
FAM189A1	0.29	0.0494	-1.66	0.0171	Up-Down
ITGB3	0.36	0.0332	-1.41	0.0350	Up-Down
GLB1L	0.43	0.0441	-1.40	0.0142	Up-Down
MYH7B	0.32	0.0006	-1.25	0.0227	Up-Down
CACNA1C	0.36	0.0000	-1.17	0.0001	Up-Down
LARGE1	0.24	0.0130	-1.16	0.0016	Up-Down
FRAS1	0.21	0.0041	-1.13	0.0014	Up-Down
TENM4	0.27	0.0007	-1.11	0.0002	Up-Down
RYR2	0.17	0.0168	-1.09	0.0124	Up-Down
MAPK4	0.29	0.0405	-1.08	0.0002	Up-Down
TRAPPC9	0.23	0.0152	-1.08	0.0000	Up-Down
TPCN1	0.34	0.0030	-1.06	0.0106	Up-Down
SLIT3	0.26	0.0012	-1.04	0.0002	Up-Down
CACNA1D	0.35	0.0002	-1.03	0.0032	Up-Down
RP3-412A9.16	1.08	0.0282	-1.03	0.0167	Up-Down
OBSCN	0.20	0.0214	-0.99	0.0236	Up-Down
SDK2	0.22	0.0055	-0.99	0.0064	Up-Down
EPHB2	0.29	0.0062	-0.98	0.0275	Up-Down
FHOD3	0.13	0.0156	-0.94	0.0008	Up-Down
PLXNA4	0.20	0.0027	-0.93	0.0006	Up-Down
FOXN3	0.15	0.0249	-0.91	0.0293	Up-Down
TAP1	0.30	0.0270	-0.90	0.0212	Up-Down
TSPAN18	0.23	0.0394	-0.89	0.0296	Up-Down
MYOM2	0.51	0.0009	-0.89	0.0011	Up-Down
MYO18B	0.22	0.0013	-0.87	0.0005	Up-Down
ACAD10	0.20	0.0405	-0.85	0.0009	Up-Down
SH3RF2	0.22	0.0118	-0.85	0.0269	Up-Down
ACACB	0.26	0.0001	-0.84	0.0145	Up-Down
SH3PXD2A	0.16	0.0213	-0.83	0.0155	Up-Down
PI4KA	0.18	0.0120	-0.79	0.0136	Up-Down
CLYBL	0.40	0.0030	-0.77	0.0102	Up-Down
GREB1	0.22	0.0005	-0.77	0.0056	Up-Down
TANC2	0.14	0.0454	-0.75	0.0245	Up-Down
PPARD	0.29	0.0045	-0.72	0.0040	Up-Down
IGSF9B	0.22	0.0036	-0.70	0.0477	Up-Down
PACS2	0.23	0.0112	-0.70	0.0392	Up-Down

FREM1	0.26	0.0011	-0.69	0.0156	Up-Down
ZNF76	0.27	0.0061	-0.67	0.0406	Up-Down
PATJ	0.16	0.0479	-0.67	0.0012	Up-Down
POLE	0.17	0.0495	-0.67	0.0006	Up-Down
MYOM1	0.14	0.0159	-0.66	0.0064	Up-Down
KCNH7	0.50	0.0005	-0.65	0.0398	Up-Down
PHKA2	0.20	0.0111	-0.64	0.0059	Up-Down
PLEKHA7	0.17	0.0193	-0.64	0.0006	Up-Down
FAM189A2	0.18	0.0211	-0.63	0.0091	Up-Down
FGFR2	0.17	0.0319	-0.61	0.0274	Up-Down
ANXA6	0.24	0.0049	-0.60	0.0351	Up-Down
CAPZB	0.13	0.0417	-0.59	0.0073	Up-Down
MYH6	0.35	0.0000	-0.59	0.0310	Up-Down
TLN2	0.19	0.0104	-0.58	0.0040	Up-Down
TSPAN9	0.18	0.0107	-0.57	0.0142	Up-Down
DGLUCY	0.18	0.0122	-0.56	0.0067	Up-Down
ITGA3	0.19	0.0460	-0.53	0.0458	Up-Down
MATN2	0.22	0.0279	-0.53	0.0200	Up-Down
LDB3	0.16	0.0045	-0.52	0.0048	Up-Down
MECR	0.27	0.0221	-0.52	0.0403	Up-Down
PPP6R2	0.22	0.0206	-0.52	0.0171	Up-Down
LSS	0.37	0.0004	-0.51	0.0240	Up-Down
KALRN	0.19	0.0282	-0.51	0.0094	Up-Down
ITGB1BP2	0.25	0.0139	-0.51	0.0342	Up-Down
DROSHA	0.15	0.0339	-0.51	0.0009	Up-Down
CARS2	0.24	0.0267	-0.47	0.0275	Up-Down
PRMT7	0.25	0.0085	-0.46	0.0167	Up-Down
RUSF1	0.30	0.0159	-0.45	0.0447	Up-Down
TNNI1	0.18	0.0499	-0.43	0.0277	Up-Down
PYROXD2	0.42	0.0003	-0.42	0.0260	Up-Down
CAMK2A	0.37	0.0005	-0.38	0.0165	Up-Down
PREP	0.17	0.0138	-0.36	0.0131	Up-Down
PWWP3A	0.23	0.0128	-0.33	0.0464	Up-Down
SELENOW	0.18	0.0140	-0.28	0.0388	Up-Down
SH3GLB1	-0.14	0.0258	0.27	0.0386	Down-Up
RNF2	-0.24	0.0014	0.27	0.0478	Down-Up
FAM222B	-0.22	0.0166	0.33	0.0424	Down-Up
SSB	-0.20	0.0119	0.33	0.0237	Down-Up
CHORDC1	-0.20	0.0211	0.33	0.0447	Down-Up
PRR13	-0.21	0.0386	0.34	0.0408	Down-Up
PAFAH1B2	-0.17	0.0161	0.35	0.0138	Down-Up
CNOT8	-0.16	0.0401	0.35	0.0325	Down-Up
TOMM20	-0.13	0.0341	0.36	0.0010	Down-Up

ZNF229	-0.21	0.0357	0.36	0.0461	Down-Up
RAB5A	-0.20	0.0068	0.36	0.0188	Down-Up
GASK1B	-0.20	0.0237	0.37	0.0468	Down-Up
BZW1	-0.20	0.0015	0.37	0.0053	Down-Up
YWHAG	-0.12	0.0322	0.38	0.0167	Down-Up
RIT1	-0.14	0.0166	0.38	0.0491	Down-Up
DNAJA1	-0.12	0.0431	0.40	0.0029	Down-Up
PHF10	-0.18	0.0156	0.40	0.0431	Down-Up
EIF5	-0.15	0.0035	0.41	0.0126	Down-Up
ZNF697	-0.18	0.0188	0.42	0.0251	Down-Up
CCT6A	-0.16	0.0049	0.43	0.0457	Down-Up
PPP4R2	-0.28	0.0004	0.43	0.0459	Down-Up
RMND5A	-0.18	0.0451	0.44	0.0009	Down-Up
CGGBP1	-0.21	0.0070	0.44	0.0423	Down-Up
MAPK6	-0.17	0.0148	0.44	0.0481	Down-Up
RAB14	-0.17	0.0087	0.45	0.0342	Down-Up
SMAD5	-0.18	0.0260	0.45	0.0279	Down-Up
TERF2IP	-0.21	0.0016	0.45	0.0097	Down-Up
MCL1	-0.17	0.0015	0.45	0.0106	Down-Up
SIRT1	-0.20	0.0089	0.46	0.0466	Down-Up
PSMD12	-0.14	0.0403	0.46	0.0005	Down-Up
ARF4	-0.17	0.0293	0.47	0.0117	Down-Up
RAD21	-0.11	0.0484	0.47	0.0290	Down-Up
PLEKHA3	-0.26	0.0009	0.48	0.0185	Down-Up
PNRC2	-0.16	0.0279	0.48	0.0217	Down-Up
RRM2B	-0.28	0.0128	0.48	0.0456	Down-Up
GMFB	-0.18	0.0208	0.49	0.0227	Down-Up
GXYLT1	-0.24	0.0132	0.49	0.0130	Down-Up
ZNF639	-0.26	0.0007	0.49	0.0115	Down-Up
SOCS4	-0.20	0.0392	0.50	0.0472	Down-Up
MTHFD2	-0.19	0.0103	0.50	0.0405	Down-Up
NAA50	-0.22	0.0021	0.50	0.0047	Down-Up
KIF5B	-0.19	0.0068	0.50	0.0026	Down-Up
CCN2	-0.18	0.0179	0.51	0.0329	Down-Up
KLHL28	-0.33	0.0013	0.51	0.0405	Down-Up
SLU7	-0.26	0.0005	0.52	0.0058	Down-Up
SYNPO2L	-0.23	0.0064	0.52	0.0062	Down-Up
SAMD8	-0.31	0.0010	0.52	0.0211	Down-Up
ZBTB10	-0.34	0.0025	0.54	0.0193	Down-Up
CNIH1	-0.17	0.0397	0.54	0.0003	Down-Up
MAP1LC3B	-0.18	0.0128	0.54	0.0049	Down-Up
CD2AP	-0.19	0.0230	0.55	0.0036	Down-Up
RLF	-0.20	0.0052	0.55	0.0165	Down-Up

ARL5B	-0.24	0.0198	0.55	0.0192	Down-Up
ZBTB6	-0.30	0.0119	0.56	0.0322	Down-Up
TOR1AIP1	-0.19	0.0220	0.57	0.0114	Down-Up
GNA13	-0.19	0.0235	0.57	0.0124	Down-Up
TWF1	-0.21	0.0162	0.57	0.0035	Down-Up
PNN	-0.19	0.0063	0.59	0.0040	Down-Up
CSGALNACT2	-0.21	0.0441	0.60	0.0001	Down-Up
RSBN1	-0.30	0.0063	0.61	0.0100	Down-Up
TSC22D2	-0.20	0.0244	0.61	0.0036	Down-Up
TENT4B	-0.21	0.0173	0.61	0.0403	Down-Up
SERP1	-0.28	0.0007	0.62	0.0000	Down-Up
CDKN2AIP	-0.26	0.0016	0.62	0.0430	Down-Up
ATF1	-0.17	0.0419	0.63	0.0358	Down-Up
SLC38A2	-0.26	0.0050	0.63	0.0415	Down-Up
FBXO28	-0.19	0.0248	0.64	0.0005	Down-Up
RNF6	-0.28	0.0013	0.65	0.0024	Down-Up
STX3	-0.19	0.0485	0.66	0.0108	Down-Up
ZNF281	-0.28	0.0004	0.66	0.0289	Down-Up
RSL1D1	-0.14	0.0283	0.66	0.0266	Down-Up
ZNF24	-0.17	0.0233	0.67	0.0160	Down-Up
MORC3	-0.16	0.0213	0.67	0.0237	Down-Up
DBF4	-0.21	0.0225	0.69	0.0172	Down-Up
RCN1	-0.18	0.0253	0.70	0.0042	Down-Up
DNAJB4	-0.24	0.0084	0.70	0.0185	Down-Up
ZXDB	-0.20	0.0445	0.72	0.0480	Down-Up
HBEGF	-0.26	0.0324	0.73	0.0032	Down-Up
BCLAF1	-0.18	0.0245	0.73	0.0000	Down-Up
ANKRD1	-0.22	0.0107	0.74	0.0141	Down-Up
NUS1	-0.20	0.0196	0.76	0.0000	Down-Up
IRS2	-0.35	0.0281	0.78	0.0466	Down-Up
CEBPG	-0.17	0.0325	0.80	0.0257	Down-Up
CCSAP	-0.29	0.0126	0.80	0.0304	Down-Up
FOSL2	-0.35	0.0218	0.81	0.0067	Down-Up
EIF5A2	-0.35	0.0112	0.82	0.0027	Down-Up
SPTY2D1	-0.18	0.0439	0.83	0.0002	Down-Up
KBTBD8	-0.28	0.0054	0.85	0.0027	Down-Up
ARL6IP1	-0.29	0.0000	0.86	0.0002	Down-Up
ELOVL4	-0.32	0.0028	0.92	0.0008	Down-Up
RND3	-0.25	0.0195	0.93	0.0043	Down-Up
PHLDA1	-0.21	0.0058	0.95	0.0037	Down-Up
CTD-3157E16.2	-0.38	0.0499	0.98	0.0178	Down-Up
HEXIM1	-0.16	0.0434	1.03	0.0001	Down-Up

ARID5B	-0.43	0.0039	1.14	0.0015	Down-Up
ATF3	-0.32	0.0324	1.15	0.0033	Down-Up
KLF5	-0.70	0.0340	1.51	0.0009	Down-Up
ANXA1	-0.31	0.0127	1.74	0.0023	Down-Up
ZNF252P-AS1	-1.25	0.0429	1.81	0.0082	Down-Up
RP11-135F9.4	-1.04	0.0377	2.08	0.0113	Down-Up
KLF10	-0.49	0.0048	2.39	0.0000	Down-Up

Supplemental Table 12. List of protein encoding genes predicted to interact with *LINC00881* with a prediction score > 14 in the RNAct database

Gene Name	UniProt Accession	Length	Protein Status	Prediction Score
NISCH	Q9Y2I1	1504 aa		18.66
ABCC9	O60706	1549 aa		17.21
DNAJC5B	Q9UF47	199 aa		17.06
CCDC180	Q9P1Z9	1646 aa		16.67
DNAJC5	Q9H3Z4	198 aa		16.36
RB1CC1	Q8TDY2	1594 aa		15.99
PABPN1	Q86U42	306 aa	Known RBP eCLIP	15.93
DCAF8L2	P0C7V8	631 aa		15.82
NACAD	O15069	1562 aa		15.81
CECR2	Q9BXF3	1484 aa		15.63
MYO15B	Q96JP2	1530 aa		15.6
BICRA	Q9NZM4	1560 aa		15.56
UNC13A	Q9UPW8	1703 aa		15.56
CC2D2A	Q9P2K1	1620 aa		15.5
ZCCHC6	Q5VYS8	1495 aa	Known RBP	15.47
DCAF1	Q9Y4B6	1507 aa		15.41
SHROOM4	Q9ULL8	1493 aa		15.28
EIF4G1	Q04637	1599 aa	Known RBP	15.24
CUX2	O14529	1486 aa		15.24
SCRIB	Q14160	1630 aa		15.24
TRIM41	Q8WV44	630 aa		15.17
ERCC6	Q03468	1493 aa		14.91
NCAPD3	P42695	1498 aa		14.6
ATAD2B	Q9ULI0	1458 aa		14.56
NES	P48681	1621 aa		14.53
SMARCA2	P51531	1590 aa		14.53
THOC2	Q8NI27	1593 aa	Known RBP	14.53
AEBP2	Q6ZN18	517 aa	Predicted RBP	14.53
SMARCA4	P51532	1647 aa		14.52
BAZ1A	Q9NRL2	1556 aa	Predicted RBP	14.49
HMGXB3	Q12766	1538 aa		14.48
OSCAR	Q8IYS5	282 aa		14.46
BAZ1B	Q9UIG0	1483 aa	Known RBP	14.44
PEG3	Q9GZU2	1588 aa		14.38
MROH2B	Q7Z745	1585 aa		14.37
PDS5B	Q9NTI5	1447 aa		14.29
LAMC3	Q9Y6N6	1575 aa		14.29
TRH	P20396	242 aa	Predicted RBP	14.27

RIMBP3C	A6NJZ7	1639 aa		14.26
RIMBP3B	A6NNM3	1639 aa		14.26
RIMBP3	Q9UFD9	1639 aa		14.26
KIF21A	Q7Z4S6	1674 aa		14.25
CADPS	Q9ULU8	1353 aa		14.19
MRC2	Q9UBG0	1479 aa		14.17
WIZ	O95785	1651 aa		14.17
CYB5RL	Q6IPT4	315 aa		14.16
WDR62	O43379	1518 aa		14.15
ARHGEF11	O15085	1522 aa		14.14
RSF1	Q96T23	1441 aa	Predicted RBP	14.12
ILDR1	Q86SU0	546 aa	Predicted RBP	14.1
TOP2A	P11388	1531 aa	Known RBP	14.03
CEP164	Q9UPV0	1460 aa		14.02
CFTR	P13569	1480 aa		14.01
FANCD2	Q9BXW9	1451 aa		14.01

Supplemental Table 13. List of PCR primer and LNA GapmeR sequences

**RT-qPCR**	
**Gene**	**Primers 5'-3'**
LINC00881	ACAGTCACGGTACTCGTTTCC
	TTCCCTGTCATGCCAGATCC
AKAP13	ACCGGAGTTCAATGCGAGTT
	CACCAGCTCCTCCTGTCAAG
HTRA1	AACTTTATCGCGGACGTGGT
	CCGGCACCTCTCGTTTAGAA
RPTOR	GGACCTCGTGAAGGACAACG
	TGACGATCACGGCGAGAATG
HDAC9	AGTAAGGATGGTGGCTGTGC
	CGGTCTCTGTCTCCTCTTGC
EFCAB13	TGGACAAGGACCTTCATACAGC
	CCTTGCCACTTTCATGTTCAGG
FBXO16	AGCACCTGGACACCCCTAAA
	TGTCAAACCATTTGCCAAGCA
TBX3	CGCTGTGACTGCATACCAGA
	GTGTCCCGGAAACCTTTTGC
GATA4	GTCCTCGCCAGTCTACGTG
	CGCCCTGGAGGTAGGACA
HAND2	ACTTCCATGGCTGGCTCATC
	ATACTCGGGGCTGTAGGACA
TBX5	AGAATATCCCGTGGTCCCCA
	GACTCGCTGCTGAAAGGACT
TNNT2	AGAGGAGGAGGAGCTCGTTT
	CTCCTTCTCCCGCTCATTCC
MYH7	CTCGCTTCGGCAGCACA
	AACGCTTCACGAATTTGCGT
SYNPO2L	ACCTGGATGAAAAGCCTCGG
	GTCTTGTAACTCCTGGCCCC
CACNA1C	GCTTATGGGGCTTTCTTGCAC
	ACTGGACTGGATGCCAAAGG
RYR2	GAGCCAGTGTCATCCACCAA
	AGGTGGCTGAAAGAATGAGCA
SMARCA4	TACAAGGACAGCAGCAGTGG
	TAGTACTCGGGCAGCTCCTT
18S	GTAACCCGTTGAACCCCATT
	CCATCCAATCGGTAGTAGCG
GAPDH	ACCACAGTCCATGCCATCAC
	TCCACCACCCTGTTGCTGTA
U6	CTCGCTTCGGCAGCACA
	AACGCTTCACGAATTTGCGT
**Chromatin Accessibility**	
**Gene**	**Primers 5'-3'**
RYR2	ATGCTTAATGGGGACCGAGG
	TGCTGAGTGGCACAGAGTTG
CACNA1C	TCAGGATTTACCACACACCG
	CATTGCCTGTTGCCTCAAACA
MYH6	TCTGTTCTTCTCTCTGCCCG
	ACCTGGTTATCCCTTCACGG
**GapmeR**	
**Name**	**Sequence 5'-3'**
LINC00881	A*G*A*A*C*A*G*G*C*A*G*G*A*G*G*T
Negative Control	A*A*C*A*C*G*T*C*T*A*T*A*C*G*C
